# A circuit for secretion‐coupled cellular autonomy in multicellular eukaryotic cells

**DOI:** 10.15252/msb.202211127

**Published:** 2023-03-01

**Authors:** Lingxia Qiao, Saptarshi Sinha, Amer Ali Abd El‐Hafeez, I‐Chung Lo, Krishna K Midde, Tony Ngo, Nicolas Aznar, Inmaculada Lopez‐Sanchez, Vijay Gupta, Marilyn G Farquhar, Padmini Rangamani, Pradipta Ghosh

**Affiliations:** ^1^ Department of Mechanical and Aerospace Engineering, Jacob's School of Engineering University of California San Diego La Jolla CA USA; ^2^ Department of Cellular and Molecular Medicine, School of Medicine University of California San Diego La Jolla CA USA; ^3^ Skaggs School of Pharmacy and Pharmaceutical Science University of California San Diego La Jolla CA USA; ^4^ Moores Comprehensive Cancer Center University of California San Diego La Jolla CA USA; ^5^ Department of Medicine, School of Medicine University of California San Diego La Jolla CA USA; ^6^ Veterans Affairs Medical Center La Jolla CA USA; ^7^ Present address: Pharmacology and Experimental Oncology Unit, Cancer Biology Department, National Cancer Institute Cairo University Cairo Egypt

**Keywords:** cellular autonomy, dose–response alignment (DoRA), epidermal growth factor receptor (EGFR), G proteins, Golgi secretion, Computational Biology, Signal Transduction

## Abstract

Cancers represent complex autonomous systems, displaying self‐sufficiency in growth signaling. Autonomous growth is fueled by a cancer cell's ability to “secrete‐and‐sense” growth factors (GFs): a poorly understood phenomenon. Using an integrated computational and experimental approach, here we dissect the impact of a feedback‐coupled GTPase circuit within the secretory pathway that imparts secretion‐coupled autonomy. The circuit is assembled when the Ras‐superfamily monomeric GTPase Arf1, and the heterotrimeric GTPase Giαβγ and their corresponding GAPs and GEFs are coupled by GIV/Girdin, a protein that is known to fuel aggressive traits in diverse cancers. One forward and two key negative feedback loops within the circuit create closed‐loop control, allow the two GTPases to coregulate each other, and convert the expected switch‐like behavior of Arf1‐dependent secretion into an unexpected dose–response alignment behavior of sensing and secretion. Such behavior translates into cell survival that is self‐sustained by stimulus‐proportionate secretion. Proteomic studies and protein–protein interaction network analyses pinpoint GFs (e.g., the epidermal GF) as key stimuli for such self‐sustenance. Findings highlight how the enhanced coupling of two biological switches in cancer cells is critical for multiscale feedback control to achieve secretion‐coupled autonomy of growth factors.

## Graphical abstract

A combined computational modeling and experimental approach is used to dissect a Golgi‐localized GTPase circuitry in eukaryotes and to understand how it empowers cancer cells to achieve self‐sufficiency in growth factor signaling.

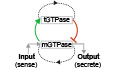

## Introduction

Self‐sufficiency in growth signaling, a.k.a, growth signaling autonomy, is the first of the six hallmarks of all cancers to have been clearly defined (Hanahan & Weinberg, 2000). While most growth factors (GFs) are made by one cell type to stimulate the proliferation of another, many cancer cells synthesize GFs to which they are responsive, creating a positive feedback signaling loop called autocrine stimulation (Fedi *et al*, 1997). Serum‐free cell culture studies squarely implicate such stimulation as key support for intracellular mechanisms that impart autonomy (reviewed in Chigira *et al*, 1990). Autonomy in cancer cells obviates dependence on extrinsic GFs, as illustrated in the case of platelet‐derived GF (PDGF) and tumor GF α (TGFα) in glioblastomas and sarcomas, respectively (Fedi *et al*, 1997). Beyond cancers, “secrete‐and‐sense” circuits that allow cells to secrete and sense the same signaling molecule are ubiquitous (Youk & Lim, 2014); these autocrine secrete‐and‐sense mechanisms do not just enable autonomy (Maire & Youk, 2015) but also generate diverse social behaviors, and recur across species (Youk & Lim, 2014).

Autocrine secretion of GFs relies on an essential, efficient, and accurate molecular machinery that constitutes a central paradigm of modern cell biology, that is, the secretory pathway (Trombetta & Parodi, 2003; Matlin & Caplan, 2017). This pathway consists of various modules that are compartmentalized on the endoplasmic reticulum (ER) and the Golgi apparatus, and are responsible for folding, processing of the post‐translational modifications, and trafficking of the proteins routed to the cell membrane (Kelly, 1985; Rothman & Orci, 1992). Nearly all these aspects of the secretory pathway have been found to be dysregulated in cancers, ranging from observed changes in Golgi shape (“onco‐Golgi”; Petrosyan, 2015), or its function (Zhang, 2021), which inspired the development of disruptors of this ER‐Golgi secretory system as anti‐cancer agents (Wlodkowic *et al*, 2009; Ohashi *et al*, 2012, 2016, 2017; Luchsinger *et al*, 2018; Núñez‐Olvera *et al*, 2020).

**Figure 1:**
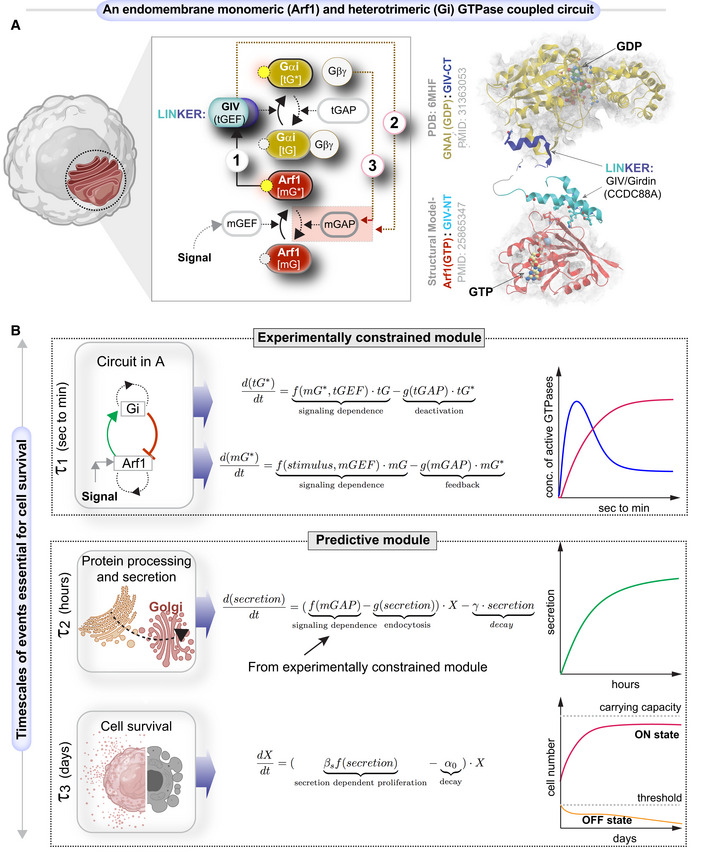
Study design and approach Schematic shows a system of two species of GTPases, mGTPases (mG), and heterotrimeric GTPases (tG), coupled by the linker protein, GIV/Girdin, that is localized on the Golgi membranes within the secretory pathway as the focus of this study. The circuit begins when active Arf1‐GTP directly binds GIV's N‐term HOOK domain, recruits GIV to Golgi membranes, and activates Gi (Lo *et al*, 2015; arrow 1). The circuit is completed when GIV's C‐terminus orchestrates two feedback loops (arrows 2 and 3), both of which are essential for the inactivation of Arf1 (Lo *et al*, 2015; Kalogriopoulos *et al*, 2019). See also Fig EV1 for illustrations detailing the sequential steps within the dynamic nature of the motif, and Movie EV1 for the visualization of these dynamic steps as a movie gif.Schematic of the mathematical model that we used to study the role of such coupling of GTPase (top panel) in autocrine secretion‐supported cell survival and proliferation (bottom panel). The modeling in the top panel is experimentally constrained, and the modeling in the bottom panel is a predictive module. This model is based on the nominal time scale of these events (left panel) and has the typical behavior shown in the right panel.

Despite these insights, the core mechanisms of cell secretion that impart cell autonomy remain poorly understood. To begin with, it is still unknown whether or not secretion is proportional to GF stimulation, and whether such secretion is sufficient to support cell survival, perhaps via closed‐loop autocrine sensing and signaling (the so‐called “secrete‐and‐sense” loop; Youk & Lim, 2014). A recent study has shown that the secretory functions of the Golgi apparatus require the unlikely coupling of two distinct species of GTPases at the Golgi (Lo *et al*, 2015; Fig 1A): one is the small or monomeric (m) GTPase Arf1 and the other is the heterotrimeric (t) GTPases Gi. GTPases serve as molecular switches that gate signal transduction: “on” when GTP‐bound (active) and “off” when GDP‐bound (inactive). The “ADP‐ribosylation factor” (Arf1; Kahn & Gilman, 1986) mGTPase is localized to the Golgi complex in mammalian cells and is essential for the secretory pathway (Stearns *et al*, 1990); it associates with Golgi membranes upon activation and is released from Golgi membranes into the cytosol upon inactivation. Such cycles of association and dissociation are regulated by Golgi‐associated, guanine nucleotide exchange factors (GEFs), and GTPase activating proteins (GAPs). Trimeric GTPases were detected in the Golgi over three decades ago (Stow *et al*, 1991; Barr *et al*, 1992), and numerous studies have provided clues that they may regulate membrane traffic and maintain the structural integrity of the Golgi (reviewed in Cancino & Luini, 2013). However, the concept of G protein activation at the Golgi and the potential impact of such activation remained controversial, primarily due to the lack of direct proof of G protein activation. The study that reported the coupling of Arf1 mGTPase and Giαβγ tGTPase provided direct evidence, the first of its kind, that the two GTPases are coupled by a linker protein, Gα‐Interacting vesicle‐associated protein (GIV; Lo *et al*, 2015). Activation of Arf1 mGTPase facilitates the recruitment of GIV on the membrane via a direct, nucleotide‐dependent interaction. Upon recruitment, GIV binds and activates Gαi serving its role as a GEF for the tGTPase, Gi. Such activation of Gi at the Golgi affects two fundamental functions of the Golgi, that is, vesicle trafficking and the structural organization of the Golgi stacks—both via modulation of Arf1 signaling. These findings firmly established that Gαi is functionally active in the Golgi.

Because tGTPases are known to primarily transduce extracellular signals (“sensing”) into intracellular signals that shape cellular responses, we asked how coupling of the two GTPases, one that guards cell secretion (Arf1) and another that gates signal sensing (Gi), may impact the cell's ability to secrete‐and‐sense. In systematically interrogating this question, we viewed the experimentally validated interactions and functions of the two GTPases and their GEFs and GAPs as a circuit of coupled GTPases. Such coupling, whose structural basis has been experimentally validated (Fig 1A‐*right*), forms a closed loop that is comprised of one forward reaction and two negative feedback loops (Figs 1A‐*left* and EV1; Movie EV1; Materials and Methods). The forward reaction is the recruitment of GIV/Girdin by active Arf1 on Golgi membranes (arrow 1). GIV is a multi‐modular cytosolic signal transducer that is a prototypical member of the family of guanine nucleotide exchange modulators (GEM) of tGTPases; GIV's GEM domain binds and activates the tGTPase Gαi, and thereby, serves as a tGEF within this circuit. One negative feedback loop involves the activation of the GAP for Arf1 (ArfGAP2/3) by GIV, which terminates Arf1 signaling (arrow 2); the other is due to GIV's role as a GEF to activate Gi and thus enhance Arf1GAP2/3, which also lead to the termination of Arf1 signaling (arrow 3). This phenomenon of co‐regulation between the two classes of GTPases maintains the Golgi shape and function, two closely intertwined processes that are regulated by Arf1. The triggers for and the consequence(s) of such co‐regulation on signal sensing/response remained unknown.

**Figure EV1:**
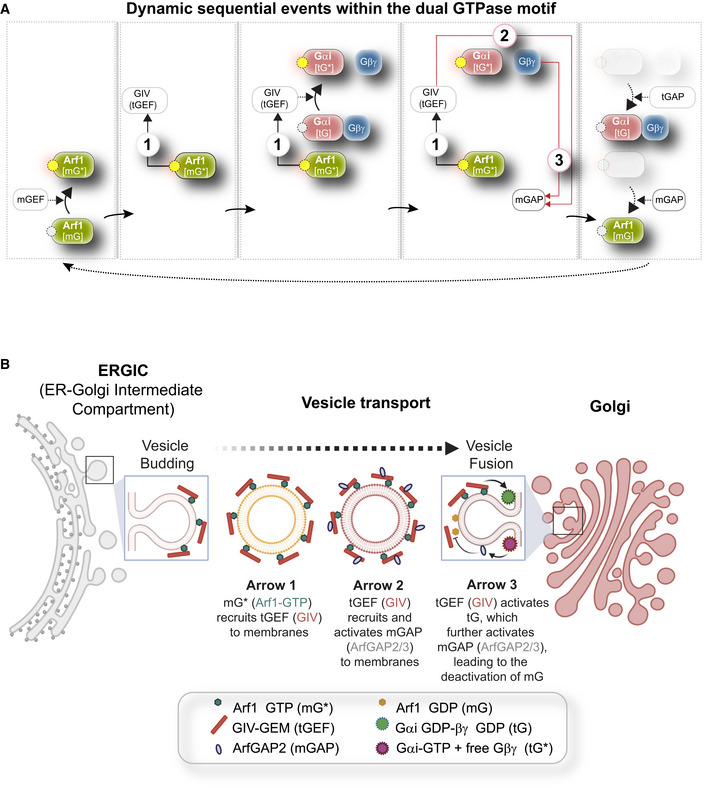
An endomembrane network motif of two species of GTPases regulates membrane trafficking through the secretory pathway and regulates Golgi functions Dynamics within the endomembrane GTPase system. Left to right panels display the deconstructed arrows denoting key molecular events/chemical reaction cascades within this system, in which, the GIV‐GEM links the monomeric (m) and trimeric (t) GTPase systems and enables the conversion of extracellular stimulus (ligand; left) into membrane trafficking events (e.g., vesicle uncoating/budding/fusion; right). The forward and feedback reactions (arrows) are numbered 1–3. See Movie EV1 for a gif of the circuit.Schematic summarizing the findings reported by Lo *et al* (2015) delineating how arrows 1–3 within the endomembrane GTPase system regulate the finiteness of Arf1 signaling for efficient secretion.

Because coupling of two species of GTPase switches, Arf1 and Gi, with feedback control is likely to generate complex, nonlinear, and non‐intuitive emergent properties, we use cross‐disciplinary approaches to dissect the role of the coupled GTPases within the secretory pathway and explore its functional significance in eukaryotic cells. Using computational biology approaches and explicit integration of experimental biology and computational methods, we also assess the impact of perturbing this motif, that is, uncoupling the GTPases. Our findings show how coupling makes secretion responsive to GFs, in particular the epidermal GF (EGF), and appears to impart secretion‐coupled autonomy.

## Results

### An integrated computational and experimental approach to dissect a Golgi‐localized GTPase circuit

We began by developing a mathematical model for this coupled circuit (Fig 1B; see Materials and Methods) and drawing clues from protein–protein interaction (PPI) network analyses, to generate testable hypotheses and validate them experimentally. The integrated approach allowed us to connect across time scales of the emergent behavior of the coupled GTPase circuit with cellular secretion, cell survival, and ultimately, secretion‐coupled survival, that is, autocrine autonomy.

The first part of the mathematical model is an experimentally constrained module for the coupled GTPases switches (upper panel in Fig 1B), where normalized Hill functions are used (Saucerman & McCulloch, 2004; Cao *et al*, 2020; see Materials and Methods for details). This approach was chosen to capture the key timescales and molecular players involved rather than focus on the specific biochemical reactions. Additionally, this approach has fewer free parameters than the traditional approach of building networks with large numbers of reactions (Getz *et al*, 2019), leading to less ambiguity in decision‐making for model development. The kinetic parameters of the coupled GTPases module were subsequently tuned to fit the time course data of GTPases in control cells and GIV‐depleted cells.

The second part of the mathematical model is a predictive module for cell secretion and secretion‐coupled cell survival (lower panel in Fig 1B). The coupling of this predictive module with the above experimentally constrained module is achieved by setting the secretion rate as a function of mGAP. The following findings allow us to make this coupling in the model: the finiteness of the Arf1 activation‐inactivation cycle was assumed to be a surrogate indicator of successful anterograde cargo movement through the compartments within the secretory pathway, that is, the ER–Golgi intermediate compartment (ERGIC) to the Golgi, because Arf1 regulates membrane traffic through a cycle of GTP binding and hydrolysis (Donaldson & Jackson, 2011); GTP binding is a pre‐requisite for membrane curvature and vesicle formation (Beck *et al*, 2008) from the donor compartment, whereas GTP hydrolysis is a pre‐requisite for vesicle uncoating (Tanigawa *et al*, 1993) and fusion with acceptor compartment. Therefore, we set the secretion rate as a function of GTP hydrolysis, a process regulated by mGAP. Except this setting for secretion rate, the model for cell secretion and cell survival/proliferation is similar to the model proposed by Hart *et al* (2014), where the kinetic parameters are from biologically plausible ranges reported previously (Adler *et al*, 2018).

We chose two different cancer cell lines to conduct the experiments: cervical (HeLa) and breast (MDA‐MB231) cancer cell lines. Our choice was guided by two reasons: (i) HeLa cells not only represent the most robust system to study Golgi structure (Ayala & Colanzi, 2016; Wortzel *et al*, 2017) and function (Rauter *et al*, 2020) but also provide continuity with prior work because all biophysical and functional studies that led to the discovery of the coupled GTPases at the Golgi were performed in this model and (ii) we and others have shown that transcriptional upregulation or post‐transcriptional activation (Dunkel *et al*, 2012; Bhandari *et al*, 2015; Sasaki *et al*, 2015) of GIV (the “linker” between the two GTPases; Fig 1A) supports several aggressive tumor cell properties (of which, many were demonstrated in MDA‐MB231 cells (Jiang *et al*, 2008; Lopez‐Sanchez *et al*, 2015; Wang *et al*, 2015; Wang *et al*, 2017; Midde *et al*, 2018; Rahman‐Zaman *et al*, 2018; Rohena *et al*, 2020)), including, invasion, matrix degradation, proliferation, and survival (Garcia‐Marcos *et al*, 2015; Aznar *et al*, 2016). Elevated expression of GIV has also been reported in a variety of solid tumors (Garcia‐Marcos *et al*, 2015; Getz *et al*, 2019), both in primary tumors (Ghosh, 2015; Ghosh *et al*, 2016b) as well as in circulating tumor cells (Barbazan *et al*, 2016; Dunkel *et al*, 2018), and has been shown to correlate with tumor aggressiveness and poor survival across cancers.

Finally, model and PPI network‐driven predictions of uncoupling the GTPases or interrupting secrete‐and‐sense autonomy were experimentally validated in the two cancer cell lines that lack GTPase coupling in the absence of the GIV linker protein.

### EGF activates Arf1 (mG*) at the Golgi and triggers the recruitment of a GEF for trimeric Giαβγ

First, we sought to model the impact of coupling on m/tGTPase signaling in response to the input signal (Fig 1A). Key events within the circuit were measured experimentally using available tools and experimental approaches (Arrows 1–3; Materials and Methods). The EGF was prioritized as an input signal because of prior evidence documenting its role in the regulation of Golgi secretion (Blagoveshchenskaya *et al*, 2008), its fragmentation during mitosis (Shaul & Seger, 2006), and most importantly, in the activation of Arf1 (Boulay *et al*, 2008; Haines *et al*, 2014, 2015).

**Figure 2:**
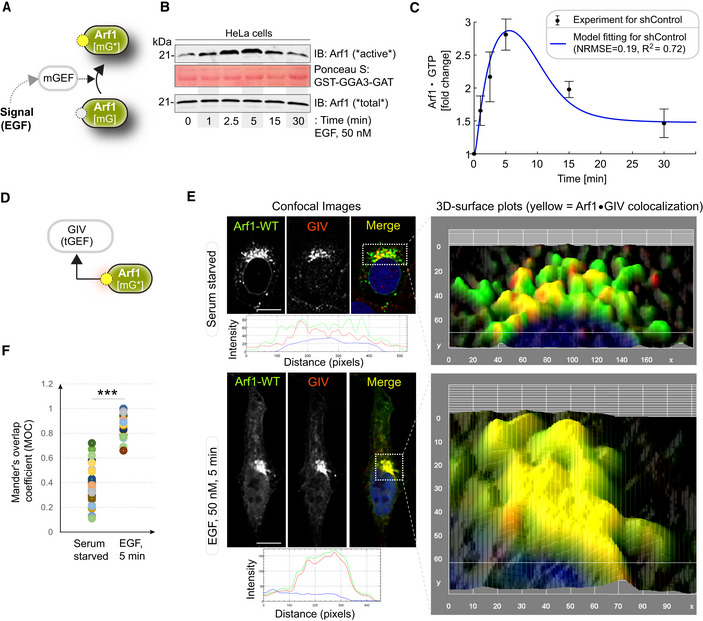
EGF activates Arf1 (mG*) and triggers the recruitment of GIV‐GEM on Golgi Schematic showing the specific step being interrogated in panels (B, C), that is, Arf1 activation under EGF stimulation.Immunoblot shows GST‐GGA‐GAT domain bound Arf1 (active; top) and total Arf1 (input lysates; bottom) from equal aliquots of lysates of HeLa cells that were stimulated with EGF for the indicated time points prior to lysis.Graphs display the model fitting for Arf1 activation dynamics. The experimentally determined Arf1 activation (in B) dynamics are displayed as black dots with error bars, representing mean ± SEM (*n* = 3 biological replicates), and numerical simulation is shown by the blue continuous line.Schematic showing the specific step being interrogated in panels (E, F), that is, recruitment of GIV‐GEM on Golgi.HeLa cells expressing Arf1‐HA were serum starved overnight (E, top) and subsequently stimulated with EGF for 5 min (E, bottom) prior to fixation with PFA. Fixed cells were stained for Arf1 (HA; green) and GIV (red) and nuclei (DAPI; blue). Panels on the left show overlay of all three stains and representative RGB plots of sections through the Arf1‐stained pixels. Panels on the right display the magnified 3D surface plots of the boxed regions in the left panels. Scale bar = 10 μm.Scatter plot shows the Mandler's overlap coefficient (MOC) for Arf1‐HA and GIV colocalization in (E) that was calculated on 13–15 cells/experiment, *n* = 3 independent experiments. *P*‐values were determined using Mann–Whitney *t*‐test: ***P = 0.0002. Source data are available online for this figure.

We measured Arf1 activity in response to EGF using an established pull‐down assay (Fig 2A and B) with the Glutathione S Transferase (GST)‐tagged GAT domain of GGA3. This domain is known to selectively bind the active GTP‐bound pool of Arf1 (Cohen & Donaldson, 2010). The levels of Arf1·GTP were increased ~3‐fold within 5 min after ligand stimulation, followed by a return toward baseline by 30 min, which we assume reflects the level of Arf1 activity in cells at a steady state (Fig 2B). These temporal dynamics were used to fit the parameters for Arf1 activity in the computational model of the circuit (blue line in Fig 2C; R^2^ and normalized RMSE are 0.72 and 0.19 respectively; see Materials and Methods and Table EV1 for model parameters). Such fitting completed the characterization of the first GTPase switch, that is, Arf1; in this case, the input is ligand stimulus (EGF) and the output is Arf1‐GTP (OUTPUT #1; mG*).

A key consequence of Arf1 activity within the coupled GTPase circuit is the first segment of the Gi activation pathway, that is, the recruitment of GIV (Fig 2D), which is not only an effector of Arf1 but also the GEF of Gi (Lo *et al*, 2015). Previous studies showed that an evolutionarily conserved region in the N‐terminal Hook domain of GIV can directly and preferentially bind to the active GTP‐bound conformation of Arf1 (Lo *et al*, 2015), revealing the structural basis of the recruitment of GIV by active Arf1 (Fig 1A‐*right*). To test whether GIV recruitment occurs in cells responding to EGF, we used immunofluorescence microscopy to observe HA‐tagged Arf1 (green; Fig 2E) and endogenous GIV (red; Fig 2E). Membrane‐colocalization of Arf1 and GIV was significantly increased within 5 min after EGF stimulation for serum‐starved cells, as determined by quantification of the Arf1‐positive Golgi regions using a Mander's overlap coefficient (MOC; Fig 2F). These results indicate that EGF‐induced Arf1 activity triggers the recruitment of GIV at the Golgi.

### EGF triggers the activation of Gi (tG*) on Golgi membranes, and then activates ArfGAP, terminating Arf1 signaling via feedback loops within the closed‐loop system

**Figure 3:**
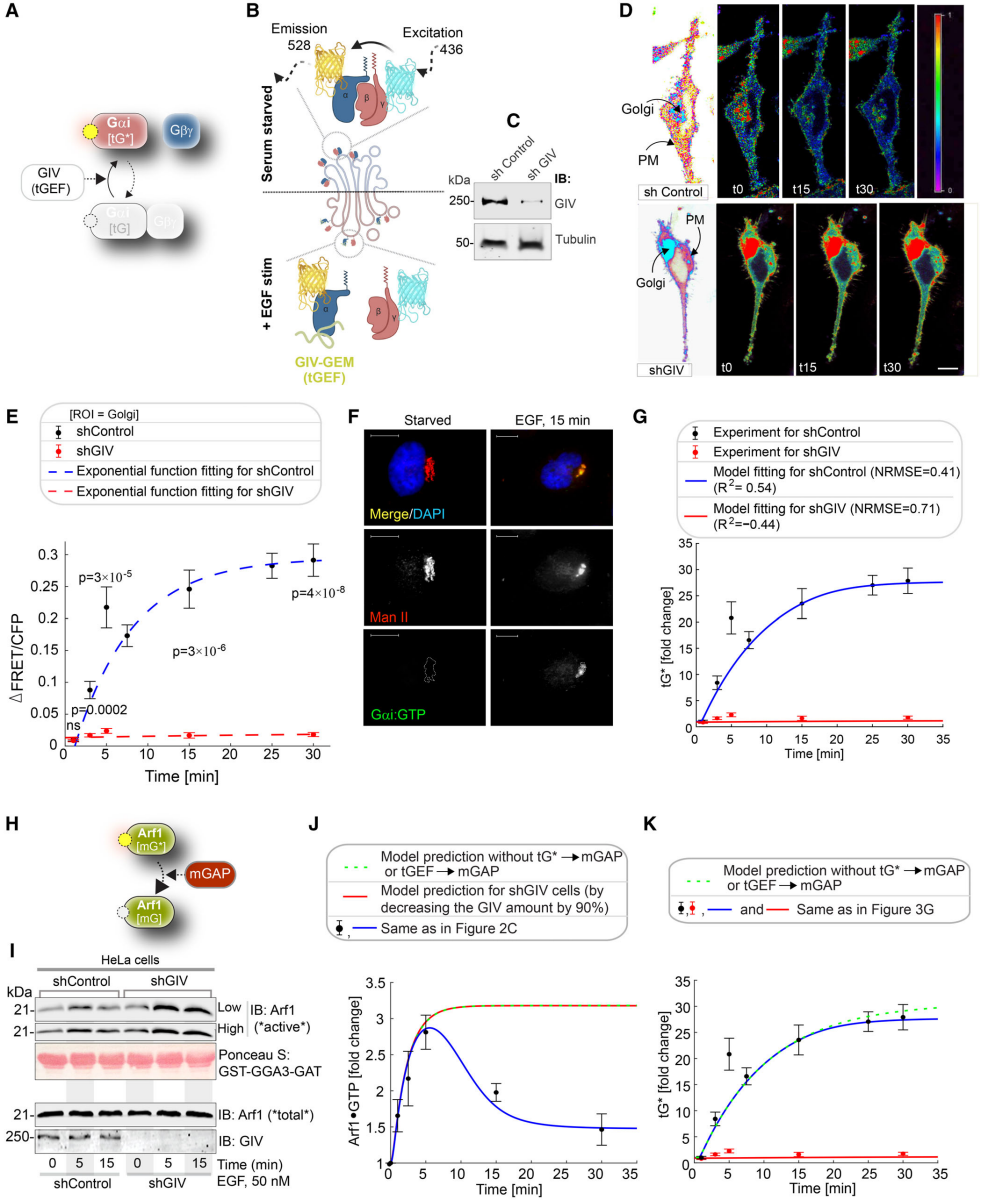
EGF triggers the activation of Gi (tG*) on Golgi membranes, activates ArfGAP, and terminates Arf1 signaling via a feedback loop ASchematic showing the specific step being interrogated in (B–G), that is, Gi activation.BSchematic describing the mechanism of the FRET Gαi activity reporter. Serum‐starved conditions are expected to have more inactive trimeric Gi, and hence show high FRET (top). Upon ligand stimulation, GIV‐dependent dissociation of trimers is expected, with a resultant loss of intermolecular FRET.CEqual aliquots (~45 μg) of whole cell lysates of control (shControl; top) and GIV‐GEM depleted (shGIV; bottom) HeLa cells were analyzed for GIV and tubulin (loading control) by immunoblotting (IB).DControl (sh Control; top) and GIV‐GEM depleted (shGIV; bottom) HeLa cells were co‐transfected with Gαi1‐YFP, Gβ1‐CFP and Gγ2 (untagged), and live cells were analyzed by FRET imaging at steady state, after being serum starved in 0.2% FBS overnight and then after stimulation with 50 nM EGF. Representative freeze‐frame FRET images are shown. FRET image panels display intensities of acceptor emission due to efficient energy transfer in each pixel. The FRET scale is shown in the inset. Golgi and PM regions of interest are indicated with arrows. Scale bar = 10 μm. See also Fig EV2A and B for free‐frame images for additional time points in control HeLa cells.EΔFRET/CFP at the Golgi (derived from D) as a function of time. The data are represented as mean ± SEM. Interrupted lines display the fitting results using exponential functions for shControl (blue) and shGIV cells (red). Data represent five regions of interest (ROIs) analyzed over the pixels corresponding to the Golgi of 3–5 cells from two independent biological experiments, that is, *n* = 8 biological replicates. *P*‐values, as determined against t0 using Mann–Whitney are displayed.FHeLa cells starved with 0.2% FBS overnight or stimulated subsequently with 50 nM EGF were fixed and stained for active Gαi (green; anti‐Gαi:GTP mAb) and Man II (red) and analyzed by confocal microscopy. Activation of Gαi was detected exclusively after EGF stimulation. When detected, active Gαi colocalizes with Man II (yellow pixels in merge panel). See also Fig EV2C and D for additional time points and stimulus. Scale bar = 7.5 μm.GModel fit for the fold change of active tGTPase (denoted as tG*). Experiment data are the fold change of ΔFRET/CFP in (D) and is shown as mean ± SEM (*n* = 8; 3–5 cells from two independent biological experiments). Continuous lines display the model simulation results after parameter fitting (See Table EV1 for parameters).HSchematic shows the step being interrogated in (I–K), that is, the termination of Arf1 signaling.IImmunoblot shows bound Arf1 (active; top) and total Arf1 (input lysates; bottom) from equal aliquots of lysates of control (sh Control) and GIV‐depleted (shGIV) HeLa cells. Cells were stimulated with EGF for the indicated time points prior to lysis. Bar graphs in Fig EV2E display the fold change in Arf1 activity normalized to t0 min. “Low” and “high” indicate exposures.J, KModel predictions of Arf1 activation dynamics (J) and Gαi activation dynamics (K) when negative feedback do not exist. The depletion of negative feedback in the model is achieved by deleting either ${\mathrm{tG}}^{*}\to \text{mGAP}$ or tGEF→mGAP (interrupted green line). These two depletion ways have no difference due to AND gate logic; please see also Fig EV3 for model predictions using OR logic. The red line in (J) was obtained by setting the GIV amount to 10% of the control cell, matching the low concentration of GIV in shGIV cells. As a reference, the experimental data (i.e., error bars in black and red) and model fit results (curves in blue and red) in Figs 2C and 3G are also displayed here, which were plotted in a same way as in Figs 2C and 3G. Source data are available online for this figure.

We next evaluated the second segment of the Gi activation pathway, that is, the ability of membrane‐recruited GIV to bind and activate the tGTPase Gi at the Golgi (Fig 3A). To be more specific, we compared the Gi activation level between control cells and GIV‐depleted cells. The Gi activation level is measured by a well‐established Förster resonance energy transfer (FRET)‐based assay (Gibson & Gilman, 2006). In this assay, the α and βγ subunits of Gi were tagged with YFP and CFP, respectively; if Gi is activated, that is, the α and βγ subunits dissociate, YFP and CFP stay far from each other, leading to low FRET (Fig 3B; See Materials and Methods for details). Besides, the GIV‐depleted cells were obtained using a short hairpin RNA to target GIV (shGIV cells; Fig 3C; See Materials and Methods for details). When we conducted FRET assays in these cells, we found that there was a significant drop in FRET (i.e., activation of Gi and trimer dissociation after EGF stimulation) at the Golgi within 5 min after EGF stimulation in control cells. Activation of Gi continued to peak by 15 min in control cells, reaching a plateau by 25–30 min (Figs 3D *top* and E, and EV2A and B for FRET at the PM). We noted that the temporal propagation of the input signal (EGF) takes ~5 min to trigger events at the Golgi, which is considerably delayed compared to most of the well‐defined EGF‐stimulated, receptor‐proximal events (Fig EV4A) which begin within ~2–5 s (Reddy *et al*, 2016). This delay is consistent with the concept of propagation delay in networks (Brent, 2009). On the other hand, in shGIV cells, such tGTPase activation was abolished due to the non‐changed FRET (Figs 3D *bottom* and E). Taken together, these results demonstrate that Gi is activated at the Golgi upon EGF stimulation and that such activation requires GIV.

**Figure EV2:**
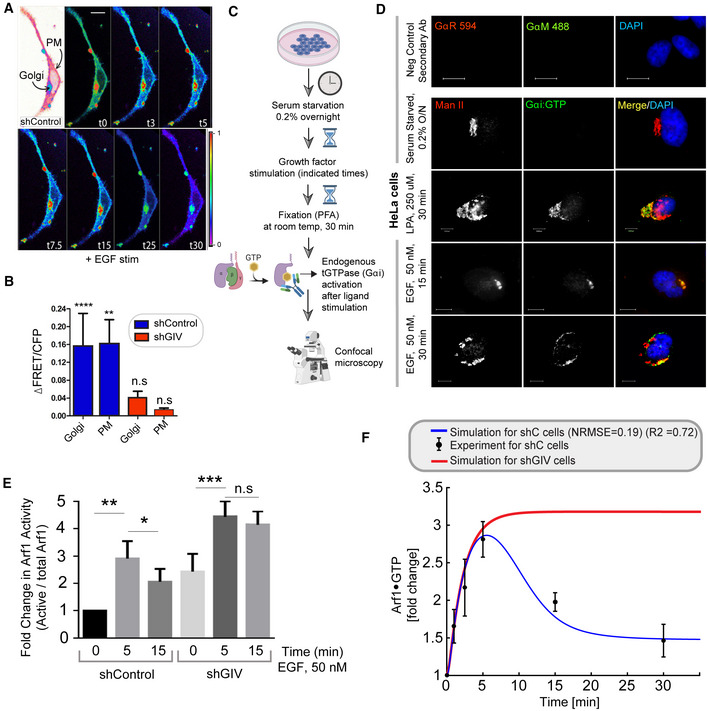
GEV‐GEM is required for Gi activation at the Golgi and for maintaining the finiteness of Arf1 signaling upon EGF stimulation FRET‐based studies were carried out in sh Control cells as in Fig 3B and C. Briefly, HeLa cells were co‐transfected with Gαi1‐YFP, Gβ1‐CFP and Gγ2 (untagged), and live cells were analyzed by FRET imaging at steady state, after being serum starved in 0.2% FBS overnight and then after stimulation with 50 nM EGF. Representative freeze‐frame FRET images are shown. FRET image panels display intensities of acceptor emission due to efficient energy transfer in each pixel. The FRET scale is shown in the inset. Golgi and PM regions of interest are indicated with arrows. Scale bar = 10 μm.Bar graphs display the change in FRET at t5 min at the Golgi and the PM regions of 3–5 cells, from four independent biological replicates. Scale bar = 7.5 μm. Results are displayed as mean ± SEM. Statistical significance was determined by student *t*‐test and the *P*‐values are depicted as: **P < 0.01; ****P < 0.0001.Schematic showing how a conformation‐specific anti‐Gαi·GTP antibody detects GTP‐bound active Gαi *in situ*.HeLa cells starved with 0.2% FBS overnight or stimulated subsequently with 50 nM EGF or 250 μM LPA were fixed and stained for active Gαi (green; anti‐Gαi:GTP mAb) and Man II (red) and analyzed by confocal microscopy. Activation of Gαi was detected exclusively after LPA/EGF stimulation. When detected, active Gαi colocalizes with Man II (yellow pixels in merge panel). Negative control (secondary antibody) staining was carried out on cells stimulated with EGF, 15 min. Scale bar = 10 μm.Control (sh Control) and GIV‐depleted (shGIV) HeLa cells that were stimulated with EGF for the indicated time points prior to lysis were assessed for Arf1 activity. Immunoblots are shown in Fig 3I. Bar graphs display the fold change in Arf1 activity normalized to t0 min that was observed in control (shControl) and GIV‐depleted (shGIV) cells. Results are expressed as mean ± SEM; *n* = 3 biological replicates; *P*‐values were determined using Mann–Whitney *t*‐test compared to t0: *P < 0.05; **P < 0.01; ***P < 0.001. Immunoblots are representative of findings from at least three independent repeats.Line graph in red displays a model‐derived simulation of Arf1 activation dynamics (mG*) in cells without tGEF (shGIV). As a reference, the results of model‐derived simulation fit to experimental data in control cells are displayed in blue, and the experimental data for Arf1 activation dynamics are shown as mean ± SEM (*n* = 3 biological replicates).

Because FRET studies require the overexpression of G protein subunits at levels much higher than relevant in physiology, we sought to validate our FRET‐based findings on endogenous Gi. To this end, we performed confocal immunofluorescence microscopy using a *bona fide* marker of the organelle, the Golgi‐localized α‐mannosidase II (Man II; Zuber *et al*, 2000), and anti‐Gαi·GTP mAb, which selectively recognizes the active (GTP‐bound) conformation of the G protein (Lane *et al*, 2008). These signals colocalized not only in EGF‐stimulated cells (Fig 3F) but also in cells exposed to other stimuli, for example, 10% serum (containing a mixture of GFs) and lysophosphatidic acid (LPA), a ligand for the GPCR, LPA‐receptor (LPAR; Fig EV2C and D). The findings confirmed that Gi is activated on Golgi membranes after GF stimulation and suggested the prevalence of this event in response to diverse stimuli.

We fitted the above experimental data by tuning the kinetic parameters. We obtained a good fit for the fold change of Gi activation in both control and shGIV cells (Fig 3G; R^2^ and RMSE, 0.54 and 0.41 for control cells; −0.44 and 0.71 for shGIV cells). The low level of GIV in shGIV cells was mimicked by decreasing the levels of expression of GIV to 10% of that in control cells (Fig 3C). Thus, the model matched the overall trend of experimental data in both cells (see Table EV1 for model parameters).

We next evaluated the feedback loops, which are critical for the “closed loop” architecture of the circuit, that is, the deactivation of Arf1 (mG*) by ArfGAP2/3 (mGAP; Fig 3H). Two negative feedback loops activate ArfGAP2/3 (arrows 2 and 3 in Fig 1A). Arrow 2 represents GIV's ability to bind and recruit ArfGAP2/3 to COPI vesicles and the Golgi membranes; failure to do so results in elevated levels of Arf1·GTP and stalled anterograde secretion in these cells (Lo *et al*, 2015). Arrow 3 represents GIV's ability to activate Gi and release “free” Gβγ; GIV's GEF function triggers this (Lo *et al*, 2015) and “free” Gβγ is a co‐factor for ArfGAP2/3. Both negative feedback loops depend on the forward reaction, arrow 1, which involves the recruitment of GIV (tGEF; Fig 1A). Using the Arf1 activity after ligand stimulation as a readout, we next measured the activity of ArfGAP2/3 in control and GIV‐depleted (i.e., shGIV) cells responding to EGF (Fig 3I). We found that Arf1 activity peaked within 5 min after EGF stimulation and rapidly reduced thereafter by 15 min in control cells but remained sustained until 15 min in GIV‐depleted cells (Figs 3I, and EV2E and F), suggesting that EGF activates both mGEFs and mGAPs of Arf1. While activation of Arf1 is brought on by mGEF(s) (the identity of which remains unknown) and achieves similar levels of activation regardless of the presence or absence of GIV, termination of Arf1 activity by mGAP (ArfGAP) requires GIV.

**Figure EV3:**
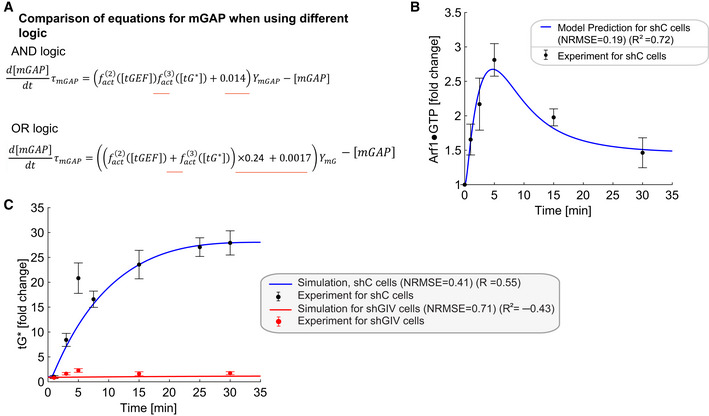
Simulations of Arf1 activation dynamics (mG*) and Gi activation dynamics (tG*) when using OR logic AComparisons of equations for mGAP when using different logics. The AND and OR logic are modeled by $f_{\mathrm{act}}^{\left(2\right)}\left(\left[\text{tGEF}\right]\right)f_{\mathrm{act}}^{\left(3\right)}\left(\left[{\mathrm{tG}}^{*}\right]\right)$ and $f_{\mathrm{act}}^{\left(2\right)}\left(\left[\text{tGEF}\right]\right)+f_{\mathrm{act}}^{\left(3\right)}\left(\left[{\mathrm{tG}}^{*}\right]\right)$, respectively. The constants 0.24 and 0.0017 ensure that the steady‐state values of all species when using OR logic are the same as those for AND logic. The differences between the two models are underlined in red in the equations.B, CSimulations of Arf1 (B) and Gi (C) activation dynamics for OR logic. The OR logic also captures the experimental data reasonably well but the AND logic is better informed experimentally. The experimental data are the same as those in Fig 3J and K.

Finally, we used the model which was fitted to the experimental data in Figs 2C and 3G to make predictions. We conducted two simulations: one in which we decreased the GIV level to simulate the Arf1 activation dynamics in the shGIV cell line (red line in Fig 3J), and one in which we deleted either arrows 2 or 3 to simulate the Arf1 and Gi activation dynamics for the uncoupled GTPase switches. Based on the experimental results before (Lo *et al*, 2015), arrows 2 and 3 are modeled by an “AND gate”‐like digital logical operation (Kime & Mano, 2003), that is, a HIGH output (ArfGAP2/3 activity, and resultant termination of Arf1 signaling) results only if both the inputs to the AND gate (arrows 2 and 3) are HIGH. We also tested the “OR” logic for the negative feedback (Fig EV3) and found the model predictions to be indistinguishable from those obtained with the AND gate. It is possible that one of these logical modes of operation is more efficient than the other under certain circumstances. For the first simulation, the simulated Arf1 activation dynamics (red line in Fig 3J) captured the sustained activation of Arf1 dynamics in shGIV cells, indicating the ability of the model to capture the experimental data. For the second simulation, the simulated Arf1 dynamics (green line in Fig 3J) is the same as that in shGIV cells, suggesting the equivalency of deleting GIV and uncoupling GTPase switches. The simulated Gi dynamics (green line in Fig 3K) is similar to (maybe even slightly higher than) that in control cells, which is consistent with the fact that the feedback loops have no effect on Gi. Thus, negative feedback within the “closed‐loop control” (CLC) exerts a significant effect on the mGTPase (Arf1) and little or no effect on the tGTPase (Gi).

### Coupled GTPases are predicted to enable high‐fidelity concordant response to EGF

**Figure 4:**
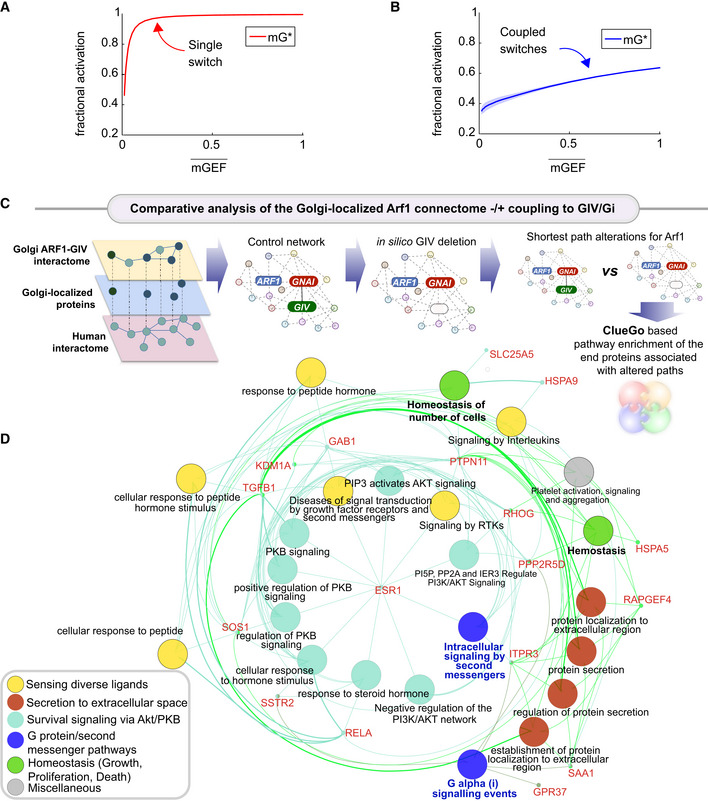
The predicted impacts of uncoupling the coupled switches A, BFractional activations of mGEF vs. active Arf1 (mG*) for the single switch (A; mG alone) and coupled switches (B; mG and tG). We perform stochastic simulations in the presence of noise in EGF (see Materials and Methods for details). The mean and the standard deviation (SD) of species are evaluated at steady states based on 1,000 repeated independent simulations of ODEs in the presence of noise. $\bar{\text{mGEF}}$ denotes the mean of mGEF; the shading shows the SD. The dimensionless EGF concentrations in the simulations are obtained through normalization, that is, dividing the EGF concentration by 217.4 nM (=50 nM/0.23). In all simulations, noise is introduced only in stimulus (i.e., EGF).
C, D
A comparative analysis of the Golgi‐localized Arf1 (mG) connectome with/without coupling to GIV (tGEF) and Gi (tGTPase). Workflow (C) shows how the list of Golgi‐localized Arf1 and GIV interacting proteins (Appendix Fig S1 and Dataset EV1) were used as “seeds” to construct a PPI network from the STRING database to fetch the linking nodes to connect the seed proteins. The network was then perturbed by *in silico* deletion of GIV, followed by a topological analysis of how such perturbation impacts the shortest paths associated with Arf1 to all other nodes in the network (see Materials and Methods). A network representation (D) using the ClueGo algorithm of the cellular processes associated with the end proteins that were most frequently encountered in the most impacted shortest paths associated with Arf1 (listed in Appendix Fig S2 E). The deleted or newly added shortest paths were only considered using the differential network approach (see Materials and Methods). The key in the lower left corner displays the color code of various overarching themes encountered in the network.

**Figure EV4:**
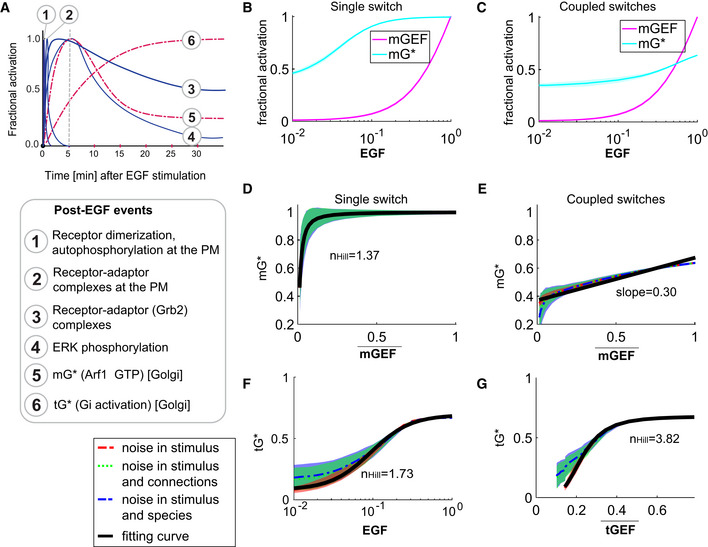
Coupled switches enable the alignment of endomembrane responses (Arf1 and tG* activities) to the dose of an extracellular stimulus APublished dynamics of EGF‐stimulated events that are initiated at the PM (blue, continuous) or experimentally determined dynamics of events at the Golgi confirmed here (red, interrupted) are compared. The interrupted line at 5 min provides a reference frame for the observed peak Arf1 activity upon EGF stimulation.B, CDose responses of fractional activations of mGEF and active Arf1 (mG*) for the single switch (B; mG alone) and coupled switches (C; mG and tG). We perform stochastic simulations in the presence of noise in EGF (see Materials and Methods for details). The mean and the standard deviation (SD) of species are evaluated at steady states. The dimensionless EGF concentrations in the simulations are obtained through normalization, that is, dividing the EGF concentration by 217.4 nM (=50 nM/0.23). In all simulations, noise is introduced only in stimulus (i.e., EGF).D, EThe same plots as in Fig 4A and B but in the presence of three different types of noise: noise in stimulus (shown in red), noise in stimulus and connections simultaneously (shown in green), and noise in stimulus and species simultaneously (shown in blue; see Materials and Methods for details). Data are shown as mean values (dashed curves), with the shading showing the SD. The black curves are fitting curves (*r* ^2^ > 0.95) for red dashed curves (see Materials and Methods for the calculations of *r* ^2^ and $n_{\text{Hill}}$).F, GThe fractional activations of tGTPase (tG*) as a function of EGF (F) or tGEF (G). The plots are generated in a similar way as in (F, G). $\bar{\text{tGEF}}$ denotes the mean of tGEF. *r* ^2^ > 0.95 for all fitting curves. The EGF‐tG* relationship shows a Hill coefficient of 1.73, and the tGEF→tG* switch (switch #2) shows a dose–response behavior close to the saturation regime of an ultrasensitive curve (*n*_*Hill*_ = 3.82).

To gain insights into how coupling impacts information transduction, we compared the dose–response alignment (DoRA) performance between the coupled and uncoupled GTPase circuits. Typically, DoRA, referring to the close match of the receptor occupancy and the downstream response no matter what the stimuli level is (Andrews *et al*, 2016), is believed to improve information transduction, since the downstream molecules reflect the receptor occupancy faithfully. We regarded the mGEF as an alternative to the receptor because it serves as the first input to the coupled circuit via its ability to trigger the activation of the mGTPase switch. Therefore, a close match of dose–response curves of mGEF and mG* is equivalent to the linear relation between mGEF and mG*. Using the model that has been fitted to the data in Figs 2C and 3G, we simulated the steady‐state value of mG* and mGEF over a wide range of stimuli and then plotted the fractional activation of mG* for a given mGEF activity to observe the linearity. The misalignment in the case of a single switch is evident; a single Arf1 switch displays hyperresponsiveness, in that, max mG* is achieved even with minimal mGEF activity (Fig 4A). In the case of coupled switches, similar plots of fractional activation of mG* for a given mGEF activity show DoRA with an unexpected linear relationship (Fig 4B). These results also hold in the presence of noise, such as noise in EGF stimulus and the intracellular noise [simulated within the concentrations of the different species (nodes) and the connections between them (arrows)] (see Materials and Methods and Fig EV4). These results suggest that coupled switches exhibit higher fidelity in information transduction than uncoupled switches. Although unexpected for a GTPase switch, this finding is consistent with what is generally expected in a closed loop with negative feedback (Becskei & Serrano, 2000; Åström & Murray, 2021).

### Coupled GTPases are predicted to support secretion that is linked to autocrine signaling and survival

To understand the impact of uncoupling of the GTPase circuit on Arf1‐dependent secretory functions of the Golgi, we carried out: (i) PPI network analysis and (ii) the mathematical modeling based on ordinary differential equations (ODEs).

To restrict the Arf1 interactome to the Golgi, we first extracted a Golgi‐annotated subcellular localization network of high‐confidence GIV and Arf1 correlators, based on a proximity‐dependent biotinylation map of a human cell (Go *et al*, 2021; Appendix Fig S1). Next, the list of Golgi‐localized proteins was expanded by incorporating the GIV interactors from BioGRID (Oughtred *et al*, 2021; Appendix Fig S2A and B). Arf1's connectivity in the coupled network (in which Arf1·GIV·Gi interactions were intact) was compared against an uncoupled network created *in silico* by the removal of GIV from the network (Appendix Fig S2C). Network analysis (see the workflow in Fig 4C; and as detailed in Materials and Methods) showed that Arf1's connectivity with many proteins (“nodes”) and pathways was altered in the uncoupled state (listed in Appendix Fig S2D–G). These altered pathways share three key themes: (i) “sensing” of diverse ligands/stimulus, for example, GFs, peptide and steroid hormones, and cytokines (yellow nodes in Fig 4D), (ii) “secreting” proteins to the extracellular space (red nodes in Fig 4D), and (iii) “survival” signaling via the PI3K‐Akt pathways (teal nodes in Fig 4D). As anticipated in the absence of GIV, Gi, and second messenger signaling (blue nodes in Fig 4D), cellular homeostasis and cell number (green nodes in Fig 4D) were predicted to be impacted. These findings suggest that removing GIV may impact secretion that is critical for auto/paracrine sensing/signaling, which maintains cell number via balanced proliferation and/or death.

**Figure 5:**
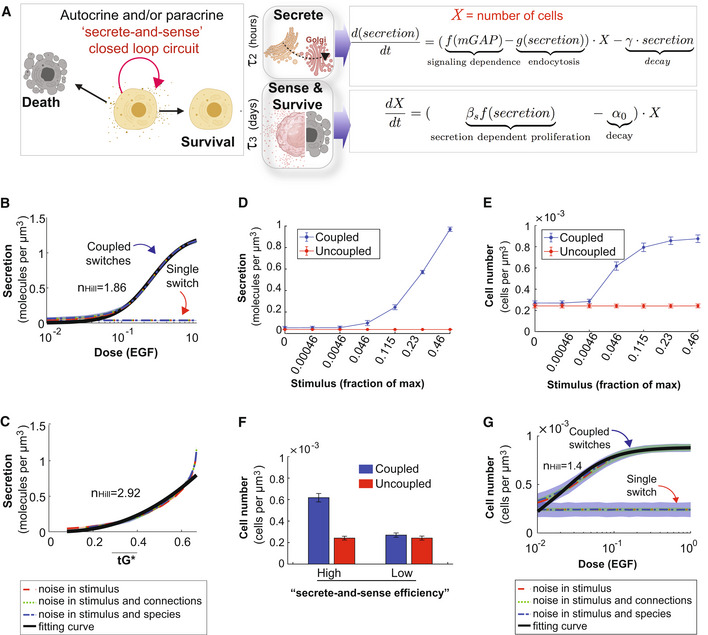
Coupled GTPases are predicted to support secrete‐and‐sense autonomy and maintenance of cell number ASchematic of the key features of the auto/paracrine loop that we hypothesize is regulated by the coupled GTPase circuit (left) and the corresponding phenomenological models to capture these key effects (right).B, CModel prediction for secretion as a function of stimulus in cells with coupled and uncoupled GTPases. Noise is introduced into the system in a similar way as described in Fig EV4D–G. *r* ^2^ > 0.99 in (B); *r* ^2^ > 0.94 in (C).D, EThe secretion (D) or the cell number (E) as a function of stimulus in coupled and uncoupled switches. The stimulus = 0, 0.00046, 0.0046, 0.046, 0.115, 0.23, and 0.46 correspond to varying doses of EGF in simulations, ranging from 0, 0.1, 1, 10, 25, 50, and 100 nM, respectively. The error bar denotes SD based on 1,000 repeated independent simulations of ODEs when noise is in the stimulus and connections.FThe bar plot depicts cell numbers achieved by cells with either coupled or uncoupled switches, at different levels of stimulus. For the first two bars, the height and error bars are the mean and SD of cell number when stimulus = 0.046 in (E), respectively. For the last two bars, the height and error bars are the mean and SD of cell number when stimulus = 0 in (E), respectively.GRelation between cell number and EGF in the presence of noise, which was introduced in a similar way as described in Fig EV4D–G. *r* ^2^ > 0.95.

We next used computational modeling approaches to interrogate how coupled (CLC) vs. uncoupled (open loop) GTPase systems at the Golgi impact cargo secretion and cell number upon sensing GF stimulus (Fig 5A). However, unlike m/tG* activity assays (which happen in a second to minute), cell secretion may begin within minutes but is measured in hours, and their impact on cell number requires a longer time scale (several hours and even days). The secretion function was predicted to show an ultrasensitive response (*n*_*Hill*_ = 1.86) as a function of the stimulus (i.e., a given dose of EGF) when the two GTPase switches are coupled; it was predicted to be reduced in the absence of coupling (Fig 5B–D). Though the secretion response is a constant in the absence of the coupling, it sets the baseline for EGF‐to‐Arf coupling and thus supplies a platform for the comparison with the coupled GTPases system. Intriguingly, secretion in the coupled state shows different responses for most ranges of Gi activity (tG*; Fig 5C), indicating faithful information transduction between Gi and secretion. Furthermore, the cell number is higher for the coupled vs. the uncoupled cells (Fig 5E and Appendix Fig S3). To specifically analyze the impact of secrete‐and‐sense autocrine autonomy, we carried out the simulations under restrictive growth conditions. These simulations under restrictive growth conditions revealed that cells with coupled switches display a higher cell number compared to the cells with uncoupled switches only when the secrete‐and‐sense loop is highly efficient; this advantage is lost if the loop is abolished (Fig 5F). That coupling of GTPases that is required for maintaining cell numbers was reproduced using EGF as the stimulus (Fig 5G), providing continuity with prior model‐derived predictions. We also confirmed that the system and the conclusions are not only robust to biological noise (Fig 5B, C and G) but also robust to the variations in the kinetic parameter (Appendix Fig S4).

### GTPase coupling by GIV is required for time and dose‐dependent secretion of diverse cargo proteins

**Figure 6:**
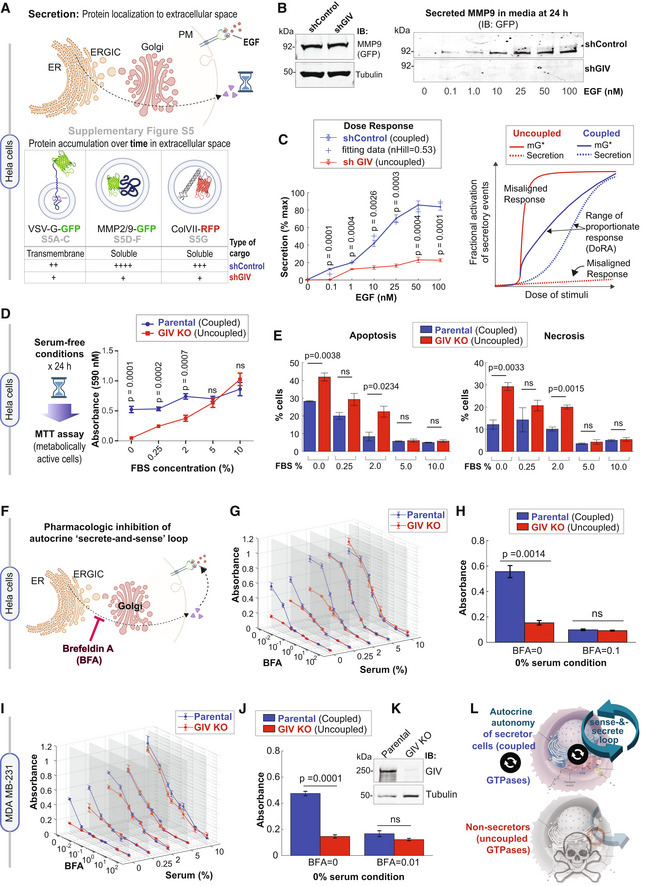
Coupling of GTPases by GIV is required for growth factor‐independent cell survival that relies upon autocrine secretion ASchematic summarizes the findings showcased in Fig EV5, which investigate the secretion of diverse cargo proteins [temperature‐sensitive (ts) VSV‐G, MMP2/9, and ColVII], as determined by their accumulation in extracellular space over time after the stimulus (EGF or serum). For each cargo tested, compared to cells with GIV (shControl), ligand‐stimulated secretion was impaired in cells without GIV (shGIV).BImmunoblots showing intracellular (left) and secreted (in the media; right) GFP‐MMP9 at 24 h after stimulation with varying doses of EGF. Tubulin, used as a loading control, confirms the presence of a similar number of plated cells in the assay.C
*Left*: Graph displays experimentally determined secretion of GFP‐MMP9 in response to varying doses of EGF in control (shControl) and GIV‐depleted (shGIV) HeLa cells (as in B), and quantified by band densitometry. Results are expressed as mean ± SEM; *n* = 3 biological replicates. *P*‐values were determined by a two‐sided unpaired *t*‐test. Right: Schematic diagram of dose responses (mG* and secretion) for the single switch and coupled switches. Coupled switches stretch the range of proportionate responses. Single mG switch results in misaligned responses. DoRA, dose–response alignment.DLeft: Schematic summarizing the colorimetric assay used here to determine the number of metabolically viable cells. Right: The graph displays formazan absorbance expressed as a measure of cell viability from the HeLa cells (*Y*‐axis) cultured at varying conc. of serum (*X*‐axis). Results are expressed as mean ± SEM; *n* = 3 biological replicates. *P*‐values were determined by a two‐sided unpaired *t*‐test.EBar graphs display the % apoptotic (*left*) or necrotic (*right*) control (parental) and GIV‐depleted (GIV KO) HeLa cells after 24 h growth in varying concentrations of serum, as assessed by annexin V staining and flow cytometry. See also Appendix Fig S5 A–C for dot plots and early and late apoptotic fractions. Results are expressed as mean ± SEM; *n* = 3 biological replicates. *P*‐values were determined by a two‐sided unpaired *t*‐test.FSchematic showing the rationale for and mechanism of action of fungal toxin, BFA, for interrupting the secrete‐and‐sense autocrine loop in cells.
G, H
Control (parental) and GIV‐depleted (GIV KO) HeLa cells grown in different concentrations of serum (FBS%) were treated or not with varying concentrations of BFA (μM) as indicated. Line graphs in 3D (G) depict the formazan absorbance expressed as a measure of cell viability from the HeLa cells in various conditions tested. Bar graphs (H) depict the cell number in serum‐free growth conditions that are supported exclusively by the autocrine secrete‐and‐sense loop (without BFA; BFA = 0.0 μM) or when such loop is interrupted (BFA = 0.1 μM). Results are expressed as mean ± SEM; *n* = 3 biological replicates. Statistical significance was determined by one‐way ANOVA.
I–K
Control (parental) and GIV‐depleted (GIV KO) MDA MB‐231 cells grown in different concentrations of serum (FBS%) were treated or not with varying concentrations of BFA (μM) as in (G, H). Line graphs in 3D (I) depict the formazan absorbance expressed as a measure of cell viability from the MDA MB‐231 cells in various conditions tested. Bar graphs (J) depict the viability of the MDA MB‐231 cells in serum‐free growth conditions that are supported exclusively by the autocrine secrete‐and‐sense loop (without BFA; BFA = 0.0 μM) or when such loop is interrupted (BFA = 0.1 μM). Results are expressed as mean ± SEM; *n* = 3 biological replicates. Statistical significance was determined by one‐way ANOVA. Immunoblots (K) of equal aliquots of whole cell lysates confirm the depletion of GIV compared to tubulin (loading control). See also Appendix Fig S5 D–H for dot plots and early and late apoptotic fractions. Results are expressed as mean ± SEM; *n* = 3 biological replicates.LSummary of conclusions of this work. *Top*: Coupling of GTPases within the secretory pathway enables dose–response alignment of secretion to stimulus, which appears to be essential for “secrete‐and‐sense” autocrine autonomy in cancer cells. *Bottom*: Uncoupling of the GTPases within the secretory pathway disrupts such autonomy and leads to cell death. Source data are available online for this figure.

**Figure EV5:**
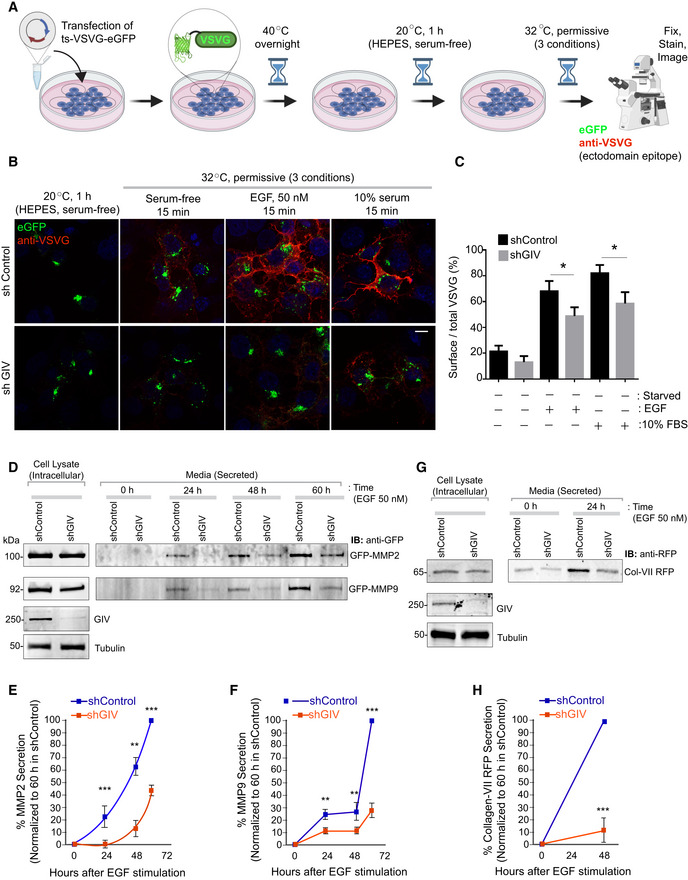
GIV‐GEM is required for EGF‐triggered secretion of diverse cargo proteins through the Golgi compartment ASchematic shows the basis for measuring secretion of transmembrane cargo protein, ts‐VSVG‐eGFP. This temperature‐sensitive mutant VSVG is retained in the ER at 40°C, at the Golgi at 20°C, and moves out of the Golgi to the PM when shifted to 32°C (Gallione & Rose, 1985). When visualized with immunofluorescence under non‐permeabilized conditions, a VSVG‐ectodomain targeting antibody selectively detects PM‐localized cargo, whereas a GFP tag allows the visualization of total VSVG in the cell.B, CControl (sh Control; top) and GIV‐depleted (shGIV; bottom) Cos7 cells were transfected with tsO45‐VSVG‐GFP and cells were shifted to 40°C for O/N and then incubated at 20°C for 1 h in HEPES buffered serum‐free media followed by temperature shift at 32°C for 15 min in plain DMEM and or containing 50 nM EGF or 10% serum. Coverslips were fixed and stained with VSVG‐ectodomain‐specific monoclonal antibody (red). Representative images are shown in (B). Scale bar = 10 μm. Green fluorescence indicates total VSVG expression whereas red fluorescence shows the surface‐localized pool of VSVG. Bar graphs in (C) display the Red:Green intensity ratio indicative of the fraction VSVG that is secreted to the cell surface. Results are expressed as mean ± SEM; *n* = 3 biological replicates; *P*‐values were determined using Mann–Whitney *t*‐test compared to t0: *P < 0.05.D–HControl (sh Control) and GIV‐depleted (shGIV) HeLa cells were analyzed for EGF‐stimulated secretion of three soluble cargo proteins, MMP2 (D, E), MMP9 (D, F), and Collagen‐Vii RFP (G, H), as detected from the supernatants at indicated time points after EGF stimulation. Results are expressed as mean ± SEM; *n* = 3 replicates; *P*‐values were determined using Mann–Whitney *t*‐test compared to t0: **P < 0.01; ***P < 0.001. Immunoblots are representative of findings from at least three independent repeats.

We next sought to experimentally validate the predicted impact of uncoupling on cell secretion by studying the time‐dependent secretion of a few well‐established transmembrane and soluble cargo proteins. We began with the transmembrane cargo, vesicular stomatitis virus G protein (VSVG) using the well‐characterized GFP‐tagged VSVG‐tsO45 mutant (Gallione & Rose, 1985). This mutant VSVG is retained in the ER at 40°C, and accumulates in Golgi stacks at a 20°C temperature block, from where it escapes to the PM at a permissive 32°C (Fig EV5A). Considerable VSVG accumulated in the Golgi region in both control and GIV‐depleted cells under serum‐starved conditions at 20°C. EGF or serum stimulation was permissive to the transport of the VSV‐G protein to the PM in control cells at 32°C, but such transport was significantly diminished in GIV‐depleted cells (Fig EV5B and C). Similar results were observed also in the case of EGF‐stimulated secretion of three separate soluble cargo proteins, MMP2, MM9 (Fig EV5D–F), and Collagen (Fig EV5G and H); these cargo proteins were chosen because of GIV's published role in ECM degradation during cancer metastasis (Rahman‐Zaman *et al*, 2018) and tissue fibrosis (Lopez‐Sanchez *et al*, 2014). These findings show that the secretion of diverse proteins in response to GFs is blunted in GIV‐depleted cells with uncoupled GTPases (Fig 6A).

We next asked if the DoRA predicted earlier in the case of Arf1 activity (Fig 4B) translates into a similar alignment in the case of cell secretion. We analyzed the efficiency of secretion of one of the three cargo proteins, MMP9, from control and GIV‐depleted cells responding to a range of EGF concentrations for 24 h (Fig 6B). Quantitative immunoblotting confirmed that dose‐dependent secretion was observed in the case of control cells (coupled GTPases) but not in GIV‐depleted cells (uncoupled GTPases; Figs 6B and C *left*). We conclude that the DoRA of Arf1 activity indeed translates into DoRA of cell secretion in cells with coupled GTPases; by contrast, a misaligned Arf1 activity (hyperresponsive; Figs 4A and 6C, right) translates into misaligned secretion (hyporesponsive; Fig 6C, right) in cells with uncoupled GTPases.

### GTPase coupling by GIV is required for cell survival that relies upon autocrine secretion

We next assessed by MTT assays the total number of metabolically active cells that develop self‐sufficiency in GF signaling, that is, survive in GF‐free conditions (0% serum; Fig 6D, *left*). The number of cells in serum‐free or low‐serum conditions was significantly higher in the presence of GIV (parental HeLa cells; coupled) than in the absence of GIV (GIV‐KO cells; uncoupled; Fig 6D); this survival gap closed at higher serum concentrations (see 10% Fetal Bovine Serum (FBS), Fig 6D). Reduced cell number in GIV‐KO cells in the low/no serum conditions was associated with a concomitant increase in cell death via apoptosis and necrosis (Fig 6E and Appendix Fig S5A–C). We then sought to validate the results of the simulations in growth‐restrictive conditions which showed that interrupting the coupled GTPase circuit at the Golgi will reduce cell numbers (Fig 5F). We analyzed the number of metabolically active cells with (coupled) or without (uncoupled) GIV across a range of serum conditions and varying concentrations of the mycotoxin Brefeldin A (BFA), a well‐known tool to inhibit secretion via its ability to inhibit Arf1 activation (Prieto‐Dominguez *et al*, 2019; Fig 6F). We made three observations: (i) cells with coupled circuits have a significant survival advantage in serum‐restricted conditions (see 0–2.0% FBS; Fig 6G); (ii) that advantage depends on sensing what the cells secrete because blocking secretion with BFA also eliminates such advantage (Fig 6H); and (iii) survival in the presence of serum (5–10%) is similar for both “coupled” and “uncoupled” cells, implying non‐secreting cells with uncoupled circuits can survive if they can “sense” stimuli that they did not generate (e.g., serum ~5–10% range; Fig 6G).

To avoid overreliance on a single model system (i.e., HeLa cells), we generated a second model, GIV‐depleted MDA MB‐231 cells (using CRISPR/Cas‐mediated genome editing, see Materials and Methods) and sought to reproduce key findings (Fig 6I–K). As in the case of HeLa cells, the survival advantage of MDA MB‐231 cells with coupled circuit (with GIV, Parental cells) over those with uncoupled circuit (GIV KO) was observed exclusively in low/no serum conditions (see 0–2.0% FBS; Fig 6I) and blocking secretion with BFA eliminates such advantage (Fig 6J). Reduced cell survival in cells without GIV (uncoupled state) was associated with higher early and later apoptosis and necrosis (Appendix Fig S5D–H).

These findings show that the coupled GTPase circuit is required for cell survival that is supported exclusively by autocrine secretion (i.e., independent of external GFs), and by that token, essential for a functional autocrine “secrete‐and‐sense” loop (Fig 6L, *top*). Interrupting the coupled GTPase circuit at the Golgi appears to disrupt the “secrete‐and‐sense” loop and abrogate cell survival that is supported by such secretion (Fig 6L, *bottom*). Because “secrete‐and‐sense” loop is a key feature of cellular autonomy (Youk & Lim, 2014; Maire & Youk, 2015), taken together our findings show that the coupled GTPase circuit in the cell's secretory pathway may be critical for autocrine autonomy.

### GTPase coupling supports self‐sufficiency in GF signaling

**Figure 7:**
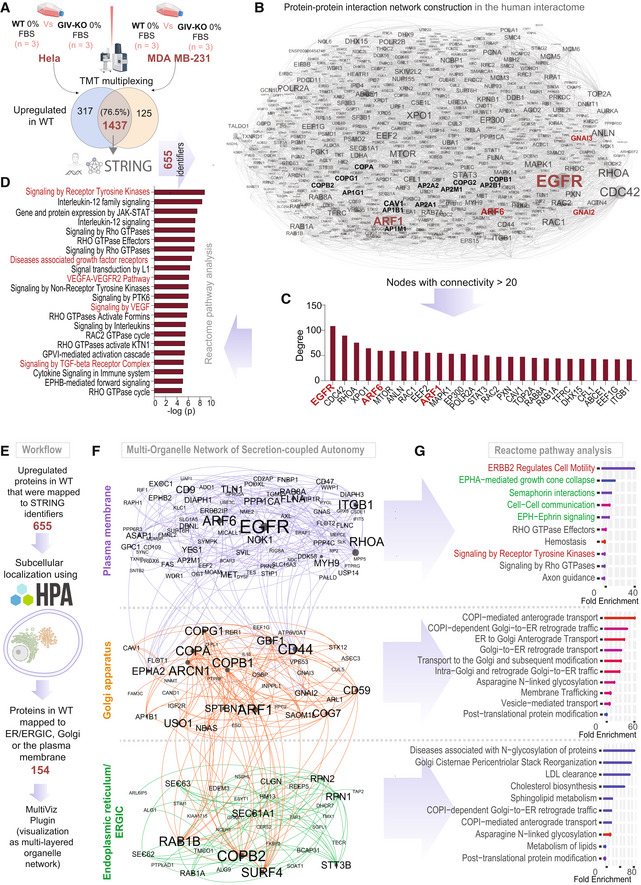
Differential proteomics of autonomy enabled vs. disabled MDA MB‐231 and HeLa cells Workflow for comparative proteomics on autonomy enabled vs. disabled cells by tandem mass tag (TMT) multiplex technique followed by mapping of upregulated proteins in WT cells using the STRING database (see Materials and Methods).A protein–protein interaction (PPI) network shows the interactions between upregulated mapped proteins in WT cells. Node and font sizes correlate positively with the degrees of connectivity.Bar plot shows the degree distribution of highly connected (degree > 20) nodes in the PPI network in (B).Reactome pathway analysis of the pathways enriched in the most connected proteins in (C). Red = pathways associated with growth factor signaling.Workflow for the construction of a multi‐organelle network of autonomy‐enabled cells using subcellular localization of upregulated proteins in the WT cells.Visualization of a multi‐organelle network of proteins that partake in secretion‐coupled autonomy across three compartments, the plasma membrane, the Golgi, and the ER/ERGIC.Reactome pathway analyses of the pathways enriched within the three organelles in (F). Red pathways associated with RTK/EGFR signaling and Green pathways associated with multi‐cellular cell–cell communication in the plasma membrane.

To discern the nature of the pathway/processes whose autocrine autonomy is supported by the coupled GTPases, we analyzed HeLa and MDA MB‐231 cells with coupled (WT) or uncoupled (GIV KO) circuits by tandem mass tag (TMT) proteomics. The studies were carried out in serum‐free/restricted conditions (Fig 7A) to maximally enrich the proteome that supports auto‐/paracrine secretion‐coupled sensing. To our surprise, the majority (76%; 1,437 proteins, including EGFR; see the complete list in Dataset EV2) of the differentially upregulated proteins (DEPs) in the two WT cell lines overlapped (despite the vast differences between HeLa and MDA MB‐231 cell lines in origin, genetics, and nearly every other possible way). This suggests that the presence or absence of GTPase coupling via GIV may impact both cells similarly. The interactions between the DEPs were fetched from the STRING database to build a PPI network, in which we found several major coat proteins (AP1, AP2, COP, and CAV), the monomeric GTPases (Arfs, Rabs, Rho, CDC42, and Rac1) and trimeric GTPases (GNAI; Fig 7B). A connectivity analysis revealed that EGFR and the Arfs are some of the most highly connected nodes in the interactome (Fig 7C). A reactome pathway enrichment analysis confirmed that the most highly connected proteins primarily engage in a variety of GF signaling pathways (Fig 7D).

Because protein functions are determined by subcellular localization, we sought to map the DEPs that are upregulated in the WT cells based on their subcellular localization. To this end, we used a Human Cell Atlas‐supported explorer platform (that uses a large collection of confocal microscopy images of patterns of subcellular localization of human proteins) to include the proteins that localize to three organelles, the Golgi, ER/ERGIC, and the PM (Fig 7E; see Materials and Methods). Visualization of the DEP‐derived PPI networks as multi‐layered networks that are comprised of intra‐ and inter‐organelle interactions (Fig 7F) revealed greater insights. As expected, reactome pathway analysis of the ER/ERGIC and the Golgi interactomes showed an enrichment of protein processing and secretory processes, respectively, and the PM‐localized interactome showed an enrichment of GF signaling (Fig 7G). The PM‐localized interactome also showed an enrichment of cell–cell contact and contact‐dependent signaling pathways (such as Semaphorins and the Eph/ephrin system; green; Fig 7G), which enable cell–cell coordination in multicellular eukaryotes. These findings indicate that the coupled GTPase system supports a network of proteins that primarily enable secretion‐coupled GF sensing and thereby, growth signaling autonomy.

## Discussion

The major novelty we report here is the creation of an experimentally constrained multi‐timescale model for cell survival that relies on GF‐responsive cell secretion. One major consequence of such a phenomenon is autonomous growth/survival in the absence of external GFs. We formally define the molecular basis for such autonomy and demonstrate the consequences when it is manipulated/perturbed. The insights and models derived from this study are expected to inform and impact at least three fields, that is, signal transduction, cell secretion, and cancer cell biology in the following ways.

In the field of signal transduction, emergent properties of ectomembrane signaling circuits at the PM have been identified using mathematical modeling based on ODEs; however, none thus far have coupled the events at the ectomembrane to the events in the cell's interior, that is, the endomembrane of organelles. Our study experimentally validated a Golgi‐localized natural coupling between the two GTPase switches with exquisite feedback control that enables linear activation of Arf1 in response to EGF, which in turn enables the Golgi to mount a response (protein secretion) that is proportionate to the stimulus (sensed at the PM) and robust to noise. The model reveals two notable features: First we show that the CLC system generated DoRA, enabling a linear increase in Arf1/mG* activation and protein secretion. Such DoRA has been described in several major receptor‐initiated signaling cascades at the PM (from the pheromone response system in yeast to the Wnt → βCatenin, TGFβ→SMAD2/3 and EGFR→MAPK cascades in mammals; Andrews *et al*, 2016), but never in endomembrane GTPases. Because a linear DoRA maximally preserves any information during its propagation (Andrews *et al*, 2018), we conclude that one of the major discernible consequences of the closed‐loop coupling of two GTPases is its ability to faithfully transmit information from the PM to the Golgi for the latter to mount a concordant secretory response. Second, although the first switch, that is, Arf1/mG* showed a linear response, the subsequent steps (switch #2 and the step of membrane mechanics leading to secretion) become progressively ultrasensitive. The net result of this is that the closed‐loop feedback control allows for a tighter alignment of secretion with respect to EGF by “stretching” out the dose–response curve across a series of switches to propagate the signal from the extracellular space to the interior of the cell. Because the stability behavior of a mathematically simpler version of this closed‐loop system of coupled GTPases showed that coupling afforded a wide range of steady states (Stolerman *et al*, 2021), it is tempting to speculate that the coupled system allows flexibility in responses over a wide range of stimulus. In fact, follow‐up work has now revealed how ranges of activity of the mGTPase Arf1, reaction kinetics, the negative feedback loop (mGAP), and the cascade length affect DoRA (Qiao *et al*, 2023).

When it comes to the field of protein secretion, the cell's secretory pathway was originally believed to be a constitutive function that is regulated by “housekeeping” genes/proteins that maintain the integrity of the local (membrane or lumenal) environment (Arvan *et al*, 2002). The earliest evidence that secretion is regulated by exogenous GFs emerged in 2008 when the phosphoinositide phosphatase SAC1 was implicated as a “brake” in anterograde Golgi secretion that is released by GFs (Blagoveshchenskaya *et al*, 2008). Despite these insights, what remained unknown was how the secretory system (or any intracellular organelle/system) responds proportionately to external cues. The functional consequences of an endomembrane coupled GTPase system we dissected here fill that knowledge gap. We show that coupling of m/tGTPases with CLC within cells is critical to set up feedback controls in yet another scale, that is, cell secretion and cell fate (i.e., survival vs. death).

Finally, when it comes to the field of cancer cell biology, it is well‐accepted that self‐sufficiency in growth signaling is a hallmark of all cancer cells (Hanahan & Weinberg, 2000); we show here how cells achieve such self‐sufficiency for the prototypical GF system, that is, EGF/EGFR. Existing theories linking genetic circuits to cellular autonomy, although quantifiable and tunable (Youk & Lim, 2014; Maire & Youk, 2015; Doğaner *et al*, 2016; Kamino *et al*, 2017; Tang *et al*, 2021), do not apply to multicellular eukaryotes. In dissecting the behavior of the coupled GTPase system, and revealing the consequences of its disruption, both *in silico* and in two different cancer cells, we fill that knowledge gap. Second, intratumoral cellular heterogeneity is known to give rise to an ecosystem of clonal interactions (Basanta & Anderson, 2013; Tabassum & Polyak, 2015) that can drive tumor growth, therapeutic resistance, and progression (Merlo *et al*, 2006; Basanta & Anderson, 2017; Maley *et al*, 2017; Li & Thirumalai, 2019). Therefore, it is possible that a few autonomous secretor clones with an intact secrete‐and‐sense loop could be sufficient to support the survival of neighboring non‐secretor clones. If so, uncoupling the GTPases and disrupting the secrete‐and‐sense autonomy could serve as an impactful therapeutic strategy. Finally, the evolutionary significance of our findings is noteworthy. For example, the linker between the GTPases, that is, GIV, evolved later in multicellular organisms such as worms (Nechipurenko *et al*, 2016) and flies (Puseenam *et al*, 2009; Yamaguchi *et al*, 2010; Ha *et al*, 2015; Houssin *et al*, 2015). GIV's HOOK module (binds mGTPase) evolved in worms and flies (Puseenam *et al*, 2009; Yamaguchi *et al*, 2010; Ha *et al*, 2015; Houssin *et al*, 2015); its GEM domain (a short motif that binds and modulates tGTPases) evolved later in fish (DiGiacomo *et al*, 2018) and remains to date. Thus, the coupled GTPase circuit likely evolved in higher eukaryotes, and as suggested by our multi‐organelle proteomic analyses, is geared to support autonomy in multicellular organisms. This is consistent with the fact that evolution appears to favor efficient signaling circuits that can accomplish many different tasks (Milo *et al*, 2002; Shen‐Orr *et al*, 2002). Because GIV is overexpressed in the most aggressive tumor cells, it is likely that the GTPase coupled circuit is more frequently assembled in those cells. If so, the circuit may represent an evolutionary masterpiece of multiscale feedback control to achieve autonomy, adaptability, and flexibility. Follow‐up work has now shed light on the importance of this phenomenon in the orchestration of self‐sustained EGFR/ErbB signaling in tumor cells (preprint: Sinha *et al*, 2022). Such autonomy in growth signaling appears to be critical for the maintenance of high metastatic potential and epithelial–mesenchymal plasticity during the blood‐borne dissemination of human breast cancer.

### Limitations of the study

The multi‐timescale model we built ignores the spatial aspects of the various feedback control loops. Because the spatial organization of signaling motifs will influence their temporal behaviors, we anticipate the need for further refinement of the current model. By depleting GIV, we disconnect the GTPases and dismantle the entire circuit; selective disruption of various connections within the Golgi‐localized circuit is not possible currently due to the lack the experimental tools (e.g., specific point mutants of GIV, GEF, or GAPs or perturbagens such as a small molecule or peptides). Although we studied four different cargo proteins (VSV‐G, MMP2/9, and Col‐VII) and two types of stimuli (EGF and serum), a more comprehensive assessment of the cell's secretome is expected to reveal how the intracellular GTPase circuit controls the composition of the extracellular space. We chose to use mathematical modeling to test the experimentally determined key components by design, but there may be missing components that enable other emergent properties (such as advantages of AND vs. OR gate mechanisms in the feedback loops); future work is expected to build upon this framework to fill these knowledge gaps. Conducting experiments across the full range of stimuli to assess “proportionality/linearity” of response was possible in some instances (e.g., cell survival) but not possible in others (e.g., FRET, Arf1 activity, etc.) due to technical limitations of the assays and/or detection thresholds. Finally, our mathematical model ignores the effect of the physical location and heterogeneity of cells. To explore such homogeneous and heterogeneous cell population (Gerlee & Anderson, 2008; Sottoriva *et al*, 2010; Poleszczuk *et al*, 2015) future studies will need to include agent‐based models (Wang *et al*, 2007; Chao Dennis *et al*, 2008; Norton & Popel, 2014), in which each cell is regarded as an individual agent that “senses” the environment and “decides/acts” in response.

## Materials and Methods

### Reagents and Tools table

**Table:** 

Reagent or Resource	Source	Identifier
**Antibodies**		
Mouse monoclonal anti‐Gαi‐GTP	Graeme Milligan (Lane *et al*, 2008)	26901
Rabbit anti‐Arf1	Paul Randazzo (Marshansky *et al*, 1997)	n/a
Rabbit anti‐Mannosidase (Man)‐II	Gift from K. Moreman (Velasco *et al*, 1993)	n/a
Anti‐GFP	Living Colors, Invitrogen (Thermo Scientific)	Catalog # MA5‐15256
Anti‐RFP	Invitrogen (Thermo Scientific)	Catalog # MA5‐15257
Anti‐GIV coiled coil antibody	Millipore (Sigma)	ABT80
Goat anti‐Rabbit IgG, Alexa Fluor 594 conjugated	Thermo Fisher Scientific	A11072
Goat anti‐Mouse IgG, Alexa Fluor 488 conjugated	Thermo Fisher Scientific	A11017
IRDye 800CW Goat anti‐Mouse IgG Secondary (1:10,000)	LI‐COR Biosciences	926‐32210
IRDye 680RD Goat anti‐Rabbit IgG Secondary (1:10,000)	LI‐COR Biosciences	926‐68071
**Biological samples**		
N/a		
**Chemicals, peptides, and recombinant proteins**		
DAPI (4′,6‐Diamidino‐2‐Phenylindole, Dilactate)	Thermo Fisher Scientific	D3571
MTT	Millipore Sigma	475989‐1GM
Puromycin	Sigma	P9620‐10ML
Brefeldin A	Sigma	B6542‐5MG
Fetal Bovine Serum	PEAK SERUM	PS‐FB1
Paraformaldehyde 16%	Electron Microscopy Biosciences	15710
Glutathione Sepharose^â^ 4B	Sigma–Aldrich	GE17‐0756‐04
Protease inhibitor cocktail	Roche	11 873 580 001
Tyr phosphatase inhibitor cocktail	Sigma–Aldrich	P5726
Ser/Thr phosphatase inhibitor cocktail	Sigma–Aldrich	P0044
PVDF Transfer Membrane, 0.45 mM	Thermo Scientific	88518
Prolong Glass	Thermo Fisher Scientific	P36980
Paraformaldehyde 16%	Electron Microscopy Biosciences	15710
Guava Cell Cycle Reagent	Millipore Sigma	4700‐0160
**Commercial kits**		
Dead Cell Apoptosis Kit with Annexin V Alexa Fluor™ 488 & Propidium Iodide (PI)	Thermo Fisher Scientific	V13241
**Experimental models: Cell lines**		
HeLa parental	ATCC	ATCC® CCL‐2
HeLa GIV KO (CRISPR Cas9)	Prior work (Abd El‐Hafeez *et al*, 2023)	n/a
MDA‐MB‐231	ATCC	ATCC® HTB‐26
MDA‐MB‐231 parental and GIV KO (CRISPR Cas9) lines	Prior work (Abd El‐Hafeez *et al*, 2023)	n/a
HeLa shControl	Prior work (Lo *et al*, 2015; Lopez‐Sanchez *et al*, 2015; Rohena *et al*, 2020)	n/a
HeLa shGIV	Prior work (Rohena *et al*, 2020)	n/a
Cos7 shControl	Prior work (Ma *et al*, 2015; Rohena *et al*, 2020)	n/a
Cos7 shGIV	Prior work (Ma *et al*, 2015; Rohena *et al*, 2020)	n/a
COS7	ATCC	ATCC® CRL‐1651™
HEK293T	ATCC	ATCC® CRL‐1573™
**Recombinant DNA**		
Internally tagged Gαi_1_‐YFP	Moritz Bünemann (Bunemann *et al*, 2003; Gibson & Gilman, 2006; Lo *et al*, 2015; Midde *et al*, 2015)	N/A
Girdin CRISPR/Cas9 KO Plasmid (h2)	Santa Cruz Biotechnology (SCBT) Inc.	Sc‐402236‐KO‐2
CFP‐Gβ_1_	Lo *et al* (2015)	N/A
Temperature sensitive (ts)VSVG‐eGFP	Lo *et al* (2015)	N/A
MMP2‐GFP	Marc Coppolino (Kean *et al*, 2009)	N/A
MMP9‐GFP	Marc Coppolino (Kean *et al*, 2009)	N/A
Col VII‐RFP	Anderzej Fertala (Chung *et al*, 2009)	N/A
GST GAT (GGA)	Stuart Kornfeld (Dell'Angelica *et al*, 2000)	N/A
**Other: Software**		
ImageJ	National Institute of Health	https://imagej.net/Welcome
IX81 FV1000 inverted confocal laser scanning microscope	Olympus	n/a
ClueGO	Cytoscape	Bindea *et al* (2009)
NetworkX	Python	https://networkx.org
Gephi	Gephi	https://gephi.org
Prism	GraphPad	https://www.graphpad.com/scientific‐software/prism/
LAS‐X	Leica	www.leica‐microsystems.com/products/microscope‐software/p/leica‐las‐x‐ls
Illustrator	Adobe	https://www.adobe.com/products/illustrator.html
MATLAB	MathWorks	https://www.mathworks.com/
ImageStudio Lite	LI‐COR	https://www.licor.com/bio/image‐studio‐lite/

### Methods and Protocols

#### Modeling approaches

##### Model assumptions

We restrict our modeling considerations to the secretory pathway on Golgi and its interactions with cell survival. The secretory pathway on Golgi consists of mGTPases, tGTPases, their GEFs and GAPs, and the secretion machinery. In the secretory pathway on Golgi, EGF mediates the recruitment of GEF for mGTPase (mGEF) and triggers the activation of corresponding mGTPases. Then active mGTPase can recruit GIV to vesicles. GIV is GEF for tGTPase (tGEF), and subsequently activates tGTPase. Upon activation of tGTPase, Gβγ is released and activates the GAP for mGTPase (mGAP). Besides, mGAP is also regulated by GIV, which binds to mGAP and works as a co‐factor for GAP activity. mGAP has a dual role in this circuit: one is to turn “OFF” mGTPase, and the other is to promote the vesicle formation. The vesicle formation is essential for secretion, and the secreted GFs leads to cell proliferation. The increase in cell number in turn enhances the secretion.

To model the above circuit, we assume that

- The total number of each type of GTPases is constant.
- The copy number of GAP for tGTPase (tGAP) is constant since it is not regulated by other species.
- The species are present in large enough quantities that deterministic approaches can be used to capture the dynamics of the system.
- The process of secretion can be modeled using a simplified function that depends on mGAP.

Therefore, the circuit is modeled by a set of ODEs with six species: active mGTPase, active tGTPase, mGEF, mGAP, tGEF, and the secreted GFs. Besides, the cell's survival number is also modeled by an ODE. We note that our model does not include the spatial or mechanical aspects associated with these signaling pathways.

##### Governing equations

Our model consists of two parts: one experimentally constrained module for coupled switches on the Golgi and the other module to predict the influence of coupled switches on the secrete‐and‐sense autonomy (Fig 1B). In the module for coupled switches, we modeled all the species interactions by normalized‐Hill functions (Saucerman & McCulloch, 2004; Cao *et al*, 2020) to capture the overall input–output relationships. We did not consider all the intermediary steps in the signaling pathway for the sake of simplicity. When active tGTPase and tGEF both regulate mGAP, the “AND” logic is applied and modeled as $f_{\mathrm{act}}\left(\text{tGTPases}\right)\cdot f_{\mathrm{act}}\left(\text{tGEF}\right)$. Thus, the dynamics of the system can be described by the following equations:

(1) $$\frac{d\left[\text{mGEF}\right]}{\mathrm{dt}}\tau_{\text{mGEF}}=\left(f_{\mathrm{act}}^{\left(1\right)}\left(\text{stimulus}\right)+k_{\text{mGEF}}\right)Y_{\text{mGEF}}^{\max}-\left[\text{mGEF}\right]$$

(2) $$\frac{d\left[\text{mGAP}\right]}{\mathrm{dt}}\tau_{\text{mGAP}}=\left(f_{\mathrm{act}}^{\left(2\right)}\left(\left[\text{tGEF}\right]\right)f_{\mathrm{act}}^{\left(3\right)}\left(\left[{\mathrm{tG}}^{*}\right]\right)+k_{\text{mGAP}}\right)Y_{\text{mGAP}}^{\max}-\left[\text{mGAP}\right]$$

(3) $$\frac{d\left[{\mathrm{mG}}^{*}\right]}{\mathrm{dt}}\tau_{{\mathrm{mG}}^{*}}=\left(f_{\mathrm{act}}^{\left(4\right)}\left(\left[\text{mGEF}\right]\right)+k_{{\mathrm{mG}}^{*}}\right)\left(1-\left[{\mathrm{mG}}^{*}\right]\right)-f_{\mathrm{act}}^{\left(5\right)}\left(\left[\text{mGAP}\right]\right)\left[{\mathrm{mG}}^{*}\right]$$

(4) $$\frac{d\left[\text{tGEF}\right]}{\mathrm{dt}}\tau_{\text{tGEF}}=\left(f_{\mathrm{act}}^{\left(6\right)}\left(\left[{\mathrm{mG}}^{*}\right]\right)+k_{\text{tGEF}}\right)Y_{\text{tGEF}}^{\max}-\left[\text{tGEF}\right]$$

(5) $$\frac{d\left[{\mathrm{tG}}^{*}\right]}{\mathrm{dt}}\tau_{{\mathrm{tG}}^{*}}=\left(f_{\mathrm{act}}^{\left(7\right)}\left(\left[\text{tGEF}\right]\;\right)+k_{{\mathrm{tG}}^{*}}\right)\left(1-\left[{\mathrm{tG}}^{*}\right]\right)-f_{\mathrm{act}}^{\left(8\right)}\left(\left[\text{tGAP}\right]\right)\left[{\mathrm{tG}}^{*}\right]$$

where variables $\left[\text{mGEF}\right]$, $\left[\text{mGAP}\right]$, $\left[{\mathrm{mG}}^{*}\right]$, $\left[\text{tGEF}\right]$, and $\left[{\mathrm{tG}}^{*}\right]$ denote the fractional activation of mGEF, mGAP, mGTPase, tGEF, and tGTPase, respectively. Here, the fractional activation is the copy number divided by the maximal copy number, which changes between 0 and 1. The variable stimulus denotes the input signal EGF; the τ's are time scale; k's are basal production rates, and $Y_{i}^{\max},i=\text{mGEF},\text{mGAP},\text{tGEF}$ are maximal fractional activations for species. The function $f_{\mathrm{act}}^{\left(i\right)}\;\left(i=1,2,\cdots ,9\right)$ is the normalized‐Hill function, which takes the following form:

(6) factX=BXnKn+Xn,if0≤X<11,ifX≥1

where $B=\frac{{\mathrm{EC}}_{50}^{n}-1}{2{\mathrm{EC}}_{50}^{n}-1}$ and $K={\left(B-1\right)}^{1/n}$. Here, ${\mathrm{EC}}_{50}$ and n are half‐maximal activation and Hill coefficient, respectively. With these choices of constants B and K, we have $f_{\mathrm{act}}\left(0\right)=0$, $f_{\mathrm{act}}\left({\mathrm{EC}}_{50}\right)=0.5$. It should be noted that constants B and K can be different in different functions $f_{\mathrm{act}}^{\left(i\right)}$. In most cases, we used k=0 and $Y^{\max}\leq 1$, so the maximal value of variables $\left(1+k\right)Y^{\max}$ is smaller than 1 to ensure the range of the fractional activation. But, when we used a non‐zero k, the variable may be larger than 1, and then we regard the variable as the relative activation, which is normalized by a number smaller than the maximal copy number. We refer to this model as the coupled system throughout our study.

To predict the effect of coupled switches on the secrete‐and‐sense autonomy, we also built a model for secretion (denoted by S) and cell number (denoted by X). Since the activation‐deactivation circle of mGTPase is necessary for the secretion, we assume the secretion rate is positively correlated to $f_{\mathrm{act}}^{\left(9\right)}\left(\left[\text{mGAP}\right]\right)$. In addition, the proliferation of cells is regulated by secreted GFs to ensure homeostasis (Hart *et al*, 2014; Adler *et al*, 2018). Then, the dynamics of S and X are governed by:

(7) $$\frac{\mathrm{dS}}{\mathrm{dt}}=\left(\beta_{S}\left(f_{\mathrm{act}}^{\left(9\right)}\left(\left[\text{mGAP}\right]\right)+k_{S}\right)-\alpha_{S}\frac{S}{S+K_{2}}\right)X-\mathrm{\gamma S}$$

(8) $$\frac{\mathrm{dX}}{\mathrm{dt}}=\left(\lambda \frac{S}{S+K_{1}}\left(1-\frac{X}{K}\right)-\mu \right)X$$

where $\beta_{S}$ is the maximal secretion rate; $k_{S}$ is the basal secretion rate; $\alpha_{S}$ is the maximal endocytosis rate; γ is the degradation rate of secreted GFs; $K_{2}$ is the binding affinity of secreted GFs. In equation (8), λ and μ are cell proliferation and death rates by the cells, respectively; $K_{1}$ is the value of S when the Hill function $\frac{S}{S+K_{1}}$ is 0.5; K is the carrying capacity, that is, once the cell number is K, the cell proliferation rate is zero, preventing the cell number from exceeding K.

#### Single switch model

For the circuit that only contains the single switch of mGTPases, its dynamics is described by equations ((1), (2), (3)), except that equation (2) is replaced by

(9) $$\frac{d\left[\text{mGAP}\right]}{\mathrm{dt}}\tau_{\text{mGAP}}=k_{\text{mGAP}}Y_{\text{mGAP}}-\left[\text{mGAP}\right]$$

Note that this equation also can be used to describe the dynamics of mGAP when the regulation from tGEF to mGAP or the regulation from active tGTPase to mGAP does not exist.

##### Numerical simulations for the deterministic model

Numerical simulations were implemented in MATLAB. We use the solver ode15s to simulate the dynamics on the time interval [0, 1,440] min unless otherwise specified.

##### Fitting against experimental data

To fit the time course data for control cells and GIV‐depleted cells, we manually tuned the parameters in our model until the normalized RMSE between simulated and measured fold changes of active Arf1 was less than 0.2 and that for active tGTPase less than 0.45. Moreover, parameters for secretion and cell survival are taken from their biologically plausible ranges (Adler *et al*, 2018). Our fitting goal was to capture the experimentally observed trends rather than obtain kinetic parameters since our model does not include all the reactions in the pathway(s). Here, the normalized RMSE is the RMSE over the mean value of all experimental data; the baseline for the simulation result is the initial fractional activation when simulating dynamics for control cells, and those for experimental Arf1 and tGTPase data are initial states in control cells. The obtained parameter values are listed in Table EV1. In all simulations, the initial condition is the starved state when stimulus=0, and then stimulus is set to be 0.23 to simulate the dynamics under the EGF‐stimulated condition. In all simulations, we use normalized values of EGF concentrations. The normalization was conducted such that the value of 0.23 EGF used in simulations corresponds to 50 nM in the experiments. The dimensionless EGF concentrations in the simulations are obtained by dividing the EGF concentration by 217.4 nM (=50 nM/0.23).

##### Testing model

We verify that our setting in the model for GIV‐deplete cells indeed makes the system behave like the uncoupled system. We set the maximal fractional activation of tGEF as 0.1 (i.e., $Y_{\text{tGEF}}=0.1$) but keep other parameters unchanged to model the system in GIV‐depleted cells. The initial state is determined by the steady‐state values of all species when the stimulus is zero, which are obtained as follows: we set *stimulus* zero and chose an arbitrary initial condition (e.g., all species are 0.5), and then simulated the deterministic dynamics on the time interval [0, 2,400 h] to ensure that the steady state is reached. → Then we changed the stimulus to 0.23 to simulate the dynamics of all species when EGF = 50 nM. We find that GIV‐depleted cells are more likely behave as the uncoupled system (Appendix Fig S3A). For these two systems, mGEF and mG* both increase upon the stimulus of EGF, and mGAP will not increase because of low activation of tGEF in GIV‐depleted cells or the absence of the positive regulation from tGEF and tG* in the uncoupled system. Due to the non‐increasing level of mGAP, these two systems both show non‐decreasing fractional activation of mG*, low secretion, and low cell number. The only difference between these two systems is the dynamics of tGTPase switch: tGEF is low in GIV‐depleted cells and thus cannot activate tG*, while in the uncoupled system the fractional activations of tGEF and tG* are both high. The schematics of these three systems are shown in Appendix Fig S3B–D.

##### Sensitivity analysis

To test the robustness of the model, we performed sensitivity analyses. The sensitivity measures how the system output is vulnerable to the parameter change and can be captured by the following quantity:

$$\text{Sensitivity}=\frac{\mathrm{dln}\left(X\right)}{\mathrm{dln}\left(\alpha \right)}$$

where the X is the system's output and α is the kinetic parameter. In our analyses, we calculated this sensitivity for each kinetic parameter, that is, the α can be every kinetic parameter. The output X is the normalized RMSE value for simulated mG* or tG* dynamics, or steady‐state values of the secretion or the cell number. This derivative is approximated by the ratio of the difference of $\ln\left(X\right)$ when α is 1.1×α and 0.9×α to the 0.2×α. We found that, perturbations of the half‐maximal activation ${\mathrm{EC}}_{50}$ will cause large changes in the normalized RMSEs and the steady‐state value of the secretion for coupled switches (Appendix Fig S4). Except ${\mathrm{EC}}_{50}$, the mG* and tG* dynamics seem robust to other kinetic parameters, since the sensitivities for other kinetic parameters are between −0.5 and 0.5. Besides, the steady state of the secretion in coupled switches is sensitive to the maximal secretion rate $\alpha_{S}$ and the maximal endocytosis rate $\beta_{S}$. These not very large sensitivities indicate that the main conclusions hold under small perturbations.

##### The stochastic model

To investigate the impact of noise, we consider three different sources of noise: stimulus, species, and connections. A noisy stimulus is modeled by the summation of the mean and a noise term $\eta_{\mathrm{sti}}\left(t\right)$; another type of noise, originated from species, is generated by adding a noise term $\eta_{\mathrm{spe}}\left(t\right)$ in the equation for each species, and tGAP is also perturbed by a noise term $\eta_{\mathrm{spe}}^{\text{tGAP}}\left(t\right)$; the third type of noise, which comes from connections, is modeled by adding a noise term $\eta_{\text{link}}\left(t\right)$ to each activation function $f_{\mathrm{act}}$ and nonlinear reaction rates in equations for the secretion and the cell number. Here, these noise terms are independent of each other, and all modeled by the following Ornstein–Uhlenbeck process:

(10) $$\tau_{j}^{\text{noise}}d\eta_{j}=-\eta_{j}\mathrm{dt}+\sigma_{j}dW_{t}^{j}$$

where j=sti,spe,link, and $W_{t}^{j}$'s are independently and identically distributed standard Wiener processes. This equation implies that $\eta_{j}\left(t\right)$ has zero mean and variance $\frac{\sigma_{j}^{2}}{2\tau_{j}^{\text{noise}}}$ . The equations for active tGEF, the secretory protein, and the cell number in the presence of noise are taken as an example: when noise exists only in species, the dynamics of active tGEF, the secretory protein, and the cell number are described by

(11) $$\frac{d\left[\text{tGEF}\right]}{\mathrm{dt}}\tau_{\text{mGEF}}=\left(f_{\mathrm{act}}^{\left(6\right)}\left(\left[{\mathrm{mG}}^{*}\right]\right)+k_{\text{tGEF}}\right)Y_{\text{tGEF}}-\left[\text{tGEF}\right]+\eta_{\mathrm{spe}}^{\text{tGEF}}$$

(12) $$\frac{\mathrm{dS}}{\mathrm{dt}}=\left(\beta_{S}\left(f_{\mathrm{act}}^{\left(9\right)}\left(\left[\text{mGAP}\right]\right)+k_{S}\right)-\alpha_{S}\frac{S}{S+K_{2}}\right)X-\mathrm{\gamma S}+\eta_{\mathrm{spe}}^{S}$$

(13) $$\frac{\mathrm{dX}}{\mathrm{dt}}=\left(\lambda \frac{S}{S+K_{1}}\left(1-\frac{X}{K}\right)-\mu \right)X+\eta_{\mathrm{spe}}^{X}$$

while the corresponding dynamics when noise are present in connections are governed by

(14) $$\frac{d\left[\text{tGEF}\right]}{\mathrm{dt}}\tau_{\text{mGEF}}=\left(f_{\mathrm{act}}^{\left(6\right)}\left(\left[{\mathrm{mG}}^{*}\right]\right)+\eta_{\text{link}}^{\text{tGEF}}+k_{\text{tGEF}}\right)Y_{\text{tGEF}}-\left[\text{tGEF}\right]$$

(15) $$\frac{\mathrm{dS}}{\mathrm{dt}}=\left(\beta_{S}\left(f_{\mathrm{act}}^{\left(9\right)}\left(\left[\text{mGAP}\right]\right)+k_{S}\right)+\eta_{\text{link}}^{S,1}-\alpha_{S}\frac{S}{S+K_{2}}+\eta_{\text{link}}^{S,2}\right)X-\mathrm{\gamma S}$$

(16) $$\frac{\mathrm{dX}}{\mathrm{dt}}=\left(\lambda \frac{S}{S+K_{1}}\left(1-\frac{X}{K}\right)+\eta_{\text{link}}^{X}-\mu \right)X$$

where $\eta_{\text{link}}$'s with different superscripts are independent noise terms.

##### Numerical simulations for the stochastic model

Numerical simulations were implemented in MATLAB. We used the Milstein scheme (Kloeden & Platen, 1992) to numerically solve the noise term $\eta_{j}$ (j=sti,spe,link), and used the Euler scheme to solve the dynamics of molecules on the time interval [0, 1,440 min]. To be specific, the noise term $\eta_{j}$ at n+1 time step is determined in the following manner ($\tau_{j}^{\text{noise}}=1$):

$$\eta_{j}^{n+1}=\eta_{j}^{n}-\eta_{j}^{n}\mathrm{dt}+\sigma_{j}{\mathrm{\delta W}}_{n}+\frac{1}{2}\sigma_{j}^{2}\left[{\left({\mathrm{\delta W}}_{n}\right)}^{2}-\mathrm{dt}\right]$$

where dt is the time step and ${\mathrm{\delta W}}_{n}$ obeys the normal distribution with mean zero and variance dt. Then, the activation of molecules or the cell number is solved by the Euler scheme. For example, when noise is only in stimulus, the mGEF at n+1 time step, denoted as ${\left[\text{mGEF}\right]}^{n+1}$, is obtained by the following equation:

$${\text{[mGEF]}}^{n+1}=\begin{array}{l} {\text{[mGEF]}}^{n}+\mathrm{dt}\frac{1}{\tau_{\text{mGEF}}}((f_{\mathrm{act}}^{\left(1\right)}\left(\text{stimulus}+\eta_{\mathrm{sti}}^{n+1}\right)\\ +\;k_{\text{mGEF}})Y_{\text{mGEF}}-{\text{[mGEF]}}^{n}), \end{array}$$

the schemes to solve equations (12) and (15) are

$$S^{n+1}=\begin{array}{l} S^{n}+\mathrm{dt}(\left(\beta_{S}\left(f_{\mathrm{act}}^{\left(9\right)}\left({\text{[mGAP]}}^{n}\right)+k_{S}\right)-\alpha_{S}\frac{S^{n}}{S^{n}+K_{2}}\right)X-\gamma S^{n}\\ +{\left(\eta_{\mathrm{spe}}^{S}\right)}^{n+1}), \end{array}$$

and

$$S^{n+1}=\begin{array}{l} S^{n}+\mathrm{dt}((\beta_{S}\left(f_{\mathrm{act}}^{\left(9\right)}\left({\text{[mGAP]}}^{n}\right)+k_{S}\right)+{\left(\eta_{\text{link}}^{S,1}\right)}^{n+1}\\ -\;\alpha_{S}\frac{S^{n}}{S^{n}+K_{2}}+{\left(\eta_{\text{link}}^{S,2}\right)}^{n+1})X-\gamma S^{n}), \end{array}$$

respectively.

We compare coupled switches with the single switch of mGTPase for three different cases of noise: noise in the stimulus, noise in the stimulus and species simultaneously, and noise in the stimulus and connections simultaneously. The values of noise amplitudes used for simulations are listed as follows:

- When noise is only in the stimulus, the parameter $\sigma_{\mathrm{sti}}$ for $\eta_{\mathrm{sti}}\left(t\right)$ is 0.02, and $\tau_{\mathrm{sti}}^{\text{noise}}$ is 1.
- When noise is in the stimulus and species simultaneously, parameters $\sigma_{\mathrm{sti}}$ and $\tau_{\mathrm{sti}}^{\text{noise}}$ for $\eta_{\mathrm{sti}}\left(t\right)$ are the same as those when noise is only in the stimulus. In addition, for the noise term $\eta_{\mathrm{spe}}\left(t\right)$, $\tau_{\mathrm{spe}}^{\text{noise}}=1$, and $\sigma_{\mathrm{spe}}$ is 0.02 for all species except the secretion and cell number. Since the secretion and cell number have small reaction rates, $\sigma_{\mathrm{spe}}^{S}$ and $\sigma_{\mathrm{spe}}^{X}$ are set to be $2\times 10^{-5}$ and $2\times 10^{-6}$ respectively, and thus the noisy behaviors cannot overwhelm the deterministic behaviors.
- When noise is in the stimulus and connections simultaneously, parameters $\sigma_{\mathrm{sti}}$ and $\tau_{\mathrm{sti}}^{\text{noise}}$ for $\eta_{\mathrm{sti}}\left(t\right)$ are still the same as those when noise is only in the stimulus. Moreover, $\tau_{\text{link}}^{\text{noise}}=1$, and $\sigma_{\text{link}}^{\text{noise}}=0.02$ for all species except the cell number. The $\sigma_{\text{link}}^{X}$ is 0.002 to ensure the same order of the noise and the production rate of cell number.

In this study, for a given input signal, we performed 1,000 repeated simulations on the time interval [0, 1,440 min] (with the steady state under this signal as the initial state). The time step dt is set to be 0.01.

#### Computational and bioinformatics approaches

##### Identification of a Golgi‐localized Arf1 and GIV interactome

We have previously extracted an annotated subcellular localization network of high‐confidence GIV correlators (Ear *et al*, 2021), based on Human Cell Map (HCM; Go *et al*, 2021). From the same HCM data set, a set of high‐confidence Arf1 correlators were also extracted. Using the combined set of proteins that were correlated with GIV and Arf1, a full correlation network between every protein was extracted. Annotated unique GIV interactors from BioGRID (Oughtred *et al*, 2021) were also incorporated to expand the GIV–Arf1 interaction network. To assign subcellular localization of the GIV interactors from BioGRID (Oughtred *et al*, 2021), they were first matched to subcellular localization as annotated by HCM. For those proteins that were not assigned by HCM, they were then matched to Gene Ontology (GO) Cellular Component terms, Uniprot (The UniProt, 2019), and Human Protein Atlas (Uhlén *et al*, 2015), which were all used as a guide to manually assign them based on their biological function. The complete list of this “Golgi‐localized Arf1‐GIV interactome” is provided as Dataset EV1.

##### Protein–protein interaction network construction, *in silico* perturbation, and topological analyses

The list of proteins (Dataset EV1) was used as “seed” to generate the Golgi‐specific Arf1‐GIV network by fetching other connecting interactions and proteins from STRING database (Franceschini *et al*, 2013). The shortest path NetworkX algorithm (Sinha *et al*, 2021) was used to trace the connected proteins and interactions between every possible pair of proteins from the above‐mentioned list. The highest possible interaction cutoff score was used to avoid false positive interactions. To understand the impact of GIV deletion, a similar network was prepared, except without GIV. The shortest path alteration fraction (Sinha *et al*, 2021) associated with Arf1 was calculated using differential shortest path analysis of the original and GIV‐depleted PPI network. Here only the paths having shortest path alteration fraction 1 were considered which indicated only the deleted or newly added shortest paths due to GIV deletion. GO Biological Process (BP) analysis of the proteins identified using shortest path alteration fraction analysis was performed using the Cytoscape tool ClueGO (Bindea *et al*, 2009) and significant GO BP terms were visualized.

##### TMT proteomics analysis, network construction, and multi‐layer visualization

Proteins that are upregulated in WT were mapped using the STRING database (https://string-db.org/). A pathway enrichment analysis of the most highly connected nodes was performed using the Reactome database (https://reactome.org/). The compartmentalized distribution of proteins within the PPI network based on their organelle‐specific location was mapped using the Cell Atlas Uniform Manifold Approximation and Projection (UMAP) explorer that was generated using the large collection of confocal microscopy images showing the subcellular localization patterns of human proteins, curated and made available at Human protein atlas (https://www.proteinatlas.org/). Multilayer visualization of an organelle‐based interaction network was constructed using MultiViz plugin (preprint: Jayamohan Pillai *et al*, 2022) of Gephi platform. All the source codes for network analysis are available at https://github.com/sinha7290/PPIN. MultiViz plugin source code is available at https://github.com/JSiv/gephi-plugins.

#### Experimental model and subject details

##### Cell lines and culture methods

HeLa, Cos7, and MDA‐MB‐231 cells were grown at 37°C in their suitable media, according to their supplier instructions, supplemented with 10% FBS, 100 U/ml penicillin, 100 μg/ml streptomycin, 1% l‐glutamine, and 5% CO_2_.

##### GIV CRISPR/Cas9 gene editing and validation

Pooled guide RNA plasmids (commercially obtained from Santa Cruz Biotechnology; Cat# sc‐402236‐KO‐2) were used to generate both HeLa and MDA MB‐231 GIV KO lines as described before (Ear *et al*, 2021). Briefly, these CRISPR/Cas9 KO plasmids consist of GFP and Girdin‐specific 20 nt guide RNA sequences derived from the GeCKO (v2) library and target human Girdin exons 6 and 7. Plasmids were transfected into Hela and MDA‐MB‐231 cells using PEI. Cells were sorted into individual wells using a cell sorter based on GFP expression. To identify cell clones harboring mutations in the gene coding sequence, genomic DNA was extracted using 50 mM NaOH and boiling at 95°C for 60 min. After extraction, pH was neutralized by the addition of 10% volume 1.0 M Tris‐pH 8.0. The crude genomic extract was then used in PCR reactions with primers flanking the targeted site. Amplicons were analyzed for insertions/deletions (indels) using a TBE‐PAGE gel. Indel sequence was determined by cloning amplicons into a TOPO‐TA cloning vector (Invitrogen) following manufacturer's protocol.

##### Reagents and antibodies

All sources for key reagents are listed in the *Resource Table* above. Unless otherwise mentioned, all chemicals were purchased from Sigma (St Louis, MO). A mouse mAb against the active conformation of Gαi was obtained from Dr. Graeme Milligan (University of Glasgow, UK). Rabbit anti‐Arf1 IgG was prepared as described (Marshansky *et al*, 1997). Rabbit polyclonal anti‐α‐mannosidase II (Man II) serum was prepared as described (Velasco *et al*, 1993). Highly cross‐absorbed Alexa Fluor 594 or 488 F(ab)'_2_ fragments of goat anti‐mouse or anti‐rabbit IgG (H + L) for immunofluorescence were purchased from Invitrogen (Carlsbad, CA). Goat anti‐rabbit and anti‐mouse Alexa Fluor 680 or IRDye 800 F(ab)'_2_ for immunoblotting, were obtained from LI‐COR Biosciences.

##### Cell culture, transfection, ligand stimulation, and lysis

HeLa and MDA MB‐231 (American Type Culture Collection, Manassas, VA) were maintained in DMEM (Invitrogen) supplemented with 10% FBS (Hyclone, Logan, UT), 100 U/ml penicillin, 100 μg/ml streptomycin, 1% l‐glutamine, and 5% CO_2_. Control and GIV shRNA HeLa and Cos7 stable cell lines were selected with 2 μg/ml of Puromycin (GIBCO) using a plasmid expressing an shRNA targeting its 3′ UTR (Ghosh *et al*, 2016a). Depletion of GIV was verified using a GIV‐CT antibody with an efficiency of ~95% and cells were extensively validated in prior studies (Lo *et al*, 2015; Ma *et al*, 2015; Rohena *et al*, 2020). Transfection of cells with fluorescent plasmids (FRET studies) was carried out using transit‐LT1 (Mirus Bio, Madison, WI) following the manufacturer's protocol. Cells were checked for mycoplasma contamination and authenticated by STR profiling periodically.

For ligand stimulation of cells, serum starvation was carried out overnight (~16–18 h) by replacing media with 0.2% FBS‐containing media in the case of HeLa prior to exposing them to the ligands.

Lysates used as a source of proteins in pulldown assays were prepared by resuspending cells in Tx‐100 lysis buffer [20 mM HEPES, pH 7.2, 5 mM Mg‐acetate, 125 mM K‐acetate, 0.4% Triton X‐100, 1 mM DTT, supplemented with sodium orthovanadate (500 mM), phosphatase (Sigma) and protease (Roche) inhibitor cocktails], after which they were passed through a 28G needle at 4°C, and cleared (10,000 × *g* for 10 min) before use in subsequent experiments.

##### Arf1 activation assays

Purification of GST‐GAT protein and assessment of Arf1 activation was described previously. In brief, cells were lysed with 1% Triton X‐100, 50 mM Tris, pH 7.5, 100 mM NaCl, 2 mM MgCl_2_, 0.1% SDS, 0.5% sodium deoxycholate, and 10% glycerol with protease inhibitors. Equal amounts of lysates were incubated with GST‐GGA3 (~40 μg) prebound glutathione‐Sepharose 4B beads at 4°C for 1 h. Beads were washed, and the bound proteins were eluted by boiling in Laemmli sample buffer for 5 min, resolved on a 15% SDS–PAGE, and analyzed by immunoblotting.

##### Quantitative immunoblotting

For immunoblotting, protein samples were boiled in Laemmli sample buffer, separated by SDS–PAGE and transferred onto 0.45 mM PVDF membrane (Millipore) prior to blotting. The duration of transfer was 30 min, at 100 V. Post transfer, membranes were blocked using 5% non‐fat milk or 5% BSA dissolved in PBS. Primary antibodies were prepared in a blocking buffer containing 0.1% Tween‐20 and incubated with blots, rocking overnight at 4*°*C. After incubation, blots were incubated with secondary antibodies for 1 h at room temperature, washed, and imaged using a dual‐color Li‐Cor Odyssey imaging system.

##### Immunofluorescence and confocal microscopy

For immunofluorescence, cells grown on coverslips were fixed in 3% paraformaldehyde (PFA) and processed as described previously (Ghosh *et al*, 2010). Antibody dilutions were as follows: Man II, 1:800; anti‐Gαi·GTP, 1:25; goat anti‐mouse or anti‐rabbit Alexa 488 or Alexa 594, 1:500. DAPI was used at 1:10,000. To estimate the degree of colocalization (Mander's overlap coefficient; MOC) in immunofluorescence assays, an ImageJ plugin, JACoP (https://imagej.nih.gov/ij/plugins/track/jacop2.html) was used. This was preferred over Pearson's because it is a good indicator of the proportion of the green signal (active G protein) coincident with a signal in the red channel (Man II, indicative of Golgi membranes) over its total intensity, which may even apply if the intensities in both channels are really different from one another. Coverslips were mounted using Prolong Gold (Invitrogen) and imaged using a Leica SPE CTR4000 confocal microscope.

##### Image processing

All images were processed on ImageJ software (NIH) and assembled into figure panels using Photoshop and Illustrator (Adobe Creative Cloud). All graphs were generated using GraphPad Prism.

##### FRET studies

Intramolecular FRET was detected by sensitized emission using the three‐cube method performed as previously reported by Midde *et al* (2015). Briefly, previously validated internally tagged Gαi_1_‐YFP and CFP‐Gβ_1_ FRET probe pairs were used (Bunemann *et al*, 2003; Gibson & Gilman, 2006). Cells were transfected with the probes, serum‐starved overnight, and then stimulated with EGF (50 nM) exactly as done previously (Midde *et al*, 2015; Kalogriopoulos *et al*, 2020). All fluorescence microscopy assays were performed on single cells in a mesoscopic regime to avoid inhomogeneities from samples as shown previously by Midde *et al* (Borejdo *et al*, 2012; Midde *et al*, 2015). Briefly, cells were sparsely split into sterile 35 mm MatTek glass bottom dishes and transfected with 1 μg of indicated constructs. To optimize the signal‐to‐noise ratio in FRET imaging, various expression levels of the transfected FRET probes were tested. However, to minimize complexities arising from molecular crowding, FRET probes were overexpressed by ∼1.5‐ to 2‐fold compared with the endogenous proteins. Because the stoichiometry of FRET probes has a significant impact on FRET efficiency, cells that expressed equimolar amounts of donor and acceptor probes (as determined by computing the intensity of the fluorescence signal by a photon‐counting histogram) were chosen selectively for FRET analyses. An Olympus IX81 FV1000 inverted confocal laser scanning microscope was used for live cell FRET imaging (UCSD‐Neuroscience core facility). The microscope is stabilized on a vibration‐proof platform, caged in temperature controlled (37°C) and CO_2_ (5%) supplemented chamber. A PlanApo 60× 1.40 N.A. oil immersed objective designed to minimize chromatic aberration and enhance resolution for 405–605 nm imaging was used. Olympus Fluoview inbuilt software was used for data acquisition. A 515 nm Argon‐ion laser was used to excite EYFP and a 405 nm laser diode was used to excite ECFP as detailed by Claire Brown's group (Broussard *et al*, 2013). Spectral bleed‐through coefficients were determined through FRET‐imaging of donor‐only and acceptor‐only samples (i.e., cells expressing a single donor or acceptor FP). Enhanced CFP emission was collected from 425–500 nm and EYFP emission was collected through 535–600 nm and passed through a 50 nm confocal pinhole before being sent to a photomultiplier tube to reject out‐of‐plane focused light. Every field of view (FOV) is imaged sequentially through ECFPex/ECFPem, ECFPex/EYFPem, and EYFPex/EYFPem (3 excitation and emission combinations) and saved as a donor, FRET, and acceptor image files through an inbuilt wizard. To obtain the FRET images and efficiency of energy transfer values a RiFRET plugin in Image J software was used (Roszik *et al*, 2009). Prior to FRET calculations, all images were first corrected for uneven illumination, registered, and background subtracted. For FRET quantification, regions of interest (ROI) were drawn in the juxtanuclear area presumably in the Golgi region (or at the cell periphery, presumed to be the plasma membrane regions) to compute energy transfer. Individual cells with fluorescence intensity in the mesoscopic regime detected in the donor and acceptor channels were selected for FRET analysis to avoid inhomogeneities between samples (Midde *et al*, 2013, 2014).

Manual and automatic registration of each individual channel in ImageJ was critical to correct motion artifacts associated with live cell imaging. Controls were performed in which images were obtained in different orders. The order in which images were obtained had no effect. FRET images were obtained by pixel‐by‐pixel ratiometric intensity method and the efficiency of transfer was calculated by the ratio of intensity in the transfer channel to the quenched (corrected) intensity in the donor channel. The following corrections were applied to all FOVs imaged: for crosstalk correction, cells transfected with CFP or YFP alone were imaged under all three previously mentioned excitation and emission combinations. FRET efficiency was quantified from 3–4 Regions of Interests (ROI) per cell drawn exclusively along the P.M. Because expression of FRET probes may have a significant impact on FRET efficiency, cells that expressed similar amounts of probes, as determined by computing the fluorescence signal/intensity by a photon counting histogram were selectively chosen for FRET analyses. Furthermore, untransfected cells and a field of view without cells were imaged to correct for background, autofluorescence, and light scattering. To avoid artifacts of photobleaching, Oxyfluor (www.oxyrase.com) was used to minimize the formation of reactive oxygen species.

##### GFP‐tsO45‐VSVG transport assays

To monitor anterograde (ER to Golgi) trafficking control or GIV‐depleted COS7 cells were transiently transfected with GFP‐tsO45‐VSV‐G plasmid (Presley *et al*, 1997). Transfected cells were incubated for 14–16 h at the restrictive temperature (40°C) to accumulate VSV‐G protein in the ER, shifted to 32°C for 0–60 min to release VSV‐G protein in the conditions described (i.e., 10% serum, EGF, or starved condition) and then fixed and processed under non‐permeabilized conditions (without detergent) for immunofluorescence. The rate of VSV‐G trafficking from the secretory compartments to the PM was determined by calculating the ratio of VSV‐G that was already at the PM (as determined using an anti‐VSV‐G ectodomain antibody; red pixels) normalized to the total cellular pool of VSV‐G (GFP; green pixels, using NIH ImageJ software).

##### Metalloprotease and collagen secretion assays

HeLa cells grown in a 6‐well plate were transfected with 2 μg of GFP‐MMP2, GFP‐MMP9, or Collagen‐RFP for 5 h. After 5 h, cells were fed with fresh media without FBS. Media was subsequently changed the next day (without FBS; exactly 1.5 ml/well) and stimulated with EGF. Media (100 μl) was collected just before the addition of EGF, as T = 0 h, and at the indicated time points after EGF stimulation. Each aliquot was subjected to high‐speed (14,000 × *g*) spin for 10 min prior to the addition of 50 μl of Laemmli sample buffer and boiling at 100°C.

##### MTT assay

Cell proliferation was measured using the MTT reagent and cells cultured in 96‐well plates. Parental or GIV‐KO HeLaor MDA‐MB‐231 cells were cultured in different concentrations of FBS (0, 0.25, 2, 5, and 10%). Then the cell lines were incubated with MTT for 4 h at 37°C. After incubation, culture media was removed and 150 μl of DMSO was added to solubilize the MTT formazan crystals. Optical density was determined at 590 nm using a TECAN plate reader. At least three independent experiments were performed. In an independent experiment, we tested the effect of using a Brefeldin A (BFA), a well‐known tool to inhibit secretion, on cell proliferation. The cell lines were cultured in different concentrations of FBS (0, 0.25, 2, 5, or 10%) and then treated with different concentrations of BFA (0, 0.01, 0.05, 0.1, 0.5, 1, 10, or 100 μM) and the MTT assays were done as described.

##### Cell cycle and apoptosis analyses

Cell cycle analysis and apoptotic cell quantification were performed using the Guava cell cycle reagent (Millipore Sigma) or the annexin V/propidium iodide (PI) staining kit (Thermo Fisher Scientific), respectively, according to the manufacturer's instructions. Cells were quantified on a BD™ (BD Biosciences) LSR II flow cytometer and analyzed using FlowJo software (FlowJo, Ashland, OR, USA).

##### Tandem Mass Tag™ (TMT) proteomics

WT and GIV‐KO MDA‐MB231 cells were maintained in 0 and 10% serum concentration in p10 dishes (Corning) for 16 h prior to harvest, and cell pellets were subsequently processed for TMT proteomics using LUMOS Orbitrap‐Fusion analyzer. Samples were processed at the UC San Diego Biomolecular and Proteomics Mass Spectrometry Core Facility (https://bpmsf.ucsd.edu/). Peptides are identified and mapped using Peaks X Pro pipeline. The intensity ratio of each identified protein in WT MDA‐MB231 vs. GIV‐KO MDA‐MB231 cells has been identified and selected if the significance score > 20. A list of differentially expressed proteins is provided in Dataset EV2. The mass spectrometry proteomics data have been deposited to the ProteomeXchange Consortium via the PRIDE partner repository (Perez‐Riverol *et al*, 2022) with the dataset identifier PXD037253.

#### Quantification and statistical analysis

##### Statistical analyses in modeling approaches

In the deterministic model, we fitted the dose–response curve by finding the best‐fit function with the form $a\frac{x^{n}}{x^{n}+K}+d$. We solved this optimal problem using “lsqcurvefit” in Matlab, and d can be deleted depending on the effect of the fitting. The only exception is for the mG vs. mGEF, where we used linear function ax+d. The difference between the fitted curve and the original curve is measured by R^2^, and it is defined as $1-\frac{\sum_{i}{\left(y_{i}-f_{i}\right)}^{n}}{\sum_{i}{\left(y_{i}-\bar{y}\right)}^{n}}$, where $y_{i}$ is the point in the original curve and $f_{i}$ is the prediction for $y_{i}$ based on the best‐fit curve. In the stochastic model, the standard deviation is calculated based on the data at 1,440 min, which is defined as the

$$\sqrt{\frac{\sum_{i=1}^{N}{\left(x_{i}-\bar{x}\right)}^{2}}{N-1}},$$

where N=1,000 and $\bar{x}=\frac{\sum_{i=1}^{N}x_{i}}{N}$. The $x_{i}$ is the molecular activation or the cell number at 1,440 min in the i‐th simulation.

##### Statistical analyses in protein–protein network analyses

An interaction cutoff score has been optimized while fetching the new proteins and their interactions from the STRING database, such that all the possible proteins will be included keeping the cutoff very high. In this instance, an interaction cutoff score of 667 has been used to include all the proteins from the seed list (Dataset EV1).

##### Statistical analyses in experimental studies and replication

All experiments were repeated at least three times (biological replicates, conducted on different days), and results were presented either as one representative experiment or as average ± SEM. Statistical significance was assessed with two‐sided unpaired Student *t*‐test and Mann–Whitney *t*‐test. For all tests, a *P*‐value of 0.05 was used as the cutoff to determine significance. The actual *P*‐values are indicated in each figure. All statistical analysis was performed using GraphPad Prism 8 or Matlab. Experiments undertaken did not require blinding; nor did they require sample size calculation or randomization.

#### Materials availability

This study did not generate new unique reagent.

## Author contributions

**Lingxia Qiao:** Methodology; investigation; formal analysis; visualization; writing – original draft; writing – review and editing. **Saptarshi Sinha:** Software; methodology; investigation; formal analysis; validation; visualization; writing – original draft; writing – review and editing. **Amer Ali Abd El‐Hafeez:** Validation; investigation; formal analysis. **I‐Chung Lo:** Investigation; formal analysis. **Krishna K Midde:** Investigation; formal analysis; validation; methodology. **Tony Ngo:** Validation; methodology. **Nicolas Aznar:** Investigation; formal analysis. **Inmaculada Lopez‐Sanchez:** Investigation; formal analysis. **Vijay Gupta:** Investigation; formal analysis. **Marilyn G Farquhar:** Resources; methodology. **Padmini Rangamani:** Conceptualization; methodology; data curation; funding acquisition; project administration; supervision; writing – original draft; writing – review and editing. **Pradipta Ghosh:** Conceptualization; methodology; data curation; visualization; funding acquisition; project administration; supervision; writing – original draft; writing – review and editing.

## Disclosure and competing interests statement

The authors declare that they have no conflict of interest.

## Supporting information



Appendix

Expanded View Figures PDF

Table EV1

Movie EV1

Dataset EV1

Dataset EV2

PDF+

Source Data for Figure 2

Source Data for Figure 3

Source Data for Figure 6

## Data Availability

Modeling computer scripts: GitHub (https://github.com/RangamaniLabUCSD/Coupled‐switches‐secretion). Protein–protein network analyses: Github (https://github.com/RangamaniLabUCSD/Coupled‐switches‐secretion). TMT proteomics datasets: PXD037253 (http://www.proteomexchange.org).

## References

[msb202211127-bib-0001] Abd El‐Hafeez AA , Sun N , Chakraborty A , Ear J , Roy S , Chamarthi P , Rajapakse N , Das S , Luker KE , Hazra TK *et al* (2023) Regulation of DNA damage response by trimeric G‐proteins. iScience 26: 105973 36756378 10.1016/j.isci.2023.105973 PMC9900518

[msb202211127-bib-0002] Adler M , Mayo A , Zhou X , Franklin RA , Jacox JB , Medzhitov R , Alon U (2018) Endocytosis as a stabilizing mechanism for tissue homeostasis. Proc Natl Acad Sci USA 115: E1926–E1935 29429964 10.1073/pnas.1714377115 PMC5828590

[msb202211127-bib-0003] Andrews SS , Peria WJ , Richard CY , Colman‐Lerner A , Brent R (2016) Push‐pull and feedback mechanisms can align signaling system outputs with inputs. Cell Syst 3: e442 10.1016/j.cels.2016.10.002 PMC5134923 27894998

[msb202211127-bib-0004] Andrews SS , Brent R , Balázsi G (2018) Signaling systems: transferring information without distortion. Elife 7: e41894 30358530 10.7554/eLife.41894 PMC6202050

[msb202211127-bib-0005] Arvan P , Zhao X , Ramos‐Castaneda J , Chang A (2002) Secretory pathway quality control operating in Golgi, plasmalemmal, and endosomal systems. Traffic 3: 771–780 12383343 10.1034/j.1600-0854.2002.31102.x

[msb202211127-bib-0006] Åström KJ , Murray RM (2021) Feedback systems: an introduction for scientists and engineers. Princeton, NJ: Princeton University Press

[msb202211127-bib-0007] Ayala I , Colanzi A (2016) Assays to study the fragmentation of the Golgi complex during the G2–M transition of the cell cycle. In The Golgi Complex: Methods and Protocols, Brown WJ (ed), pp 173–185. New York, NY: Springer New York 10.1007/978-1-4939-6463-5_14 27632010

[msb202211127-bib-0008] Aznar N , Kalogriopoulos N , Midde KK , Ghosh P (2016) Heterotrimeric G protein signaling via GIV/Girdin: breaking the rules of engagement, space, and time. Bioessays 38: 379–393 26879989 10.1002/bies.201500133 PMC5123561

[msb202211127-bib-0009] Barbazan J , Dunkel Y , Li H , Nitsche U , Janssen K‐P , Messer K , Ghosh P (2016) Prognostic impact of modulators of G proteins in circulating tumor cells from patients with metastatic colorectal cancer. Sci Rep 6: 22112 26916336 10.1038/srep22112 PMC4768264

[msb202211127-bib-0010] Barr FA , Leyte A , Huttner WB (1992) Trimeric G proteins and vesicle formation. Trends Cell Biol 2: 91–94 14732001 10.1016/0962-8924(92)90001-4

[msb202211127-bib-0011] Basanta D , Anderson ARA (2013) Exploiting ecological principles to better understand cancer progression and treatment. Interface Focus 3: 20130020 24511383 10.1098/rsfs.2013.0020 PMC3915838

[msb202211127-bib-0012] Basanta D , Anderson ARA (2017) Homeostasis back and forth: an ecoevolutionary perspective of cancer. Cold Spring Harb Perspect Med 7: a028332 28289244 10.1101/cshperspect.a028332 PMC5580509

[msb202211127-bib-0013] Beck R , Sun Z , Adolf F , Rutz C , Bassler J , Wild K , Sinning I , Hurt E , Brügger B , Béthune J (2008) Membrane curvature induced by Arf1‐GTP is essential for vesicle formation. Proc Natl Acad Sci USA 105: 11731–11736 18689681 10.1073/pnas.0805182105 PMC2575275

[msb202211127-bib-0014] Becskei A , Serrano L (2000) Engineering stability in gene networks by autoregulation. Nature 405: 590–593 10850721 10.1038/35014651

[msb202211127-bib-0015] Bhandari D , Lopez‐Sanchez I , To A , Lo I‐C , Aznar N , Leyme A , Gupta V , Niesman I , Maddox AL , Garcia‐Marcos M *et al* (2015) Cyclin‐dependent kinase 5 activates guanine nucleotide exchange factor GIV/Girdin to orchestrate migration–proliferation dichotomy. Proc Natl Acad Sci USA 112: E4874–E4883 26286990 10.1073/pnas.1514157112 PMC4568279

[msb202211127-bib-0016] Bindea G , Mlecnik B , Hackl H , Charoentong P , Tosolini M , Kirilovsky A , Fridman W‐H , Pagès F , Trajanoski Z , Galon J (2009) ClueGO: a Cytoscape plug‐in to decipher functionally grouped gene ontology and pathway annotation networks. Bioinformatics 25: 1091–1093 19237447 10.1093/bioinformatics/btp101 PMC2666812

[msb202211127-bib-0017] Blagoveshchenskaya A , Cheong FY , Rohde HM , Glover G , Knödler A , Nicolson T , Boehmelt G , Mayinger P (2008) Integration of Golgi trafficking and growth factor signaling by the lipid phosphatase SAC1. J Cell Biol 180: 803–812 18299350 10.1083/jcb.200708109 PMC2265582

[msb202211127-bib-0018] Borejdo J , Rich R , Midde K (2012) Mesoscopic analysis of motion and conformation of cross‐bridges. Biophys Rev 4: 299–311 28510208 10.1007/s12551-012-0074-y PMC5425693

[msb202211127-bib-0019] Boulay P‐L , Cotton M , Melançon P , Claing A (2008) ADP‐ribosylation factor 1 controls the activation of the phosphatidylinositol 3‐kinase pathway to regulate epidermal growth factor‐dependent growth and migration of breast cancer cells. J Biol Chem 283: 36425–36434 18990689 10.1074/jbc.M803603200 PMC2662303

[msb202211127-bib-0020] Brent R (2009) Cell signaling: what is the signal and what information does it carry? FEBS Lett 583: 4019–4024 19917282 10.1016/j.febslet.2009.11.029

[msb202211127-bib-0021] Broussard JA , Rappaz B , Webb DJ , Brown CM (2013) Fluorescence resonance energy transfer microscopy as demonstrated by measuring the activation of the serine/threonine kinase Akt. Nat Protoc 8: 265–281 23306460 10.1038/nprot.2012.147 PMC3756929

[msb202211127-bib-0022] Bunemann M , Frank M , Lohse MJ (2003) Gi protein activation in intact cells involves subunit rearrangement rather than dissociation. Proc Natl Acad Sci USA 100: 16077–16082 14673086 10.1073/pnas.2536719100 PMC307695

[msb202211127-bib-0023] Cancino J , Luini A (2013) Signaling circuits on the Golgi complex. Traffic 14: 121–134 23078632 10.1111/tra.12022

[msb202211127-bib-0024] Cao S , Aboelkassem Y , Wang A , Valdez‐Jasso D , Saucerman JJ , Omens JH , McCulloch AD (2020) Quantification of model and data uncertainty in a network analysis of cardiac myocyte mechanosignalling. Philos Trans A Math Phys Eng Sci 378: 20190336 32448062 10.1098/rsta.2019.0336 PMC7287329

[msb202211127-bib-0025] Chao Dennis L , Eck JT , Brash Douglas E , Maley Carlo C , Luebeck EG (2008) Preneoplastic lesion growth driven by the death of adjacent normal stem cells. Proc Natl Acad Sci USA 105: 15034–15039 18815380 10.1073/pnas.0802211105 PMC2567488

[msb202211127-bib-0026] Chigira M , Noda K , Watanabe H (1990) Autonomy in tumor cell proliferation. Med Hypotheses 32: 249–254 2172752 10.1016/0306-9877(90)90101-j

[msb202211127-bib-0027] Chung HJ , Steplewski A , Uitto J , Fertala A (2009) Fluorescent protein markers to tag collagenous proteins: the paradigm of procollagen VII. Biochem Biophys Res Commun 390: 662–666 19822129 10.1016/j.bbrc.2009.10.024 PMC2796180

[msb202211127-bib-0028] Cohen LA , Donaldson JG (2010) Analysis of Arf GTP‐binding protein function in cells. Curr Protoc Cell Biol 48: 14.12.11–14.12.17 10.1002/0471143030.cb1412s48 PMC2969170 20853342

[msb202211127-bib-0029] Dell'Angelica EC , Puertollano R , Mullins C , Aguilar RC , Vargas JD , Hartnell LM , Bonifacino JS (2000) GGAsa family of ADP ribosylation factor‐binding proteins related to adaptors and associated with the Golgi complex. J Cell Biol 149: 81–94 10747089 10.1083/jcb.149.1.81 PMC2175099

[msb202211127-bib-0030] DiGiacomo V , Marivin A , Garcia‐Marcos M (2018) When heterotrimeric G proteins are not activated by G protein‐coupled receptors: structural insights and evolutionary conservation. Biochemistry 57: 255–257 29035513 10.1021/acs.biochem.7b00845 PMC6082369

[msb202211127-bib-0031] Doğaner BA , Yan LKQ , Youk H (2016) Autocrine signaling and quorum sensing: extreme ends of a common Spectrum. Trends Cell Biol 26: 262–271 26671200 10.1016/j.tcb.2015.11.002

[msb202211127-bib-0032] Donaldson JG , Jackson CL (2011) ARF family G proteins and their regulators: roles in membrane transport, development and disease. Nat Rev Mol Cell Biol 12: 362–375 21587297 10.1038/nrm3117 PMC3245550

[msb202211127-bib-0033] Dunkel Y , Ong A , Notani D , Mittal Y , Lam M , Mi X , Ghosh P (2012) STAT3 protein up‐regulates Gα‐interacting vesicle‐associated protein (GIV)/Girdin expression, and GIV enhances STAT3 activation in a positive feedback loop during wound healing and tumor invasion/metastasis. J Biol Chem 287: 41667–41683 23066027 10.1074/jbc.M112.390781 PMC3516717

[msb202211127-bib-0034] Dunkel Y , Reid AL , Ear J , Aznar N , Millward M , Gray E , Pearce R , Ziman M , Ghosh P (2018) Prognostic relevance of CCDC88C (Daple) transcripts in the peripheral blood of patients with cutaneous melanoma. Sci Rep 8: 18036 30575751 10.1038/s41598-018-36173-x PMC6303298

[msb202211127-bib-0035] Ear J , Abd El‐Hafeez AA , Roy S , Ngo T , Rajapakse N , Choi J , Khandelwal S , Ghassemian M , McCaffrey L , Kufareva I (2021) A long isoform of GIV/Girdin contains a PDZ‐binding module that regulates localization and G‐protein binding. J Biol Chem 296: 100493 33675748 10.1016/j.jbc.2021.100493 PMC8042451

[msb202211127-bib-0036] Fedi P , Tronick SR , Aaronson SA (1997) Cancer Medicine. Baltimore, MD: Williams and Wilkins

[msb202211127-bib-0037] Franceschini A , Szklarczyk D , Frankild S , Kuhn M , Simonovic M , Roth A , Lin J , Minguez P , Bork P , von Mering C *et al* (2013) STRING v9.1: protein‐protein interaction networks, with increased coverage and integration. Nucleic Acids Res 41: D808–D815 23203871 10.1093/nar/gks1094 PMC3531103

[msb202211127-bib-0038] Gallione CJ , Rose JK (1985) A single amino acid substitution in a hydrophobic domain causes temperature‐sensitive cell‐surface transport of a mutant viral glycoprotein. J Virol 54: 374–382 2985803 10.1128/jvi.54.2.374-382.1985 PMC254807

[msb202211127-bib-0039] Garcia‐Marcos M , Ghosh P , Farquhar MG (2015) GIV/Girdin transmits signals from multiple receptors by triggering trimeric G protein activation. J Biol Chem 290: 6697–6704 25605737 10.1074/jbc.R114.613414 PMC4358093

[msb202211127-bib-0040] Gerlee P , Anderson ARA (2008) A hybrid cellular automaton model of clonal evolution in cancer: the emergence of the glycolytic phenotype. J Theor Biol 250: 705–722 18068192 10.1016/j.jtbi.2007.10.038 PMC2846650

[msb202211127-bib-0041] Getz M , Swanson L , Sahoo D , Ghosh P , Rangamani P (2019) A predictive computational model reveals that GIV/girdin serves as a tunable valve for EGFR‐stimulated cyclic AMP signals. Mol Biol Cell 30: 1621–1633 31017840 10.1091/mbc.E18-10-0630 PMC6727633

[msb202211127-bib-0042] Ghosh P (2015) Heterotrimeric G proteins as emerging targets for network based therapy in cancer: end of a long futile campaign striking heads of a hydra. Aging (Albany NY) 7: 469–474 26224586 10.18632/aging.100781 PMC4543036

[msb202211127-bib-0043] Ghosh P , Beas AO , Bornheimer SJ , Garcia‐Marcos M , Forry EP , Johannson C , Ear J , Jung BH , Cabrera B , Carethers JM *et al* (2010) A G{alpha}i‐GIV molecular complex binds epidermal growth factor receptor and determines whether cells migrate or proliferate. Mol Biol Cell 21: 2338–2354 20462955 10.1091/mbc.E10-01-0028 PMC2893996

[msb202211127-bib-0044] Ghosh P , Aznar N , Swanson L , Lo IC , Lopez‐Sanchez I , Ear J , Rohena C , Kalogriopoulos N , Joosen L , Dunkel Y (2016a) Biochemical, biophysical and cellular techniques to study the guanine nucleotide exchange factor, GIV/Girdin. Curr Protoc Chem Biol 8: 265–298 27925669 10.1002/cpch.13 PMC5154557

[msb202211127-bib-0045] Ghosh P , Tie J , Muranyi A , Singh S , Brunhoeber P , Leith K , Bowermaster R , Liao Z , Zhu Y , LaFleur B *et al* (2016b) Girdin (GIV) expression as a prognostic marker of recurrence in mismatch repair‐proficient stage II colon cancer. Clin Cancer Res 22: 3488–3498 27029492 10.1158/1078-0432.CCR-15-2290 PMC4947424

[msb202211127-bib-0046] Gibson SK , Gilman AG (2006) Giα and Gβ subunits both define selectivity of G protein activation by α2‐adrenergic receptors. Proc Natl Acad Sci USA 103: 212–217 16371464 10.1073/pnas.0509763102 PMC1325004

[msb202211127-bib-0047] Go CD , Knight JDR , Rajasekharan A , Rathod B , Hesketh GG , Abe KT , Youn JY , Samavarchi‐Tehrani P , Zhang H , Zhu LY *et al* (2021) A proximity‐dependent biotinylation map of a human cell. Nature 595: 120–124 34079125 10.1038/s41586-021-03592-2

[msb202211127-bib-0048] Ha A , Polyanovsky A , Avidor‐Reiss T (2015) Drosophila Hook‐related protein (Girdin) is essential for sensory dendrite formation. Genetics 200: 1149–1159 26058848 10.1534/genetics.115.178954 PMC4574259

[msb202211127-bib-0049] Haines E , Saucier C , Claing A (2014) The adaptor proteins p66Shc and Grb2 regulate the activation of the GTPases ARF1 and ARF6 in invasive breast cancer cells. J Biol Chem 289: 5687–5703 24407288 10.1074/jbc.M113.516047 PMC3937643

[msb202211127-bib-0050] Haines E , Schlienger S , Claing A (2015) The small GTPase ADP‐ribosylation factor 1 mediates the sensitivity of triple negative breast cancer cells to EGFR tyrosine kinase inhibitors. Cancer Biol Ther 16: 1535–1547 26176330 10.1080/15384047.2015.1071737 PMC4846185

[msb202211127-bib-0051] Hanahan D , Weinberg RA (2000) The hallmarks of cancer. Cell 100: 57–70 10647931 10.1016/s0092-8674(00)81683-9

[msb202211127-bib-0052] Hart Y , Reich‐Zeliger S , Antebi YE , Zaretsky I , Mayo AE , Alon U , Friedman N (2014) Paradoxical signaling by a secreted molecule leads to homeostasis of cell levels. Cell 158: 1022–1032 25171404 10.1016/j.cell.2014.07.033

[msb202211127-bib-0053] Houssin E , Tepass U , Laprise P (2015) Girdin‐mediated interactions between cadherin and the Actin cytoskeleton are required for epithelial morphogenesis in drosophila. Development 142: 1777–1784 25968313 10.1242/dev.122002

[msb202211127-bib-0054] Jayamohan Pillai CS , Chatterjee A , Geetha M , Mukherjee A (2022) MultiViz: a Gephi plugin for scalable visualization of multi‐layer networks. arXiv 10.48550/arXiv.2209.03149 [PREPRINT]

[msb202211127-bib-0055] Jiang P , Enomoto A , Jijiwa M , Kato T , Hasegawa T , Ishida M , Sato T , Asai N , Murakumo Y , Takahashi M (2008) An Actin‐binding protein Girdin regulates the motility of breast cancer cells. Cancer Res 68: 1310–1318 18316593 10.1158/0008-5472.CAN-07-5111

[msb202211127-bib-0056] Kahn RA , Gilman AG (1986) The protein cofactor necessary for ADP‐ribosylation of Gs by cholera toxin is itself a GTP binding protein. J Biol Chem 261: 7906–7911 3086320

[msb202211127-bib-0057] Kalogriopoulos NA , Rees SD , Ngo T , Kopcho NJ , Ilatovskiy AV , Sun N , Komives EA , Chang G , Ghosh P , Kufareva I (2019) Structural basis for GPCR‐independent activation of heterotrimeric Gi proteins. Proc Natl Acad Sci USA 116: 16394–16403 31363053 10.1073/pnas.1906658116 PMC6697900

[msb202211127-bib-0058] Kalogriopoulos NA , Lopez‐Sanchez I , Lin C , Ngo T , Midde KK , Roy S , Aznar N , Murray F , Garcia‐Marcos M , Kufareva I *et al* (2020) Receptor tyrosine kinases activate heterotrimeric G proteins via phosphorylation within the interdomain cleft of Gαi. Proc Natl Acad Sci USA 117: 28763–28774 33139573 10.1073/pnas.2004699117 PMC7682395

[msb202211127-bib-0059] Kamino K , Kondo Y , Nakajima A , Honda‐Kitahara M , Kaneko K , Sawai S (2017) Fold‐change detection and scale invariance of cell‐cell signaling in social amoeba. Proc Natl Acad Sci USA 114: E4149–E4157 28495969 10.1073/pnas.1702181114 PMC5448168

[msb202211127-bib-0060] Kean MJ , Williams KC , Skalski M , Myers D , Burtnik A , Foster D , Coppolino MG (2009) VAMP3, syntaxin‐13 and SNAP23 are involved in secretion of matrix metalloproteinases, degradation of the extracellular matrix and cell invasion. J Cell Sci 122: 4089–4098 19910495 10.1242/jcs.052761

[msb202211127-bib-0061] Kelly RB (1985) Pathways of protein secretion in eukaryotes. Science 230: 25–32 2994224 10.1126/science.2994224

[msb202211127-bib-0062] Kime CR , Mano MM (2003) Logic and computer design fundamentals. Hoboken, NJ: Prentice Hall

[msb202211127-bib-0063] Kloeden PE , Platen E (1992) Strong Taylor approximations. In Numerical Solution of Stochastic Differential Equations, Kloeden PE , Platen E (eds), pp 339–371. Berlin, Heidelberg: Springer Berlin Heidelberg

[msb202211127-bib-0064] Lane JR , Henderson D , Powney B , Wise A , Rees S , Daniels D , Plumpton C , Kinghorn I , Milligan G (2008) Antibodies that identify only the active conformation of G(i) family G protein alpha subunits. FASEB J 22: 1924–1932 18199696 10.1096/fj.07-100388

[msb202211127-bib-0065] Li X , Thirumalai D (2019) Share, but unequally: a plausible mechanism for emergence and maintenance of intratumour heterogeneity. J R Soc Interface 16: 20180820 30958159 10.1098/rsif.2018.0820 PMC6364648

[msb202211127-bib-0066] Lo I‐C , Gupta V , Midde KK , Taupin V , Lopez‐Sanchez I , Kufareva I , Abagyan R , Randazzo PA , Farquhar MG , Ghosh P (2015) Activation of Gαi at the Golgi by GIV/Girdin imposes finiteness in Arf1 signaling. Dev Cell 33: 189–203 25865347 10.1016/j.devcel.2015.02.009 PMC4415880

[msb202211127-bib-0067] Lopez‐Sanchez I , Dunkel Y , Roh Y‐S , Mittal Y , De Minicis S , Muranyi A , Singh S , Shanmugam K , Aroonsakool N , Murray F (2014) GIV/Girdin is a central hub for profibrogenic signalling networks during liver fibrosis. Nat Commun 5: 1–18 10.1038/ncomms5451 PMC4107319 25043713

[msb202211127-bib-0068] Lopez‐Sanchez I , Kalogriopoulos N , Lo I‐C , Kabir F , Midde KK , Wang H , Ghosh P (2015) Focal adhesions are foci for tyrosine‐based signal transduction via GIV/Girdin and G proteins. Mol Biol Cell 26: 4313–4324 26446841 10.1091/mbc.E15-07-0496 PMC4666128

[msb202211127-bib-0069] Luchsinger C , Aguilar M , Burgos PV , Ehrenfeld P , Mardones GA (2018) Functional disruption of the Golgi apparatus protein ARF1 sensitizes MDA‐MB‐231 breast cancer cells to the antitumor drugs actinomycin D and vinblastine through ERK and AKT signaling. PLoS ONE 13: e0195401 29614107 10.1371/journal.pone.0195401 PMC5882166

[msb202211127-bib-0070] Ma GS , Aznar N , Kalogriopoulos N , Midde KK , Lopez‐Sanchez I , Sato E , Dunkel Y , Gallo RL , Ghosh P (2015) Therapeutic effects of cell‐permeant peptides that activate G proteins downstream of growth factors. Proc Natl Acad Sci USA 112: E2602–E2610 25926659 10.1073/pnas.1505543112 PMC4443320

[msb202211127-bib-0071] Maire T , Youk H (2015) Molecular‐level tuning of cellular autonomy controls the collective behaviors of cell populations. Cell Syst 1: 349–360 27136241 10.1016/j.cels.2015.10.012

[msb202211127-bib-0072] Maley CC , Aktipis A , Graham TA , Sottoriva A , Boddy AM , Janiszewska M , Silva AS , Gerlinger M , Yuan Y , Pienta KJ *et al* (2017) Classifying the evolutionary and ecological features of neoplasms. Nat Rev Cancer 17: 605–619 28912577 10.1038/nrc.2017.69 PMC5811185

[msb202211127-bib-0073] Marshansky V , Bourgoin S , Londono I , Bendayan M , Vinay P (1997) Identification of ADP‐ribosylation factor‐6 in brush‐border membrane and early endosomes of human kidney proximal tubules. Electrophoresis 18: 538–547 9150938 10.1002/elps.1150180334

[msb202211127-bib-0074] Matlin KS , Caplan MJ (2017) The secretory pathway at 50: a golden anniversary for some momentous grains of silver. Mol Biol Cell 28: 229–232 28082520 10.1091/mbc.E16-07-0508 PMC5231891

[msb202211127-bib-0075] Merlo LMF , Pepper JW , Reid BJ , Maley CC (2006) Cancer as an evolutionary and ecological process. Nat Rev Cancer 6: 924–935 17109012 10.1038/nrc2013

[msb202211127-bib-0076] Midde K , Rich R , Marandos P , Fudala R , Li A , Gryczynski I , Borejdo J (2013) Comparison of orientation and rotational motion of skeletal muscle cross‐bridges containing phosphorylated and dephosphorylated myosin regulatory light chain. J Biol Chem 288: 7012–7023 23319584 10.1074/jbc.M112.434209 PMC3591611

[msb202211127-bib-0077] Midde K , Rich R , Saxena A , Gryczynski I , Borejdo J , Das HK (2014) Membrane topology of human presenilin‐1 in SK‐N‐SH cells determined by fluorescence correlation spectroscopy and fluorescent energy transfer. Cell Biochem Biophys 70: 923–932 24839116 10.1007/s12013-014-9999-z

[msb202211127-bib-0078] Midde KK , Aznar N , Laederich MB , Ma GS , Kunkel MT , Newton AC , Ghosh P (2015) Multimodular biosensors reveal a novel platform for activation of G proteins by growth factor receptors. Proc Natl Acad Sci USA 112: E937–E946 25713130 10.1073/pnas.1420140112 PMC4352799

[msb202211127-bib-0079] Midde K , Sun N , Rohena C , Joosen L , Dhillon H , Ghosh P (2018) Single‐cell imaging of metastatic potential of cancer cells. iScience 10: 53–65 30500482 10.1016/j.isci.2018.11.022 PMC6263091

[msb202211127-bib-0080] Milo R , Shen‐Orr S , Itzkovitz S , Kashtan N , Chklovskii D , Alon U (2002) Network motifs: simple building blocks of complex networks. Science 298: 824–827 12399590 10.1126/science.298.5594.824

[msb202211127-bib-0081] Nechipurenko IV , Olivier‐Mason A , Kazatskaya A , Kennedy J , McLachlan IG , Heiman MG , Blacque OE , Sengupta P (2016) A conserved role for Girdin in basal body positioning and Ciliogenesis. Dev Cell 38: 493–506 27623382 10.1016/j.devcel.2016.07.013 PMC5023068

[msb202211127-bib-0082] Norton K‐A , Popel AS (2014) An agent‐based model of cancer stem cell initiated avascular tumour growth and metastasis: the effect of seeding frequency and location. J R Soc Interface 11: 20140640 25185580 10.1098/rsif.2014.0640 PMC4191089

[msb202211127-bib-0083] Núñez‐Olvera SI , Chávez‐Munguía B , del Rocío Terrones‐Gurrola MC , Marchat LA , Puente‐Rivera J , Ruíz‐García E , Campos‐Parra AD , Vázquez‐Calzada C , Lizárraga‐Verdugo ER , Ramos‐Payán R *et al* (2020) A novel protective role for microRNA‐3135b in Golgi apparatus fragmentation induced by chemotherapy via GOLPH3/AKT1/mTOR axis in colorectal cancer cells. Sci Rep 10: 10555 32601379 10.1038/s41598-020-67550-0 PMC7324564

[msb202211127-bib-0084] Ohashi Y , Iijima H , Yamaotsu N , Yamazaki K , Sato S , Okamura M , Sugimoto K , Dan S , Hirono S , Yamori T (2012) AMF‐26, a novel inhibitor of the Golgi system, targeting ADP‐ribosylation factor 1 (Arf1) with potential for cancer therapy. J Biol Chem 287: 3885–3897 22158626 10.1074/jbc.M111.316125 PMC3281721

[msb202211127-bib-0085] Ohashi Y , Okamura M , Hirosawa A , Tamaki N , Akatsuka A , Wu K‐M , Choi H‐W , Yoshimatsu K , Shiina I , Yamori T *et al* (2016) M‐COPA, a Golgi disruptor, inhibits cell surface expression of MET protein and exhibits antitumor activity against MET‐addicted gastric cancers. Cancer Res 76: 3895–3903 27197184 10.1158/0008-5472.CAN-15-2220

[msb202211127-bib-0086] Ohashi Y , Okamura M , Katayama R , Fang S , Tsutsui S , Akatsuka A , Shan M , Choi H‐W , Fujita N , Yoshimatsu K *et al* (2017) Targeting the Golgi apparatus to overcome acquired resistance of non‐small cell lung cancer cells to EGFR tyrosine kinase inhibitors. Oncotarget 9: 1641–1655 29416720 10.18632/oncotarget.22895 PMC5788588

[msb202211127-bib-0087] Oughtred R , Rust J , Chang C , Breitkreutz BJ , Stark C , Willems A , Boucher L , Leung G , Kolas N , Zhang F *et al* (2021) The BioGRID database: a comprehensive biomedical resource of curated protein, genetic, and chemical interactions. Protein Sci 30: 187–200 33070389 10.1002/pro.3978 PMC7737760

[msb202211127-bib-0088] Perez‐Riverol Y , Bai J , Bandla C , García‐Seisdedos D , Hewapathirana S , Kamatchinathan S , Kundu DJ , Prakash A , Frericks‐Zipper A , Eisenacher M *et al* (2022) The PRIDE database resources in 2022: a hub for mass spectrometry‐based proteomics evidences. Nucleic Acids Res 50: D543–D552 34723319 10.1093/nar/gkab1038 PMC8728295

[msb202211127-bib-0089] Petrosyan A (2015) Onco‐Golgi: is fragmentation a gate to cancer progression? Biochem Mol Biol J 1: 16 27064441 10.21767/2471-8084.100006 PMC4824322

[msb202211127-bib-0090] Poleszczuk J , Hahnfeldt P , Enderling H (2015) Evolution and phenotypic selection of cancer stem cells. PLoS Comput Biol 11: e1004025 25742563 10.1371/journal.pcbi.1004025 PMC4351192

[msb202211127-bib-0091] Presley JF , Cole NB , Schroer TA , Hirschberg K , Zaal KJ , Lippincott‐Schwartz J (1997) ER‐to‐Golgi transport visualized in living cells. Nature 389: 81–85 9288971 10.1038/38001

[msb202211127-bib-0092] Prieto‐Dominguez N , Parnell C , Teng Y (2019) Drugging the small GTPase pathways in cancer treatment: promises and challenges. Cell 8: 255 10.3390/cells8030255 PMC6468615 30884855

[msb202211127-bib-0093] Puseenam A , Yoshioka Y , Nagai R , Hashimoto R , Suyari O , Itoh M , Enomoto A , Takahashi M , Yamaguchi M (2009) A novel drosophila Girdin‐like protein is involved in Akt pathway control of cell size. Exp Cell Res 315: 3370–3380 19560458 10.1016/j.yexcr.2009.06.019

[msb202211127-bib-0094] Qiao L , Ghosh P , Rangamani P (2023) Design principles of improving the dose‐response alignment in coupled GTPase switches. NPJ Syst Biol Appl 9: 3 36720885 10.1038/s41540-023-00266-9 PMC9889403

[msb202211127-bib-0095] Rahman‐Zaman A , Shan S , Reinhart‐King CA (2018) Cell migration in microfabricated 3D collagen microtracks is mediated through the prometastatic protein girdin. Cell Mol Bioeng 11: 1–10 29403565 10.1007/s12195-017-0511-x PMC5795616

[msb202211127-bib-0096] Rauter T , Burgstaller S , Gottschalk B , Ramadani‐Muja J , Bischof H , Hay JC , Graier WF , Malli R (2020) ER‐to‐Golgi transport in HeLa cells displays high resilience to Ca^2+^ and energy stresses. Cells 9: 2311 33080790 10.3390/cells9102311 PMC7603030

[msb202211127-bib-0097] Reddy RJ , Gajadhar AS , Swenson EJ , Rothenberg DA , Curran TG , White FM (2016) Early signaling dynamics of the epidermal growth factor receptor. Proc Natl Acad Sci USA 113: 3114–3119 26929352 10.1073/pnas.1521288113 PMC4801278

[msb202211127-bib-0098] Rohena C , Rajapakse N , Lo I‐C , Novick P , Sahoo D , Ghosh P (2020) GIV/Girdin and Exo70 collaboratively regulate the mammalian polarized exocytic machinery. iScience 23: 101246 32590327 10.1016/j.isci.2020.101246 PMC7322189

[msb202211127-bib-0099] Roszik J , Lisboa D , Szöllősi J , Vereb G (2009) Evaluation of intensity‐based ratiometric FRET in image cytometry—approaches and a software solution. Cytometry A 75: 761–767 19591240 10.1002/cyto.a.20747

[msb202211127-bib-0100] Rothman JE , Orci L (1992) Molecular dissection of the secretory pathway. Nature 355: 409–415 1734280 10.1038/355409a0

[msb202211127-bib-0101] Sasaki K , Kakuwa T , Akimoto K , Koga H , Ohno S (2015) Regulation of epithelial cell polarity by PAR‐3 depends on Girdin transcription and Girdin‐Galphai3 signaling. J Cell Sci 128: 2244–2258 25977476 10.1242/jcs.160879

[msb202211127-bib-0102] Saucerman JJ , McCulloch AD (2004) Mechanistic systems models of cell signaling networks: a case study of myocyte adrenergic regulation. Prog Biophys Mol Biol 85: 261–278 15142747 10.1016/j.pbiomolbio.2004.01.005

[msb202211127-bib-0103] Shaul YD , Seger R (2006) ERK1c regulates Golgi fragmentation during mitosis. J Cell Biol 172: 885–897 16533948 10.1083/jcb.200509063 PMC2063732

[msb202211127-bib-0104] Shen‐Orr SS , Milo R , Mangan S , Alon U (2002) Network motifs in the transcriptional regulation network of *Escherichia coli* . Nat Genet 31: 64–68 11967538 10.1038/ng881

[msb202211127-bib-0105] Sinha S , Samaddar S , Das Gupta SK , Roy S (2021) Network approach to mutagenesis sheds insight on phage resistance in mycobacteria. Bioinformatics 37: 213–220 33416849 10.1093/bioinformatics/btaa1103

[msb202211127-bib-0106] Sinha S , Farfel A , Luker KE , Sahoo D , Parker BA , Yeung K , Luker GD , Ghosh P (2022) Growth signaling autonomy in circulating tumor cells aids metastatic seeding. bioRxiv 10.1101/2022.12.02.518910 [PREPRINT] PMC10833458 38312224

[msb202211127-bib-0107] Sottoriva A , Verhoeff JJC , Borovski T , McWeeney SK , Naumov L , Medema JP , Sloot PMA , Vermeulen L (2010) Cancer stem cell tumor model reveals invasive morphology and increased phenotypical heterogeneity. Cancer Res 70: 46–56 20048071 10.1158/0008-5472.CAN-09-3663

[msb202211127-bib-0108] Stearns T , Willingham MC , Botstein D , Kahn RA (1990) ADP‐ribosylation factor is functionally and physically associated with the Golgi complex. Proc Natl Acad Sci USA 87: 1238–1242 2105501 10.1073/pnas.87.3.1238 PMC53446

[msb202211127-bib-0109] Stolerman LM , Ghosh P , Rangamani P (2021) Stability analysis of a signaling circuit with dual species of GTPase switches. Bull Math Biol 83: 34 33609194 10.1007/s11538-021-00864-w PMC8378325

[msb202211127-bib-0110] Stow JL , de Almeida JB , Narula N , Holtzman EJ , Ercolani L , Ausiello DA (1991) A heterotrimeric G protein, G alpha i‐3, on Golgi membranes regulates the secretion of a heparan sulfate proteoglycan in LLC‐PK1 epithelial cells. J Cell Biol 114: 1113–1124 1910049 10.1083/jcb.114.6.1113 PMC2289129

[msb202211127-bib-0111] Tabassum DP , Polyak K (2015) Tumorigenesis: it takes a village. Nat Rev Cancer 15: 473–483 26156638 10.1038/nrc3971

[msb202211127-bib-0112] Tang Y , Rijal R , Zimmerhanzel David E , McCullough Jacquelyn R , Cadena Louis A , Gomer Richard H , Heitman J (2021) An autocrine negative feedback loop inhibits *Dictyostelium discoideum* proliferation through pathways including IP3/Ca^2+^ . mBio 12: e0134721 34154396 10.1128/mBio.01347-21 PMC8262924

[msb202211127-bib-0113] Tanigawa G , Orci L , Amherdt M , Ravazzola M , Helms JB , Rothman JE (1993) Hydrolysis of bound GTP by ARF protein triggers uncoating of Golgi‐derived COP‐coated vesicles. J Cell Biol 123: 1365–1371 8253837 10.1083/jcb.123.6.1365 PMC2290881

[msb202211127-bib-0114] The UniProt C (2019) UniProt: a worldwide hub of protein knowledge. Nucleic Acids Res 47: D506–D515 30395287 10.1093/nar/gky1049 PMC6323992

[msb202211127-bib-0115] Trombetta ES , Parodi AJ (2003) Quality control and protein folding in the secretory pathway. Annu Rev Cell Dev Biol 19: 649–676 14570585 10.1146/annurev.cellbio.19.110701.153949

[msb202211127-bib-0116] Uhlén M , Fagerberg L , Hallström BM , Lindskog C , Oksvold P , Mardinoglu A , Sivertsson Å , Kampf C , Sjöstedt E , Asplund A *et al* (2015) Tissue‐based map of the human proteome. Science 347: 1260419 25613900 10.1126/science.1260419

[msb202211127-bib-0117] Velasco A , Hendricks L , Moremen KW , Tulsiani DR , Touster O , Farquhar MG (1993) Cell type‐dependent variations in the subcellular distribution of alpha‐mannosidase I and II. J Cell Biol 122: 39–51 8314846 10.1083/jcb.122.1.39 PMC2119607

[msb202211127-bib-0118] Wang Z , Zhang L , Sagotsky J , Deisboeck TS (2007) Simulating non‐small cell lung cancer with a multiscale agent‐based model. Theor Biol Med Model 4: 50 18154660 10.1186/1742-4682-4-50 PMC2259313

[msb202211127-bib-0119] Wang A , Wang J , Sun L , Jin J , Ren H , Yang F , Diao K , Wei M , Mi X (2015) Expression of tumor necrosis factor receptor‐assicated factor 4 correlates with expression of Girdin and promotes nuclear translocation of Girdin in breast cancer. Mol Med Rep 11: 3635–3641 25591657 10.3892/mmr.2015.3211

[msb202211127-bib-0120] Wang H , Zhang J , Zhang M , Wei L , Chen H , Li Z (2017) A systematic study of Girdin on cell proliferation, migration and angiogenesis in different breast cancer subtypes. Mol Med Rep 16: 3351–3356 28713924 10.3892/mmr.2017.6971

[msb202211127-bib-0121] Wlodkowic D , Skommer J , McGuinness D , Hillier C , Darzynkiewicz Z (2009) ER–Golgi network—a future target for anti‐cancer therapy. Leuk Res 33: 1440–1447 19595459 10.1016/j.leukres.2009.05.025 PMC2749752

[msb202211127-bib-0122] Wortzel I , Koifman G , Rotter V , Seger R , Porat Z (2017) High throughput analysis of Golgi structure by imaging flow cytometry. Sci Rep 7: 788 28400563 10.1038/s41598-017-00909-y PMC5429768

[msb202211127-bib-0123] Yamaguchi M , Suyari O , Nagai R , Takahashi M (2010) dGirdin a new player of Akt/PKB signaling in *Drosophila melanogaster* . Front Biosci (Landmark Ed) 15: 1164–1171 20515748 10.2741/3668

[msb202211127-bib-0124] Youk H , Lim WA (2014) Secreting and sensing the same molecule allows cells to achieve versatile social behaviors. Science 343: 1242782 24503857 10.1126/science.1242782 PMC4145839

[msb202211127-bib-0125] Zhang X (2021) Alterations of Golgi structural proteins and glycosylation defects in cancer. Front Cell Dev Biol 9: 665289 34055798 10.3389/fcell.2021.665289 PMC8149618

[msb202211127-bib-0126] Zuber C , Spiro MJ , Guhl B , Spiro RG , Roth J (2000) Golgi apparatus immunolocalization of Endomannosidase suggests post‐endoplasmic reticulum glucose trimming: implications for quality control. Mol Biol Cell 11: 4227–4240 11102520 10.1091/mbc.11.12.4227 PMC15069

