# Supplementary material for: A circuit for secretion‐coupled cellular autonomy in multicellular eukaryotic cells
Source: Mol Syst Biol. 2023 Mar 1;19(4):e11127. doi: 10.15252/msb.202211127 (PMC10090951; doi:10.15252/msb.202211127)
Supplement: Supplementary file 1 — Appendix [file MSB-19-e11127-s011.pdf]

Appendix to

A Circuit for Secretion-coupled Cellular Autonomy in Multicellular Eukaryotic cells

Lingxia Qiao[1†], Saptarshi Sinha[2†], Amer Ali Abd El-Hafeez[2¶], I-Chung Lo[2], Krishna K. Midde[2], Tony Ngo[3], Nicolas Aznar[2], Inmaculada Lopez-Sanchez[2], Vijay Gupta[2], Marilyn G. Farquhar[2€], Padmini Rangamani[1]* and Pradipta Ghosh[2, 4-6]*

Affiliations:
[1]Department of Mechanical and Aerospace Engineering, Jacob's School of Engineering, University of California San Diego, La Jolla, CA.
[2]Department of Cellular and Molecular Medicine, School of Medicine, University of California San Diego, La Jolla, CA.
[3]Skaggs School of Pharmacy and Pharmaceutical Science, University of California San Diego, La Jolla, CA.
[4]Moores Comprehensive Cancer Center, University of California San Diego.
[5]Department of Medicine, School of Medicine, University of California San Diego, La Jolla, CA.
[6]Veterans Affairs Medical Center, La Jolla, CA.

[†]These authors contributed equally.
[€]Acknowledged posthumously
*Correspondence to: prangamani@ucsd.edu and prghosh@ucsd.edu
[¶]Secondary Affiliation: Pharmacology and Experimental Oncology Unit, Cancer Biology Department, National Cancer Institute, Cairo University, Cairo, Egypt

Contents

Appendix Figure S1. 2
Appendix Figure S2. 3
Appendix Figure S3. 4
Appendix Figure S4. 5
Appendix Figure S5. 6

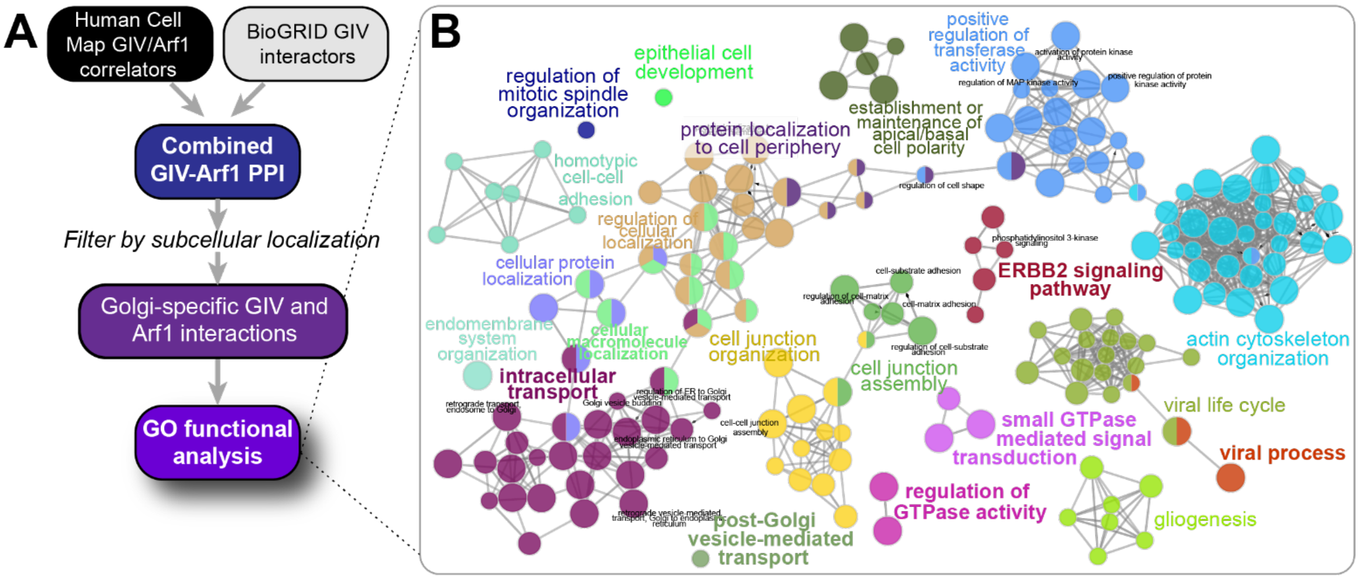

Appendix Figure S1. A comparative analysis of the Golgi-localized Arf1 (mG) connectome with/without coupling to GIV (tGEF) and Gi (tGTPase).

A. Workflow for extracting the proteins that bind Arf1 and GIV and are localized at the Golgi. The complete list of these proteins is provided as **Dataset EV1**.

B. Gene Ontology (GO) analysis of a Golgi-specific GIV and Arf1 interaction network, as visualized using ClueGO. Groups/clusters were collapsed and only those nodes are displayed that have the highest logP value. As anticipated, the list of proteins in such a Golgi-restricted interactome was enriched for cellular processes that are normally dependent on the secretory pathway, e.g., intracellular transport, post-Golgi vesicle transport, etc., and that require the secretory pathway, e.g., cell junctions, cell polarity, and growth factor signaling (see also **Appendix Figure S2A**).

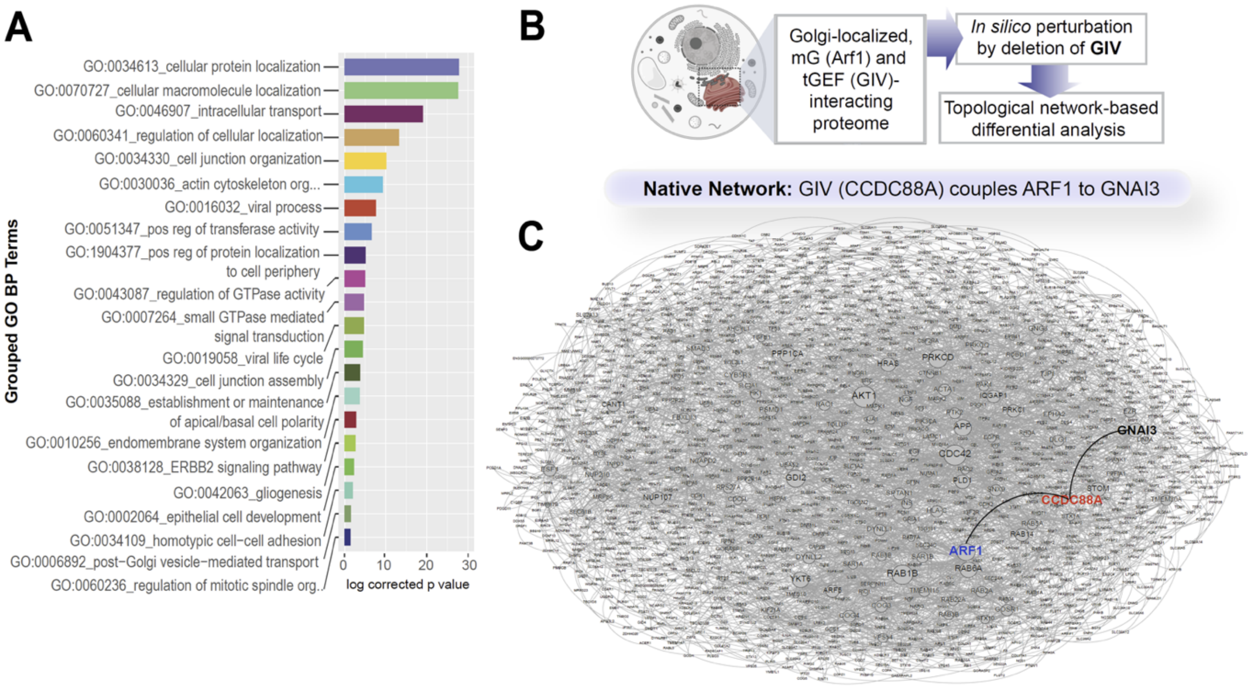

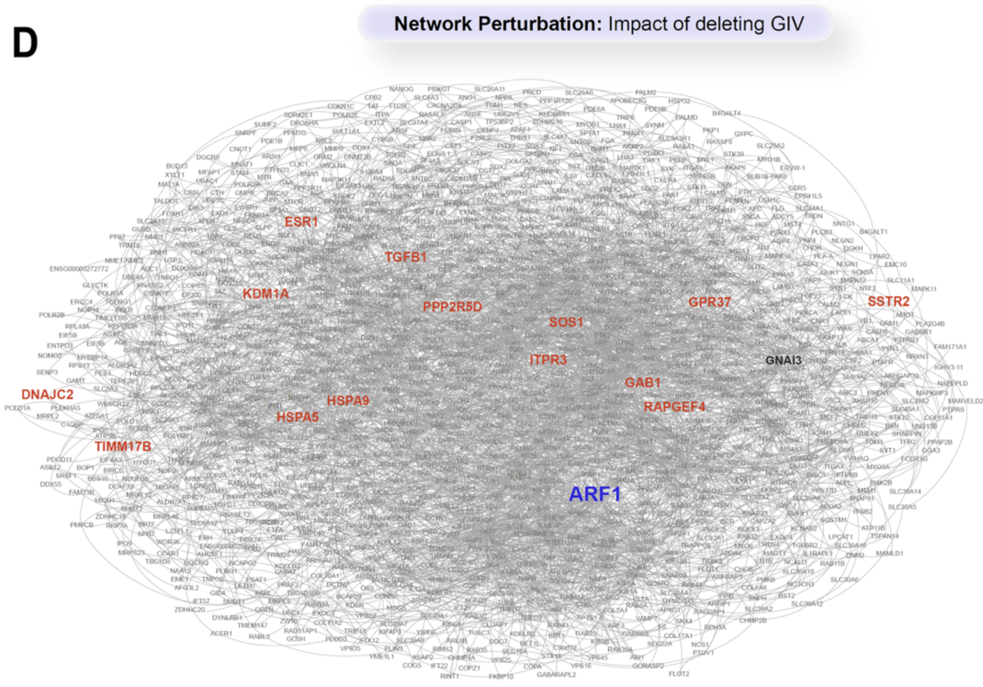

E

End proteins	Frequency of appearance	Z Score of frequency
GPR37	24	2.82
GAB1	23	2.648
TGFB1	20	2.133
RAPGEF4	20	2.133
SOS1	18	1.789
HSPA5	17	1.617
DNAJC2	17	1.617
KDM1A	16	1.446
ESR1	16	1.446
SSTR2	16	1.446
TIMM17B	15	1.274
PPP2R5D	15	1.274
ITPR3	15	1.274
HSPA9	14	1.102

Appendix Figure S2. Protein-Protein Interaction (PPI) network analysis to predict the functions of Golgi-localized coupled GTPases.

A. Gene Ontology biological processes (BP) analysis of a Golgi-specific GIV and Arf1 interaction network. See also **Dataset EV1** for the complete list of proteins.
B. Workflow schematic for PPI network analysis. See also **Figure 4C**.
C. A PPI network created using Golgi specific interactions of Arf1 and GIV fetched from HCM using Golgi specific proteins. Here the diameter of each node corresponds to its degree of connectivity within this PPI network. The interactions of GIV that allow it to serve as a linker between Arf1 (mGTPase) and GNAI3 (tGTPase) are highlighted.
D. Consequences of GIV(CCDC88A) deletion on the shortest paths alterations associated with Arf1. The end proteins of the most affected shortest paths have been highlighted in red.
E. Table displays a selected list of end proteins associated with altered shorted paths of Arf1 due to GIV deletion. The proteins are selected based on their frequency of appearance as end proteins in these shortest paths, using a Z score of the frequency >=1 as cut-off.

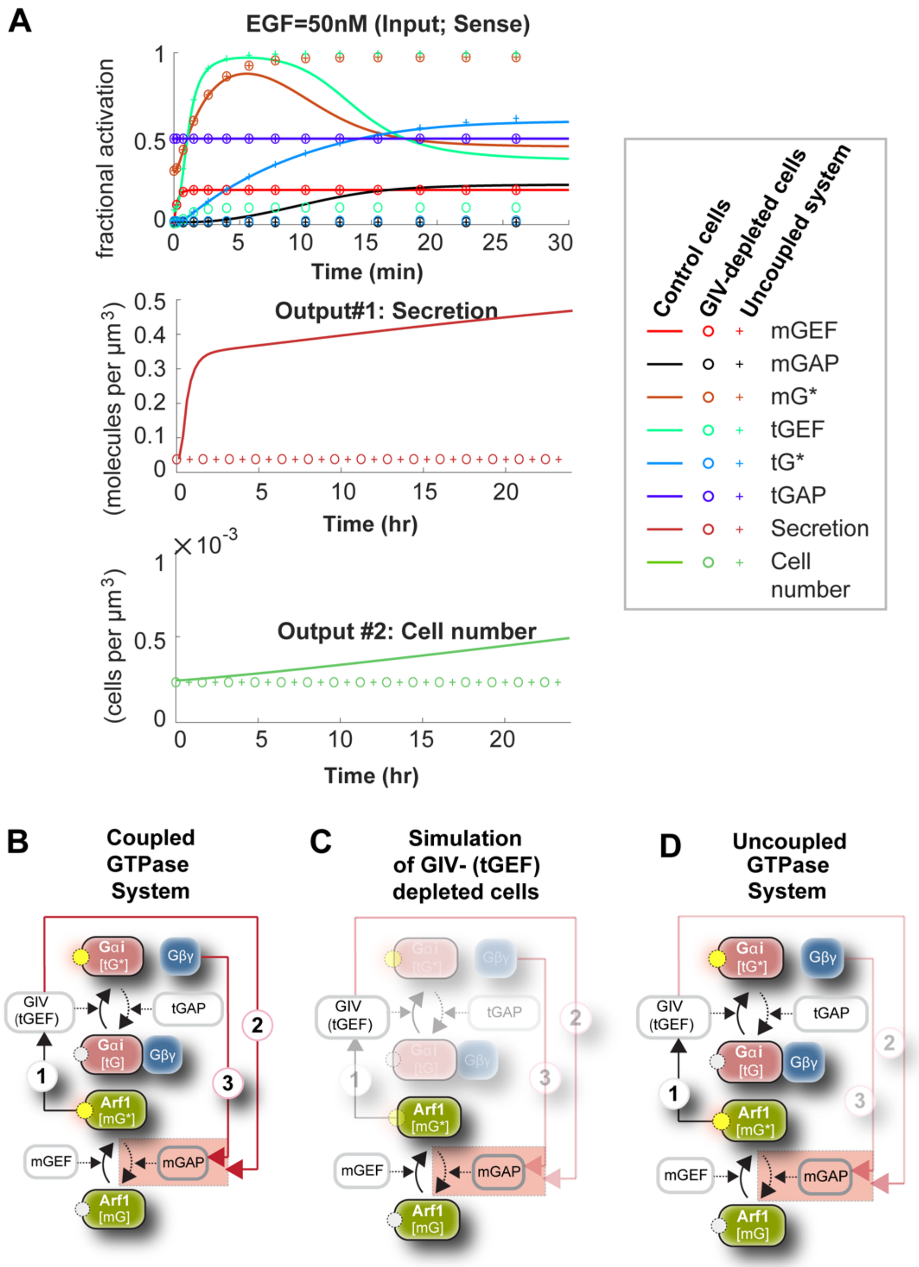

Appendix Figure S3. Simulations of secretion and cell number models for control cells (with a fully coupled GTPase system), in GIV-depleted cell (arrows 1-3) and cells with an uncoupled GTPase system (missing feedback control arrows 2 and 3).

A. Model-derived simulation of dynamics for control cells, GIV-depleted cells and the uncoupled system when EGF=50nM. Schematics in **B-D** display these three conditions. The initial condition is the steady state in serum-starved condition except that the cell number is set to be the carrying capacity K. The equations (1)-(7) and (9) with parameters in **Table EV1** are used, and the $stimulus$ changes to 0.23 at time zero.

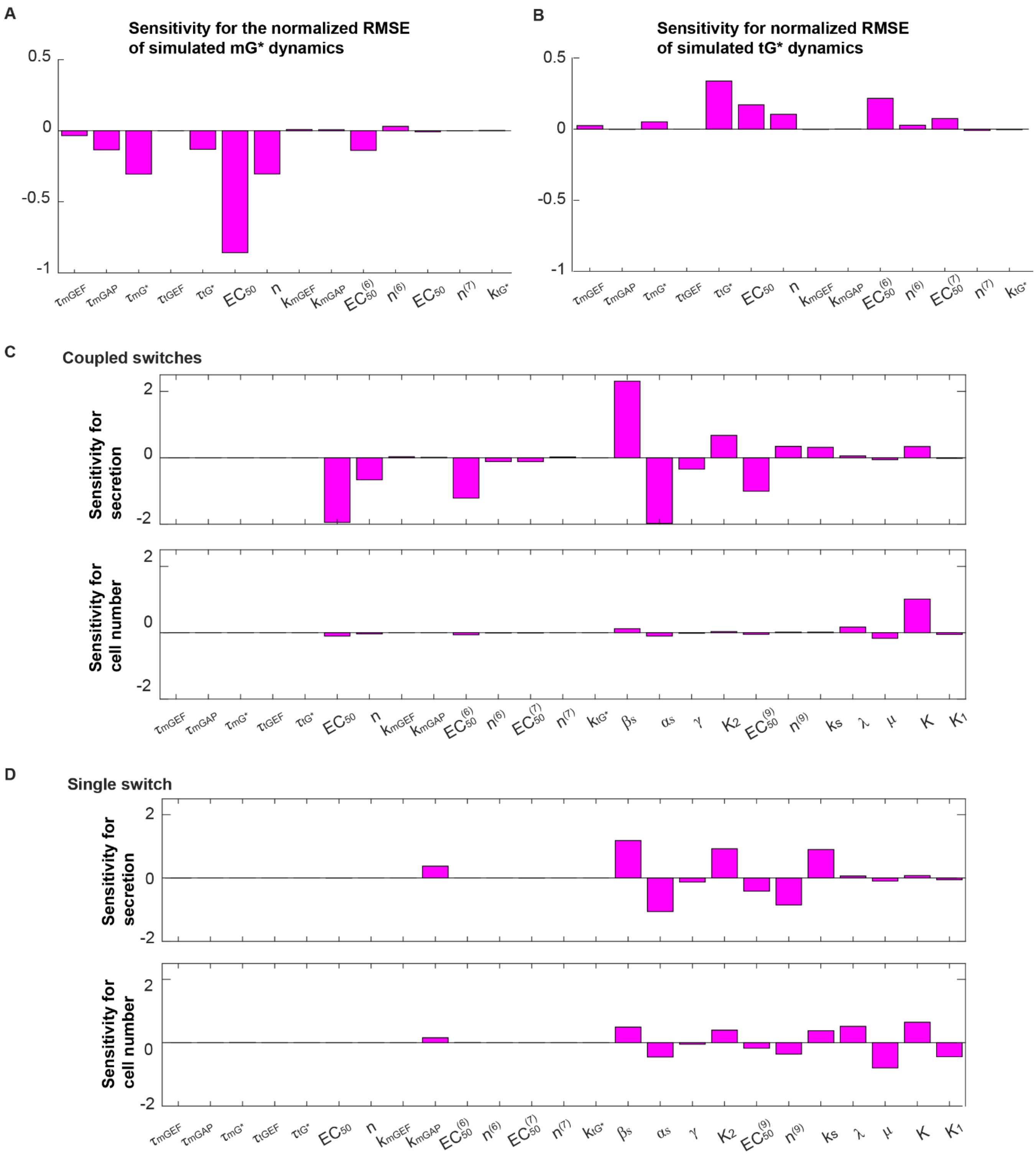

Appendix Figure S4. Sensitivity analysis*.*

A-B. Sensitivities for the normalized RMSE of simulated mG* (A) or tG* (B) dynamics. The normalized RMSE is calculated in the same way as in **Figure 2C** or **Figure 3G**.

C-D. Sensitivities for the steady-state values for the secretion and the cell number in coupled switches (C) or the single switch (D). The steady state values are calculated with $stimulus = 0.23$ to mimic the case when EGF=50 nM.

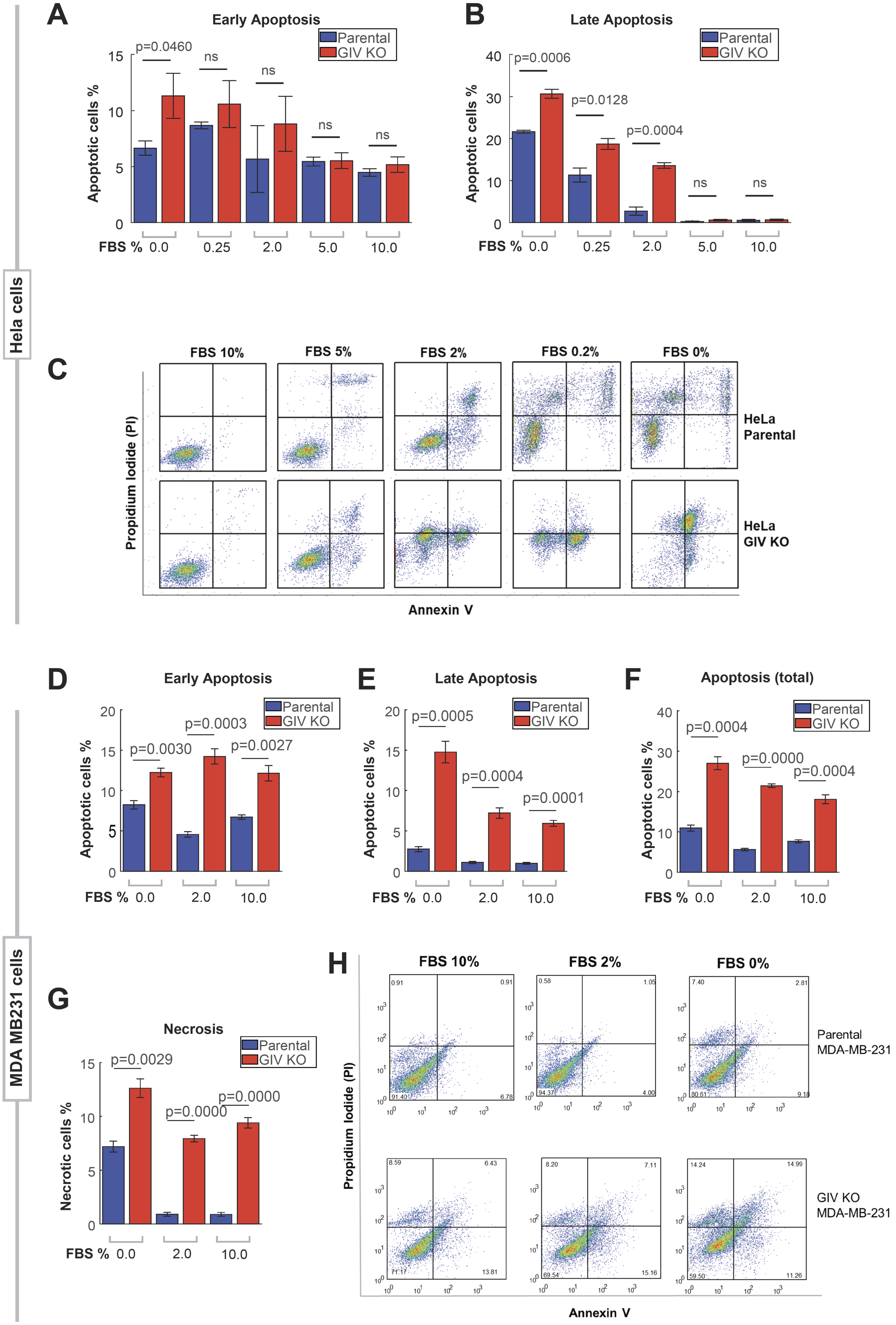

Appendix Figure S5. GIV-GEM is required for cell survival.

A-C. Bar graphs display the % early (A) or late (B) apoptotic control (parental) and GIV-depleted (GIV KO) HeLa cells after 24 h growth in varying concentration of serum, as assessed by annexin V staining and flow cytometry. The dot plot diagrams are shown in C.

D-H. Bar graphs display the % early (D), late (E) or total (early + late; F) apoptotic and necrotic (G) control (parental) and GIV-depleted (GIV KO) MDA MB-231 cells after 24 h growth in varying concentration of serum, as assessed by annexin V staining and flow cytometry. The dot plot diagrams are shown in H. Results are expressed as mean ± S.E.M; n = 3. p values were determined by unpaired t-test.
