# Supplementary material for: A circuit for secretion‐coupled cellular autonomy in multicellular eukaryotic cells
Source: Mol Syst Biol. 2023 Mar 1;19(4):e11127. doi: 10.15252/msb.202211127 (PMC10090951; doi:10.15252/msb.202211127)
Supplement: Supplementary file 2 — Expanded View Figures PDF [file MSB-19-e11127-s005.pdf]

Expanded View Figures

Figure EV1. An endomembrane network motif of two species of GTPases regulates membrane trafficking through the secretory pathway and regulates Golgi functions.

A Dynamics within the endomembrane GTPase system. Left to right panels display the deconstructed arrows denoting key molecular events/chemical reaction cascades within this system, in which, the GIV-GEM links the monomeric (m) and trimeric (t) GTPase systems and enables the conversion of extracellular stimulus (ligand; left) into membrane trafficking events (e.g., vesicle uncoating/budding/fusion; right). The forward and feedback reactions (arrows) are numbered 1–3. See Movie EV1 for a gif of the circuit.

B Schematic summarizing the findings reported by Lo *et al* (2015) delineating how arrows 1–3 within the endomembrane GTPase system regulate the finiteness of Arf1 signaling for efficient secretion.

 © 2023 The Authors

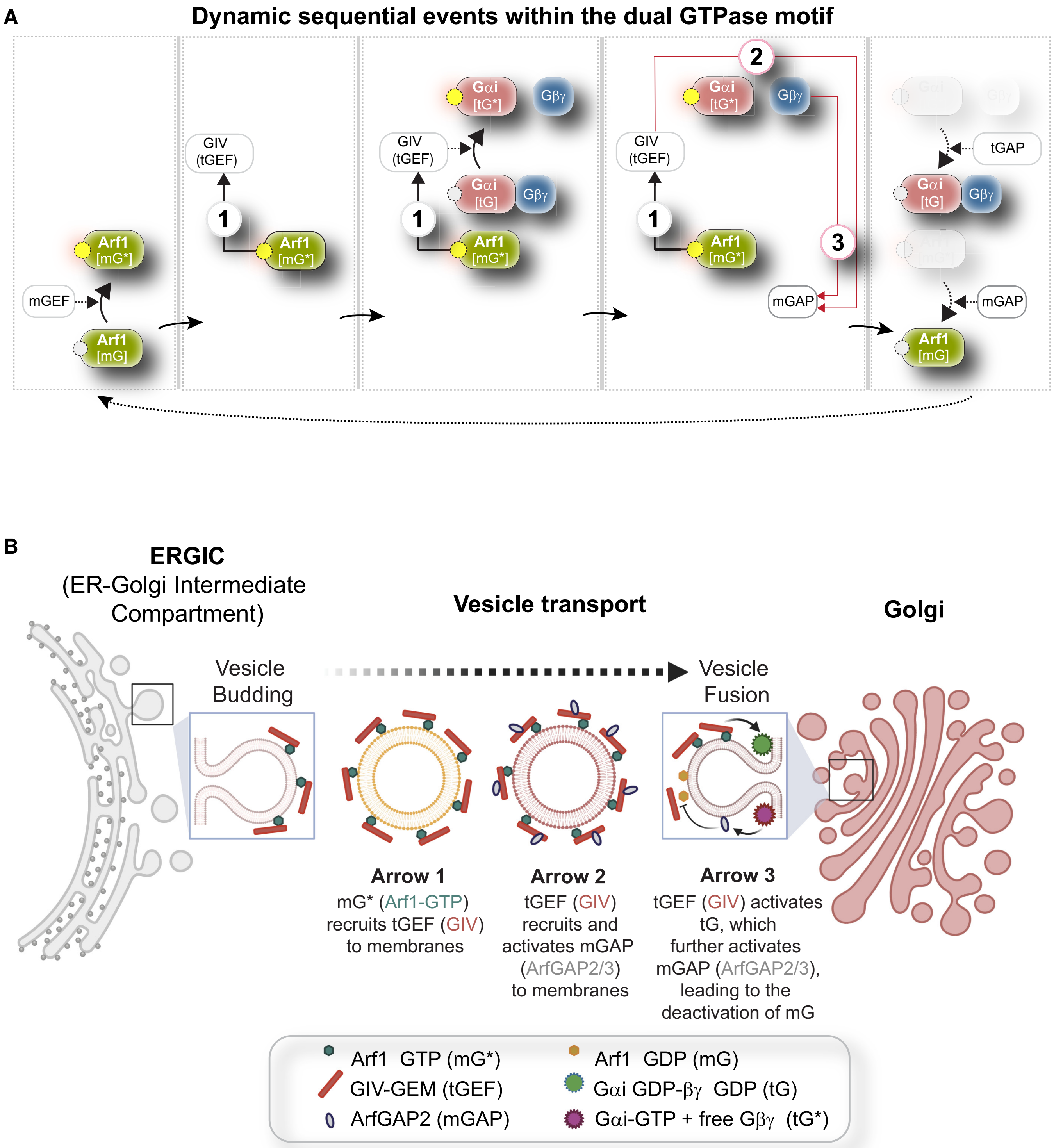
A
Dynamic sequential events within the dual GTPase motif
Arf1 [mG*]
mGEF
Arf1 [mG]
GIV (tGEF)
1
Gαi [tG*]
Gβγ
Gαi [tG]
2
3
mGAP
tGAP
B
ERGIC
(ER-Golgi Intermediate Compartment)
Vesicle transport
Golgi
Vesicle Budding
Vesicle Fusion
Arrow 1
mG* (Arf1-GTP) recruits tGEF (GIV) to membranes
Arrow 2
tGEF (GIV) recruits and activates mGAP (ArfGAP2/3) to membranes
Arrow 3
tGEF (GIV) activates tG, which further activates mGAP (ArfGAP2/3), leading to the deactivation of mG
Arf1 GTP (mG*)
GIV-GEM (tGEF)
ArfGAP2 (mGAP)
Arf1 GDP (mG)
Gαi GDP-βγ GDP (tG)
Gαi-GTP + free Gβγ (tG*)

Figure EV1.

Figure EV2. GEV-GEM is required for Gi activation at the Golgi and for maintaining the finiteness of Arf1 signaling upon EGF stimulation.

A FRET-based studies were carried out in sh Control cells as in Fig 3B and C. Briefly, HeLa cells were co-transfected with Gαi1-YFP, Gβ1-CFP and Gγ2 (untagged), and live cells were analyzed by FRET imaging at steady state, after being serum starved in 0.2% FBS overnight and then after stimulation with 50 nM EGF. Representative freeze-frame FRET images are shown. FRET image panels display intensities of acceptor emission due to efficient energy transfer in each pixel. The FRET scale is shown in the inset. Golgi and PM regions of interest are indicated with arrows. Scale bar = 10 μm.

B Bar graphs display the change in FRET at t5 min at the Golgi and the PM regions of 3–5 cells, from four independent biological replicates. Scale bar = 7.5 μm. Results are displayed as mean ± SEM. Statistical significance was determined by student *t*-test and the *P*-values are depicted as: $^{**}P < 0.01$; $^{****}P < 0.0001$.

C Schematic showing how a conformation-specific anti-Gαi·GTP antibody detects GTP-bound active Gαi *in situ*.

D HeLa cells starved with 0.2% FBS overnight or stimulated subsequently with 50 nM EGF or 250 μM LPA were fixed and stained for active Gαi (green; anti-Gαi:GTP mAb) and Man II (red) and analyzed by confocal microscopy. Activation of Gαi was detected exclusively after LPA/EGF stimulation. When detected, active Gαi colocalizes with Man II (yellow pixels in merge panel). Negative control (secondary antibody) staining was carried out on cells stimulated with EGF, 15 min. Scale bar = 10 μm.

E Control (sh Control) and GIV-depleted (shGIV) HeLa cells that were stimulated with EGF for the indicated time points prior to lysis were assessed for Arf1 activity. Immunoblots are shown in Fig 3I. Bar graphs display the fold change in Arf1 activity normalized to t0 min that was observed in control (shControl) and GIV-depleted (shGIV) cells. Results are expressed as mean ± SEM; $n = 3$ biological replicates; *P*-values were determined using Mann–Whitney *t*-test compared to t0: $^{*}P < 0.05$; $^{**}P < 0.01$; $^{***}P < 0.001$. Immunoblots are representative of findings from at least three independent repeats.

F Line graph in red displays a model-derived simulation of Arf1 activation dynamics (mG*) in cells without tGEF (shGIV). As a reference, the results of model-derived simulation fit to experimental data in control cells are displayed in blue, and the experimental data for Arf1 activation dynamics are shown as mean ± SEM ($n = 3$ biological replicates).

 © 2023 The Authors

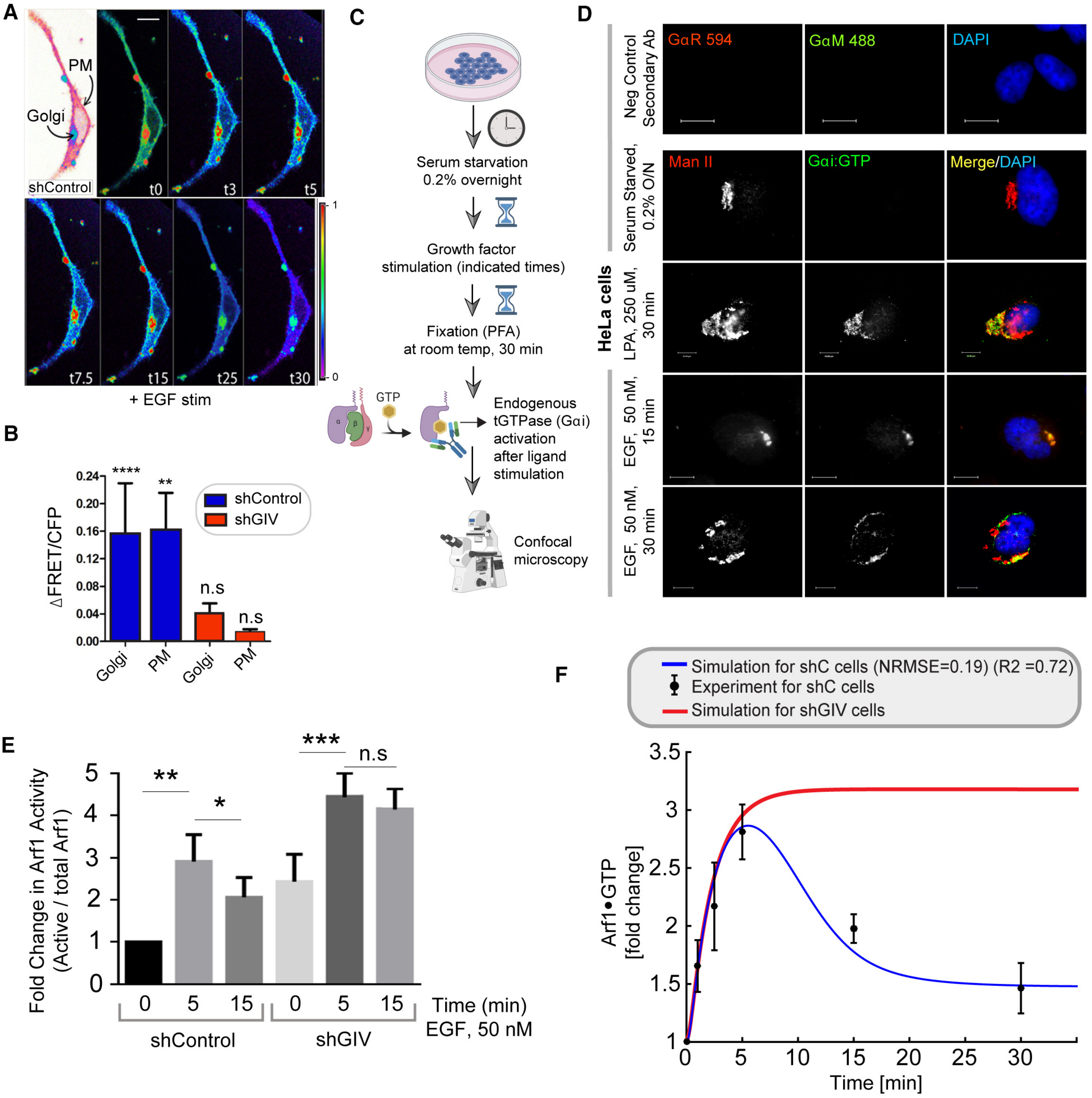

A
PM
Golgi
shControl
t0
t3
t5
t7.5
t15
t25
t30
1
0
+ EGF stim
B
ΔFRET/CFP
0.24
0.20
0.16
0.12
0.08
0.04
0.00
****
**
n.s
n.s
shControl
shGIV
Golgi
PM
Golgi
PM
C
Serum starvation
0.2% overnight
Growth factor
stimulation (indicated times)
Fixation (PFA)
at room temp, 30 min
GTP
Endogenous
tGTPase (Gαi)
activation
after ligand
stimulation
Confocal
microscopy
D
Neg Control
Secondary Ab
Serum Starved,
0.2% O/N
HeLa cells
LPA, 250 uM,
30 min
EGF, 50 nM,
15 min
EGF, 50 nM,
30 min
GαR 594
GαM 488
DAPI
Man II
Gαi:GTP
Merge/DAPI
E
Fold Change in Arf1 Activity
(Active / total Arf1)
**
*
***
n.s
0
5
15
0
5
15
Time (min)
shControl
shGIV
EGF, 50 nM
F
Simulation for shC cells (NRMSE=0.19) (R2 =0.72)
Experiment for shC cells
Simulation for shGIV cells
Arf1•GTP
[fold change]
3.5
3
2.5
2
1.5
1
Time [min]
0
5
10
15
20
25
30

Figure EV2.

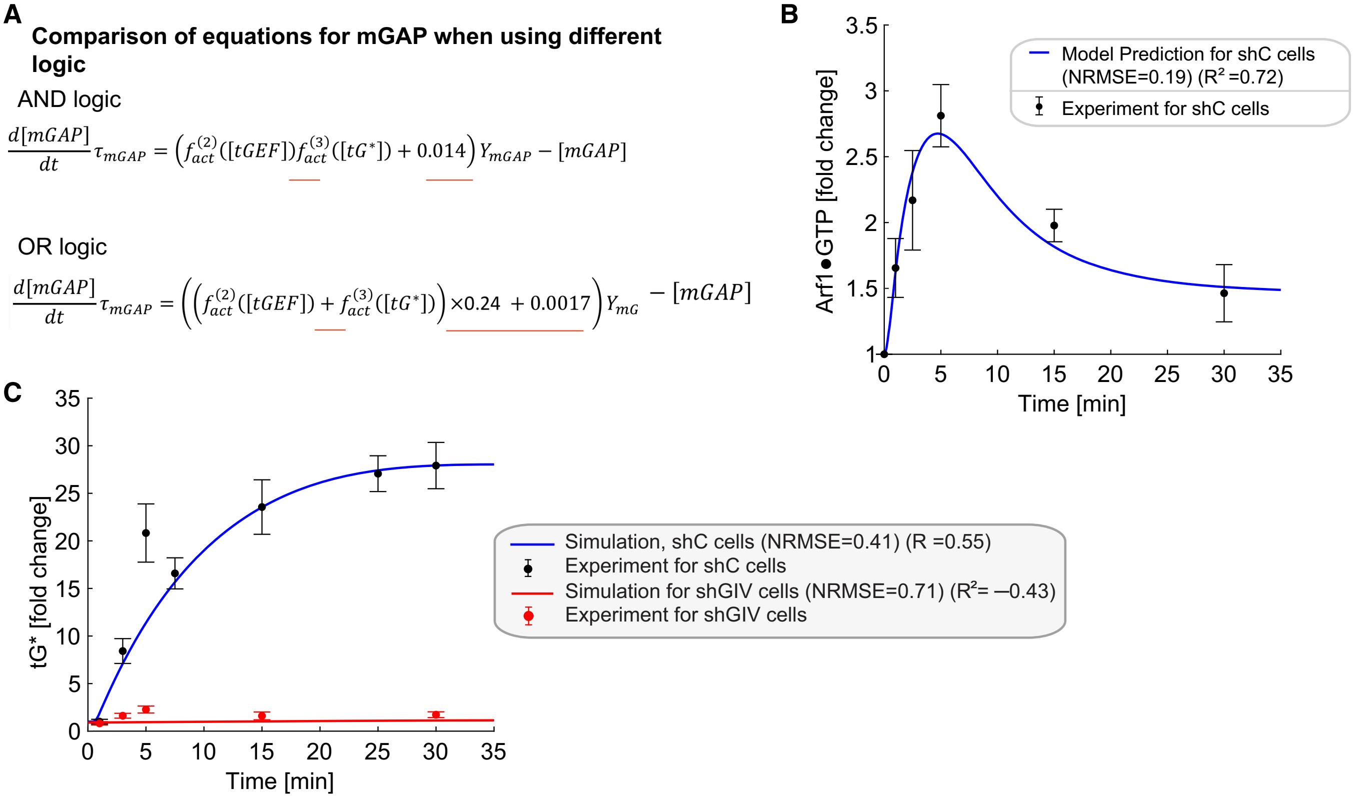

Figure EV3. Simulations of Arf1 activation dynamics (mG*) and Gi activation dynamics (tG*) when using OR logic.

A Comparisons of equations for mGAP when using different logics. The AND and OR logic are modeled by $f_{act}^{(2)}([tGEF])f_{act}^{(3)}([tG^*])$ and $f_{act}^{(2)}([tGEF]) + f_{act}^{(3)}([tG^*])$, respectively. The constants 0.24 and 0.0017 ensure that the steady-state values of all species when using OR logic are the same as those for AND logic. The differences between the two models are underlined in red in the equations.

B, C Simulations of Arf1 (B) and Gi (C) activation dynamics for OR logic. The OR logic also captures the experimental data reasonably well but the AND logic is better informed experimentally. The experimental data are the same as those in Fig 3J and K.

 © 2023 The Authors

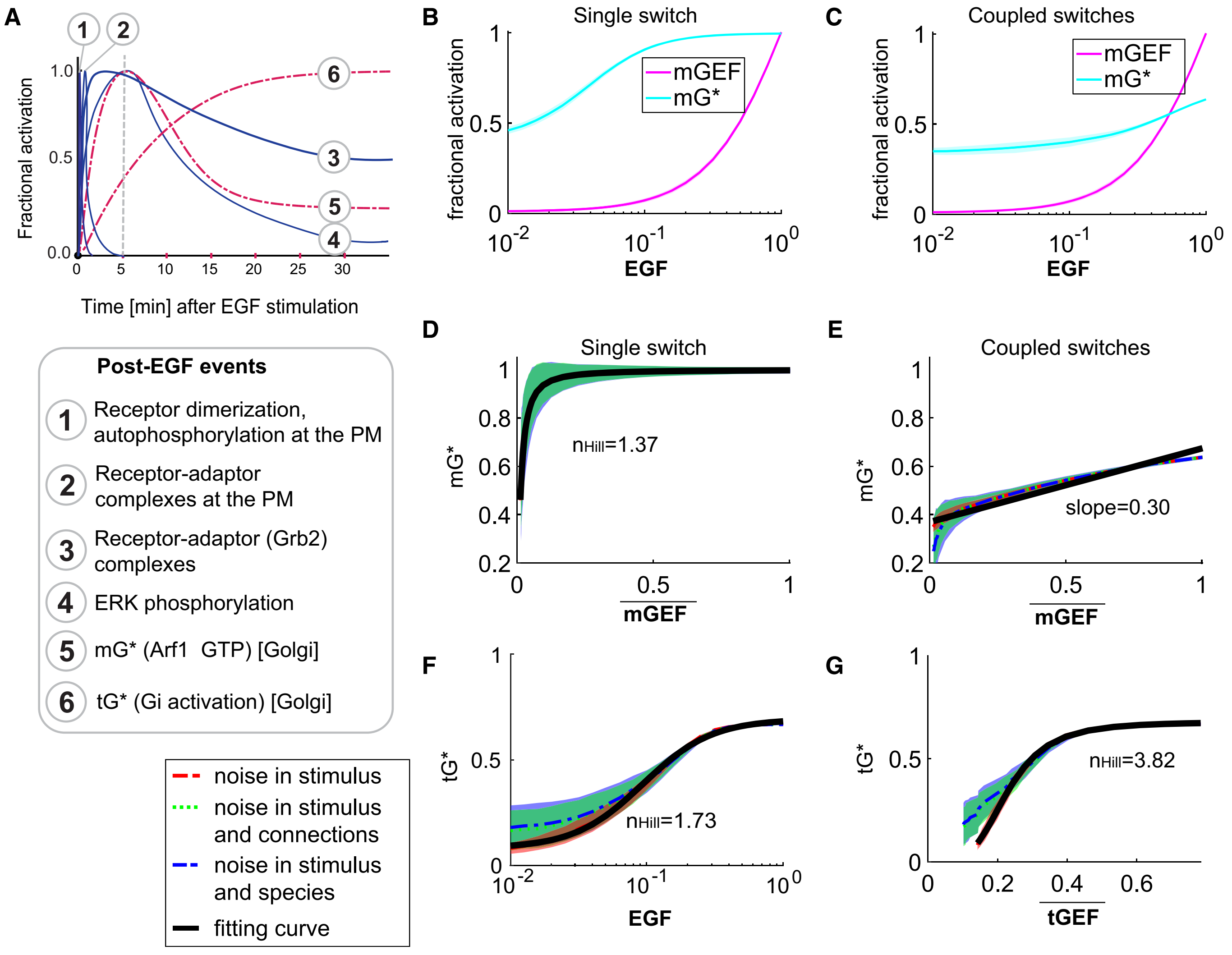

Figure EV4. Coupled switches enable the alignment of endomembrane responses (Arf1 and tG* activities) to the dose of an extracellular stimulus.

A Published dynamics of EGF-stimulated events that are initiated at the PM (blue, continuous) or experimentally determined dynamics of events at the Golgi confirmed here (red, interrupted) are compared. The interrupted line at 5 min provides a reference frame for the observed peak Arf1 activity upon EGF stimulation.

B, C Dose responses of fractional activations of mGEF and active Arf1 (mG*) for the single switch (B; mG alone) and coupled switches (C; mG and tG). We perform stochastic simulations in the presence of noise in EGF (see Materials and Methods for details). The mean and the standard deviation (SD) of species are evaluated at steady states. The dimensionless EGF concentrations in the simulations are obtained through normalization, that is, dividing the EGF concentration by 217.4 nM (=50 nM/0.23). In all simulations, noise is introduced only in stimulus (i.e., EGF).

D, E The same plots as in Fig 4A and B but in the presence of three different types of noise: noise in stimulus (shown in red), noise in stimulus and connections simultaneously (shown in green), and noise in stimulus and species simultaneously (shown in blue; see Materials and Methods for details). Data are shown as mean values (dashed curves), with the shading showing the SD. The black curves are fitting curves ($r^2 > 0.95$) for red dashed curves (see Materials and Methods for the calculations of r^2 and n_{Hill}).

F, G The fractional activations of tGTPase (tG*) as a function of EGF (F) or tGEF (G). The plots are generated in a similar way as in (F, G). $\overline{tGEF}$ denotes the mean of tGEF. $r^2 > 0.95$ for all fitting curves. The EGF-tG* relationship shows a Hill coefficient of 1.73, and the tGEF→tG* switch (switch #2) shows a dose–response behavior close to the saturation regime of an ultrasensitive curve ($n_{Hill} = 3.82$).

Figure EV5. GIV-GEM is required for EGF-triggered secretion of diverse cargo proteins through the Golgi compartment.

A Schematic shows the basis for measuring secretion of transmembrane cargo protein, ts-VSVG-eGFP. This temperature-sensitive mutant VSVG is retained in the ER at 40°C, at the Golgi at 20°C, and moves out of the Golgi to the PM when shifted to 32°C (Gallione & Rose, 1985). When visualized with immunofluorescence under non-permeabilized conditions, a VSVG-ectodomain targeting antibody selectively detects PM-localized cargo, whereas a GFP tag allows the visualization of total VSVG in the cell.

B, C Control (sh Control; top) and GIV-depleted (shGIV; bottom) Cos7 cells were transfected with tsO45-VSVG-GFP and cells were shifted to 40°C for O/N and then incubated at 20°C for 1 h in HEPES buffered serum-free media followed by temperature shift at 32°C for 15 min in plain DMEM and or containing 50 nM EGF or 10% serum. Coverslips were fixed and stained with VSVG-ectodomain-specific monoclonal antibody (red). Representative images are shown in (B). Scale bar = 10 μm. Green fluorescence indicates total VSVG expression whereas red fluorescence shows the surface-localized pool of VSVG. Bar graphs in (C) display the Red:Green intensity ratio indicative of the fraction VSVG that is secreted to the cell surface. Results are expressed as mean ± SEM; $n = 3$ biological replicates; P-values were determined using Mann–Whitney t-test compared to t0: $^{*}P < 0.05$.

D–H Control (sh Control) and GIV-depleted (shGIV) HeLa cells were analyzed for EGF-stimulated secretion of three soluble cargo proteins, MMP2 (D, E), MMP9 (D, F), and Collagen-Vii RFP (G, H), as detected from the supernatants at indicated time points after EGF stimulation. Results are expressed as mean ± SEM; $n = 3$ replicates; P-values were determined using Mann–Whitney t-test compared to t0: $^{**}P < 0.01$; $^{***}P < 0.001$. Immunoblots are representative of findings from at least three independent repeats.

 © 2023 The Authors

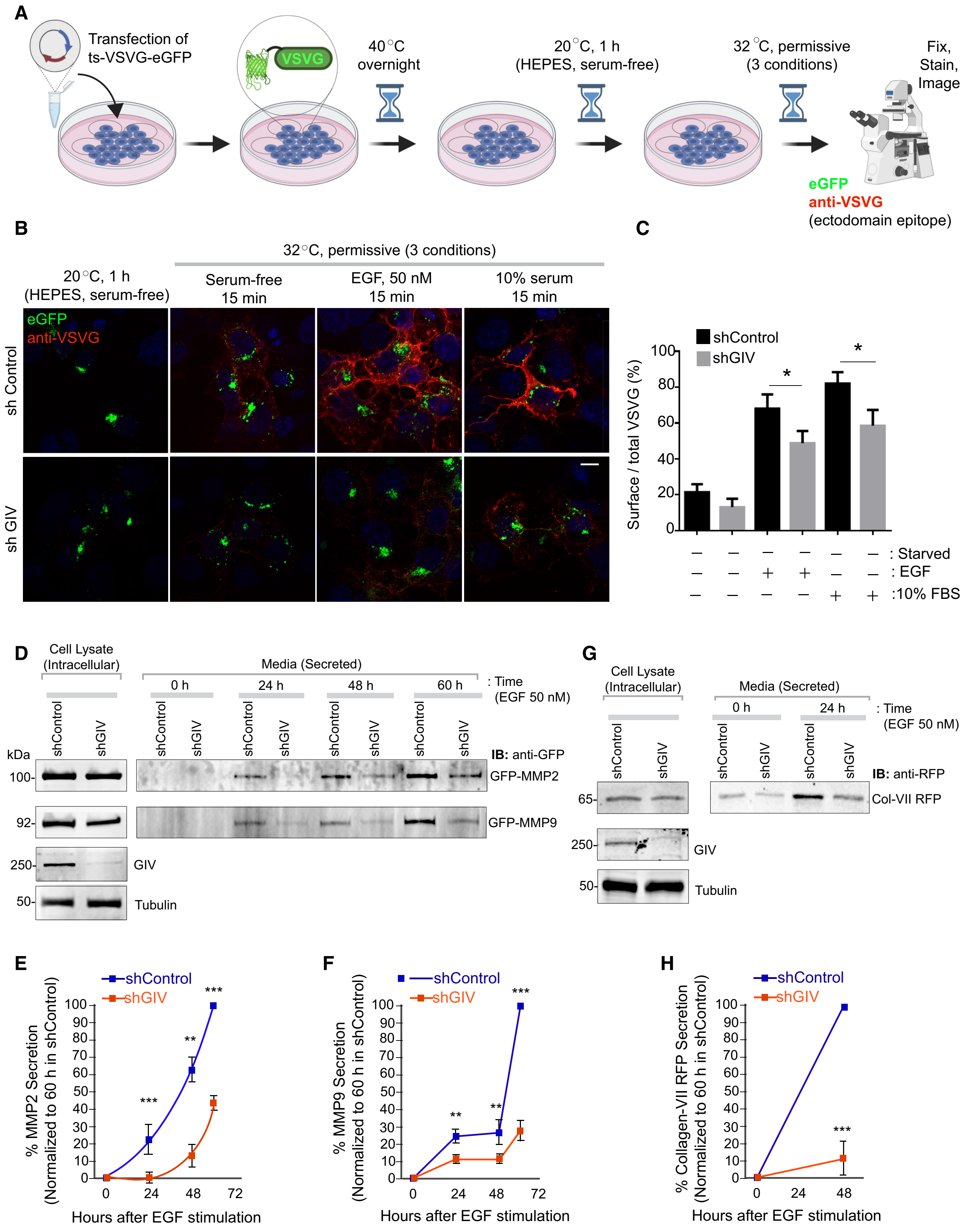
A
Transfection of ts-VSVG-eGFP
VSVG
40°C overnight
20°C, 1 h (HEPES, serum-free)
32°C, permissive (3 conditions)
Fix, Stain, Image
eGFP
anti-VSVG
(ectodomain epitope)
B
32°C, permissive (3 conditions)
20°C, 1 h (HEPES, serum-free)
Serum-free 15 min
EGF, 50 nM 15 min
10% serum 15 min
eGFP
anti-VSVG
sh Control
sh GIV
C
shControl
shGIV
Surface / total VSVG (%)
: Starved
: EGF
:10% FBS
D
Cell Lysate (Intracellular)
Media (Secreted)
0 h
24 h
48 h
60 h
: Time (EGF 50 nM)
kDa
IB: anti-GFP
GFP-MMP2
GFP-MMP9
GIV
Tubulin
G
Cell Lysate (Intracellular)
Media (Secreted)
IB: anti-RFP
Col-VII RFP
E
% MMP2 Secretion (Normalized to 60 h in shControl)
Hours after EGF stimulation
F
% MMP9 Secretion (Normalized to 60 h in shControl)
Hours after EGF stimulation
H
% Collagen-VII RFP Secretion (Normalized to 60 h in shControl)
Hours after EGF stimulation

Figure EV5.
