# Supplementary figures and images for: A circuit for secretion‐coupled cellular autonomy in multicellular eukaryotic cells
Source: Mol Syst Biol. 2023 Mar 1;19(4):e11127. doi: 10.15252/msb.202211127 (PMC10090951; doi:10.15252/msb.202211127)

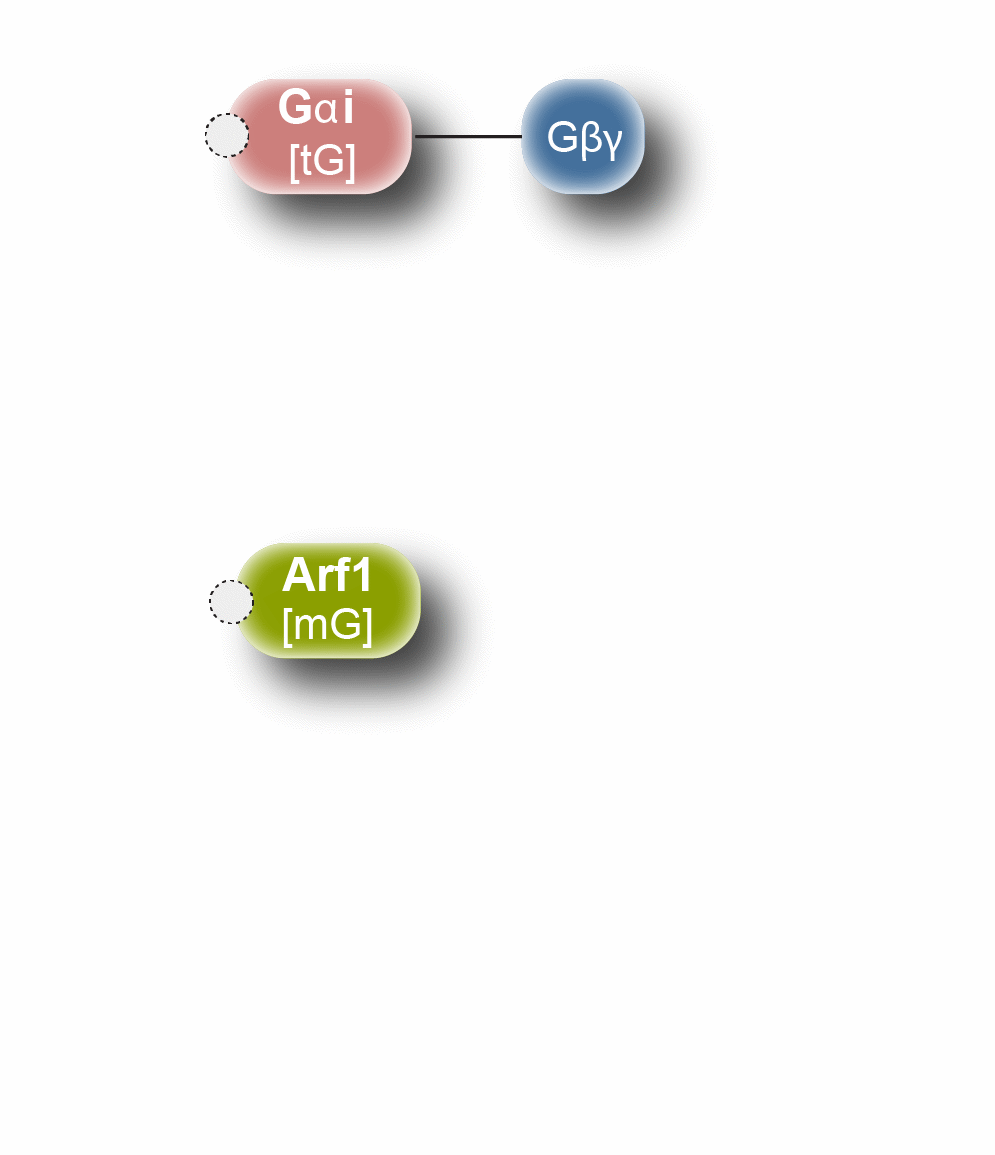

Supplement: Supplementary file 4 — Movie EV1 [file MSB-19-e11127-s002.zip › Movie EV1/Moive EV1.gif]

## Slide 1
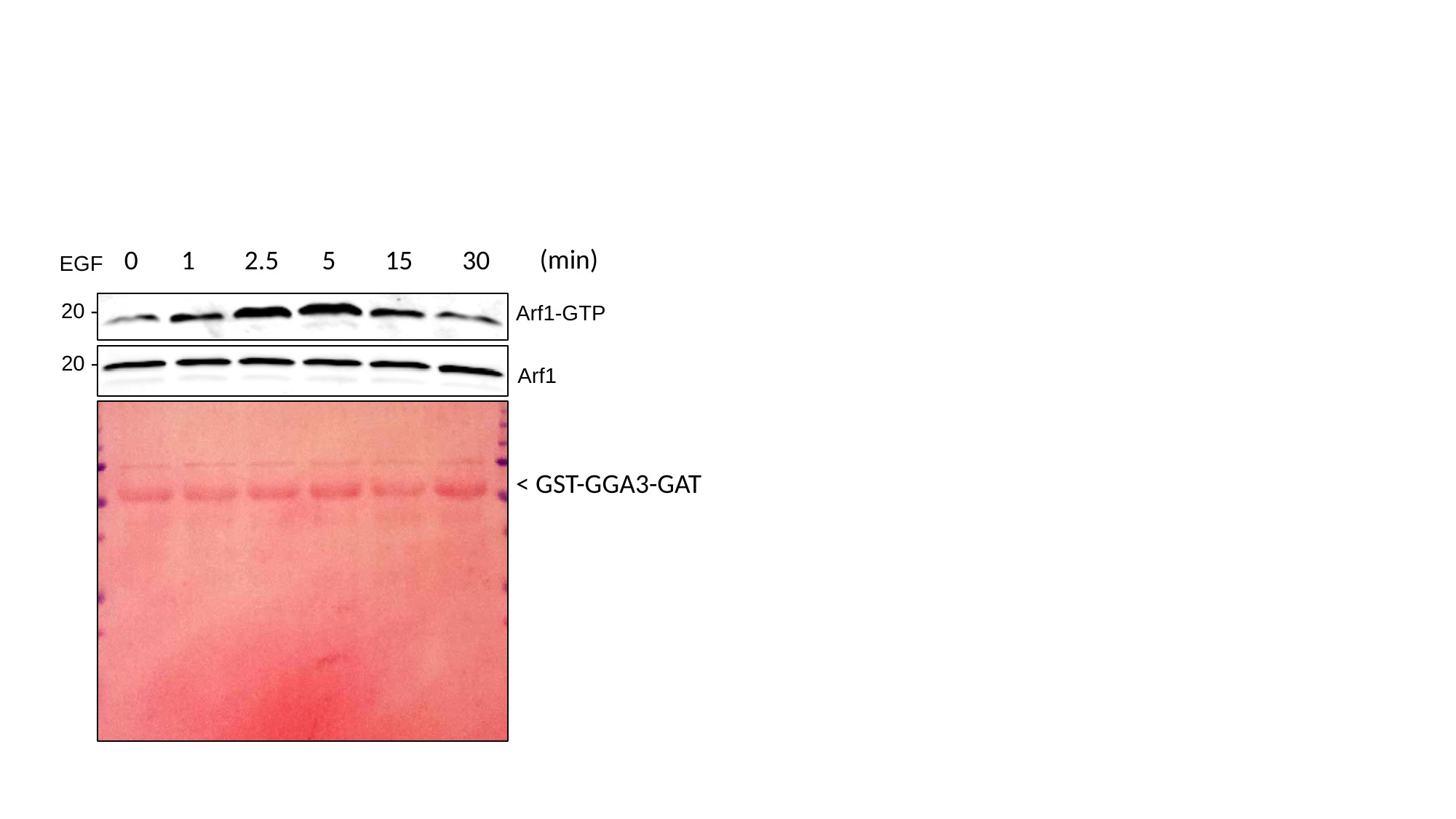

(min)
0 1 2.5 5 15 30
EGF
20 -
Arf1-GTP
20 -
Arf1
< GST-GGA3-GAT

Supplement: Supplementary file 8 — Source Data for Figure 2 [file MSB-19-e11127-s001.zip › Figure 2/2B/Immunoblot.pptx]

## Slide 1
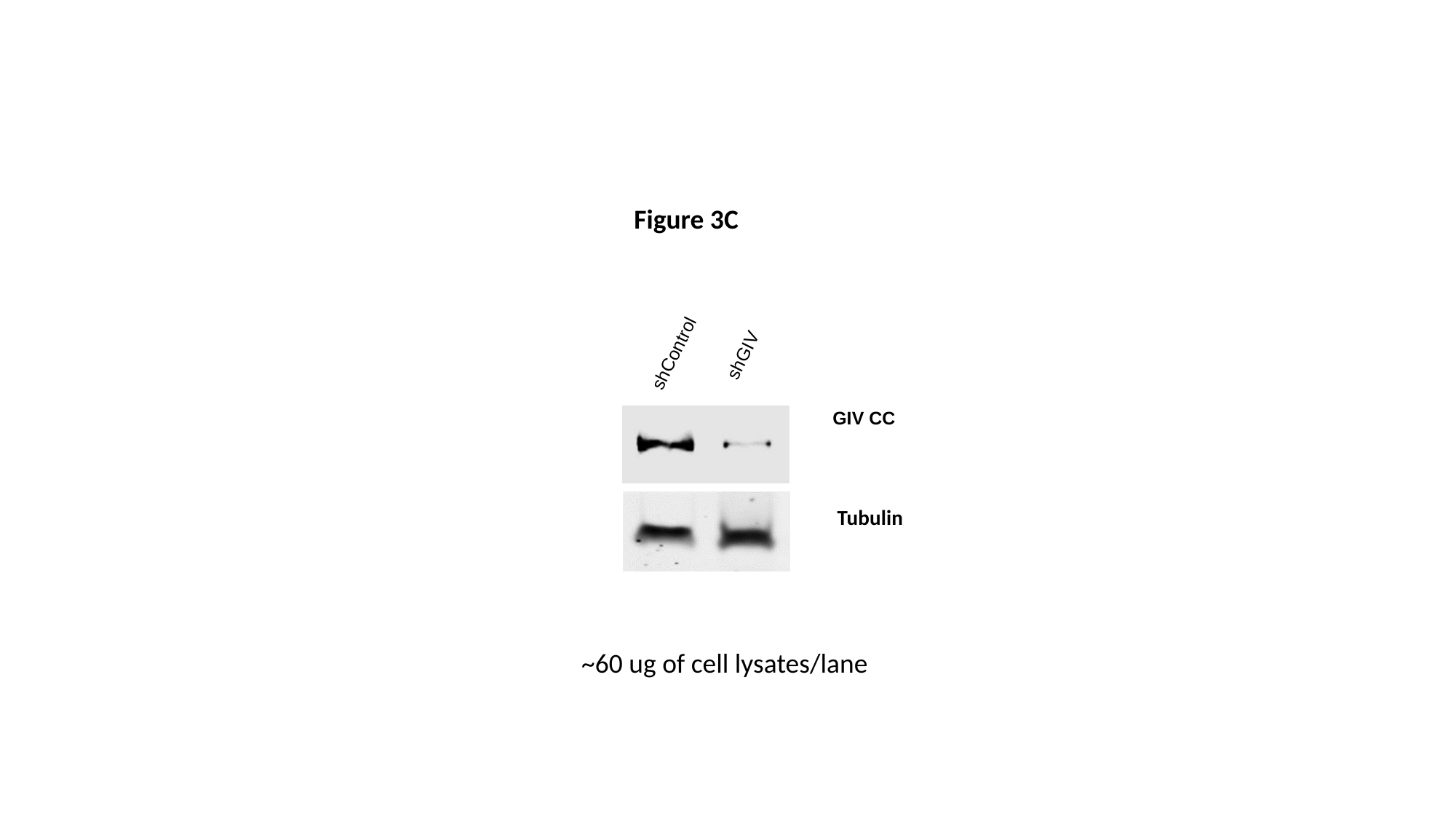

Figure 3C
shGIV
shControl
GIV CC
Tubulin
~60 ug of cell lysates/lane

Supplement: Supplementary file 9 — Source Data for Figure 3 [file MSB-19-e11127-s008.zip › Figure 3/3C/Immunoblot.pptx]

## Slide 1
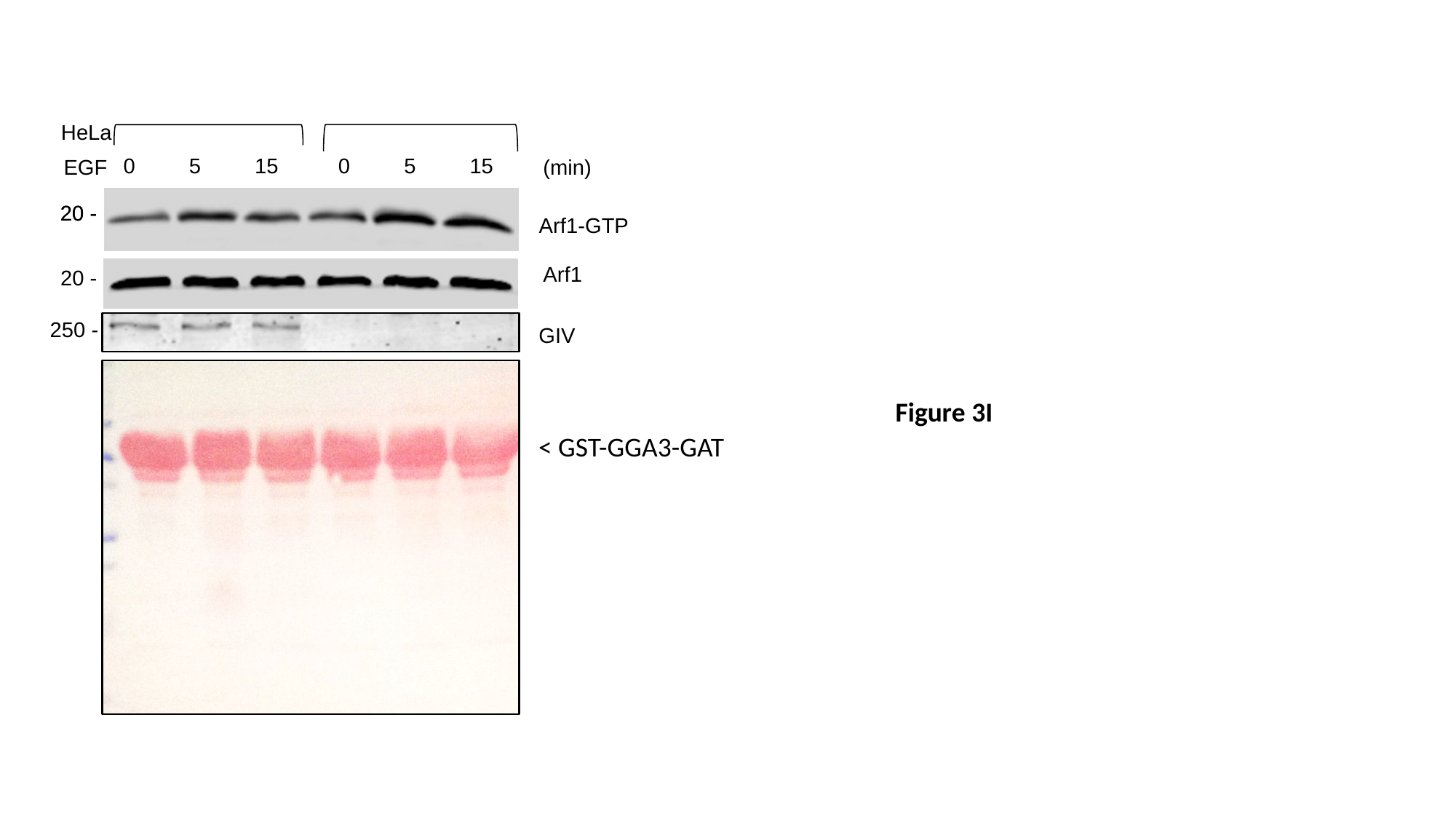

HeLa
0 5 15 0 5 15
EGF
(min)
20 -
20 -
Arf1-GTP
Arf1
20 -
250 -
GIV
Figure 3I
< GST-GGA3-GAT

Supplement: Supplementary file 9 — Source Data for Figure 3 [file MSB-19-e11127-s008.zip › Figure 3/3I/Immunoblot.pptx]

## Slide 1
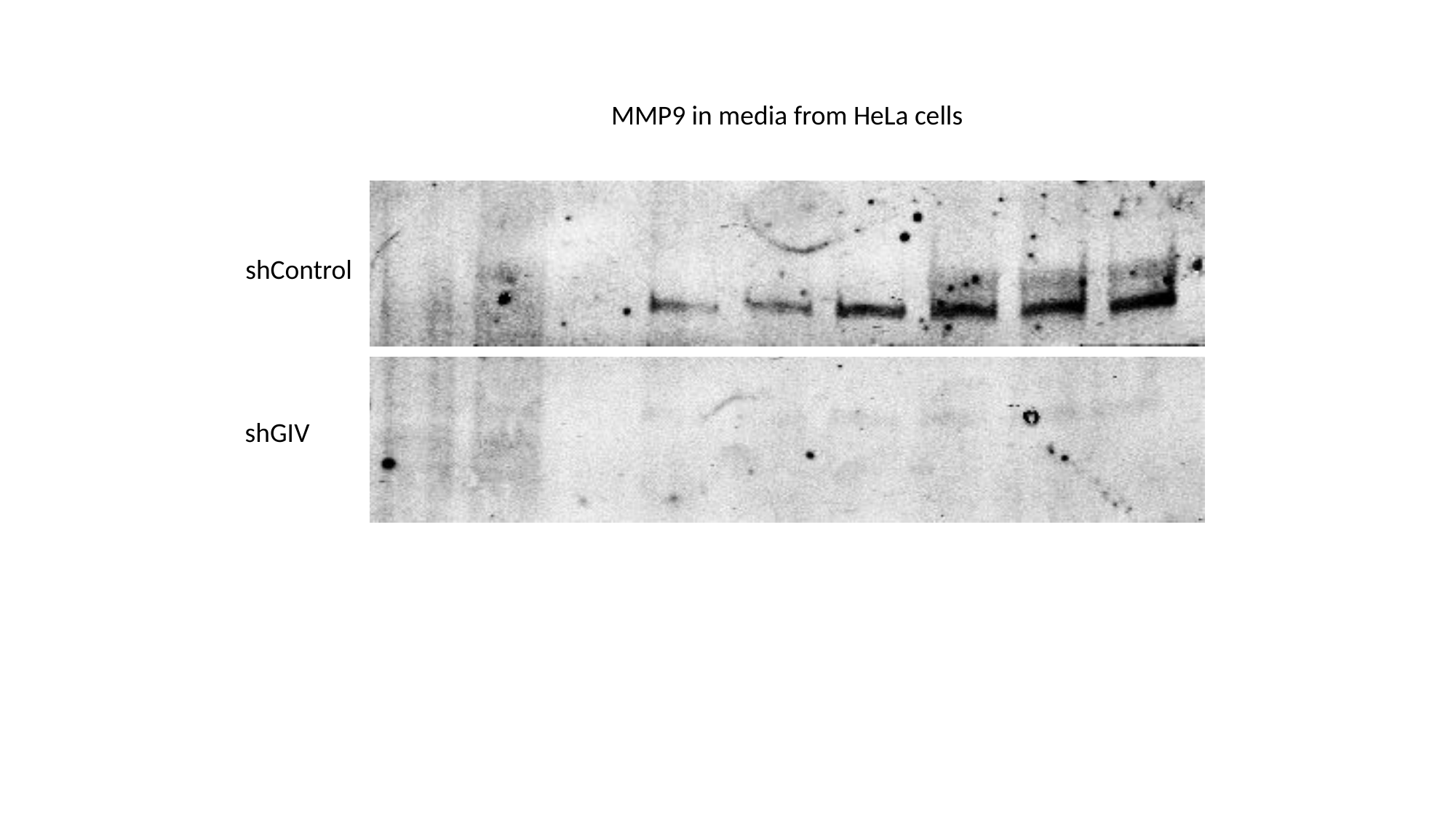

MMP9 in media from HeLa cells
shControl
shGIV

## Slide 2
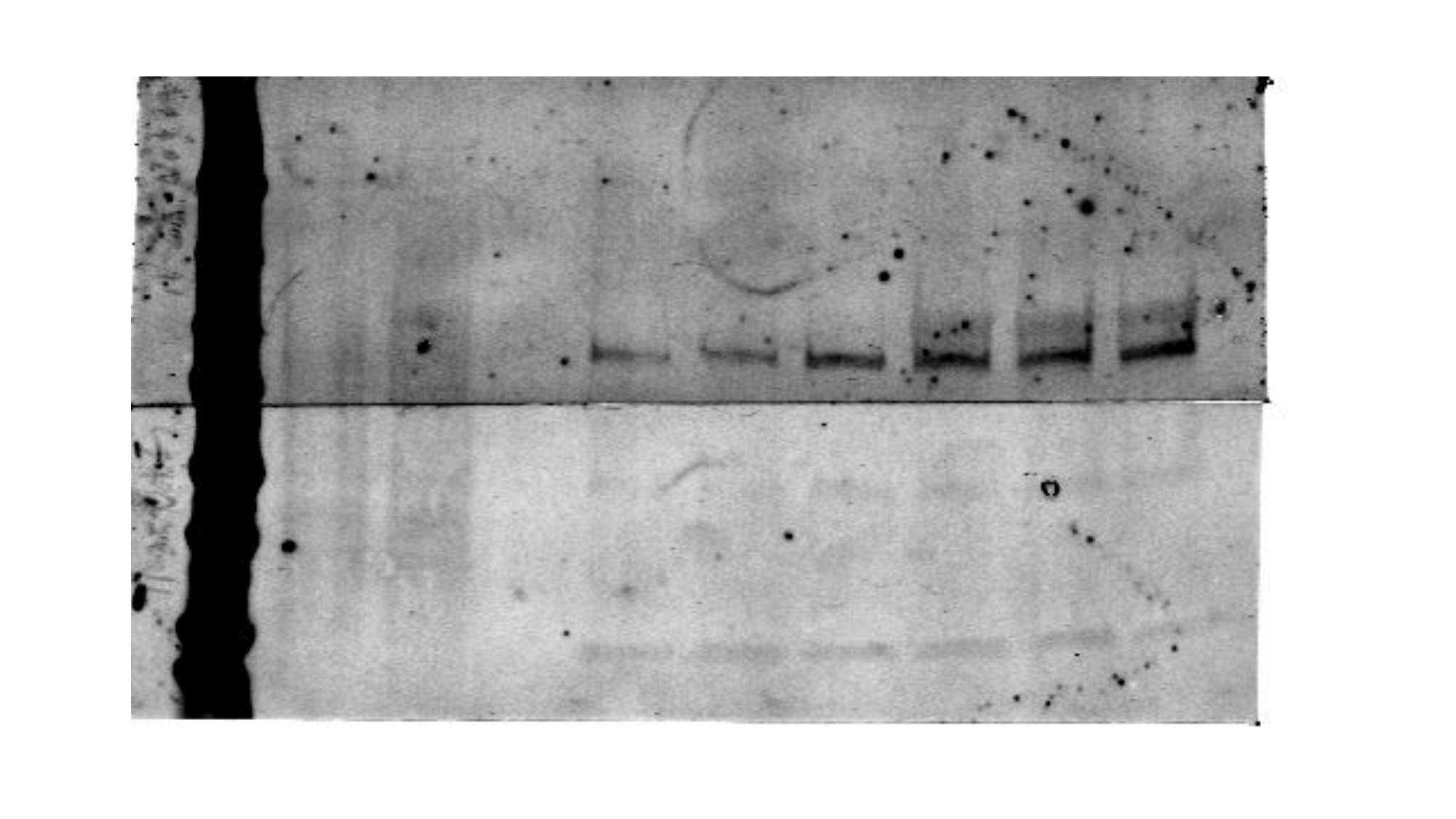

## Slide 3
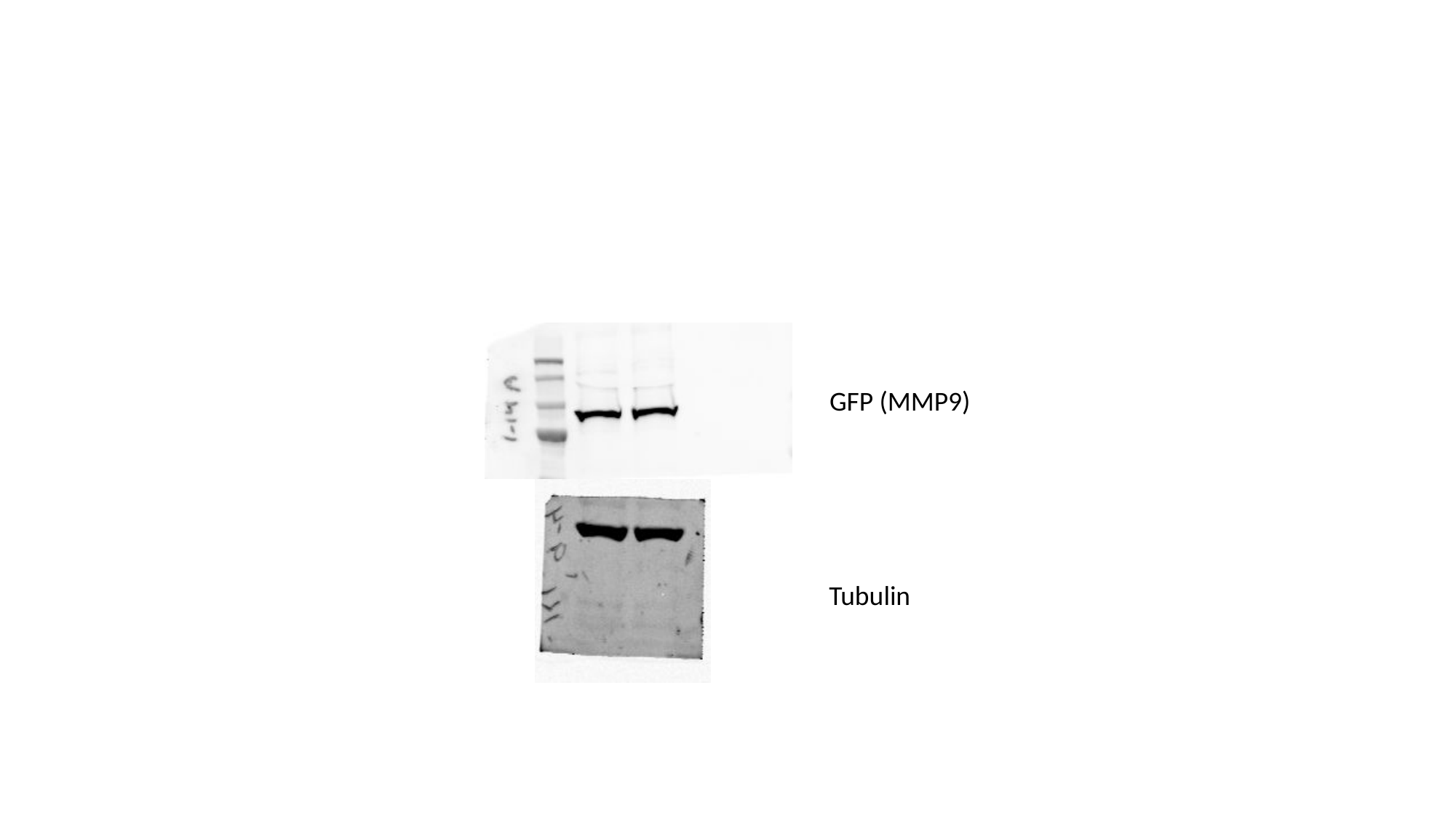

GFP (MMP9)
Tubulin

Supplement: Supplementary file 10 — Source Data for Figure 6 [file MSB-19-e11127-s007.zip › Figure 6/6B/Immunoblot2.pptx]

## Slide 1
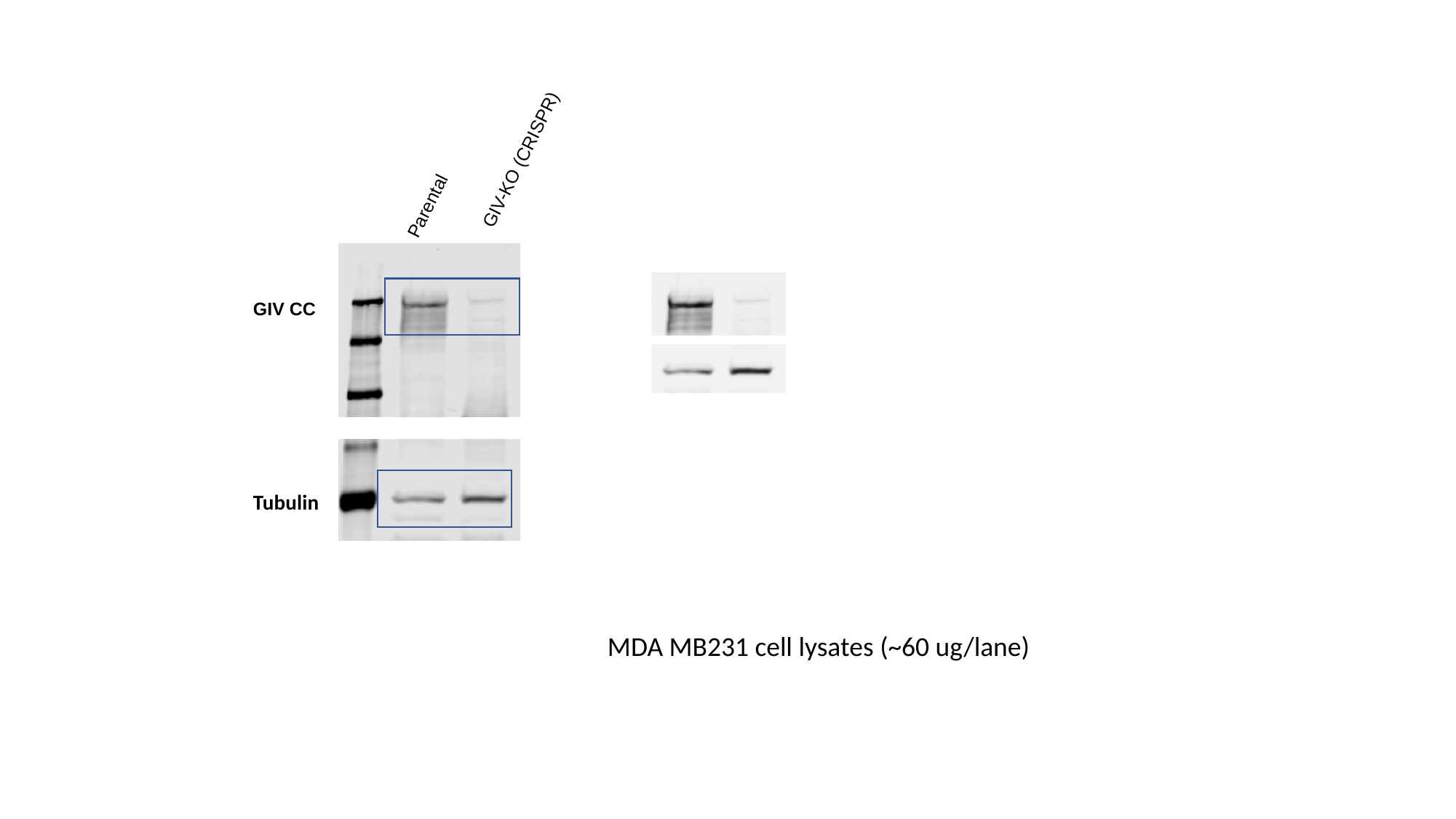

GIV-KO (CRISPR)
Parental
GIV CC
Tubulin
MDA MB231 cell lysates (~60 ug/lane)

Supplement: Supplementary file 10 — Source Data for Figure 6 [file MSB-19-e11127-s007.zip › Figure 6/6K/Immunoblot.pptx]
