# Supplementary material for: A circuit for secretion‐coupled cellular autonomy in multicellular eukaryotic cells
Source: Mol Syst Biol. 2023 Mar 1;19(4):e11127. doi: 10.15252/msb.202211127 (PMC10090951; doi:10.15252/msb.202211127)
Supplement: Supplementary file 10 — Source Data for Figure 6 [file MSB-19-e11127-s007.zip › Figure 6/6B/Immunoblot.pptx]

## Slide 1
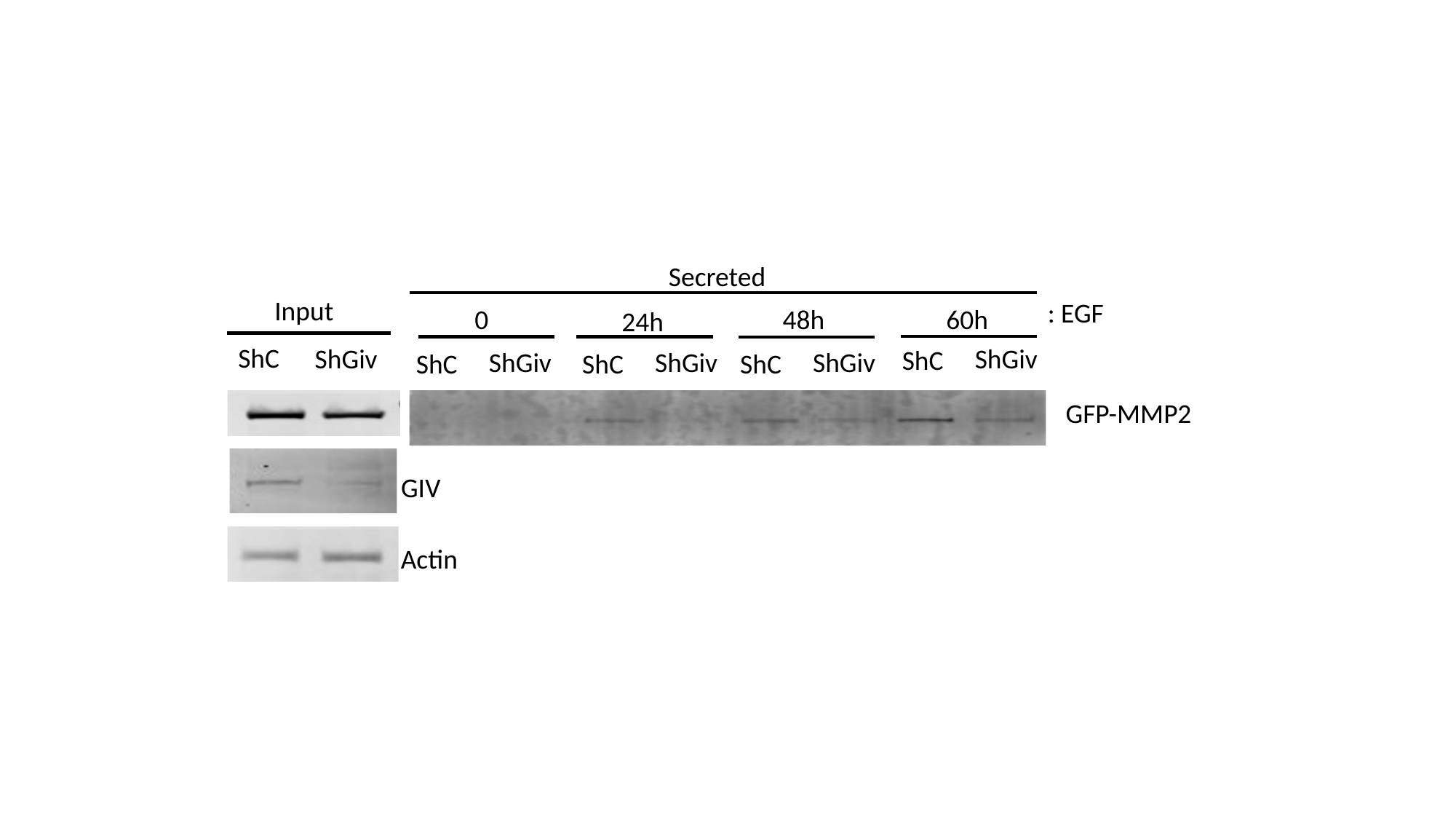

Secreted
Input
: EGF
0
48h
60h
24h
ShC
ShGiv
ShGiv
ShC
ShGiv
ShGiv
ShGiv
ShC
ShC
ShC
GFP-MMP2
GIV
Actin

## Slide 2
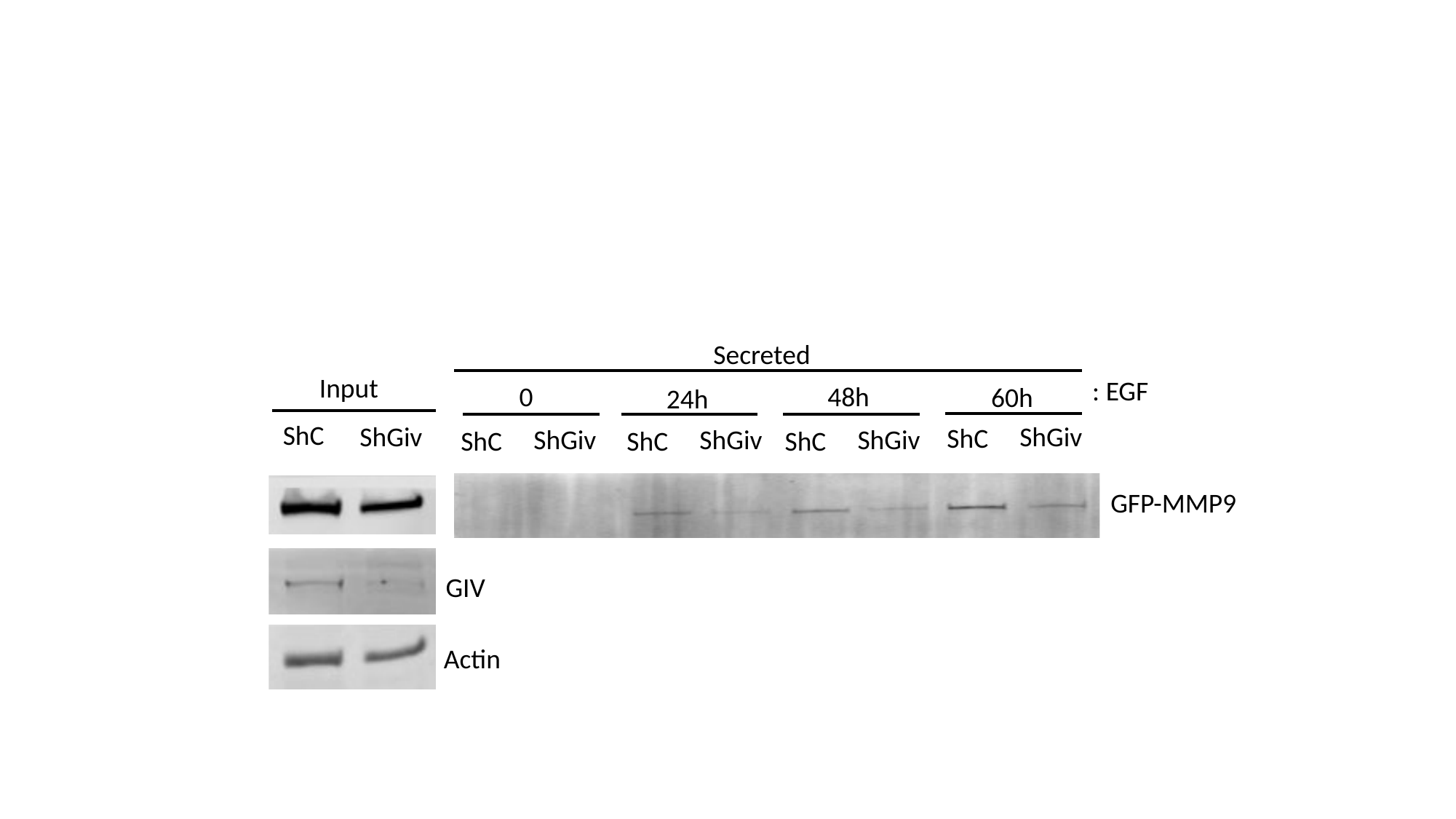

Secreted
Input
: EGF
0
48h
60h
24h
ShC
ShGiv
ShGiv
ShC
ShGiv
ShGiv
ShGiv
ShC
ShC
ShC
GFP-MMP9
GIV
Actin
