# Supplementary material for: A circuit for secretion‐coupled cellular autonomy in multicellular eukaryotic cells
Source: Mol Syst Biol. 2023 Mar 1;19(4):e11127. doi: 10.15252/msb.202211127 (PMC10090951; doi:10.15252/msb.202211127)
Supplement: Supplementary file 11 [file MSB-19-e11127-s009.pdf]

# A Circuit for Secretion-coupled Cellular Autonomy in Multicellular Eukaryotic cells

Lingxia Qiao, Saptarshi Sinha, Amer Abd El-Hafeez, I-Chung Lo, Krishna Midde, Tony Ngo, Nicolas Aznar, Inmaculada Lopez-Sanchez, Vijay Gupta, Marilyn Gist Farquhar, Padmini Rangamani, and Pradipta Ghosh
**DOI: 10.15252/msb.202211127**

*Corresponding author(s): Pradipta Ghosh (prghosh@ucsd.edu) , Padmini Rangamani (padmini.rangamani@eng.ucsd.edu)*



## Review Timeline:

| | |
|---|---|
| Submission Date: | 18th May 22 |
| Editorial Decision: | 5th Jul 22 |
| Revision Received: | 22nd Dec 22 |
| Editorial Decision: | 2nd Feb 23 |
| Revision Received: | 6th Feb 23 |
| Accepted: | 7th Feb 23 |

*Editor: Jingyi Hou*


## Transaction Report:

(Note: With the exception of the correction of typographical or spelling errors that could be a source of ambiguity, letters and reports are not edited. Depending on transfer agreements, referee reports obtained elsewhere may or may not be included in this compilation. Referee reports are anonymous unless the Referee chooses to sign their reports.)

## 1st Editorial Decision — 5th Jul 2022

Thank you for submitting your work to Molecular Systems Biology. We have now heard back from two of the three reviewers who agreed to evaluate your manuscript. In the interest of time, and since the recommendations of the two reviewers are quite similar, I prefer to make a decision now rather than further delay the process. If we receive comments from Reviewer #3, we will forward them to you so that you can address any further issues raised. As you will see from the reports below, the reviewers acknowledge the interest of the study and appreciate the amount of data generated. They raise, however, a series of concerns, which we would ask you to address in a major revision.

The reviewers' recommendations are relatively clear, so there is no need to reiterate the points listed below. In particular, both reviewers mentioned that the flow of the manuscript must be substantially revised, and additional clarifications and details need to be provided. Considering these comments, we would ask you to improve the presentation of the study to make the data and the main conclusions easily accessible to the readers. All other issues raised by the reviewers need to be satisfactorily addressed as well. As you may already know, our editorial policy allows in principle a single round of major revision, and it is therefore essential to provide responses to the reviewers' comments that are as complete as possible.

On a more editorial level, we would ask you to address the following issues:

--------------------------------------------------------

Reviewer #1:

In this paper, Qiao, El-Hafeez, Lo and colleagues investigate, using a combined computational and experimental approach, how the coupling between two GTPases (the monomeric GTPase Arf1 and the heterotrimeric GTPase Gi ) imparts to the cells the ability to secrete growth factors and respond to them proportionally, creating a so-called 'secrete-and-sense' loop that explains how, for instance, cancer cells can sustain themselves even in the absence of external growth factors.
The authors build a mathematical model made of two parts: one that models the circuit, localized at the Golgi, that senses the growth factor; and one that models protein secretion and cell growth and proliferation, which are a consequence of the sensing part. The three processes (sensing, secretion and proliferation) happen on different time scales; thus, the models are distinct. The authors show how the guanine nucleotide exchange factor GIV/Girdin functions as a bridge between Arf1 and Gi first, Arf1 is activated by the growth factor EGF (meaning it gets into the GTP-bound form); the consequence of Arf1 activation is the recruitment of GIV/Girdin on Golgi membranes. Once recruited there, GIV/Girdin activates Gi by triggering the dissociation of G i1 from G 1 2. There are then two negative feedback loops that takes place: (1) GIV/Girdin binds to and recruits to the Golgi membranes ArfGAP2/3, which being a GTPase activating factor (GAF) for Arf1, makes Arf1 go back to the GDP-bound state, which is the inactive state; (2) free G 1 2 is a co-factor for ArfGAP2/3, therefore, it also contributes to terminating the signaling cascade. The authors convincingly show that GIV/Girdin is necessary for the coupling of the two GTPases, for the dose-dependent secretion of various cargos, which, in turn, is necessary for the survival of the cells when they rely on autocrine secretion. Thus, taken together, this paper gives strong evidence of how cells can sense and respond to growth factors secreted by them and provides interesting ideas on how one could interfere with this process to, for instance, counteract cancer.

I was positively impressed by the amount of data, and by the modeling, which also entails a bioinformatics aspect (the authors generated Arf1's interactome networks with and without GIV/Girdin to predict what the role of this protein).
I actually do not have suggestions for additional experiments! I rather have suggestions to improve the flow of the paper and the figures.

1. I think the introduction lacks important parts, such as what are GTPases, GAPs, GEFs, what is Arf1, Gi, ArfGAP2/3, etc. This information is either entirely absent or scattered in the results. I believe it would help the reader to find it all in one place, the introduction.
2. The first part of the results belongs, in my opinion, to the end of the introduction. Typically, the introduction ends with some paragraphs describing what was done in the paper. In the results, I would go directly into the description of the experiments, and avoid statements on the approach etc.
3. "unlikely": this adjective sounds misplaced here. Unlikely means improbable, implausible. I would keep only "distinct".
4. The authors write: "using the general framework presented in 23,24": I would find it useful to the readers if the framework were at least named and briefly explained.
5. ERGIC: please define
6. Figure 2A: I honestly do not see how this panel represents the "coupling"...
7. GST-GAT domain: is GST the tag (Glutathione S Transferase)? If so, why didn't the authors use an anti-GST antibody? Why showing the Ponceau staining?
8. Figure 1C: I do not see the need for showing such a scheme. I think readers can understand the concept of a pull-down without seeing it depicted.
9. Excellent fitness: I think that R2 of 0.54 does not represent excellent, but rather a good fit between model and data...
10. In the paper, the word "stimuli" is often used when the singular form, stimulus, should be used instead. I suggest the authors to check this.
11. Man II: what is this?
12. "These findings simulate": findings to not simulate. I would rephrase: "the model predictions reproduced what was experimentally demonstrated before18"
13. At the end of page 8, in the sentence starting with "; because the first input", a verb seems to be missing.
14. When stating that negative feedback reduces noise, I would suggest the authors to cite also this experimental paper: Becksei, Serrano: Engineering stability in gene networks by autoregulation. Nature volume 405, pages 590-593 (2000)
15. DoRA: please define
16. "We observed 3 things„: I would rephrase: "we made three observations"
17. The authors write existing theories linking genetic circuits to cellular autonomy exist (and cite several papers), but state they do not apply to the eukaryotic cell. I saw that reference 63 is a paper in which the eukaryote Dictystilium was used. So why this statement?
18. GEM module: GEM domain?
19. "Our model chose to test": a model does not choose. Re-phrase "we chose to model..."
20. In the legend to Figure 1B: GIV-GEM is mentioned. This is not mentioned anywhere else in the next. In the text, it is always GIV/Girdin. Please explain.
21. I do not think the scheme in Fig. 3G is needed
22. Figure 3I: what do low and high indicate?
23. Figure 6C: is this really linear?

Reviewer #2:

Summary:
Previous work identified the molecular parts that cancer cells use to secrete and sense growth factors. But we currently do not understand how these parts combine to supposedly allow a cancer cell to autonomously grow. Specifically, for a cancer cell, it's unclear how a sensing of its own growth factor controls the secretion of that growth factor. Answering this question inherently requires a quantitative and systems approach. The authors use such an integrated approach to try to address this fundamental question in cancer biology.

To answer this question, the authors begin with a model that spans multiple scales both spatially and temporally (Fig. 1). The authors investigate the relatively fast, intracellular processing of growth factors in the ER and golgi appartus and the relatively slow, extracellular secretion and sensing of these growth factors. The mathematical model describing these processes are simple and phenomenological equations that are experimentally constrained, with the constraints coming from the authors' own measurements in HeLa cells, breast cancer cells (MDA-MB231), and in vitro experiments. These experiments focused on two GTPases at the golgi, "Arf1" and "Gi", and how they co-regulate the production and eventual secretion of the growth factors. The main finding from studying Arf1 and Gi is that the GTP hydrolysis rate, which is regulated by mGAP, primarily sets the secretion rate of growth factors.

The main contribution of this work is systematically tying together various intracellular parts that control how a cell senses and secretes EGF. It's impressive how the authors used various techniques to assemble the various parts together. But this assembly process is not clearly explained. So I think it's difficult to see the forest from the trees. I think a rewriting of the manuscript will highlight the main, systems-level conclusions without having the reader getting lost in all the details.

In fact, I found this paper to be very difficult to understand. For one, it was challenging to understand many of the experiments because the authors did not fully explain the experimental set up and the goal of each experiment. The manuscript also assumes that the reader have a sufficient background knowledge of Arf1, GTPases, golgi apparatus, and the importance of all these to cancer cells.

Overall recommendation:
This study contains many and technically impressive experiments. The authors do an admirable job of trying to combine these different experiments, spanning vast time scales (hours to days) and spatial scales (organelle to multiple cells). But I think the process of combining the different results isn't explained well or is missing some key experiments/interpretations. Thus, I recommend a major revision that mainly focuses on two aspects:

1. Redoing some of the key experiments in Figs. 2-3 with various concentrations of EGF as opposed to the current presentation which used just one or a few concentrations of EGF.

2. Vastly rewriting the manuscript by:
- giving a more full explanations of the experimental details
- excluding some figures and results (e.g., PPI analysis in Fig. 5 and large parts of Fig. 7)
- giving a roadmap along the way that says which parameters are being fitted in the experiments in Figs. 2-3 and how these parameter values that you get here will be used in the model in Fig. 6 (i.e., which parameters in which equations in Fig. 6 are being measured in the experiments of Figs. 2-3?)
- a more extensive Introduction that states the main purpose of this work, a lot more background on Arf1 and other molecular players and their relevance to cancer and autocrine signaling.
- rewriting by assuming that most readers don't have the background knowledge to understand some of the key experiments without further guidance.

After a major revision, I'd be happy to evaluate this manuscript again. I think there are many interesting pieces of results here that can be tied together to yield the final picture (Fig. 6) that the authors aimed to reveal. I would be supportive of publication after such a revision.

Major points:
1. Unclear connection between the extracellular, days-long process (Fig. 1B: top row) and the intracellular, hours-long process (Fig. 1B: middle row). The authors establish that intracellular process sets the secretion rate ("f(secretion)" in dX/dt in top row of

Fig. 1B). While it's satisfying to see how the secretion rate (f(secretion)) arises from the intracellular processes, one does not need to know the intracellular processes to obtain the graph of cell number VS days in the top row of Fig. 1B. One would get these curves from a phenomenological model in which the shape of function f(secretion) is chosen to be sigmoidal or hyperbolic (sigmoidal with Hill coefficient = 1) without knowing any of the intracellular processes. So I think Fig. 1B may be framing the work in a misleading way. I think that the bottom row of Fig. 1B ("sensing at the Golgi apparatus") is the main process that the work focuses on rather than the extracellular, slow process depicted in the top row of Fig. 1B. After all, given that the timescale of extracellular processes (days) is widely separated from the timescale of intracellular processes (hours), one does not need to know the faster (intracellular) processes to study the extracellular processes.

2. Related to above: Fig. 1 seems misplaced and unnecessary. It is supposed to summarize the purpose and main conclusions of this study. I don't think a summary of the main conclusions (Fig. 1D) is necessary as a figure here. I think the authors can just mention the main findings at the end of the Introduction. As for setting the stage for this work, I think Fig. 1A is too complicated and can be introduced as a later figure, when the authors describe their results. Fig. 1B's top row is not really linked to the rest of FIg. 1B (see my point 1). Fig. 1C gives an overview of the approaches that the authors use but I don't think this requires a figure and can be stated in a sentence in the introduction. Fig. 1D is the main conclusion of the study which I also don't think should be part of Fig 1. In summary, I would prefer that the authors either eliminate Fig. 1 and start directly with Fig. 2 or that they only retain Fig. 1B's middle and last rows as the new Fig. 1 (simpler and sets a more appropriate stage for their work).

3. The "secretion vs hours" graph (green graph in Fig. 1B) is not precise and can be misleading. By "secretion", do you mean the extracellular concentration of the secreted growth factor or the instantaneous secretion rate? If it's the former, I think this graph cannot be right because it should eventually saturate (once the degradation rate matches the secretion rate). If it's the latter, then the graph still cannot be right because the secretion rate cannot become infinite.

4. Related to point 2: the first page of the Results section (Pg. 5) is confusing because it states the main conclusions and molecular players before the reader is exposed to the experiments that led to these conclusions. Moreover, for non-experts, it's unclear why Golgi, Arf1, and GTPases are mentioned here: they come out of the blue. These are also mentioned here for the first time without telling the non-experts what they are and why there are relevant for studying secretion of growth factors. In fact, the importance of Arf1 is only clear in Fig. 2, due to EGF's role in activating Arf1. It's impossible for a reader to appreciate this before seeing this result in Fig. 2. I think the authors can introduce the readers to these molecular parts and explain their basic relevance to secretion of growth factors (background info) by rewriting the Introduction section. The current Introduction does a good job of giving a broad overview of secrete-and-sense circuitry and its relevance to cancer cells. I recommend the authors extend the Introduction with a background info on the molecular parts. Then I think the Results section can start with the first sentence that describes Fig. 2 ("We first sought to model the impact of GTPase coupling on .... " on Pg. 6).

5. I would be very clear about which growth factor you're talking about. Here, "EGF" is the growth factor. I would just state that in the Introduction to set the stage for the paper.

6. Fig. 2F: why does the Arf1-GTP level not return to a value of 1 after 30 minutes? It apparently plateaus at a level higher than the pre-stimulus level. What is the significance of this plateau? Would the Arf1-GFP level increase again from this plateau if the EGF level increased further to further stimulate ligands?

7. Fig. 2E: this is just a different representation of Fig. 2F and is thus redundant. Instead of this bar graph, I think the authors need to show how Fig. 2F would look when different concentrations of EGF are used to activate Arf1-GTP. I would plot several curves in Fig. 2F, one for each [EGF]. Then, instead of Fig. 2E, the authors can summarize the additional curves in Fig. 2F by plotting a dose-response curve: pulse height for each curve in Fig. 2F VS. [EGF].

8. Related to points 6 & 7: I think the explanation for this experiment is too succinct and can only be understood by experts or readers who spend the time to read the cited paper #44. This experiment should be more fully explained. At the very minimum, explain what biological process the pull-down assay is trying to highlight.

9. Fig. 2G: while this image looks beautiful, why is it important to show the co-localization of Arf1 and GIV for understanding EGF's activation of Arf1? Help the readers by explaining this in the text in simple terms without jargon.

10. Fig 3 - FRET experiments: as with the experiment in Fig. 2F, this experiment seems to be done with one concentration of EGF. The concentration of EGF used should be listed (is the concentration "physiologically relevant"?). Why was this concentration chosen? Why is it not necessary to do a dosage-response curve of the type that I recommend for Fig. 2F?

11. Figs. 3E, 3F, 3J, and 3K: the authors fit all these data to equations to get the curves shown here. It seems that the main purpose of these experiments is to extract some parameters that will go into the equations shown in Fig. 1B. If so, it's unclear what these parameters are and, therefore, what the main purpose of these experiments are. Right now, it just seems like these are just exercises in curve fitting without knowing the main purpose. Overall, the main message and purpose of Fig. 3 is obscured by many (impressive) experiments in Fig. 3.

12. Fig. 4 - simulations: the authors state that these plots are from simulating a "EGF stimuli over a wide range of

concentrations" (Pg. 8). But I'm confused because the experiments in Figs. 2-3 showed a result from a single concentration of EGF (please correct me if I'm wrong; I don't see any plots as a function of [EGF] in Figs. 2-3). This is another reason for repeating the key experiments in Figs. 2-3 with a wide range of EGF concentrations (see my points 7 & 10).

13. Fig. 4 - simulations: there's not enough detail in the text to understand these simulations. At a bare minimum, the authors should describe what exactly is being simulated. The current description is couched in jargon. I don't really know what was varied, how many free parameters there are, what equations are used, and what the main purpose of this modeling is. The authors can give a full description of the model in the supplementary text. But in the main text, the authors should still give enough summary and details so that the reader can understand the main elements and purpose of the simulations without reading the supplementary text.

14. Fig. 5 - Protein-Protein Interaction network: I don't see how this figure and the analysis therein are relevant to the author's study. Given that all the functional assays in Figs. 3-4, why is the PPI analysis necessary? For what purpose? I think that the PPI analysis (Fig. 5) is distracting from the main storyline. It also doesn't help that one short paragraph describes this complicated topic (PPI analysis) in the main text, meaning that most readers won't understand what the result here is and means.

15. Fig. 6 - extracellular processes: this is the only part of the paper that deals with how the EGF affects cell proliferation. A major weakness of this part is that the simulations use different concentrations of EGF (e.g., Fig. 6B). Yet, the intracellular processes were studied with only a single concentration of EGF. Doing the experiments mentioned in my points 7 & 10 with various [EGF] would remedy this issue.

16. Fig. 6 - extracellular processes: it's unclear how the results from Figs. 2-3 are linked to the extracellular processes and modeling described in Fig. 6 (i.e., how the secreted EGF controls cell number over time). The authors can remedy this by better explaining which parameter values in the equations in Fig. 6A come from which experiments.

17. Fig. 7: what is the relevance of this experiment after the modeling in Fig. 6? This figure contains a huge number of panels and is more about how GTPase coupling by GIV is relevant for cell proliferation. But for the storyline, I think it's very jarring to have this come after FIg. 6 which explains how the intracellular components that you described in Figs. 2-3 lead to secretion of EGF. I recommend completely removing Fig. 7 or migrating it to the supplement.

18. Instead of the current Fig. 7, I recommend a diagram that connects all the different results in one picture as a final figure. For example, a pathway diagram that starts with EGF activating Arf1, and then ..... and finally, ending with EGF secretion.

Minor points:
1. Pg. 6: "temporal finiteness" ---> "temporal fitness"? (what does either one mean?)

2. Change title to be more specific to the work done here. The current title is too generic and not very informative.

**POINT BY POINT RESPONSE TO CRITIQUES** (in blue font)

**Editor's comments**:

Guidance provided by Editor:
The reviewers' recommendations are relatively clear, so there is no need to reiterate the points listed below. In particular, both reviewers mentioned that the flow of the manuscript must be substantially revised, and additional clarifications and details need to be provided. Considering these comments, we would ask you to improve the presentation of the study to make the data and the main conclusions easily accessible to the readers. All other issues raised by the reviewers need to be satisfactorily addressed as well. As you may already know, our editorial policy allows in principle a single round of major revision, and it is therefore essential to provide responses to the reviewers' comments that are as complete as possible.

**RESPONSE TO EDITOR's COMMENTS**: We thank the Editor for handling our manuscript and for giving us the opportunity to revise and resubmit this work. In this revised version, we have done our best to not just address the concerns raised, but also provided additional evidence through new experiments. We believe that these additions have improved the manuscript overall. We hope that the Editor and the reviewers agree and find this version suitable for publication.

**Reviewer #1 Critiques**:

**General comments-R1**: In this paper, Qiao, El-Hafeez, Lo and colleagues investigate, using a combined computational and experimental approach, how the coupling between two GTPases (the monomeric GTPase Arf1 and the heterotrimeric GTPase Giabg) imparts to the cells the ability to secrete growth factors and respond to them proportionally, creating a so-called 'secrete-and-sense' loop that explains how, for instance, cancer cells can sustain themselves even in the absence of external growth factors.

The authors build a mathematical model made of two parts: one that models the circuit, localized at the Golgi, that senses the growth factor; and one that models protein secretion and cell growth and proliferation, which are a consequence of the sensing part. The three processes (sensing, secretion and proliferation) happen on different time scales; thus, the models are distinct.

The authors show how the guanine nucleotide exchange factor GIV/Girdin functions as a bridge between Arf1 and Giabg: first, Arf1 is activated by the growth factor EGF (meaning it gets into the GTP-bound form); the consequence of Arf1 activation is the recruitment of GIV/Girdin on Golgi membranes. Once recruited there, GIV/Girdin activates Giabgby triggering the dissociation of Gai1 from Gb1g2. There are then two negative feedback loops that takes place: (1) GIV/Girdin binds to and recruits to the Golgi membranes ArfGAP2/3, which being a GTPase activating factor (GAF) for Arf1, makes Arf1 go back to the GDP-bound state, which is the inactive state; (2) free Gb1g2 is a co-factor for ArfGAP2/3, therefore, it also contributes to terminating the signaling cascade. The authors convincingly show that GIV/Girdin is necessary for the coupling of the two GTPases, for the dose-dependent secretion of various cargos, which, in turn, is necessary for the survival of the cells when they rely on autocrine secretion. Thus, taken together, this paper gives strong evidence of how cells can sense and respond to growth factors secreted by them and provides interesting ideas on how one could interfere with this process to, for instance, counteract cancer.

I was positively impressed by the amount of data, and by the modeling, which also entails a bioinformatics aspect (the authors generated Arf1's interactome networks with and without GIV/Girdin to predict what the role of this protein).

I actually do not have suggestions for additional experiments! I rather have suggestions to improve the flow of the paper and the figures.

**RESPONSE TO GENERAL COMMENTS OF REVIEWER #1**: We are grateful to the reviewer for his/her encouraging comments and several generous words/phrases of praise (highlighted above). We also express our gratitude for the time and effort that this reviewer put into making such detailed, specific, and thoughtful suggestions on how to improve the manuscript. Although this reviewer did not ask for any additional experiments, we felt that some were necessary to improve the rigor of our claim that the molecular circuitry we describe here indeed supports self-sufficiency in growth factor signaling (and not an artifact in few cell lines). To this end, we have now validated our findings by TMT proteomics studies in two cell lines (HeLa and MDA MB-231) and presented such evidence as a new Figure 7 in this revised submission [see last paragraph in the 'Results' section and Legend for Fig 7].

We strongly believe that implementing these changes have indeed helped in achieving that goal. We hope the reviewer agrees with us.

**Comment 1**: I think the introduction lacks important parts, such as what are GTPases, GAPs, GEFs, what is Arf1, Gi, ArfGAP2/3, etc. This information is either entirely absent or scattered in the results. I believe it would help the reader to find it all in one place, the introduction.

**Response 1**: Thank you for this suggestion. We agree.
**Action(s) Taken**: In the revision, we added the detailed explanations for these molecules in the introduction. For the convenience of the reviewer, we have copied and pasted the revised section below, as it appears on the revised manuscript.

Despite these insights, the core mechanisms of cell secretion that impart cell autonomy remains poorly understood. To begin with, it is still unknown whether or not secretion is proportional to growth factor stimulation, and whether such secretion is sufficient to support cell survival, perhaps via closed loop autocrine sensing and signaling (the so-called 'secrete-and-sense' loop (Youk and Lim, 2014)). A recent study has shown that the secretory functions of the Golgi apparatus requires the unlikely coupling of two distinct species of GTPases at the Golgi (Lo et al., 2015) (**Figure 1A**): one is small or monomeric (m) GTPase Arf1, and the other is heterotrimeric (t) GTPases Gi. GTPases serve as molecular switches that gate signal transduction: "on" when GTP-bound (active) and "off" when GDP-bound (inactive). The "ADP-ribosylation factor" (Arf1) (Kahn and Gilman, 1986) mGTPase is localized to the Golgi complex in mammalian cells and is essential for the secretory pathway (Stearns et al., 1990); it associates with Golgi membranes upon activation, and is released from Golgi membranes into the cytosol upon inactivation. Such cycles of association and dissociation are regulated by Golgi-associated, guanine nucleotide exchange factors (GEFs) and GTPase activating proteins (GAPs). Trimeric GTPases were detected in the Golgi over three decades ago (Barr et al., 1992; Stow et al., 1991), and numerous

4 | Page

studies have provided clues that they may regulate membrane traffic and maintain the structural integrity of the Golgi (reviewed in (Cancino and Luini, 2013)). However, the concept of G protein activation at the Golgi and the potential impact of such activation remained controversial, primarily due to the lack of direct proof of G protein activation. The study that reported coupling of Arf1 mGTPase and Giαβγ tGTPase provided direct evidence, the first of its kind, that the two GTPases are coupled by a linker protein, Gα-Interacting Vesicle-associated protein (GIV) (Lo et al., 2015). Activation of Arf1 mGTPase facilitates the recruitment of GIV on the membrane via a direct, nucleotide-dependent interaction. Upon recruitment, GIV binds and activates Gαi serving its role as a GEF for the tGTPase, Gi. Such activation of Gi at the Golgi affects two fundamental functions of the Golgi, i.e., vesicle trafficking and the structural organization of the Golgi stacks-- both via modulation of Arf1 signaling. These findings firmly established that Gαi is functionally active in the Golgi.

Because tGTPases are known to primarily transduce extracellular signals ('sensing') into intracellular signals that shape cellular responses, we asked how coupling of the two GTPases, one that guards cell secretion (Arf1) and another that gates signal sensing (Gi), may impact the cell's ability to secrete-and-sense. In systematically interrogating this question, we viewed the experimentally validated interactions and functions of the two GTPases and their GEFs and GAPs as a circuit of coupled GTPases. Such coupling, whose structural basis has been experimentally validated (**Figure 1A-*right***), forms a closed loop that is comprised of one forward reaction and two negative feedback loops (**Figure 1A-*left*; Figure EV1; Movie EV1; Materials and Methods**). The forward reaction is the recruitment of GIV/Girdin by active Arf1 on Golgi membranes (arrow 1). GIV is a multi-modular cytosolic signal transducer that is a prototypical member of the family of guanine nucleotide exchange modulators (GEM) of tGTPases; GIV's GEM domain binds and activates the tGTPase Gαi, and thereby, serves as a tGEF within this circuit. One negative feedback loop is that GIV can improve the GAP for Arf1---ArfGAP2/3, thus terminating Arf1 signaling (arrow 2); the other is due to GIV's role as GEF to activate Gi and thus enhance Arf1GAP2//3, which also lead to the termination of Arf1 signaling (arrow 3). This phenomenon of co-regulation between the two classes of GTPases maintains Golgi shape and function, two closely intertwined processes that are regulated by Arf1. The triggers for and the consequence(s) of such co-regulation on signal sensing/response remained unknown.

**Comment 2**: The first part of the results belongs, in my opinion, to the end of the introduction. Typically, the introduction ends with some paragraphs describing what was done in the paper. In the results, I would go directly into the description of the experiments, and avoid statements on the approach etc.

**Response 2**: We agree that the introduction in the original submission lacked some of the typical statements that describe what was done, and what was found in the paper.

**Action(s) Taken**: In this revised submission, we have now moved significant portions of the first part of results to the end of Introduction. For the convenience of the reviewer, we have copied and pasted the last paragraph of the Introduction section below, as it appears on the revised manuscript.

> Because coupling of two species of GTPase switches, Arf1 and Gi, with feedback control is likely to generate complex, nonlinear, and non-intuitive emergent properties, we use cross-disciplinary approaches to dissect the role of the coupled GTPases within the secretory pathway and explore its functional significance in eukaryotic cells. Using systems biology approaches and explicit integration of experimental biology and computational methods, we also assess the impact of perturbing this motif, i.e., uncoupling the GTPases. Our findings show how coupling makes secretion responsive to growth factors, in particular the epidermal growth factor (EGF), and appears to impart secretion-coupled autonomy.

We also debated removing the entire first section of results, but ultimately felt compelled that we must retain for a broad audience some of the key aspects of the rationalized approach, the model systems, and readouts. This is because we anticipate that the readers of this journal may be domain experts in one area/discipline, but not others, or maybe experts in both, but not familiar with the GEM/GTPase biology. Based on the feedback during internal reviews among colleagues, we felt that it was important to explicitly lay out that the model has an experimentally constrained module and a predictive module, and to rationalize why and which cancer cells were chosen as model systems. For the convenience of the reviewer, we have copied and pasted the first paragraph of the Results section below, as it appears on the revised manuscript.

> We began by developing a dynamical systems model for this coupled circuit (**Figure 1B**; see **Materials and Methods**) and drawing clues from protein-protein interaction (PPI) network analyses, to generate testable hypotheses and validate them experimentally. The integrated approach allowed us to connect across time scales of the emergent behavior of the coupled GTPase circuit with cellular secretion, cell survival, and ultimately, secretion-coupled survival, i.e., autocrine autonomy.

**Comment 3**: "unlikely": this adjective sounds misplaced here. Unlikely means improbable, implausible. I would keep only "distinct".
**Response 3**: We agree.
**Action(s) Taken**: In the revised version, we have deleted the word “unlikely”. We retained the word “distinct”.

**Comment 4**: The authors write: "using the general framework presented in 23,24": I would find it useful to the readers if the framework were at least named and briefly explained.
**Response 4**: We agree. The framework in these two works developed normalized Hill functions to model biochemical reaction rates. This approach captures the key timescales and molecular players rather than focusing on the specific kinetics of biochemical reactions.
**Action(s) Taken**: We have now removed this phrase from the opening sentence in the Results section. Instead, we have explained in the following paragraph our rationalized approach to modeling and experimental studies (model systems, readouts, etc.).

**Comment 5**: ERGIC: please define
**Response 5**: Thank you for pointing this out.
**Action(s) Taken**: In the revision, we added the full name of ERGIC, i.e., endoplasmic-reticulum–Golgi intermediate compartment, when ERGIC appears first in text. For the convenience of the reviewer, we have copied and pasted the relevant section below, as it appears on the revised manuscript.

> within the secretory pathway, i.e., the ERGIC (endoplasmic-reticulum–Golgi intermediate compartment) to the

**Comment 6**: Figure 2A: I honestly do not see how this panel represents the "coupling"...

**Response 6**: We apologize for the lack of clarity. The confusion probably stems from redundant use of schematics in Figure 1 and 2.

**Action(s) Taken**: In the revision, we have taken two measures to achieve clarity and streamline our figure panels:

1) **Figure 1A** now combines the content that was presented in a redundant form in panels 1A and 2A.

2) Text in Introduction is updated to explain what coupling means, and what it achieves. We hope these additions make it clear to the reader what we mean by “coupled”. Please refer to response 1.

**Comment 7**: GST-GAT domain: is GST the tag (Glutathione S Transferase)? If so, why didn't the authors use an anti-GST antibody? Why showing the Ponceau staining?

**Response 7**: This is an important question. Glutathione S Transferase (GST)-tagging of proteins is a commonly used methodology to express and purify recombinant proteins. In general, it is accepted that the best way to confirm that the expressed protein is well-folded and of high quality is to conduct ponceau S/Coomassie staining of full gels to verify that most of the purified protein is expressed as single band at expected length (i.e., not as GST breakdown or other breakdown products [1]). Conducting ponceauS or similar total protein staining also helps verify protein amounts (molarity). While immunoblotting with GST can detect the tagged fragments, it ignores fragments that lack part or all of the GST tag.

**Action(s) Taken**: We have expanded the abbreviation (Glutathione S Transferase) in the text.

We measured Arf1 activity in response to EGF using an established pull-down assay (**Figure 2A-B**). with the Glutathione S Transferase (GST)-tagged GAT domain of GGA3; this domain is known to selectively bind the active GTP-bound pool of Arf1 (Cohen & Donaldson, 2010). The levels of Arf1•GTP were increased ~3-fold

**Comment 8**: Figure 1C: I do not see the need for showing such a scheme. I think readers can understand the concept of a pull-down without seeing it depicted.

**Response 8**: Thank you for the suggestion.
**Action(s) Taken**: In the revision, we have deleted this panel.

**Comment 9**: Excellent fitness: I think that R2 of 0.54 does not represent excellent, but rather a good fit between model and data...

**Response 9**: We agree.

**Action(s) Taken**: In the revision, we have rewritten the text. For the convenience of the reviewer, we have copied and pasted the relevant section below, as it appears on the revised manuscript.

We fit the above experiment data by tuning the kinetic parameters. We obtained a good fit for the fold change of Gi activation in both control and shGIV cells (**Figure 3G;** $R^2$ and RMSE, 0.54 and 0.41 for control cells; −0.44 and 0.71 for shGIV cells). The low level of GIV in shGIV cells was mimicked by decreasing the levels of

8 | Page

expression of GIV to 10% of that in control cells (**Figure 3C**). Thus, the model matched the overall trend of experiment data in both cells (see **Table EV1** for model parameters).

**Comment 10**: In the paper, the word "stimuli" is often used when the singular form, stimulus, should be used instead. I suggest the authors to check this.

**Response 10**: We thank the reviewer for pointing this out. We agree.

**Action(s) Taken**: In the revision, we replaced the word “stimuli” by “stimulus” in most places other than 4 instances where we intended to use the word to reflect plurality.

**Comment 11**: Man II: what is this?

**Response 11**: Apologies for the overuse of abbreviations without expanding them when used first in text. Golgi α-mannosidase II (GMII) is a glycoside hydrolase playing a crucial role in the *N*-glycosylation pathway. As a transmembrane protein, it is often used as an organelle marker for the Golgi [2].

**Action(s) Taken**: In this revised submission, we have added the relevant citation and rationale for the use of Man-II staining. For the convenience of the reviewer, we have copied and pasted the relevant section below, as it appears on the revised manuscript.

relevant in physiology, we sought to validate our FRET-based findings on endogenous Gi. To this end, we performed confocal immunofluorescence microscopy using a *bona fide* marker of the organelle, the Golgi-localized α-mannosidase II (Man II) (Zuber et al., 2000), and anti-Gαi•GTP mAb, which selectively recognizes the active (GTP-bound) conformation of the G protein (Lane et al., 2008). These signals colocalized not only in

**Comment 12**: "These findings simulate": findings to not simulate. I would rephrase: "the model predictions reproduced what was experimentally demonstrated before18"
**Response 12**: We thank the reviewer for this suggestion.
**Action(s) Taken**: In the revision, we rephrased the words as per the suggestions of this and other reviewers. More importantly, we used this opportunity to clarify that the "AND GATE" like logical operation was modeled based on prior experiments (and avoid any confusion or misrepresentation that it happened the other way, i.e., the model somehow predicted "AND GATE" like logical operation). For the convenience of the reviewer, we have copied and pasted the relevant section below, as it appears on the revised manuscript.

Finally, we used the model which was fitted to the experimental data in **Figure 2C** and **Figure 3G** to make predictions. We conducted two simulations: large decrease in the GIV level to simulate the Arf1 activation dynamics in shGIV cell (red line in **Figure 3J**), and delete either arrows 2 or 3 to simulate the Arf1 and Gi activation dynamics for the uncoupled GTPase switches. Based on the experimental results before (Lo et al., 2015), arrows 2 and 3 are modeled by an 'AND gate'-like digital logical operation (Kime and Mano, 2003), i.e., a HIGH output (ArfGAP2/3 activity, and resultant termination of Arf1 signaling) results only if both the inputs to the AND gate (arrows 2 and 3) are HIGH. We also tested the 'OR' logic for the negative feedback (**Figure EV3**) and found the model predictions to be indistinguishable from AND gate. It is possible that one of these logical modes of operation is more efficient than the other under certain circumstances. For the first simulation, the simulated Arf1 activation dynamics (red line in **Figure 3J**) captured the sustained activation of Arf1 dynamics in shGIV cells, indicating the efficiency of the model. For the second simulation, the simulated Arf1 dynamics (green line in **Figure 3J**) is the same as that in shGIV cells, suggesting the equivalency of deleting GIV and uncoupling GTPase switch. The simulated Gi dynamics (green line in **Figure 3K**) is similar to (maybe even slightly higher than) that in control cells, which is consistent with the fact that the feedback loops have no effect on Gi. Thus, negative feedback within the 'closed-loop control' exerts significant effect on the mGTPase (Arf1) and little or no effect on the tGTPase (Gi).

**Comment 13**: At the end of page 8, in the sentence starting with "; because the first input", a verb seems to be missing.
**Response 13**: Agree; thank you for picking up this error.
**Action(s) Taken**: We have now re-written the sentence to fix the error (see highlighted sentence below).

receptor occupancy faithfully. We regarded the mGEF as an alternative to the receptor because it serves as the first input to the coupled circuit via its ability to trigger the activation of the mGTPase switch. Moreover, a close

**Comment 14**: When stating that negative feedback reduces noise, I would suggest the authors to cite also this experimental paper: Becksei, Serrano: Engineering stability in gene networks by autoregulation. Nature volume 405, pages 590-593 (2000).

**Response 14**: We apologize for this error of omission and very much appreciate the reviewer's suggestion.
**Action(s) Taken**: We have included this citation in the revised submission.

**Comment 15**: DoRA: please define
**Response 15**: Sorry for the confusion. DoRA is the abbreviation of dose-response alignment.
**Action(s) Taken**: In the revision, we added "(DoRA)" after we mentioned the dose-response alignment: Typically, dose-response alignment (DoRA) refers to the close match of the receptor occupancy and the downstream molecules.

**Comment 16**: "We observed 3 things„: I would rephrase: "we made three observations"
**Response 16**: We appreciate this suggestion; we agree.
**Action(s) Taken**: In the revision, we rephrased the words as the reviewer suggested.

tool to inhibit secretion via its ability to inhibit Arf1 activation (Prieto-Dominguez et al, 2019) (**Figure 6F**). We made three observations: (i) cells with coupled circuits have a significant survival advantage in serum-restricted

**Comment 17**: The authors write existing theories linking genetic circuits to cellular autonomy exist (and cite several papers), but state they do not apply to the eukaryotic cell. I saw that reference 63 is a paper in which the eukaryote Dictystilium was used. So why this statement?
**Response 17**: The reviewer is right. We had failed to specify that none of the prior studies/models apply to eukaryotic cells of multicellular organisms.
**Action(s) Taken**: We have rectified this error now. The relevant section is pasted below.

Finally, when it comes to the field of cancer cell biology, it is well accepted that self-sufficiency in growth signaling is a hallmark of all cancer cells (Hanahan and Weinberg, 2000); we show here how cells achieve such self-sufficiency for the prototypical growth factor system, i.e., EGF/EGFR. Existing theories linking genetic circuits to cellular autonomy, although quantifiable and tunable (Doğaner et al., 2016; Kamino et al., 2017; Maire and Youk, 2015; Tang et al., 2021; Youk and Lim, 2014), don't apply to the multicellular eukaryotes. In dissecting

In addition, we have made two other changes:

1) Title was revised to reflect the same, i.e., importance of the findings in higher order eukaryotes in which cells maintain autonomy in the setting of multicellularity (also to be responsive to a comment from Reviewer #2, where it was suggested that we make the title more focused).
2) This additional clarity is largely drawn from new data and analyses (**Figure 7**) which showed that the PM-specific proteome is enriched in key players of the signaling pathways that are unique to multicellular life.

**Comment 18**: GEM module: GEM domain?
**Response 18**: We agree.
**Action(s) Taken**: The text has been edited as per reviewer's suggestion.

2015; Puseenam et al., 2009; Yamaguchi et al., 2010); its GEM domain (a short motif that binds and modulates tGTPases) evolved later in fish (DiGiacomo et al., 2018) and remains to date. Thus, the coupled GTPase circuit

**Comment 19**: "Our model chose to test": a model does not choose. Re-phrase "we chose to model..."
**Response 19**: Thank you.
**Action(s) Taken**: In the revision, we rephrased the words as the reviewer suggested.

**Comment 20**: In the legend to Figure 1B: GIV-GEM is mentioned. This is not mentioned anywhere else in the next. In the text, it is always GIV/Girdin. Please explain.
**Response 20**: We apologize for this inconsistency in the nomenclature.
**Action(s) Taken**: In this revised submission, we have addressed this issue upfront in the Introduction. For the convenience of the reviewer, we have copied and pasted the relevant section below, as it appears on the revised manuscript.

**Methods**). The forward reaction is the recruitment of GIV/Girdin by active Arf1 on Golgi membranes (arrow 1). GIV is a multi-modular cytosolic signal transducer that is a prototypical member of the family of guanine nucleotide exchange modulators (GEM) of tGTPases; GIV's GEM domain binds and activates the tGTPase Gαi, and thereby, serves as a tGEF within this circuit. One negative feedback loop is that GIV can improve the GAP

**Comment 21**: I do not think the scheme in Fig. 3G is needed
**Response 21**: Thank you for the suggestion.
**Action(s) Taken**: In the revision, we have deleted the scheme in Fig. 3G.

**Comment 22**: Figure 3I: what do low and high indicate?
**Response 22**: These reflect exposures for immunoblots. This was an unintended error of omission.
**Action(s) Taken**: This detail has been now included in the figure legend.

**Comment 23**: Figure 6C: is this really linear?
**Response 23**: This question refers to a figure panel that is 5C in the current version. It is not linear since the best-fit Hill coefficient is 2.92.

**Action(s) Taken**: In the revision, we rephased the text related to Figure 5C as follows:

for the comparison with the coupled GTPases system. Intriguingly, secretion in the coupled state shows different response for most ranges of Gi activity (tG*; **Figure 5C**), indicating a faithful information transduction between Gi and secretion. Besides, the cell number is higher for the coupled versus the uncoupled cells (**Figure 5E** and

**Reviewer #2 Critiques**:

**General comments-R2**: Previous work identified the molecular parts that cancer cells use to secrete and sense growth factors. But we currently do not understand how these parts combine to supposedly allow a cancer cell to autonomously grow. Specifically, for a cancer cell, it's unclear how a sensing of its own growth factor controls the secretion of that growth factor. Answering this question inherently requires a quantitative and systems approach. The authors use such an integrated approach to try to address this fundamental question in cancer biology.

To answer this question, the authors begin with a model that spans multiple scales both spatially and temporally (Fig. 1). The authors investigate the relatively fast, intracellular processing of growth factors in the ER and golgi appartus and the relatively slow, extracellular secretion and sensing of these growth factors. The mathematical model describing these processes are simple and phenomenological equations that are experimentally constrained, with the constraints coming from the authors' own measurements in HeLa cells, breast cancer cells (MDA-MB231), and in vitro experiments. These experiments focused on two GTPases at the golgi, "Arf1" and "Gi", and how they co-regulate the production and eventual secretion of the growth factors. The main finding from studying Arf1 and Gi is that the GTP hydrolysis rate, which is regulated by mGAP, primarily sets the secretion rate of growth factors.

The main contribution of this work is systematically tying together various intracellular parts that control how a cell senses and secretes EGF. It's impressive how the authors used various techniques to assemble the various parts together. But this assembly process is not clearly explained. So I think it's difficult to see the forest from the trees. I think a rewriting of the manuscript will highlight the main, systems-level conclusions without having the reader getting lost in all the details.

In fact, I found this paper to be very difficult to understand. For one, it was challenging to understand many of the experiments because the authors did not fully explain the experimental set up and the goal of each experiment. The manuscript also assumes that the reader have a sufficient background knowledge of Arf1, GTPases, golgi apparatus, and the importance of all these to cancer cells.

Overall recommendation:
This study contains many and technically impressive experiments. The authors do an admirable job of trying to combine these different experiments, spanning vast time scales (hours to days) and spatial scales (organelle to multiple cells). But I think the process of combining the different results isn't explained well or is missing some key experiments/interpretations. Thus, I recommend a major revision that mainly focuses on two aspects:

1. Redoing some of the key experiments in Figs. 2-3 with various concentrations of EGF as opposed to the current presentation which used just one or a few concentrations of EGF.

2. Vastly rewriting the manuscript by:
- giving a more full explanations of the experimental details
- excluding some figures and results (e.g., PPI analysis in Fig. 5 and large parts of Fig. 7)
- giving a roadmap along the way that says which parameters are being fitted in the experiments in Figs. 2-3 and how these parameter values that you get here will be used in the model in Fig. 6 (i.e., which parameters in which equations in Fig. 6 are being measured in the experiments of Figs. 2-3?)
- a more extensive Introduction that states the main purpose of this work, a lot more background on Arf1 and other molecular players and their relevance to cancer and autocrine signaling.
- rewriting by assuming that most readers don't have the background knowledge to understand some of the key experiments without further guidance.

After a major revision, I'd be happy to evaluate this manuscript again. I think there are many interesting pieces of results here that can be tied together to yield the final picture (Fig. 6) that the authors aimed to reveal. I would be supportive of publication after such a revision.

**RESPONSE TO GENERAL COMMENTS OF REVIEWER #2**: We are very pleased to see that the reviewer's comments are generally positive. More specifically, he/she views our contribution of "*systematically*" tying together various intracellular parts that control how cells sense and secrete EGF and the use of various techniques to do so as "*impressive*" and "*admirable*".

However, the reviewer makes two very specific suggestions on how to revise the manuscript with the intent to make the manuscript more accessible to readers who may/may not be familiar with all the concepts, molecules, or experimental approaches.

We wanted to thank the reviewer for taking the time to make such detailed and thoughtful suggestions. We have taken them very seriously and revised the manuscript accordingly. We believe that these extensive rearrangements and additional analyses and experimental studies have greatly enhanced the fundamental impact of our discoveries while making them more accessible to broad readership of the journal. We hope the reviewer agrees with our assessment.

**RESPONSE TO SPECIFIC COMMENTS:**

**Comment 1**: Unclear connection between the extracellular, days-long process (Fig. 1B: top row) and the intracellular, hours-long process (Fig. 1B: middle row). The authors establish that intracellular process sets the secretion rate ("f(secretion)" in dX/dt in top row of Fig. 1B). While it's satisfying to see how the secretion rate (f(secretion)) arises from the intracellular processes, one does not need to know the intracellular processes to obtain the graph of cell number VS days in the top row of Fig. 1B. One would get these curves from a phenomenological model in which the shape of function f(secretion) is chosen to be sigmoidal or hyperbolic (sigmoidal with Hill coefficient = 1) without knowing any of the intracellular processes. So I think Fig. 1B may be framing the work in a misleading way. I think that the bottom row of Fig. 1B ("sensing at the Golgi apparatus") is the main process that the work focuses on rather than the extracellular, slow process depicted in the top row of Fig. 1B. After all, given that the timescale of extracellular processes (days) is widely separated from the timescale of intracellular processes (hours), one does not need to know the faster (intracellular) processes to study the extracellular processes.

**Response 1**: The above comments stem from our use of schematics in the original version of **Fig 1**, which did not do a good job of showing how our model is connected across time scales. In our model, the extracellular, days-long process is connected to the intracellular, minutes-to-hours-long process of growth factor secretion; secretion, on the other hand, is set as a function of mGAP (i.e., GAP for Arf1) which is responsible for maintaining the finiteness of Arf1 activity to a few seconds-minutes.

**Action(s) Taken**: In the revision, we revised **Figure 1** and replotted **Fig. 1B** to better show how extracellular process is linked to the intracellular process.

**Comment 2**: Related to above: Fig. 1 seems misplaced and unnecessary. It is supposed to summarize the purpose and main conclusions of this study. I don't think a summary of the main conclusions (Fig. 1D) is necessary as a figure here. I think the authors can just mention the main findings at the end of the Introduction. As for setting the stage for this work, I think Fig. 1A is too complicated and can be introduced as a later figure, when the authors describe their results. Fig. 1B's top row is not really linked to the rest of FIg. 1B (see my point 1). Fig. 1C gives an overview of the approaches that the authors use but I don't think this requires a figure and can be stated in a sentence in the introduction. Fig. 1D is the main conclusion of the study which I also don't think should be part of Fig 1. In summary, I would prefer that the authors either eliminate Fig. 1 and start directly with Fig. 2 or that they only retain Fig. 1B's middle and last rows as the new Fig. 1 (simpler and sets a more appropriate stage for their work).

**Response 2**: We agree that the original Figure 1 was excessively complicated and also had redundancies with parts of Fig 2.
**Action(s) Taken**: In the revision, we revised **Figure 1** to simplify it along the lines suggested.

**Comment 3**: The "secretion vs hours" graph (green graph in Fig. 1B) is not precise and can be misleading. By "secretion", do you mean the extracellular concentration of the secreted growth factor or the instantaneous secretion rate? If it's the former, I think this graph cannot be right because it should eventually saturate (once the degradation rate matches the secretion rate). If it's the latter, then the graph still cannot be right because the secretion rate cannot become infinite.
**Response 3**: We agree on the reviewer that the curve in Fig. 1B should saturate. In the manuscript, the “secretion” means the instantaneous secretion rate.
**Action(s) Taken**: In the revision, we replotted the secretion panel in Fig. 1B to ensure that the curve comes to saturation after a long time.

**Comment 4**: Related to point 2: the first page of the Results section (Pg. 5) is confusing because it states the main conclusions and molecular players before the reader is exposed to the experiments that led to these conclusions. Moreover, for non-experts, it's unclear why Golgi, Arf1, and GTPases are mentioned here: they come out of the blue. These are also mentioned here for the first time without telling the non-experts what they are and why there are relevant for studying secretion of growth factors. In fact, the importance of Arf1 is only clear in Fig. 2, due to EGF's role in activating Arf1. It's impossible for a reader to appreciate this before seeing this result in Fig. 2. I think the authors can

introduce the readers to these molecular parts and explain their basic relevance to secretion of growth factors (background info) by rewriting the Introduction section. The current Introduction does a good job of giving a broad overview of secrete-and-sense circuitry and its relevance to cancer cells. I recommend the authors extend the Introduction with a background info on the molecular parts. Then I think the Results section can start with the first sentence that describes Fig. 2 ("We first sought to model the impact of GTPase coupling on .... " on Pg. 6).

**Response 4**: Thank you for the good suggestion.
**Action(s) Taken**: In the revision, we added the following text in the introduction. Consequently, the results section now starts by directly diving into the modeling approach and rationale for model systems. We hope the reviewer finds this version more streamlined.

Despite these insights, the core mechanisms of cell secretion that impart cell autonomy remains poorly understood. To begin with, it is still unknown whether or not secretion is proportional to growth factor stimulation, and whether such secretion is sufficient to support cell survival, perhaps via closed loop autocrine sensing and signaling (the so-called 'secrete-and-sense' loop (Youk and Lim, 2014)). A recent study has shown that the secretory functions of the Golgi apparatus requires the unlikely coupling of two distinct species of GTPases at the Golgi (Lo et al., 2015) (**Figure 1A**): one is small or monomeric (m) GTPase Arf1, and the other is heterotrimeric (t) GTPases Gi. GTPases serve as molecular switches that gate signal transduction: "on" when GTP-bound (active) and "off" when GDP-bound (inactive). The "ADP-ribosylation factor" (Arf1) (Kahn and Gilman, 1986) mGTPase is localized to the Golgi complex in mammalian cells and is essential for the secretory pathway (Stearns et al., 1990); it associates with Golgi membranes upon activation, and is released from Golgi membranes into the cytosol upon inactivation. Such cycles of association and dissociation are regulated by Golgi-associated, guanine nucleotide exchange factors (GEFs) and GTPase activating proteins (GAPs). Trimeric GTPases were detected in the Golgi over three decades ago (Barr et al., 1992; Stow et al., 1991), and numerous

4 | Page

studies have provided clues that they may regulate membrane traffic and maintain the structural integrity of the Golgi (reviewed in (Cancino and Luini, 2013)). However, the concept of G protein activation at the Golgi and the potential impact of such activation remained controversial, primarily due to the lack of direct proof of G protein activation. The study that reported coupling of Arf1 mGTPase and Giαβγ tGTPase provided direct evidence, the first of its kind, that the two GTPases are coupled by a linker protein, Gα-Interacting Vesicle-associated protein (GIV) (Lo et al., 2015). Activation of Arf1 mGTPase facilitates the recruitment of GIV on the membrane via a direct, nucleotide-dependent interaction. Upon recruitment, GIV binds and activates Gαi serving its role as a GEF for the tGTPase, Gi. Such activation of Gi at the Golgi affects two fundamental functions of the Golgi, i.e., vesicle trafficking and the structural organization of the Golgi stacks-- both via modulation of Arf1 signaling. These findings firmly established that Gαi is functionally active in the Golgi.

Because tGTPases are known to primarily transduce extracellular signals ('sensing') into intracellular signals that shape cellular responses, we asked how coupling of the two GTPases, one that guards cell secretion (Arf1) and another that gates signal sensing (Gi), may impact the cell's ability to secrete-and-sense. In systematically interrogating this question, we viewed the experimentally validated interactions and functions of the two GTPases and their GEFs and GAPs as a circuit of coupled GTPases. Such coupling, whose structural basis has been experimentally validated (**Figure 1A-*right***), forms a closed loop that is comprised of one forward reaction and two negative feedback loops (**Figure 1A-*left*; Figure EV1; Movie EV1; Materials and Methods**). The forward reaction is the recruitment of GIV/Girdin by active Arf1 on Golgi membranes (arrow 1). GIV is a multi-modular cytosolic signal transducer that is a prototypical member of the family of guanine nucleotide exchange modulators (GEM) of tGTPases; GIV's GEM domain binds and activates the tGTPase Gαi, and thereby, serves as a tGEF within this circuit. One negative feedback loop is that GIV can improve the GAP for Arf1---ArfGAP2/3, thus terminating Arf1 signaling (arrow 2); the other is due to GIV's role as GEF to activate Gi and thus enhance Arf1GAP2//3, which also lead to the termination of Arf1 signaling (arrow 3). This phenomenon of co-regulation between the two classes of GTPases maintains Golgi shape and function, two closely intertwined processes that are regulated by Arf1. The triggers for and the consequence(s) of such co-regulation on signal sensing/response remained unknown.

Because coupling of two species of GTPase switches, Arf1 and Gi, with feedback control is likely to generate complex, nonlinear, and non-intuitive emergent properties, we use cross-disciplinary approaches to dissect the role of the coupled GTPases within the secretory pathway and explore its functional significance in eukaryotic cells. Using systems biology approaches and explicit integration of experimental biology and computational methods, we also assess the impact of perturbing this motif, i.e., uncoupling the GTPases. Our findings show how coupling makes secretion responsive to growth factors, in particular the epidermal growth factor (EGF), and appears to impart secretion-coupled autonomy.

**Comment 5**: I would be very clear about which growth factor you're talking about. Here, "EGF" is the growth factor. I would just state that in the Introduction to set the stage for the paper.

**Response 5**: We understand the reviewer's viewpoint and initially toyed with the idea ourselves. However, there are two major issues that we took into consideration to ultimately decide not to: 1) We have done many assays with serum (FBS; i.e., undefined growth factors in media) at various concentration ranges. Hence, it did not seem right to claim that this pathway is specific to EGF/EGFR. 2) We, however, do share the reviewer's viewpoint that the molecular machinery that we define here is largely EGF/EGFR centric, as highlighted by our proteomic analyses that have been included in the revised manuscript as **Figure 9**. These studies have squarely pointed to a role of the Golgi localized machinery in maintaining secretion-coupled autonomy within the EGF/EGFR pathway, and that such autonomy has important ramifications in other pathways that are critical for cell-cell communication in multicellular Eukaryotes.

**Action(s) Taken**: We have made the following edit in the last paragraph of Introduction.

Because coupling of two species of GTPase switches, Arf1 and Gi, with feedback control is likely to generate complex, nonlinear, and non-intuitive emergent properties, we use cross-disciplinary approaches to dissect the role of the coupled GTPases within the secretory pathway and explore its functional significance in eukaryotic cells. Using systems biology approaches and explicit integration of experimental biology and computational methods, we also assess the impact of perturbing this motif, i.e., uncoupling the GTPases. Our findings show how coupling makes secretion responsive to growth factors, in particular the epidermal growth factor (EGF), and appears to impart secretion-coupled autonomy.

In addition, we have devoted an entire figure (**Figure 7**) consisting of new TMT proteomics analysis in two cell lines, with/without coupled circuit and analyzed it to pinpoint the nature of the growth signaling autonomy to be EGF/EGFR/ERBB centric.

**Comment 6**: Fig. 2F: why does the Arf1-GTP level not return to a value of 1 after 30 minutes? It apparently plateaus at a level higher than the pre-stimulus level. What is the significance of this plateau? Would the Arf1-GFP level increase again from this plateau if the EGF level increased further to further stimulate ligands?

**Response 6**: This is an important observation, which we had failed to explain/address in the original version of the manuscript. We measured Arf1 activity in starved state (0.2% FBS overnight for ~16 h), and then at various time points after acute EGF stimulation. We believe that the slightly elevated activity at ~30 min (compared to pre-stim/starved state) represents steady-state activity.

**Action(s) Taken**: In the revised submission, we have now addressed this observation in text/results section.

We measured Arf1 activity in response to EGF using an established pull-down assay (**Figure 2A-B**). with the Glutathione S Transferase (GST)-tagged GAT domain of GGA3; this domain is known to selectively bind the active GTP-bound pool of Arf1 (Cohen and Donaldson, 2010). The levels of Arf1•GTP were increased ~3-fold within 5 min after ligand stimulation, followed by a return towards baseline by 30 min, which we assume reflects the level of Arf1 activity in cells at a steady-state (**Figure 2B**). These temporal dynamics were used to fit the parameters for Arf1 activity in the computational model of the circuit (blue line in **Figure 2C**) ($R^2$ and normalized RMSE are 0.72 and 0.19 respectively; see **Materials and Methods** and **Table EV1** for model parameters). Such fitting completed the characterization of the first GTPase switch, i.e., Arf1; in this case, the input is ligand stimulus (EGF) and the output is Arf1-GTP (OUTPUT #1; mG*).

**Comment 7**: Fig. 2E: this is just a different representation of Fig. 2F and is thus redundant. Instead of this bar graph, I think the authors need to show how Fig. 2F would look when different concentrations of EGF are used to activate Arf1-GTP. I would plot several curves in Fig. 2F, one for each [EGF]. Then, instead of Fig. 2E, the authors can

summarize the additional curves in Fig. 2F by plotting a dose-response curve: pulse height for each curve in Fig. 2F VS. [EGF].

**Response 7**: This is a multi-part question:

Part i: *Redundancy between 2E and 2F*: We agree that in the original submission, panels 2E and 2F were redundant items, i.e., both visualized quantifiable data. While 2E was dedicated to display quantification of immunoblots, 2F superimposed numerical simulation overlaid on experimental data for model fitting.

Part ii: "*need to show how Fig. 2F would look when different concentrations of EGF are used to activate Arf1-GTP*": Panel 2F in the original submission showed simulations with model fitting for Arf1 activity upon EGF stimulation. Although we can certainly plot numerical simulation data for varying concentrations of EGF, the limitations of the Arf1 activity assay (it is a rapid GST pulldown assay that sacrifices binding time only to preserve and capture as much GTP-bound GTPase that is possible before it rapidly hydrolyses the GTP to GDP) makes it difficult for us to detect Arf1 activity at lower levels of EGF stimulation due to a high threshold of detection in the assay. For example, we have confirmed that this assay is unable to detect steady-state Arf1 activity in 10% serum (even though we and others recognize that some activity must be there).

**Action(s) Taken**:

We have removed 2E and retained 2F (which is now **2C** in this revised submission). The figure legend has been updated to reflect this change and to improve clarity.

We have also added a statement in the "STUDY LIMITATIONS" section wherein we state that while some readouts were possible to conduct at varying concentrations of stimuli (e.g., cell survival and secretion, both validation studies to address model-driven predictions), others could not. For the convenience of the reviewer, a screenshot of this is pasted below.

loops); future work is expected to build upon this framework to fill these knowledge gaps. Conducting experiments across the full range of stimuli to assess 'proportionality/linearity' of response was possible in some instance (e.g., cell survival) but not possible in others (e.g., FRET, Arf1 activity, etc.) due to technical limitations of the assays and/or detection thresholds. Finally, our mathematical model ignores the effect of the physical

**Comment 8**: Related to points 6 & 7: I think the explanation for this experiment is too succinct and can only be understood by experts or readers who spend the time to read the cited paper #44. This experiment should be more fully explained. At the very minimum, explain what biological process the pull-down assay is trying to highlight.

**Response 8**: This comment is regarding Arf1 activity (**Fig 2A-C** in this version).

**Action(s) Taken**: In this revised submission, both text (results) and legend (**Fig 2B**) have been updated to inform the reader that the GST pulldown approach is an established assay for measuring fractional activity of Arf1 in cells.

**Comment 9**: Fig. 2G: while this image looks beautiful, why is it important to show the co-localization of Arf1 and GIV for understanding EGF's activation of Arf1? Help the readers by explaining this in the text in simple terms without jargon.

**Response 9**: Sorry for the confusion. This co-localization is important to verify the recruitment of GIV by active Arf1 on the Golgi (new Fig. 2D), which occurs after EGF-induced Arf1 activation. Furthermore, after this recruitment of GIV, GIV can activate Gi on the Golgi by working as GEF for Gi, and thus trigger downstream reactions.

**Action(s) Taken:** In the revision, we added **Fig. 2D** to illustrate the importance of the co-localization of Arf1 and GIV, and wrote the following text to explain the importance:

A key consequence of Arf1 activity within the coupled GTPase circuit is the first segment of the Gi activation pathway, i.e., the recruitment of GIV (**Figure 2D**), which is not only an effector of Arf1 but also the GEF of Gi (Lo et al., 2015). Previous studies showed that an evolutionarily conserved region in the N-terminal Hook domain of GIV can directly and preferentially bind to the active GTP-bound conformation of Arf1 (Lo et al., 2015), revealing the structure basis of the recruitment of GIV by active Arf1 (**Figure 1A**-*right*). To test whether GIV recruitment occurs in cells responding to EGF, we used immunofluorescence microscopy to observe HA-tagged Arf1 (green; **Figure 2E**) and endogenous GIV (red; **Figure 2E**). Membrane-colocalization of Arf1 and GIV was significantly increased within 5 min after EGF stimulation for serum-starved cells, as determined by a

7 | Page

quantification of the Arf1-positive Golgi regions using a Mander's overlap coefficient (MOC) (**Figure 2F**). These results indicate that EGF-induced Arf1 activity triggers the recruitment of GIV at the Golgi.

**Comment 10**: Fig 3 - FRET experiments: as with the experiment in Fig. 2F, this experiment seems to be done with one concentration of EGF. The concentration of EGF used should be listed (is the concentration "physiologically relevant"?). Why was this concentration chosen? Why is it not necessary to do a dosage-response curve of the type that I recommend for Fig. 2F?

**Response 10**: This is a 3-part comment:
Part i: "*concentration of EGF used should be listed*": The concentration of EGF used in all assays was constant (50 nM) and falls within the standard ranges used by others (detailed references are provided in our Response to **Comment 19**; see below).
Part ii: "*why was this concentration chosen*": There were two reasons. (i) To maintain continuity with our own prior studies [3-5] that used the same FRET-based assay with the exact same probes, in the same (HeLa) cell lines. (ii) To get enough signals when stimulating the endogenous receptor; for example, others stimulated the endogenous EGFR in HeLa cells using same concentrations (300 ng/ml or 50 nM [6]) to monitor Rac1 activity by FRET.

Part iii: "*Why is it not necessary to do a dosage-response curve*": We could not do these studies with a wide range of lower EGF concentrations because of the well-known limitations of the approach [7]. As outlined in our methods, computing the FRET efficiency requires a ratiometric calculation [8]. Because FRET efficiencies often range from 20 to 60% for biological reporters, the fluorescence emission from the acceptor fluorophore, when excited via energy transfer, usually provides lower signal strength than measurements of acceptor fluorophore emission resulting from direct excitation. Performing ratiometric calculations using relatively weak signals as the inputs can result in compounding error propagation [9]. Thus, while FRET, as a tool, offers the ability to probe molecular distances within images, it also introduces substantial limitations in sensitivity and variance, due to low signal strength.

**Action(s) Taken**:
Part i: Legend is updated with 50 nM concentration stated therein.
Part ii: Methods is updated with the information that justifies the FRET probes, cells, and conc of ligand stimulation.
Part iii: We have informed the reader with the addition of a statement in the "STUDY LIMITATIONS".

**Comment 11**: Figs. 3E, 3F, 3J, and 3K: the authors fit all these data to equations to get the curves shown here. It seems that the main purpose of these experiments is to extract some parameters that will go into the equations shown in Fig. 1B. If so, it's unclear what these parameters are and, therefore, what the main purpose of these experiments are. Right now, it just seems like these are just exercises in curve fitting without knowing the main purpose. Overall, the main message and purpose of Fig. 3 is obscured by many (impressive) experiments in Fig. 3.

**Response 11**: We apologize for the confusion that could have resulted from lack of clarity in our flow and presentation. The Fig.3E is FRET data based on Fig 3D. Fig.3F (Fig.3G in the revision) shows the model fitting results for Gi dynamics, where the model is introduced in Fig. 1B. The green dashed lines in Fig. 3J and 3K show the predicted Arf1 and Gi dynamics when the circuit is not coupled, respectively. For the uncoupled circuit, the simulated Arf1 activation dynamics capture the sustained activation of Arf1 dynamics in shGIV cells, and the simulated Gi dynamics is similar to (maybe even slightly increased than) that in control cells.

**Action(s) Taken**: To better illustrate Fig.3G in the revision, we rephrased corresponding texts the as follows:

We fit the above experiment data by tuning the kinetic parameters. We obtained a good fit for the fold change of Gi activation in both control and shGIV cells (**Figure 3G;** $R^2$ and RMSE, 0.54 and 0.41 for control cells; –0.44 and 0.71 for shGIV cells). The low level of GIV in shGIV cells was mimicked by decreasing the levels of

8 | Page

expression of GIV to 10% of that in control cells (**Figure 3C**). Thus, the model matched the overall trend of experiment data in both cells (see **Table EV1** for model parameters).

To better illustrate Fig.3J-K in the revision, we rephrased corresponding texts the as follows:

Finally, we used the model which was fitted to the experimental data in **Figure 2C** and **Figure 3G** to make predictions. We conducted two simulations: large decrease in the GIV level to simulate the Arf1 activation dynamics in shGIV cell (red line in **Figure 3J**), and delete either arrows 2 or 3 to simulate the Arf1 and Gi activation dynamics for the uncoupled GTPase switches. Based on the experimental results before (Lo et al., 2015), arrows 2 and 3 are modeled by an 'AND gate'-like digital logical operation (Kime and Mano, 2003), i.e., a HIGH output (ArfGAP2/3 activity, and resultant termination of Arf1 signaling) results only if both the inputs to the AND gate (arrows 2 and 3) are HIGH. We also tested the 'OR' logic for the negative feedback (**Figure EV3**) and found the model predictions to be indistinguishable from AND gate. It is possible that one of these logical modes of operation is more efficient than the other under certain circumstances. For the first simulation, the simulated Arf1 activation dynamics (red line in **Figure 3J**) captured the sustained activation of Arf1 dynamics in shGIV cells, indicating the efficiency of the model. For the second simulation, the simulated Arf1 dynamics (green line in **Figure 3J**) is the same as that in shGIV cells, suggesting the equivalency of deleting GIV and uncoupling GTPase switch. The simulated Gi dynamics (green line in **Figure 3K**) is similar to (maybe even slightly higher than) that in control cells, which is consistent with the fact that the feedback loops have no effect on Gi. Thus, negative feedback within the 'closed-loop control' exerts significant effect on the mGTPase (Arf1) and little or no effect on the tGTPase (Gi).

**Comment 12**: Fig. 4 - simulations: the authors state that these plots are from simulating a "EGF stimuli over a wide range of concentrations" (Pg. 8). But I'm confused because the experiments in Figs. 2-3 showed a result from a single concentration of EGF (please correct me if I'm wrong; I don't see any plots as a function of [EGF] in Figs. 2-3). This is another reason for repeating the key experiments in Figs. 2-3 with a wide range of EGF concentrations (see my points 7 & 10).

**Response 12**: This confusion arose from lack of clarity in our writing. As explained above in our responses to **Comments 7 and 10**, Arf1 and Gi activity assays have technical limitations, which impairs our ability to explore these two readouts in cells across a wide range of stimuli (we could only test 50 nM EGF vs 0.2% FBS, which represents growth factor restricted condition). Therefore, in **Figure 4A/B**, we used the data-fitted model parameters from the experiment with a single stimulus value to explore how the output mG* will vary over a wide range of stimuli.

**Action(s) Taken**: The specific word that was changed is shown below:

> Was: "*Therefore, by using the model that has been fitted to the data in **Figure 2C** and **Figure 3G**, we calculated the steady-state value of mG* and mGEF over a wide range of stimulus, and then plotted the fractional activation of mG* for a given mGEF activity to observe the linearity".*
>
> Changed to read*: "Therefore, by using the model that has been fitted to the data in **Figure 2C** and **Figure 3G**, we simulated the steady-state value of mG* and mGEF over a wide range of stimulus, and then plotted the fractional activation of mG* for a given mGEF activity to observe the linearity*."

**Comment 13**: Fig. 4 - simulations: there's not enough detail in the text to understand these simulations. At a bare minimum, the authors should describe what exactly is being simulated. The current description is couched in jargon. I don't really know what was varied, how many free parameters there are, what equations are used, and what the main purpose of this modeling is. The authors can give a full description of the model in the supplementary text. But in the main text, the authors should still give enough summary and details so that the reader can understand the main elements and purpose of the simulations without reading the supplementary text.

**Response 13**: We apologize for the lack of clarity. In Fig. 4, we compared the efficiency of information transduction between the coupled and uncoupled circuits, where the efficiency is measured by the dose-response alignment (DoRA) performance. DoRA is equivalent to the linear relation between the receptor dose-response curve and the dose-response curve for downstream molecules and supposed to improve information transduction. Here, by using the model after fitting, we calculate the dose-response curve for mGEF and mG* in coupled and uncoupled circuits. The linear relation between mGEF and mG* dose-response curves indicate the good information transduction in coupled switches.

**Action(s) Taken**: In the revision, we have rewritten the paragraph related to Fig 4, which is shown below

> To gain insights into how coupling impacts the information transduction, we compared the dose-response alignment (DoRA) performance between the coupled and uncoupled GTPase circuits. Typically, dose-response alignment (DoRA), referring to the close match of the receptor occupancy and the downstream molecules under different stimuli, is believed to improve information transduction, since the downstream molecules reflect the receptor occupancy faithfully. We regarded the mGEF as an alternative to the receptor because it serves as the first input to the coupled circuit via its ability to trigger the activation of the mGTPase switch. Therefore, a close match of dose-response curves of mGEF and mG* is equivalent to the linear relation between mGEF and mG*. Therefore, by using the model that has been fitted to the data in **Figure 2C** and **Figure 3G**, we simulated the steady-state value of mG* and mGEF over a wide range of stimulus, and then plotted the fractional activation of mG* for a given mGEF activity to observe the linearity. The misalignment in the case of a single switch is evident; a single Arf1 switch displays hyperresponsiveness, in that, max mG* is achieved even with minimal mGEF activity (**Figure 4A**). In the case of coupled switches, similar plots of fractional activation of mG* for a given mGEF activity show dose-response alignment with an unexpected linear relationship (**Figure 4B**). These results also hold in the presence of noise, such as noise in EGF stimulus and the intracellular noise [simulated within the concentrations of the different species (nodes) and the connections between them (arrows)] (see **Materials and Methods** and **Figure EV4**). These results suggest that coupled switches exhibit higher fidelity in information transduction than the uncoupled switches. Although unexpected for a GTPase switch, this finding is consistent with what is generally expected in a closed loop with negative feedback (Åström and Murray, 2021; Becskei and Serrano, 2000).

**Comment 14**: Fig. 5 - Protein-Protein Interaction network: I don't see how this figure and the analysis therein are relevant to the author's study. Given that all the functional assays in Figs. 3-4, why is the PPI analysis necessary? For what purpose? I think that the PPI analysis (Fig. 5) is distracting from the main storyline. It also doesn't help that one short paragraph describes this complicated topic (PPI analysis) in the main text, meaning that most readers won't understand what the result here is and means.

**Response 14**: We agree that the PPI analysis, as used in the original manuscript, was underdeveloped, and most importantly, not experimentally validated. However, the analysis and its conclusions were relevant because it provided us a rationale to connect the Golgi localized GTPase circuity to three cellular functions-- “secretion”, “sensing” and “cell number/survival”—all predicted to be impacted if the GTPases are uncoupled. We also acknowledge that the reviewer’s concerns are justified, in that, this is a complex computational analysis and may not be understood by most readers.

**Action(s) Taken**: To address these concerns, we have taken the following steps:

1) We have simplified the Figure panels. We have included a workflow diagram to narrate what was done (**Figure 4C**) and the analysis of network perturbation (**Figure 4D**). In simplifying the figure, legend, and the associated Results section, it was our intention to highlight the major take home message, i.e., coupling is predicted to connect secretion to sensing and survival (autocrine signaling).
2) We have added a new Figure (**Figure 7**) which validates the PPI network using TMT proteomics data from two different cell lines used in this work. These efforts revealed, with somewhat surprising consistency, that the nature of the signaling autonomy is primarily growth factor-centric, e.g., EGFR/EGF.

**Comment 15**: Fig. 6 - extracellular processes: this is the only part of the paper that deals with how the EGF affects cell proliferation. A major weakness of this part is that the simulations use different concentrations of EGF (e.g., Fig. 6B). Yet, the intracellular processes were studied with only a single concentration of EGF. Doing the experiments mentioned in my points 7 & 10 with various [EGF] would remedy this issue.

**Response 15**: This comment reflects our failure to clearly state which efforts are model fitting and which are model-simulated predictions. Figure 6 (which is **Figure 5** in the revised submission), is our attempt to use an experimentally constrained model (using just two EGF concentrations) to simulate secretion and cell number across a wide range of

stimulus/EGF. The predictions from these simulations were then validated in **Figure 7** using a wide range of stimulus (**Figure 6** in the revised submission).

**Action(s) Taken**: We made numerous edits to clarify that **Figure 5** is dedicated for model-derived prediction. These are listed below:

- In Figure 1B: We explicitly state what was experimentally constrained vs what was predictive module. A screenshot is placed below:
-

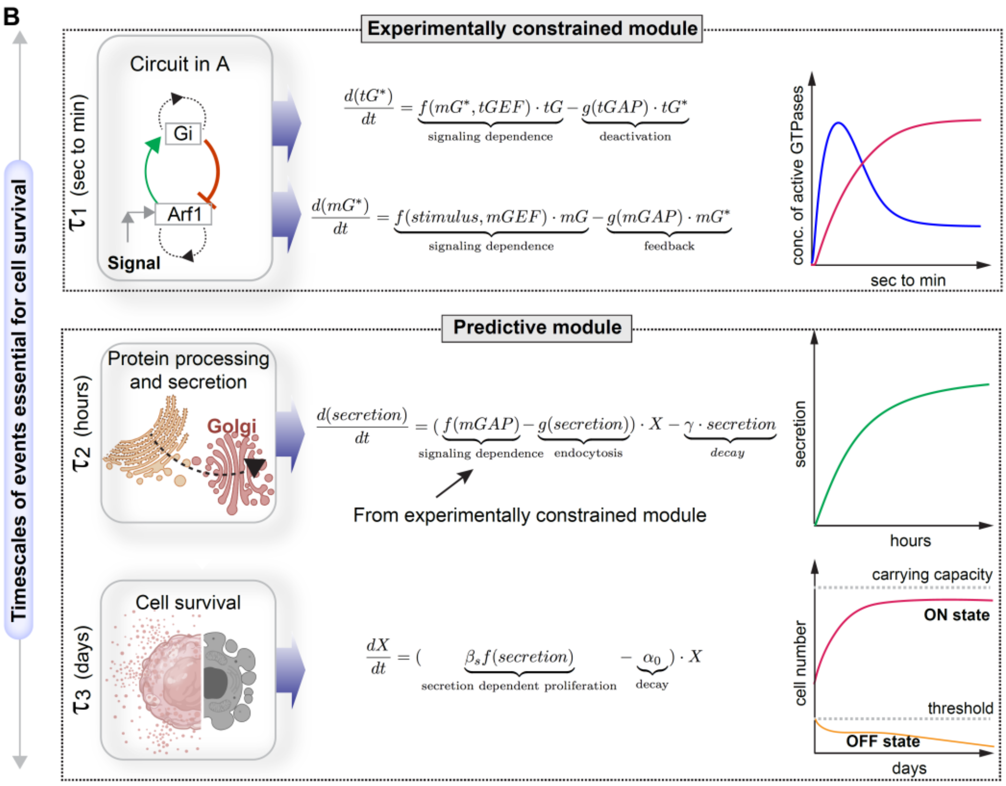


**Figure 1B**. Schematic of the dynamical systems model that we used to study the role of such coupling of GTPase (top panel) in autocrine secretion-supported cell survival and proliferation (bottom panel). The modeling in the top is experimentally constrained, and the modeling in the bottom is a predictive module. This model is based on the nominal time scale of these events (left panel) and has the typical behavior shown in the right panel.

- In results section: Text has been extensively re-written to clarify up front that secretion and survival modules are model predictions. A screen shot of the relevant text is presented below in our response to **Comment 16**.

**Comment 16**: Fig. 6 - extracellular processes: it's unclear how the results from Figs. 2-3 are linked to the extracellular processes and modeling described in Fig. 6 (i.e., how the secreted EGF controls cell number over time). The authors can remedy this by better explaining which parameter values in the equations in Fig. 6A come from which experiments.

**Response 16**: This question refers to **Figure 5** in the current version. The results in Fig. 2-3 are fitted to obtain the model for coupled GTPase switches (the first part in Figure 1B). The mGAP in this model affects the cell secretion, and the cell secretion regulates cell number through positive feedback. The kinetic parameters for secretion rate, cell production and cell decay are all from the biologically plausible range in [10].

**Action(s) Taken**: To better introduce the model in fig 5A, we rephrased the paragraphs related to fig 1B as follows:

The second part of the dynamical systems model is a predictive module for cell secretion and secretion-coupled cell survival (lower panel in **Figure 1B**). The coupling of this predictive module with the above experimentally constrained module is achieved by setting the secretion rate as a function of mGAP. The following findings allow us to make this coupling in the model: the finiteness of Arf1 activation-inactivation cycle was assumed to be a surrogate indicator of successful anterograde cargo movement through the compartments within the secretory pathway, i.e., the ERGIC (endoplasmic-reticulum–Golgi intermediate compartment) to the Golgi, because Arf1 regulates membrane traffic through a cycle of GTP binding and hydrolysis (Donaldson and Jackson, 2011); GTP binding is a pre-requisite for membrane curvature and vesicle formation (Beck et al., 2008) from the donor compartment, whereas GTP hydrolysis is a pre-requisite for vesicle uncoating (Tanigawa et al., 1993) and fusion with acceptor compartment. Therefore, we set the secretion rate as a function of GTP hydrolysis, a process regulated by mGAP. Except this setting for secretion rate, the model for cell secretion and cell survival/proliferation is similar to the model proposed by Hart *et al* (Hart et al., 2014), where the kinetic parameters are from biologically plausible ranges reported previously (Adler et al., 2018).

**Comment 17**: Fig. 7: what is the relevance of this experiment after the modeling in Fig. 6? This figure contains a huge number of panels and is more about how GTPase coupling by GIV is relevant for cell proliferation. But for the storyline, I think it's very jarring to have this come after Fig. 6 which explains how the intracellular components that you described in Figs. 2-3 lead to secretion of EGF. I recommend completely removing Fig. 7 or migrating it to the supplement.

**Response 17**: Figure 7 (which is **Figure 6** in the revised submission) is a central piece of the manuscript, in which we validate two major model-derived predictions (made in **Figure 5**), i.e., coupled GTPase circuit is critical for cell secretion (panels A-C) and secretion-coupled cell survival (panels D-K, in which we use inhibitor of Arf1/secretion as perturbagen).

**Action(s) Taken**: Exhaustive editing of text to clarify that these experimental data validate model-derived predictions.

**Comment 18**: Instead of the current Fig. 7, I recommend a diagram that connects all the different results in one picture as a final figure. For example, a pathway diagram that starts with EGF activating Arf1, and then ..... and finally, ending with EGF secretion.

**Response 18**: We appreciate this suggestion.
**Action(s) Taken**: After taking into consideration the suggestions of all reviewers, we have extensively revised the figure panels and reduced the number of schematics. We have used the SYNOPSIS IMAGE to depict what the reviewer suggested. It is our hope that the schematics that appear throughout the figure panels would go a long way to allow readers to understand key take home messages.

**Minor points:**

**Comment 19**: Pg. 6: "temporal finiteness" ---> "temporal fitness"? (what does either one mean?)
**Response 19**: We used the expression temporal finiteness to mean duration of signaling/activity. We agree that this expression is unnecessary.
**Action(s) Taken**: The words "temporal finiteness" has been removed in this revised submission. Instead of "*impact of coupling on the temporal finiteness of m/tGTPase signaling in response to input signal*" it now reads as "*impact of coupling on m/tGTPase signaling in response to input signal*"

**Comment 20**: Change title to be more specific to the work done here. The current title is too generic and not very informative.

**Response 20**: Thank you for the suggestion. In revising the title, we considered the suggestions of the other reviewers.

**Action(s) Taken**: The title has been revised to **"***A Circuit for Secretion-coupled Cellular Autonomy in Multicellular Eukaryotes***".** The abstract has also been updated to clarify that the type of autonomy is primarily self-sufficiency in growth factor signaling.

**Reviewer #3 Critiques**:

**General comments-R3**: The manuscript "A Eukaryotic Circuit for Secretion-coupled Cellular Autonomy" presents an integrated, interdisciplinary study of autocrine signaling. The team of authors is outstanding, and each component of the technical work appears to have been done well. This is very much the future direction of experimental biology, where mathematical thinking and computational models complement the experimental studies. The authors claim, "The insights and models derived from this study spur new paradigms in at least three fields, i.e., of signal transduction, cell secretion, and cancer cell biology in the following ways." Thus, their decision to send this article to a prestigious, high-impact journal for systems biology is reasonable.

However, after reading the manuscript, this Reviewer believes the authors are coming up short. The whole does not appear to be a complete, well-supported, scientific finding.

The figures are aesthetically beautiful, and the manuscript is written in a style common to systems biology.

**RESPONSE TO GENERAL COMMENTS**:

This reviewer's opinions of the quality and impact our work contrasts the opinions of the two other reviewers and the Editor (see above). Because we cannot rebut opinions, we have attempted to do our best in responding to the facts presented in his/her critiques. It is our hope that our responses (below) and the actions taken (in revising the manuscript) will mitigate some of the key concerns that were raised by the reviewer. We thank the reviewer for appreciating the effort that went into data visualization, writing and presentation.

**Major points:**

**Comment 1**: I completely respect their approach and their scientific prowess. This is a talented team of scientists, the team did each of the pieces of the study well, and they did each piece competently. However, it is not clear that the combined outcome of their effort result in a clear, coherent, well-justified set of conclusions. My opinion of the impact of this work and its ultimate influence is not in alignment with the authors claim of spurring new paradigms in three (or more!) fields.

**Response 1**: The reviewer is right in that the translational relevance of the fundamental discovery(ies) reported here is not appreciable in the current manuscript. While this work was in review, we have now extended our studies to accomplish two other studies:

1) First, we rigorously investigated the phenomenon of "Dose response alignment" using several model formulations of an experimentally validated circuit that couples two molecular switches—mGTPase (monomeric GTPase) and tGTPase (heterotrimeric GTPases) — with negative feedback loops. That work provides a framework for improving the DoRA performance in signaling motifs with negative feedback loops. This work is currently in revision at NPJ Systems Biology and is available on BioRxiv at https://www.biorxiv.org/content/10.1101/2022.06.14.496184v2
2) Second, we have made significant progress on the translational impact. Self-sufficiency in growth signaling, the earliest recognized hallmark of cancer [>64,000 Citations; Hananan and Weinberg, *Cell*; PMID: 21376230], is fueled by the tumor cell's ability to 'secrete-and-sense' growth factors; this translates into cell survival and proliferation that is self-sustained by auto-/paracrine secretion. Using breast cancer cells that are either endowed or impaired in growth signaling autonomy, we revealed how autonomy impacts cancer progression. Transcriptomic and proteomic studies show that autonomy is associated with self-sustained EGFR/ErbB signaling. A gene expression signature is derived (a.k.a., autonomy signature) which revealed that autonomy is induced in circulating tumor cells (CTCs) and particularly CTC clusters, the latter of which carry higher metastatic potential. Autonomy in CTCs tracks therapeutic response and prognosticates outcome. Autonomy is preserved during reversible (but not stable) epithelial-mesenchymal transition (EMT). These data support a role for growth signaling autonomy in multiple processes essential for the blood-borne dissemination of human breast cancer. This work is currently in revision at Journal of Clinical Investigation (JCI Insight) and is available on BioRxiv at: https://www.biorxiv.org/content/10.1101/2022.12.02.518910v1

**Action(s) Taken:** As per the reviewer's suggestions, and to avoid going beyond the evidence presented in the current work, we have updated the "discussion" section with edits/modifications that can accomplish three goals:

1) to **tone down the claim** by removing "spur new paradigms"
2) to **link the current work to the next chapters** and
3) to inform the readers of "**study limitations**".

We hope that these modifications now inform the reader more accurately of the impact as well as the caveats of the study.

**Comment 2**: The statement of significance opens with the statement "Self-sufficiency in growth signaling is one of the first hallmarks of cancer to be discovered and remains one of the least well understood." This statement does not ring

true to this Reviewer. Clearly there is room for opinion and disagreement. However, to begin the read with a questionable statement, and to then touch on only a sliver of the problem of self-sufficiency in growth signals in cancer suggests the authors' impression of the significance of the manuscript and is out of alignment with what they have. Self-sufficiency in growth signaling is a fundamental hallmark of cancer cell signaling, and the authors do not dissect how the circuit they study contributes to actual cancer phenotypes, other than some simple cancer cell line assays.

**Response 2**:
This is a **two-part** comment: **(i)** In the first part, the reviewer objects to a statement that we use in "Statement of Significance" section and questions the truth of the same. **(ii)** In the second part, the reviewer states that we do not really dissect cancer phenotypes in this work beyond 'simple cancer cell line assays'.

In response to Part i: The statement that "*self-sufficiency in growth signaling is one of the first of the six hallmarks to be discovered"* is taken from Hananan and Weinberg, *Cell*; PMID: 21376230. Cited >64,000 times so far, this article was quoted, but we could not include citation because "Statement of Significance" does not allow citations.

In response to Part ii: We agree that the current work did not address how the behavior or cancers may be impacted by the tumor cell properties (autocrine signal transduction, secretion, survival, proliferation) that were investigated here. As in most discoveries, we too were forced to make decisions regarding how best to package discoveries into focused and coherent chapters. In this instance, we struggled, as usually happens in transdisciplinary studies, to find ways to present our discoveries in discrete stand-alone publishable unit(s). While the current work represents the first (and the foundation) for this finding, we have now released in preprint the translational relevance of our findings. In continuity with the current studies, using breast cancer cells that are either endowed or impaired in growth signaling autonomy, we have revealed how autonomy impacts cancer progression. Transcriptomic and proteomic studies show that autonomy is associated with self-sustained EGFR/ErbB signaling. A gene expression signature is derived (a.k.a., autonomy signature) which revealed that autonomy is induced in circulating tumor cells (CTCs) and particularly CTC clusters, the latter of which carry higher metastatic potential. Autonomy in CTCs tracks therapeutic response and prognosticates outcome. Autonomy is preserved during reversible (but not stable) epithelial-mesenchymal transition (EMT). These data support a role for growth signaling autonomy in multiple processes essential for the blood-borne dissemination of human breast cancer. This work is currently in revision at Journal of Clinical Investigation (JCI Insight) and is available on BioRxiv at: https://www.biorxiv.org/content/10.1101/2022.12.02.518910v1

**Actions taken**: (i) The "statement of significance" has now been removed from this revised manuscript because it is not required for the Mol Sys Biology. (ii) In the "Discussion" section, we have now connected this work to the next chapters. For the convenience of the reviewer, we have copied and pasted that relevant section below.

> responses over a wide range of stimulus. In fact, follow-up work has now revealed how ranges of activity of the mGTPase Arf1, reaction kinetics, the negative feedback loop (mGAP), and the cascade length affect DoRA (Qiao et al, 2022).

> autonomy, adaptability, and flexibility. Follow-up work has now shed light on the importance of this phenomenon in the orchestration of self-sustained EGFR/ErbB signaling in tumor cells (Sinha et al, 2022). Such autonomy in growth signaling appears to be critical for the maintenance of high metastatic potential and epithelial mesenchymal plasticity during the blood-borne dissemination of human breast cancer.

**Comment 3**: This falls far short of proof in cancer. The systems analysis falls far short of what is needed/expected for systems biology. Due to the high degree of genetic and epigenetic variation between different cancer cell lines, a larger number of cell lines is typically required to show that a finding is not an artifact of a few cell lines - and to more conclusively suggest that the phenotype isn't a function of non-controlled genetic variables.

**Response 3**: This is a three-part comment, in which the reviewer points to the incompleteness of the current manuscript. We were confused by this statement because it not only contrasts those made by Reviewer #1 and 2 ("technically *impressive*"; "*admirable job*", but also his/her own statement above "*I completely respect their approach and their scientific prowess. This is a talented team of scientists, the team did each of the pieces of the study well, and they did each piece competently*." The reviewer also made another statement below (**Comment 8**) "*each component was well-executed*".

Part i: *"falls short of proof of cancer"*: We agree that the current manuscript **does not** address how the circuit/findings impact cancer progression; that was never our intent. In writing the manuscript, we remained disciplined and refrained from making any claims regarding the translational relevance of a pure fundamental paradigm. In the words of reviewer #1 we gave "*strong evidence of how cells can sense and respond to growth factors secreted by them and provides interesting ideas on how one could interfere with this process to, for instance, counteract cancer*" and the words of

reviewer #2, we believe that the major mechanistic aspect is "*systematically tying together various intracellular parts that control how a cell senses and secretes EGF*".

Having said that, and as highlighted in our **response #2** (see Part ii, above), during the review period, we have made progress in understanding the translational impact of our findings in cancer. More specifically, we gained insights into which step/where might growth signaling autonomy really be important in cancer initiation and progression. Our follow-up studies have revealed with surprising specificity that this step is most important for clustered circulating tumor cells that must colonize and create a new tumor during metastatic progression.

Part ii: "*systems analysis falls far short of what is needed/expected for systems biology*": We agree that the current manuscript **did not** go in-depth into each parameter space to interrogate the biological circuit in ways that is expected for systems biology. It was not our intention to do so, and we refrained from making claims in systems biology that were not explored here. As stated by Reviewer #2, the major goal that we accomplished in this manuscript was to use systems approached to *"combine different experiments, spanning vast time scales (hours to days) and spatial scales (organelle to multiple cells)".*

Having said that, and as highlighted in our **response #2** (see Part ii, above), during the review period, we have made progress by diving in-depth into the systems biology aspects of this work.

Part iii: "*number of cell lines is typically required to show that a finding is not an artifact of a few cell lines*". We thank the reviewer for this question. This work used 2 major cell lines, HeLa and MDA MB-231, manipulated with two different approaches, shRNA and CRISPR/Cas9 genetic editing approaches to uncouple the GTPase circuit. Both cell lines are work-horse models in cancer cell biology. HeLa provides continuity with prior body of work (by others and us) on GIV, G proteins, and Golgi secretory processes, whereas MDA MB-231 cells provide continuity with a body of literature (by others and us) on aggressive cancer cell phenotypes, GIV, G proteins, and EGF/EGFR-dependent cancers.

**Action(s) Taken**: The following two steps were taken:

To address parts i and ii, the "Discussion" section has been updated with statements that connect the current work with the next two chapters. See copied/pasted sections in our **Response #2** (above).

To address part iii, we favored going in-depth instead of superficial screen of a few more cell lines to distinguish artifacts from a fundamental shared cellular phenomenon. In this revised submission, we have now added a whole new figure (**Figure 7**) with proteomic analyses of both HeLa and MDA MB231 cell lines, -/+ coupled GTPases, grown in growth factor restricted conditions. We found that *both* cell lines require coupled GTPases for growth signaling autonomy. Most importantly, the efforts pinpointed with surprising specificity the precise nature of the self-sufficiency in growth factor signaling pathway (e.g., EGF/EGFR/Erbb) that is upheld by this cascade in *both* cell lines.

For the convenience of this reviewer, we have copied and pasted the new "Results" section from the revised manuscript.

**GTPase coupling supports self-sufficiency in growth factor signaling.**

To discern the nature of the pathway/processes whose autocrine autonomy is supported by the coupled GTPases, we analyzed HeLa and MDA MB-231 cells with coupled (WT) or uncoupled (GIV KO) circuits by tandem mass tag (TMT) proteomics. The studies were carried out in serum-free/restricted conditions (**Figure 7A**) to maximally enrich the proteome that supports auto-/paracrine secretion-coupled sensing. To our surprise, the majority (76%; 1437 proteins, includes EGFR; see complete list in **Dataset EV2**) of the differentially upregulated proteins (DEPs) in the two WT cell lines overlapped (despite the vast differences between HeLa and MDA MB-231 cell lines in origin, genetics, and nearly every other possible way). This suggests that the presence or absence of GTPase coupling via GIV may impact both cells similarly. The interactions between the DEPs were fetched from the STRING database to build a PPI network, which showed major coat proteins (AP1, AP2, COP, and CAV), monomeric GTPases (Arfs, Rabs, Rho, CDC42 and Rac1) and trimeric GTPases (GNAI) (**Figure 7B**). A degree of connectivity analysis revealed that EGFR and the Arfs are some of the most highly connected nodes in the interactome (**Figure 7C**). A reactome pathway enrichment analysis confirmed that the most highly connected proteins primarily engage in a variety of growth factor signaling pathways (**Figure 7D**).

Because protein functions are determined by subcellular localization, we sought to map the DEPs that are upregulated in the WT cells based on their subcellular localization. To this end, we used a Human Cell Atlas-supported explorer platform that was generated using the large collection of confocal microscopy images showing the subcellular localization patterns of human proteins to include in analysis three organelles, the Golgi, ER/ERGIC and the PM (**Figure 7E**; see **Materials and Methods**). Visualization of the DEP derived PPI network

13 | Page

as multi-layered networks that are comprised of intra- and inter-organelle interactions (**Figure 7F**) revealed greater insights. As expected, reactome pathway analysis of the ER/ERGIC and the Golgi interactomes showed an enrichment of protein processing and secretory processes, respectively, and the PM-localized interactome showed an enrichment of growth factor signaling (**Figure 7G**). The PM-localized interactome also showed an enrichment of cell-cell contact and contact-dependent signaling pathways (such as Semaphorins and the Eph-ephrin system; green; **Figure 7G**), which enable cell-cell coordination in multicellular eukaryotes. These findings indicate that the coupled GTPase system supports a network of proteins that primarily enable secretion-coupled growth factor sensing and thereby, growth signaling autonomy.

**Comment 4**: The issue of "proportionality" that is focused upon here - that the input/output relationship goes from looking hyperbolic to being stretched out so that (effectively) the linear part of the curve occurs where it would have otherwise been saturated is not a profound mathematical result. More importantly, the authors do not experimentally manipulate the cell between states to show that proportionality matters.

**Response 4**: This is a two-part comment. We thank the reviewer for bringing up these points.

Part i: "*the linear part of the curve occurs where it would have otherwise been saturated is not a profound mathematical result*": Indeed, the reviewer is right that the prediction is not a profound mathematical result but we note that the prediction that mGTPase and mGEF show dose response alignment when the switches are coupled is a finding that is specific to this network. This is elaborated in the companion paper [https://www.biorxiv.org/content/10.1101/2022.06.14.496184v2]. We are unsure what the reviewer means by this statement. We apologize that the lack in clarity in the way we presented the original manuscript may have contributed to this confusion. In this revised submission, we would like to draw attention to **Figure 4A** and **4B;** these panels show the impact of coupling on fractional activation of mGTPase (mG*; output) in response to mGEF (input stimuli). The finding that while the uncoupled state achieves hyperresponsive mGTPase activity (**4A**), the coupled state brings about a more linear 'proportionality' to response (**4B**), or DoRA. This, in our mind, was unexpected and novel, and is what the reviewer refers to as 'profound' mathematical / systems biology result.

Part ii: "*authors do not experimentally manipulate the cell between states*": Every experimental readout was carried out by manipulating the cells between two states (growth factor restricted and growth factor max. states). The reviewer is right in that we did not demonstrate 'proportionality' of response across multiple doses of stimuli in every experimental readout. This is simply because experimental readouts such as FRET, binding assays, or measures of interaction, signaling and secretion that use/require the use of immunoblots are fraught with threshold of detection, artifacts of supraphysiologic overexpressed constructs (FRET, pulldown assays) and noise (FRET). Consequently, in all these assays we were technologically limited to interrogate all ligand concentrations, and hence, resorted to studying the starved (0% or 0.2% serum, overnight) and stimulated states (10% FBS or 50 nM EGF).

We did, however, study two readouts (cell secretion and cell survival; see **Fig 6B-C** and **6D-I**) across a full range of stimuli because the available assay readouts allowed us to do so.

**Action(s) Taken**:
Part (i) is tackled by extensively revising the related manuscript:

To gain insights into how coupling impacts the information transduction, we compared the dose-response alignment (DoRA) performance between the coupled and uncoupled GTPase circuits. Typically, dose-response alignment (DoRA), referring to the close match of the receptor occupancy and the downstream molecules under different stimuli, is believed to improve information transduction, since the downstream molecules reflect the receptor occupancy faithfully. We regarded the mGEF as an alternative to the receptor because it serves as the first input to the coupled circuit via its ability to trigger the activation of the mGTPase switch. Therefore, a close match of dose-response curves of mGEF and mG* is equivalent to the linear relation between mGEF and mG*. Therefore, by using the model that has been fitted to the data in **Figure 2C** and **Figure 3G**, we simulated the steady-state value of mG* and mGEF over a wide range of stimulus, and then plotted the fractional activation of mG* for a given mGEF activity to observe the linearity. The misalignment in the case of a single switch is evident; a single Arf1 switch displays hyperresponsiveness, in that, max mG* is achieved even with minimal mGEF activity (**Figure 4A**). In the case of coupled switches, similar plots of fractional activation of mG* for a given mGEF activity show dose-response alignment with an unexpected linear relationship (**Figure 4B**). These results also hold in the presence of noise, such as noise in EGF stimulus and the intracellular noise [simulated within the concentrations of the different species (nodes) and the connections between them (arrows)] (see **Materials and Methods** and **Figure EV4**). These results suggest that coupled switches exhibit higher fidelity in information transduction than the uncoupled switches. Although unexpected for a GTPase switch, this finding is consistent with what is generally expected in a closed loop with negative feedback (Åström and Murray, 2021; Becskei and Serrano, 2000).

Part (ii) is tackled by addressing the issue of experimental limitations in the section "**Limitations of the study**" [which is in Discussion]. For the convenience of the reviewer, we have copied and pasted that relevant section below.

loops); future work is expected to build upon this framework to fill these knowledge gaps. Conducting experiments across the full range of stimuli to assess 'proportionality/linearity' of response was possible in some instance (e.g., cell survival) but not possible in others (e.g., FRET, Arf1 activity, etc.) due to technical limitations of the assays and/or detection thresholds. Finally, our mathematical model ignores the effect of the physical

**Comment 5**: The knock out / knock down of GIV is a good experiment - but it is exceptionally simple, and everything breaks down. More experiments are needed to justify conclusions at the level the authors.
Experiments to titrate the effect - overexpression, more modest knockdown, using specific mutants, etc. - would be required to test the ideas advanced by the author. Additionally, they could then get into whether or not there are significant "self-sufficiency in growth signals" consequences of the motif they describe.

**Response 5**: Four different modes of perturbation was used in this work.

- No ligand or serum
- With EGF ligand or full serum
- GIV depletion by
    - shRNA (HeLa)
    - CRISPR/Cas9 (HeLa and MDA MB231)
- Arf1 activity using BFA (Brefeldin A) [**Figure 6**].

**Actions taken**: In the revised submission, we have edited the "LIMITATIONS OF THE STUDY" section to inform the reader that more tools are essential before we can selectively disrupt each component/loop within the coupled GTPase circuit in cells. For the convenience of the reviewer, we have copied and pasted that relevant section below.

**LIMITATIONS OF THE STUDY**

The multi-timescale model we built, ignores the spatial aspects of the various feedback control loops. Because the spatial organization of signaling motifs will influence their temporal behaviors, we anticipate the need for further refinement of the current model. By depleting GIV, we disconnect the GTPases and dismantle the entire circuit; selective disruption of various connections within the Golgi-localized circuit is not possible currently due to the lack the experimental tools (e.g., specific point mutants of GIV, GEF or GAPs or perturbagens such as small molecule or peptides. Although we studied four different cargo proteins (VSV-G, MMP2/9 and Col-VII) and

**Comment 6**: The authors make a statement that more experiments are not possible in their manuscript. That does not seem accurate. Additionally, scientists sometimes pursue studies where the currently available techniques and reagents to advance the study are not available and where the project must be put on hold. Such a wall is not a justification for publishing an incomplete study, and is not a foundation for changing paradigms in three (or more!) fields.

**Response 6**: We respect the opinion of this reviewer. However, we could not find—in the original or this revised version of the manuscript—anything stating that "more experiments are not possible". We believe that this reviewer may have paraphrased what we declared as "Study Limitations" [section pasted immediately above as part of **Response 5**]

**Actions taken**: In the revised submission, we have edited the "LIMITATIONS OF THE STUDY" section to inform the reader why some studies could not be carried out at this time. The relevant section is copied and pasted in our response to **Comment 5** (above).

**Comment 7**: In Results Section 4 when discussing disrupting the negative feedback loops, the fact that disabling either loop is equivalent is not surprising, they are mathematically equivalent by the structure of the model (i.e. setting either term f2(tGEF)*f3(tG*) to zero is equivalent) as mentioned in the supplement.

**Response 7**: The reviewer is correct that the two negative feedback loops in the model, are inherently coupled and therefore, setting either term to zero turns off all feedback. This approach to model building was deliberate to ensure that the experimentally observed interactions could be captured faithfully in the model. If we changed the form from f2(tGEF)*f3(tG*) to f2(tGEF)+f3(tG*), i.e., from AND to OR logic, the modeling fitting results are similar (**Figure EV3**), as well as the information transduction efficiency [11].

**Action(s) Taken**: To better illustrate model predictions, we rephased the corresponding paragraph as follows:

> Finally, we used the model which was fitted to the experimental data in **Figure 2C** and **Figure 3G** to make predictions. We conducted two simulations: large decrease in the GIV level to simulate the Arf1 activation dynamics in shGIV cell (red line in **Figure 3J**), and delete either arrows 2 or 3 to simulate the Arf1 and Gi activation dynamics for the uncoupled GTPase switches. Based on the experimental results before (Lo et al., 2015), arrows 2 and 3 are modeled by an 'AND gate'-like digital logical operation (Kime and Mano, 2003), i.e., a HIGH output (ArfGAP2/3 activity, and resultant termination of Arf1 signaling) results only if both the inputs to the AND gate (arrows 2 and 3) are HIGH. We also tested the 'OR' logic for the negative feedback (**Figure EV3**) and found the model predictions to be indistinguishable from AND gate. It is possible that one of these logical modes of operation is more efficient than the other under certain circumstances. For the first simulation, the simulated Arf1 activation dynamics (red line in **Figure 3J**) captured the sustained activation of Arf1 dynamics in shGIV cells, indicating the efficiency of the model. For the second simulation, the simulated Arf1 dynamics (green line in **Figure 3J**) is the same as that in shGIV cells, suggesting the equivalency of deleting GIV and uncoupling GTPase switch. The simulated Gi dynamics (green line in **Figure 3K**) is similar to (maybe even slightly higher than) that in control cells, which is consistent with the fact that the feedback loops have no effect on Gi. Thus, negative feedback within the 'closed-loop control' exerts significant effect on the mGTPase (Arf1) and little or no effect on the tGTPase (Gi).

Last, but not least, in a manuscript (on PrePRINT: https://www.biorxiv.org/content/10.1101/2022.06.14.496184v2), we have investigated in depth how AND vs OR gate may change the properties of the network motif. We cited this work in Discussion.

**Comment 8**: Studies like this are a major undertaking, and sometimes the needed experiments are more work than can be justified. These types of projects also have a higher-risk element than a "measure lots of data" type systems biology project, which will by definition almost always result in a large volume of data - and for which the meaning of that data is unknown and also is typically presented in a speculative manner. However, for this type of systems biology - a more concerted effort to test the circuit studied experimentally is needed. I empathize with this talented group of scientists - a project was pursued that seemed promising, each component was well-executed, but as of now- it feels like it has come up short.

**Response 8**: We respect the opinion of this expert reviewer and appreciate the generous words he/she used in appreciation of such high-risk transdisciplinary work (highlighted above). This comment ["*comes short*"] is a repetition of the sentiments that are in **Comment #3** from this reviewer. We ourselves struggled to find a good way to break up a continuum of findings within a major program/project(s) into publishable units that can be peer-reviewed by reviewers despite crossing numerous disciplines. We acknowledge that the current manuscript does not answer several questions. As stated above in our **Response 1**, we have made progress in findings answers to some of those questions.

**Action(s) Taken**: To avoid redundancy, we request the reviewer to please refer to our **Response 1, 2** and **3** (above). We would also request the reviewer to kindly refer to our **Response 17** (below) where we state additional experimental findings that go above and beyond what was asked by either reviewers or the Editor. In deciding to conduct and include these experiments, and not others (that were deemed better suited as stand-alone manuscripts), we exercised our judgement of what is the central claim of this work and whether it is supported by sufficient rigor. We hope the reviewer agrees that the team has demonstrated its intention to follow-up the important claims/findings.

**Minor points:**

**Comment 9**: The authors should clarify which results are "predictions" of the model and which results the model is fit to.

**Response 9**: The reviewer brings up a very important point and reflects the lack of explicit statements and clarity in the original submission. This comment is a part of a detailed comment later in this document from the same reviewer (**Comment 11**).

**Action(s) Taken**: We have responded to this comment and stated corrective actions in our **Response 13.** To avoid redundancy, we request the reviewer to please refer to our **Response 13** (below), where we have copied and pasted the relevant sections that were extensively edited to state what was done.

**Comment 10**: The authors should update their figures to better show their anticipated causality from stimulus through measured phenotypes so it does not require readers to dig into the text and understand the biological system in depth - if it's a circuit and well-described by a simple model, it should be possible to communicate this simply instead of only with artistic, but complicated, figures.

**Response 10**: We agree. All 3 reviewers unanimously agree that we needed to reorganize figure panels, remove redundancy and smoothen the flow of the paper to allow readers to appreciate the findings despite the transdisciplinary nature of the work.

**Action(s) Taken**: In the revised submission, we have now exhaustively rewritten the manuscript and reorganized the figure panels to first narrate the experiments (**Figures 2 and 3**) that were used for ‘model fitting’. The new Figure2A, Figure 2D, Figure 3A, and Figure 3H display the schematic of four key biochemical reactions in the coupled GTPase switches, with follow-up panels showing experiment validations. Later in the manuscript, we use the model to rationalize experiments and validate the predictions (**Figure 6**).

**Comment 11**: The colors for the symbols representing tG and tG* in the legend of Fig S1B do not seem to match

**Response 11**: We thank the reviewer for his/her keen eyes and attention to this detail (which we had missed). In the original submission, tG was annotated with a deep cerulean blue circle in the legend/Key, but with a green circle in Figure S1B.

**Actions taken**: In the revised submission, this color mismatch issue was rectified in the panel (below). This figure is now EV1 in the revised submission. We have copied and pasted it below for the convenience of the reviewer.

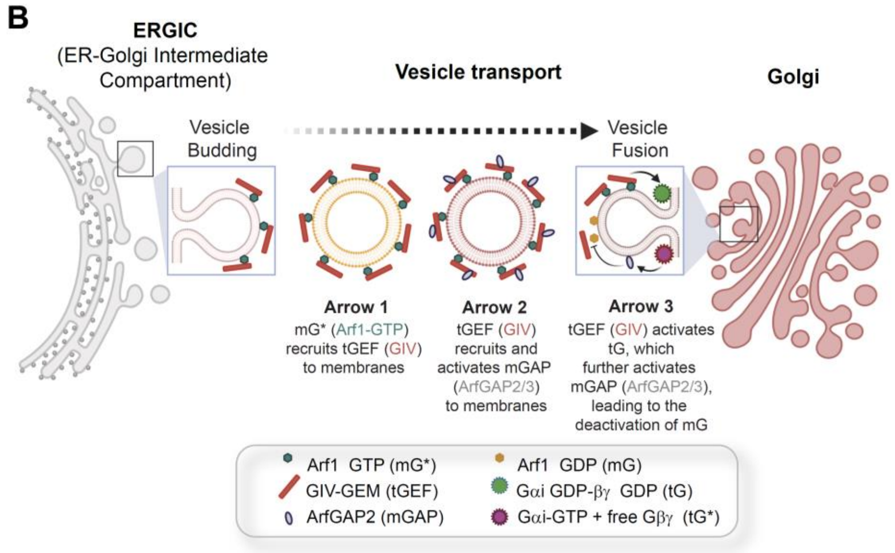


**Comment 12**: What is the NRMSE value for shGIV model "prediction" in Fig 3E being compared to?

**Response 12**: Sorry for the confusion. This value is compared to the fold change of FRET data in shGIV cells.

**Action(s) Taken**: We revised Fig 3E (now it is **Fig 3G** in the revision). In the new Fig 3G, the NRMSE value for shGIV model is compared to the fold change of tG* FRET data in shGIV cells (error bars in red).

**Comment 13**: It seems as though the pulldown timecourse data from Fig 2D-E is being used here to fit just the Arf1 module or the "single-switch" model as it is referred to in the supplement. First off, it is unclear why control HeLa cells would be used to fit the single-switch or uncoupled model as, presumably, this coupling would be intact in the cells unless perturbed. Second, based on the supplementary table, it seems as though all the parameters were fit here not just the parameters for Arf1 activity. But then, in Results Section 3: "These experimentally observed dynamics of tGTPase activation at the Golgi (OUTPUT #2) in response to EGF (INPUT) in control cells matched model predictions with excellent fitness ($R^2$ and normalized RMSE are 0.54 and 0.41 respectively; see Supplementary Table 1 for model parameters)." Compared to Results Section 1: "The kinetic parameters of the coupled GTPases module were subsequently tuned to fit the time course data of GTPases in control cells and GIV-depleted cells." And the supplement: "Fitting against experimental data: To fit the time course data for control cells and GIV-depleted cells, we manually tuned the parameters in our model until the normalized RMSE between simulated and measured fold changes of active Arf1 was less than 0.2 and that for active tGTPase less than 0.45." As written, it is completely unclear from these sections whether the model output of tG* activation in response to stimulus is a "prediction" of the model or the parameters were tuned to fit the data in Figure 3D-E. If the parameters were in fact tuned to fit the timecourse tG* data, then the wording in the Results section and in Figure 3E must be changed to reflect that the model was fit to data and not making a prediction. This has implications for the results in Figure 3I-K, again it needs to be clarified if the model is fit to this data or is, say, fit to data in Figure 2D-E and Figure 3E, then predicts behaviors in Fig J-K when the negative feedback loops are removed.

**Response 13**: This is a two-part comment, both related to confusions surrounding what experimental data was part of 'model fitting' vs what experimental data was part of validation of 'model predictions'. We apologize for the lack of clarity in our original manuscript.

Part i: "*unclear why control HeLa cells would be used to fit the single-switch or uncoupled model as, presumably, this coupling would be intact in the cells unless perturbed*"
The reviewer is right in that the model fitting was indeed done using experimental data from both control and GIV-depleted HeLa cells. The latter was expected to capture the impact of perturbing the coupled GTPase circuit.

Part ii: "*it is completely unclear from these sections whether the model output of tG* activation in response to stimulus is a "prediction" of the model or the parameters were tuned to fit the data in Figure 3D-E*."
The parameters were tuned to fit the time course Arf1 and tG* data, and thus the wording related to Fig 2D-E (now it is **Fig 2C**) and Fig 3E (now it is **Fig 3G**) should be "model fitting". The behaviors without feedback (Fig 3J-K) and the linearity between mGEF and mG* (Fig 4A-B) were "model prediction".

**Action(s) Taken**: In the revised submission, the text related to Fig 3E (now it is **Fig 3G**) was changed to:

We fit the above experiment data by tuning the kinetic parameters. We obtained a good fit for the fold change of Gi activation in both control and shGIV cells (**Figure 3G;** $R^2$ and RMSE, 0.54 and 0.41 for control cells; −0.44 and 0.71 for shGIV cells). The low level of GIV in shGIV cells was mimicked by decreasing the levels of

8 | Page

expression of GIV to 10% of that in control cells (**Figure 3C**). Thus, the model matched the overall trend of experiment data in both cells (see **Table EV1** for model parameters).

Besides, the text related to Fig 3J-K (now it is **Fig 3J**) was changed to:

Finally, we used the model which was fitted to the experimental data in **Figure 2C** and **Figure 3G** to make predictions. We conducted two simulations: large decrease in the GIV level to simulate the Arf1 activation dynamics in shGIV cell (red line in **Figure 3J**), and delete either arrows 2 or 3 to simulate the Arf1 and Gi activation dynamics for the uncoupled GTPase switches. Based on the experimental results before (Lo et al., 2015), arrows 2 and 3 are modeled by an 'AND gate'-like digital logical operation (Kime and Mano, 2003), i.e., a HIGH output (ArfGAP2/3 activity, and resultant termination of Arf1 signaling) results only if both the inputs to the AND gate (arrows 2 and 3) are HIGH. We also tested the 'OR' logic for the negative feedback (**Figure EV3**) and found the model predictions to be indistinguishable from AND gate. It is possible that one of these logical modes of operation is more efficient than the other under certain circumstances. For the first simulation, the simulated Arf1 activation dynamics (red line in **Figure 3J**) captured the sustained activation of Arf1 dynamics in shGIV cells, indicating the efficiency of the model. For the second simulation, the simulated Arf1 dynamics (green line in **Figure 3J**) is the same as that in shGIV cells, suggesting the equivalency of deleting GIV and uncoupling GTPase switch. The simulated Gi dynamics (green line in **Figure 3K**) is similar to (maybe even slightly higher than) that in control cells, which is consistent with the fact that the feedback loops have no effect on Gi. Thus, negative feedback within the 'closed-loop control' exerts significant effect on the mGTPase (Arf1) and little or no effect on the tGTPase (Gi).

**Comment 14**: The phrase in the Discussion: "The insights and models derived from this study spur new paradigms in at least three fields" is overly strong. This goes beyond rephrasing; the authors should complete this study and describe what they have shown vs. speculate what this work might ultimately be a part of.

**Response 14**: This comment is a repetition of **Comment 1** of this reviewer.

**Actions taken**: We have responded to this comment and stated corrective actions in our **Response 1.** To avoid redundancy, we request the reviewer to please refer to our **Response 1**.

**Comment 15**: The choice of experimental system (cell lines) and which lines is rather limited for a study claiming to have impact on a major cancer hallmark: self-sufficiency in growth signals Additional cell lines would help show generality, and show that the result is not an artifact of the specific genetic profile/cancer phenotype of the cells utilized.

**Response 15**: This comment is a repetition of Part iii in **Comment 3** of this reviewer.

**Actions taken**: We have responded to this comment and stated corrective actions in our Response 3/Part iii (above). To avoid redundancy, we request to please refer to our **Response 3**/**Part iii**.

In addition to the aforementioned actions, the revised manuscript includes a detailed paragraph (at the very beginning of Results section) rationalizing the use of each cell line.

Two different cancer cell lines were chosen to conduct experiments, the HeLa cervical cancer and the MDA-MB231 breast cancer cells. Our choice was guided by two reasons: *(i)* HeLa cells not only represent the most robust system to study Golgi structure (Ayala and Colanzi, 2016; Wortzel et al., 2017) and function (Rauter et al., 2020), but also provide continuity with prior work because all biophysical and functional studies that led to the discovery of the coupled GTPases at the Golgi were performed in this model; *(ii)* we and others have shown that transcriptional upregulation or post-transcriptional activation (Bhandari et al., 2015; Dunkel et al., 2012; Sasaki et al., 2015) of GIV (the 'linker' between the two GTPases; **Figure 1A**) supports several aggressive tumor

6 | Page

cell properties (of which, many were demonstrated in MDA-MB231 cells (Jiang et al., 2008; Lopez-Sanchez et al., 2015; Midde et al., 2018; Rahman-Zaman et al., 2018; Rohena et al., 2020; Wang et al., 2015; Wang et al., 2017)), including, invasion, matrix degradation, proliferation and survival (Aznar et al., 2016; Garcia-Marcos et al., 2015). Elevated expression of GIV has also been reported in a variety of solid tumors (Garcia-Marcos et al., 2015; Getz et al., 2019), both in primary tumors (Ghosh, 2015; Ghosh et al., 2016b) as well as in circulating tumor cells (Barbazan et al., 2016; Dunkel et al., 2018) have been shown to correlate with tumor aggressiveness and poor survival across cancers. Finally, model and PPI network-driven predictions of uncoupling the GTPases or interrupting secrete-and-sense autonomy were experimentally validated in the two cancer cell lines that lack GTPase coupling in the absence of the GIV linker protein.

**Comment 16**: It seems that in the model secretion is a function of mGAP and not mG* and mG. Could this be better explained?

**Response 16**: We apologize for the lack of clarity.
**Action(s) Taken**: In this revised submission, we have stated the rationale for choosing mGAP over mG* and mG. For the convenience of the reviewer, we have copied and pasted that section below:

The second part of the dynamical systems model is a predictive module for cell secretion and secretion-coupled cell survival (lower panel in **Figure 1B**). The coupling of this predictive module with the above experimentally constrained module is achieved by setting the secretion rate as a function of mGAP. The following findings allow us to make this coupling in the model: the finiteness of Arf1 activation-inactivation cycle was assumed to be a surrogate indicator of successful anterograde cargo movement through the compartments within the secretory pathway, i.e., the ERGIC (endoplasmic-reticulum–Golgi intermediate compartment) to the Golgi, because Arf1 regulates membrane traffic through a cycle of GTP binding and hydrolysis (Donaldson and Jackson, 2011); GTP binding is a pre-requisite for membrane curvature and vesicle formation (Beck et al., 2008) from the donor compartment, whereas GTP hydrolysis is a pre-requisite for vesicle uncoating (Tanigawa et al., 1993) and fusion with acceptor compartment. Therefore, we set the secretion rate as a function of GTP hydrolysis, a process regulated by mGAP. Except this setting for secretion rate, the model for cell secretion and cell survival/proliferation is similar to the model proposed by Hart *et al* (Hart et al., 2014), where the kinetic parameters are from biologically plausible ranges reported previously (Adler et al., 2018).

**Comment 17**: The PPI network analysis - it's not clear what this contributes that has significance. It feels like filler.

**Response 17**: We agree that the original version of this work used the findings of our PPI analysis as a rationale for prioritizing the two most important readouts across longer time scales (i.e., cell secretion and survival/homeostasis). As presented earlier, these PPI network-based predictions were validated by experiments (showcased in **Fig 6**), but not with actual quantitative proteomics and subsequent analysis of such dataset

**Actions taken**: In this revised submission, we have now added an in-depth quantitative TMT proteomics analysis that we conducted in both HeLa and the MDA MB-231 cell lines (coupled vs uncoupled states). Differentially expressed

proteins in both cell types pinpointed the role of the coupled GTPase circuit in maintaining self-sufficiency in growth factor (e.g., EGF/EGFR, VEGF and TGF) signaling (**Fig 7A-D**). An organelle specific sub-analysis of the PPI network showed that the differentially expressed proteins connect secretory functions of the Golgi to PM-localized sensing of diverse growth factor pathways and the processes they control (**Fig 7E-G**). These findings are now presented in **Figure 7**. We have accordingly modified the "Results" Section and the Figure legends.

## REFERENCES CITED IN THIS RESPONSE DOCUMENT


1. Harper, S., and Speicher, D.W. (2011). Purification of Proteins Fused to Glutathione S-Transferase. In Protein Chromatography: Methods and Protocols, D. Walls, and S.T. Loughran, eds. (Humana Press), pp. 259-280. 10.1007/978-1-60761-913-0_14.
2. Zuber, C., Spiro, M.J., Guhl, B., Spiro, R.G., and Roth, J. (2000). Golgi Apparatus Immunolocalization of Endomannosidase Suggests Post-Endoplasmic Reticulum Glucose Trimming: Implications for Quality Control. Molecular Biology of the Cell *11*, 4227-4240. 10.1091/mbc.11.12.4227.
3. Kalogriopoulos, N.A., Lopez-Sanchez, I., Lin, C., Ngo, T., Midde, K.K., Roy, S., Aznar, N., Murray, F., Garcia-Marcos, M., Kufareva, I., et al. (2020). Receptor tyrosine kinases activate heterotrimeric G proteins via phosphorylation within the interdomain cleft of Gαi. Proceedings of the National Academy of Sciences *117*, 28763-28774. 10.1073/pnas.2004699117.
4. Ma, G.S., Aznar, N., Kalogriopoulos, N., Midde, K.K., Lopez-Sanchez, I., Sato, E., Dunkel, Y., Gallo, R.L., and Ghosh, P. (2015). Therapeutic effects of cell-permeant peptides that activate G proteins downstream of growth factors. Proceedings of the National Academy of Sciences *112*, E2602-E2610.
5. Midde, K.K., Aznar, N., Laederich, M.B., Ma, G.S., Kunkel, M.T., Newton, A.C., and Ghosh, P. (2015). Multimodular biosensors reveal a novel platform for activation of G proteins by growth factor receptors. Proceedings of the National Academy of Sciences *112*, E937-E946.
6. Wang, H., Yang, C., Leskow, F.C., Sun, J., Canagarajah, B., Hurley, J.H., and Kazanietz, M.G. (2006). Phospholipase Cγ/diacylglycerol-dependent activation of β2-chimaerin restricts EGF-induced Rac signaling. The EMBO Journal *25*, 2062-2074. https://doi.org/10.1038/sj.emboj.7601098.
7. Leavesley, S.J., and Rich, T.C. (2014). FRET: Signals hidden within the noise. Cytometry Part A *85*, 918-920. https://doi.org/10.1002/cyto.a.22568.
8. Szöllősi, J., Damjanovich, S., Nagy, P., Vereb, G., and Mátyus, L. (2006). Principles of Resonance Energy Transfer. Current Protocols in Cytometry *38*, 1.12.11-11.12.16. https://doi.org/10.1002/0471142956.cy0112s38.
9. Berney, C., and Danuser, G. (2003). FRET or No FRET: A Quantitative Comparison. Biophysical Journal *84*, 3992-4010. https://doi.org/10.1016/S0006-3495(03)75126-1.
10. Adler, M., Mayo, A., Zhou, X., Franklin, R.A., Jacox, J.B., Medzhitov, R., and Alon, U. (2018). Endocytosis as a stabilizing mechanism for tissue homeostasis. Proceedings of the National Academy of Sciences *115*, E1926. 10.1073/pnas.1714377115.
11. Qiao, L., Ghosh, P., and Rangamani, P. (2022). Design principles of dose-response alignment in coupled GTPase switches. bioRxiv, 2022.2006.2014.496184. 10.1101/2022.06.14.496184.

Thank you for sending us your revised manuscript. We have now heard back from the three referees who agreed to evaluate your study. From the comments below, you will see that Referees #1 and #2 are satisfied with the revisions and support the publication of the manuscript. We note that Reviewer #3 still raised a number of issues regarding the experimental validations. During our pre-decision cross-commenting process (in which the referees are given a chance to make additional comments, including on each other's reports), Referees #1 and #2 explicitly indicated that they think the manuscript has done a fair job of stating the limitations of the model and experiments, and they support the publication of the revised manuscript. Therefore, given the balance of these evaluations, we feel that we can consider a revision of your manuscript.

Please address the following issues:

1. Address the minor issues raised by Referee #1.

On a more editorial level:

----------------------------------------------------------------------------

Reviewer #1:

I liked this paper already at first submission and I can only say that I like it even more now. Despite having already performed an incredible number of experiments, the authors added a new data set showing mass spectrometric analysis of PPIs in the presence and absence of the coupling between the two GTPase switches in two cell lines.
The paper is truly massive, which might represent a problem for some readers, who might be overwhelmed by so such information!
It was a good decision to eliminate some of the old supplementary data and also to streamline the figures a bit.
I definitely opt for publication of this excellent paper in Molecular Systems Biology and congratulate the authors warmly.

I do have several points concerning language and few points on the figures and the data presentation. I will start with data presentation and figures:

1. In figure 1A, I suggest to swap tGEF and GIV so that GIV is above and tGEF below in squared brackets. All in all, Arf1 and Galphai, that is, the name of the protein, is written above, and their molecular identity (GTPase) is written below in squared brackets. I would keep the same format. There are some other panels in main and supplementary figures where this nomenclature appears as well, it might be good to unify them.
2. Figure 2E, for the 3D plots, I noticed only now that there is some sort of messy white blob on top on the Y-axis below 0. I assume these are numbers just are simply so close together that they become unreadable and look like that. Perhaps there is a way to cut the plots so that this effect is not there anymore.
3. I know that the paper is already full of data and figures. Nonetheless, I believe it is common practice nowadays to show in the supplement the full membranes used for the WB that are shown cropped in the main text. I would also like to see the Poinceau stained membrane in full, given the explanation the authors gave me as why this is better than detection via anti-GST antibody. It is a fair point, but the authors so far show only a small portion of the membrane, thus the reader cannot judge if there are truncated bands etc.
4. Figure 6E: for consistency I would either write apoptosis / necrosis, or apoptotic cells / necrotic cells. At the moment the authors use a hybrid: apoptosis / necrotic cells

About language.
Since the authors changed a large portion of the text, I found several places in which I believe the language could be improved:

1. Abstract: growth factors and stimuli are plural nouns, thus the "a" should not be there in the sentence: growth factors are key stimuli"
2. Introduction, very first sentence. I would add a comma after autonomy and I would write "to have been clearly defined" instead of "to be clearly defined"
3. I find the terminology "membrane of extracellular space" odd. The extracellular space has no membrane, but "of" indicates the membrane belongs to it. How about writing simply "rooted to the cell membrane"?
4. In this sentence, the verb should be plural: "the secretory functions of the Golgi apparatus require the unlikely coupling"
5. Right after, the article is missing: "one is the small or monomeric (m) GTPase Arf1". When writing the name of the protein I would not use the article, e.g. Arf1 is a small GTPase, but when writing the type of protein followed by the name I would use the article. Please correct this throughout the text
6. I find this sentence confusing: "One negative feedback loop is that GIV can improve the GAP for Arf1---ArfGAP2/3, thus terminating Arf1 signaling", I suggest to re-phrase like this: "One negative feedback loop involves the activation of the GAP for Arf1 (ArfGAP2/3) by GIV, which terminates Arf1 signaling"
7. I personally do not like this title: "An integrated systems and experimental approach to dissect a Golgi-localized GTPase circuit" because one speaks of systems biology but not systems approach. How about "An integrated computational and experimental approach to dissect a Golgi-localized GTPase circuit"?
8. Same here, I do not like the terminology "a dynamical systems model". "A mathematical model based on ODEs" would be better.
I would write like this: "We chose two different cancer cell lines to conduct the experiments: cervical (HeLa) and breast (MDA-MB231) cancer cell lines"
9. There is something strange in this sentence: "Elevated expression of GIV has also been reported in a variety of solid tumors (Garcia-Marcos et al., 2015; Getz et al., 2019), both in primary tumors (Ghosh, 2015; Ghosh et al., 2016b) as well as in circulating tumor cells (Barbazan et al., 2016; Dunkel et al., 2018) have been shown to correlate withtumor aggressiveness and poor survival across cancers." Perhaps simply an "and" and the verb in the singular form would do the job: "elevated expression ...has been reported.... and has been shown to...".
10. The paragraph starting with: "Finally, model and PPI network-driven predictions" seems to be separated from the previous paragraph, in which an explanation is given about why those two cell lines were chosen. I would put it in a new paragraph.
11. "The Epidermal growth factor (EGF) was prioritized" : the article was missing
12. "revealing the structural basis of the recruitment of GIV by active Arf1" not the structure basis
13. „GIV-depleted cells were obtained using a short hairpin RNA to target GIV". From the methods I gather it is only one RNA, so

add the article "a"
14. Re-phrase like this: "10% serum (containing a mixture of growth factors)"
15. Add the article: "The findings confirmed that Gi is activated on ..."
16. Change to this: "We fitted the above experimental data by tuning the kinetic parameters"
17. Change to this: "Thus, the model matched the overall trend of the experimental data in both cells"
18. I would re-phrase like this: " We conducted two simulations: one in which we decreased the GIV level to simulate the Arf1 activation dynamics in the shGIV cell line (red line in Figure 3J), and one in which we deleted either arrows 2 or 3 to simulate the Arf1 and Gi activation dynamics for the uncoupled GTPase switches."
19. "We also tested the 'OR' logic for the negative feedback (Figure EV3) and found the model predictions to be indistinguishable from those obtained with the AND gate."
20. I personally would not talk of "efficiency" of the model, but rather the ability of the model to capture the experimental data
21. I do not understand this: "Typically, dose response alignment (DoRA), referring to the close match of the receptor occupancy and the downstream molecules under different stimuli"... please re-phrase to explain better
22. I would delete the therefore from this sentence: " Therefore, by using the model that has been fitted to the data" because there is another therefore just in the previous sentence
23. I would write: "To avoid overreliance on a single cell line (i.e., HeLa), we generated a second model, GIV-depleted MDA MB-231 cells (using CRISPR/Cas-mediated genome editing, see Materials and Methods)"
24. (76%; 1437 proteins, including EGFR)
25. In this sentence "The interactions between the DEPs were fetched from the STRING database to build a PPI network, which showed major coat proteins (AP1, AP2, COP, and CAV), monomeric GTPases (Arfs, Rabs, Rho, CDC42 and Rac1) and trimeric GTPases (GNAI) (Figure 7B)" perhaps "PPI network, in which we found several major coat proteins..." sounds better
26. I do not know what a "degree of connectivity analysis" is. I would write simply "A connectivity analysis revealed"
27. Here I would add the hyphen between DEP and derived and would use the plural for network, like this: " Visualization of the DEP-derived PPI networks as multi-layered networks"
28. Why is this written like this: "Eph-ephrin system"? I saw it written rather like this: "Eph/ephrin system"
29. I did not notice this before, but re-reading the paper now it occurred to me that this sentence is a little weird: "The major discovery we report here is the creation of an experimentally constrained multi-timescale model for cell survival that relies on growth factor-responsive cell secretion.". A creation is not a discovery. Perhaps one could write it like this: "The major novelty of our work is the creation" or "We report here an experimentally-constrained.."
30. I would delete "of" from this sentence: "The insights and models derived from this study are expected to inform and impact at least three fields, i.e., of signal transduction, cell secretion, and cancer cell biology in the following ways."
31. I think the article here should not be used: "don't apply to the multicellular eukaryotes". I would write: "don't apply to multicellular eukaryotes"
32. In Limitations of the study, first sentence, I would delete the comma: "The multi-timescale model we built ignores the spatial aspects of the various feedback control loops."
33. Here a parenthesis is missing after peptides: "(e.g., specific point mutants of GIV, GEF or GAPs or perturbagens such as small molecule or peptides."
34. I am not sure what the authors mean with this sentence: "We chose to model to test the experimentally determined key components by design". Do they mean "We chose to use mathematical modelling to test" or is one of the two verbs superfluous, that is, one should write either "we chose to model the experimentally..." or "we chose to test the...". Please correct accordingly.
35. In the legend to Figure 4, I would change the title like this: "Predicted impact of coupled switches on the degree of alignment of endomembrane responses to the dose of extracellular stimulus, cellular processes, and fate." Albeit this is a very long title for a figure. Could simply ""Predicted impact of coupled switches" do? I just noticed that also the titles of figs. 2 and 3 are rather long. I sugges the authors try to make shorter titles that reveal the main message of the figure without giving all the details
36. The title of figure 5 sounds strange: "Coupled GTPases are predicted to support secrete-and-sense autonomy and for the maintenance of cell number." I suggest this: "Coupled GTPases are predicted to support secrete-and-sense autonomy and maintenance of cell number"

Reviewer #2:

The authors have done a good job in addressing most of my comments. The presentation is now clearer and the work is thus now easier to understand compared to the previous version. I support publication of this interesting work in MSB.

Congratulations to the authors.

Reviewer #3:

The revised manuscript by the authors has several improvements. Notably, the authors have toned down their language in several key areas. (i.e. in the original submission, the authors claimed this study would "spur new paradigms" in at least three new fields, now they state that the work could "inform and impact" at least three new fields.) The reduction of hyperbolic language is an improvement. The authors also clarify several key aspects of their manuscript, and the adjustments to figures

seem to improve (already well-done) figures.

However, this reviewer does not see this manuscript as a satisfying complete body of work. To this reviewer, the body of work feels incomplete - a collection of observations and models that are claimed to do and show more than is shown. Models can be helpful, but a model without sufficient validation is not a major advance. The authors claims of deeper understandings through the model are not well-supported by experiments that probe the model. Indeed, the authors appear to be aware of this in their limitations section where the authors make statements that attempt to explain away experimental validation that, unfortunately, ring untrue.

The first such statement that the authors make to argue against doing a proper experimental validation is: "selective disruption of various connections within the Golgi-localized circuit is not possible currently due to the lack the experimental tools (e.g., specific point mutants of GIV, GEF or GAPs or perturbagens such as small molecule or peptides." This rings untrue for several reasons. First, the making of mutants has been a central aspect of biochemical studies of signaling pathways and G-protein biology for more than four decades. Research does not halt because one does not have a reagent that can reasonably be created by a competent practitioner of the field (which these authors are, as demonstrated by their previous experimental work on the subject). Second, structures are shown by the authors in their figures. Structural information can greatly assist with the process of identifying useful mutations, as one can reasonably infer key contacts and which residues if mutated/deleted could disrupt an interaction. Experiments can then test if these insights about breaking interactions are true, and then the constructs can be used for real experiments that validate the circuit. Additionally, the structure seems to support a structure where the domains that bind ARF and GNAI are distinct. Fragments of GIV may potentially bind only one of the two binding partners (ARF or GNAI) and be capable of serving as a mechanism to disrupt interacitons. (This reviewer completely appreciates that a comprehensive mutant/biochemical study of GIV and this system may be out of scope. For example, a full biochemical dissection of the domians of GIV with various mutations may be a body of work worthy of a separate manuscript. Nevertheless, a gap in what is currently possible and/or what the authors are currently willing to do or share does not allow a computational model to be assumed valid. Validation is done with experimental data, and a lack of data means the model's presented insights are not validated.)

Moreover, the revision (which includes many excellent new changes for communication) has a new synopsis image with Highlights/Main Findings. All four of the main findings focus on "coupling" (Coupling is the first word of three of the four). Disrupting coupling is claimed to be a therapeutic strategy. But the authors only disruption of coupling is knocking out the protein that couples - and this protein could have other effects, other protein binding partners, etc. The knockout of GIV with a forced co-localization of ARF1 and GNAI for the coupling "add-back" would be another possible experiment that could help validate the coupling hypothesis. As it is, the major findings are not supported by the work.

The second statement in the limitations section that highlights a fundamental issue with a major theme of the manuscript is: "Conducting experiments across the full range of stimuli to assess 'proportionality/linearity' of response was possible in some instance (e.g., cell survival) but not possible in others (e.g., FRET, Arf1 activity, etc.) due to technical limitations of the assays and/or detection thresholds." The linearity/proporitionality is a major theme of this manuscript. The authors cannot claim to show this as a result if they do not show it experimentally. Any segment of a continuous line looks linear for some length. Almost any model can look linear/proportional for some set of parameters and some range of inputs. Thus to make a claim, and not be able to experimentally test it, seems more like theoretical biology than systems biology. Cell survival is additionally far downstream so its linearity/proportionality or lack thereof is not a good readout of this model. The survival experiments are additionally problematic - the authors clearly show that knockdown of GIV makes the cells unhappy (more apoptosis/more necrosis) and that this is reversed with additional serum. But that does not prove that this is due to the circuit - the author assume this, but do not show this or support this well. There could be other effects of GIV that impact growth factor signaling and cell survival.

The major addition in the revision is Figure 7. This figure does not support the key conclusions of the study (coupling, linearity/proportionality) or the model(s), but is rather additional data that increases the bulk of the manuscript. The proteomic study may have some additional problems... if GIV K/O causes more apoptosis and more necrosis, could that be confounding the comparisons? i.e. does GIV K/O just disrupt the normal ability to respond to the stressor? Additionally, should the insights from the proteomics somehow be validated? This addition does not solve the core problems of the manuscript.

Other notes:
In the abstract, the authors state "Such behavior translates into cell survival that is self-sustained by stimulus-proportionate secretion". However, the term "proportionate" and "proportional" do not appear in research results, only abstract, introduction, discussion, limitations of study - and a cartoon in Fig 6C. If this is a big part of the study, the authors need to clarify where it is in the text and how they are proving it. This example reinforces the use of systems-biology jargon and buzz without the substance to back it up.

The authors also state in the abstract "convert the expected switch-like behaviorof Arf1-dependent secretion into an unexpected dose response alignment behavior of sensing and secretion. Such behavior translates into cell survival that is self-sustained by stimulus-proportionate secretion." The "expected switch-like beahvior of Arf1" is a bit of a straw man argument. Why is switchlike behavior expected? In the systems biology literature, the nonlinear switchlike behavior is a popular feature to focus upon, and it

has been projected onto many systems - but is it biologically expected? Why should Arf1 signaling be switchlike? Having a model that is switchlike does not make it expected; alternative parameterizations could make it linear for some region of inputs. This seems to all be speculation.

## Response to editor

1. Provide up to five keywords and incorporate them into the manuscript text.

**Response:** We have added five keywords after Abstract.

2. Remove the Author contribution section from the manuscript file.

**Response:** We have removed the Author contribution section.

3. We updated our journal's competing interests policy in January 2022 and requested authors to consider both actual and perceived competing interests. Please review the policy https://www.embopress.org/competing-interests and add your competing interests if necessary. Please add a disclosure statement using the heading "Disclosure statement and competing interests".

**Response:** We have reviewed the updated policies of MSB and have confirmed that there are no conflicts of interests to declare, either real or perceived.

4. Funding information: please make sure the finding information entered in the online submission system and the manuscript file are consistent. Currently, only grant number CA238042 is entered into the submission system.

**Response:** We have inserted all the grants into the portal/system.

5. Appendix file: please add a Table of Content (including page numbers) to the 1st page.

**Response:** In the appendix file, we have added a table of content that includes the page numbers to the 1st page.

6. Datasets EV1 and EV2 should be uploaded as two separate excel tables with the legend inserted in a separate tab within the same excel file. There is no need for zipping the legends and table.

**Response:** For Datasets EV1 and EV2, we have put the legend in the excel table.

7. Table EV1: please provide the table in xls. or txt format and insert the legend in a separate tab-no need for zipping the legend and table.

**Response:** For Table EV1, we have created an excel file including the table and legend.

8. Data availability: please make sure the datasets will be made publicly available upon the acceptance of the manuscript.

**Response:** In DATA AVAILABILITY section, the code in the Github is publicly available, and we also confirmed that the proteomics dataset associated with this manuscript (as below) is now requested to be publicly released. We expect this to take effect in the next 24-48 hours.

- Project Name: Growth Signaling Autonomy in Circulating Tumor Cells Aids Metastatic Seeding
- Project accession: PXD037253

9. Figure EV5D - shGV - There is a drawn band added to the figure to highlight the differences between shGV. Please remove this artistic band as it interferes with the western blots.

**Response:** We have removed the band in Figure EV5D.

10. I have slightly modified the synopsis text (see attached). Please review the changes and comment and let me know if you would like to introduce further modifications.

**Response:** Thank you for doing this. We are pleased with the edits and see that it is improved. We have addressed the Editor's comment/question. We have no further changes to suggest.

11. Our data editors have seen the manuscript and made some comments and suggestions that need to be addressed (see attached file). Please send back a revised version (in track change mode), as we will need to go through the changes.

**Response:** We have gone over these edits and found them very helpful to revise the work accordingly.

## Response to Reviewer #1:

1. In figure 1A, I suggest to swap tGEF and GIV so that GIV is above and tGEF below in squared brackets. All in all, Arf1 and Galphai, that is, the name of the protein, is written above, and their molecular identity (GTPase) is written below in squared brackets. I would keep the same format. There are some other panels in main and supplementary figures where this nomenclature appears as well, it might be good to unify them.

**Response:** We appreciate this suggestion for consistency. We have now changed in all other places: GIV and tGEF in Figure 1,2,3, Figure EV1, Appendix S3, and Movie EV1.

2. Figure 2E, for the 3D plots, I noticed only now that there is some sort of messy white blob on top on the Y-axis below 0. I assume these are numbers just are simply so close together that they become unreadable and look like that. Perhaps there is a way to cut the plots so that this effect is not there anymore.

**Response:** The panels in question were raw (uneditable) ImageJ outputs, and we had used them as is, without any changes at all, hence the y axis that was collapsed and not relevant, had axis labels (numbers) all on top of each other. The suggestions to remove what is not relevant is a good one, and that is what we have done now. We thank the editors for this suggestion.

3. I know that the paper is already full of data and figures. Nonetheless, I believe it is common practice nowadays to show in the supplement the full membranes used for the WB that are shown cropped in the main text. I would also like to see the Poinceau stained membrane in full, given the explanation the authors gave me as why this is better than detection via anti-GST antibody. It is a fair point, but the authors so far show only a small portion of the membrane, thus the reader cannot judge if there are truncated bands etc.

**Response:** We had provided full-length Ponceau S-stained membrane as **Source data for Figure 2B**. As one can see in that file, the GST GGA1 GAT domain is only seen as **full-length protein with no breakdowns**. It is possible that the reviewer did not get a chance to see it, and we certainly missed out on an opportunity to call attention to this matter during the last cycle of revision.

4. Figure 6E: for consistency I would either write apoptosis / necrosis, or apoptotic cells / necrotic cells. At the moment the authors use a hybrid: apoptosis / necrotic cells

**Response:** We thank the reviewer for picking up this issue. We agree. We have converted it to achieve consistency.

About language.

Since the authors changed a large portion of the text, I found several places in which I believe the language could be improved:

**Response:** We appreciate all these thorough and thoughtful edits and we have implemented them all.

1. Abstract: growth factors and stimuli are plural nouns, thus the "a" should not be there in the sentence: growth factors are key stimuli"

**Response:** We agree. We have revised as the reviewer suggested.

2. Introduction, very first sentence. I would add a comma after autonomy and I would write "to have been clearly defined" instead of "to be clearly defined"

**Response:** We agree. We have revised as the reviewer suggested.

3. I find the terminology "membrane of extracellular space" odd. The extracellular space has no membrane, but "of" indicates the membrane belongs to it. How about writing simply "rooted to the cell membrane"?

Response: We agree. We have changed the "membrane of extracellular space" to "cell membrane" as the reviewer suggested. We believe there was a typo in the suggestion because the reviewer may have meant “routed” instead of “rooted”.

4. In this sentence, the verb should be plural: "the secretory functions of the Golgi apparatus require the unlikely coupling"

**Response:** We agree. We have revised as the reviewer suggested.

5. Right after, the article is missing: "one is the small or monomeric (m) GTPase Arf1". When writing the name of the protein I would not use the article, e.g. Arf1 is a small GTPase, but when writing the type of protein followed by the name I would use the article. Please correct this throughout the text.

**Response:** We agree. We have revised as the reviewer suggested and corrected other articles throughout the text.

6. I find this sentence confusing: "One negative feedback loop is that GIV can improve the GAP for Arf1---ArfGAP2/3, thus terminating Arf1 signaling", I suggest to re-phrase like this: "One negative feedback loop involves the activation of the GAP for Arf1 (ArfGAP2/3) by GIV, which terminates Arf1 signaling"

**Response:** We agree. We have revised as the reviewer suggested.

7. I personally do not like this title: "An integrated systems and experimental approach to dissect a Golgi-localized GTPase circuit" because one speaks of systems biology but not systems approach. How about "An integrated computational and experimental approach to dissect a Golgi-localized GTPase circuit"?

**Response:** We agree. We have revised as the reviewer suggested.

8. Same here, I do not like the terminology "a dynamical systems model". "A mathematical model based on ODEs" would be better.

**Response:** We agree. We have changed "a dynamical systems model" to "mathematical model" or "mathematical modeling" throughout the text.

I would write like this: "We chose two different cancer cell lines to conduct the experiments: cervical (HeLa) and breast (MDA-MB231) cancer cell lines"

**Response:** We agree. We have revised as the reviewer suggested.

9. There is something strange in this sentence: "Elevated expression of GIV has also been reported in a variety of solid tumors (Garcia-Marcos et al., 2015; Getz et al., 2019), both in primary tumors (Ghosh, 2015; Ghosh et al., 2016b) as well as in circulating tumor cells (Barbazan et al., 2016; Dunkel et al., 2018) have been shown to correlate withtumor aggressiveness and poor survival across cancers." Perhaps simply an "and" and the verb in the singular form would do the job: "elevated expression ...has been reported.... and has been shown to...".

**Response:** We agree. It was a missing ‘and’ and ‘verb’, and we have changed as the reviewer suggested.

10. The paragraph starting with: "Finally, model and PPI network-driven predictions" seems to be separated from the previous paragraph, in which an explanation is given about why those two cell lines were chosen. I would put it in a new paragraph.

**Response:** We agree. We have revised as the reviewer suggested.

11. "The Epidermal growth factor (EGF) was prioritized" : the article was missing

**Response:** We agree. We have revised as the reviewer suggested.

12. "revealing the structural basis of the recruitment of GIV by active Arf1" not the structure basis

**Response:** We agree. We have changed "structure" to "structural" as the reviewer suggested.

13. „GIV-depleted cells were obtained using a short hairpin RNA to target GIV". From the methods I gather it is only one RNA, so add the article "a"

**Response:** We agree. We have revised as the reviewer suggested.

14. Re-phrase like this: "10% serum (containing a mixture of growth factors)"

**Response:** We agree. We have revised as the reviewer suggested.

15. Add the article: "The findings confirmed that Gi is activated on ..."

**Response:** We agree. We have revised as the reviewer suggested.

16. Change to this: "We fitted the above experimental data by tuning the kinetic parameters"

**Response:** We agree. We have revised as the reviewer suggested.

17. Change to this: "Thus, the model matched the overall trend of the experimental data in both cells"

**Response:** We agree. We have revised as the reviewer suggested.

18. I would re-phrase like this: " We conducted two simulations: one in which we decreased the GIV level to simulate the Arf1 activation dynamics in the shGIV cell line (red line in Figure 3J), and one in which we deleted either arrows 2 or 3 to simulate the Arf1 and Gi activation dynamics for the uncoupled GTPase switches."

**Response:** We agree. We have revised as the reviewer suggested.

19. "We also tested the 'OR' logic for the negative feedback (Figure EV3) and found the model predictions to be indistinguishable from those obtained with the AND gate."

**Response:** We agree. We have revised as the reviewer suggested.

20. I personally would not talk of "efficiency" of the model, but rather the ability of the model to capture the experimental data

**Response:** We agree. We have revised as the reviewer suggested.

21. I do not understand this: "Typically, dose response alignment (DoRA), referring to the close match of the receptor occupancy and the downstream molecules under different stimuli"... please re-phrase to explain better

**Response:** We agree. To make it clear, we have re-phrase this sentence, which is shown as follows: Typically, dose-response alignment (DoRA), referring to the close match of the receptor occupancy and the downstream response no matter what the stimuli level is (Andrews et al., 2016), is believed to improve information transduction.

22. I would delete the therefore from this sentence: " Therefore, by using the model that has been fitted to the data" because there is another therefore just in the previous sentence

**Response:** We agree. We have revised as the reviewer suggested.

23. I would write: "To avoid overreliance on a single cell line (i.e., HeLa), we generated a second model, GIV-depleted MDA MB-231 cells (using CRISPR/Cas-mediated genome editing, see Materials and Methods)"

**Response:** We agree. We have revised as the reviewer suggested.

24. (76%; 1437 proteins, including EGFR)

**Response:** We agree. We have revised as the reviewer suggested.

25. In this sentence "The interactions between the DEPs were fetched from the STRING database to build a PPI network, which showed major coat proteins (AP1, AP2, COP, and CAV), monomeric GTPases (Arfs, Rabs, Rho, CDC42 and Rac1) and trimeric GTPases (GNAI) (Figure 7B)" perhaps "PPI network, in which we found several major coat proteins..." sounds better

**Response:** We agree. We have revised as the reviewer suggested.

26. I do not know what a "degree of connectivity analysis" is. I would write simply "A connectivity analysis revealed"

**Response:** We agree. We have revised as the reviewer suggested.

27. Here I would add the hyphen between DEP and derived and would use the plural for network, like this: " Visualization of the DEP-derived PPI networks as multi-layered networks"

**Response:** We agree. We have revised as the reviewer suggested.

28. Why is this written like this: "Eph-ephrin system"? I saw it written rather like this: "Eph/ephrin system"

**Response:** We agree. We have revised as the reviewer suggested.

29. I did not notice this before, but re-reading the paper now it occurred to me that this sentence is a little weird: "The major discovery we report here is the creation of an experimentally constrained multi-timescale model for cell survival that relies on growth factor-responsive cell secretion.". A creation is not a discovery. Perhaps one could write it like this: "The major novelty of our work is the creation" or "We report here an experimentally-constrained.."

**Response:** We agree. We have revised as the reviewer suggested.

30. I would delete "of" from this sentence: "The insights and models derived from this study are expected to inform and impact at least three fields, i.e., of signal transduction, cell secretion, and cancer cell biology in the following ways."

**Response:** We agree. We have revised as the reviewer suggested.

31. I think the article here should not be used: "don't apply to the multicellular eukaryotes". I would write: "don't apply to multicellular eukaryotes"

**Response:** We agree. We have revised as the reviewer suggested.

32. In Limitations of the study, first sentence, I would delete the comma: "The multi-timescale model we built ignores the spatial aspects of the various feedback control loops."

**Response:** We agree. We have revised as the reviewer suggested.

33. Here a parenthesis is missing after peptides: "(e.g., specific point mutants of GIV, GEF or GAPs or perturbagens such as small molecule or peptides."

**Response:** We agree. We have revised as the reviewer suggested.

34. I am not sure what the authors mean with this sentence: "We chose to model to test the experimentally determined key components by design". Do they mean "We chose to use mathematical modelling to test" or is one of the two verbs superfluous, that is, one should write either "we chose to model the experimentally..." or "we chose to test the...". Please correct accordingly.

**Response:** We agree. We have revised as the reviewer suggested.

35. In the legend to Figure 4, I would change the title like this: "Predicted impact of coupled switches on the degree of alignment of endomembrane responses to the dose of extracellular stimulus, cellular processes, and fate." Albeit this is a very long title for a figure. Could simply ""Predicted impact of coupled switches" do? I just noticed that also the titles of figs. 2 and 3 are rather long. I sugges the authors try to make shorter titles that reveal the main message of the figure without giving all the details

**Response:** We agree. We have now shortened the title of Figure 4 to "Predicted impact of uncoupling the coupled switches". The title of Figure 2 has been shortened to "EGF activates Arf1 (mG*) and triggers the recruitment of GIV-GEM on Golgi".

36. The title of figure 5 sounds strange: "Coupled GTPases are predicted to support secrete-and-sense autonomy and for the maintenance of cell number." I suggest this: "Coupled GTPases are predicted to support secrete-and-sense autonomy and maintenance of cell number"

**Response:** We agree. We have revised as the reviewer suggested.

## REFERENCES CITED

Andrews, S.S., Peria, W.J., Yu, R.C., Colman-Lerner, A., and Brent, R. (2016). Push-Pull and Feedback Mechanisms Can Align Signaling System Outputs with Inputs. *Cell Systems* 3, 444-455.e442.

## 2nd Revision - Editorial Decision 7th Feb 2023

Thank you again for sending us your revised manuscript. We are now satisfied with the modifications made and I am pleased to inform you that your paper has been accepted for publication.

## EMBO Press Author Checklist


| | 
|---|
| Corresponding Author Name: Pradipta Ghosh |
| Journal Submitted to: Molecular Systems Biology |
| Manuscript Number: MSB-2022-11127R |


**USEFUL LINKS FOR COMPLETING THIS FORM**
The EMBO Journal - Author Guidelines
EMBO Reports - Author Guidelines
Molecular Systems Biology - Author Guidelines
EMBO Molecular Medicine - Author Guidelines

**Reporting Checklist for Life Science Articles (updated January**
This checklist is adapted from Materials Design Analysis Reporting (MDAR) Checklist for Authors. MDAR establishes a minimum set of requirements in transparent reporting in the life sciences (see Statement of Task: 10.31222/osf.io/9sm4x). Please follow the journal's guidelines in preparing your
**Please note that a copy of this checklist will be published alongside your article.**

**Abridged guidelines for figures**
**1. Data**
The data shown in figures should satisfy the following conditions:
- the data were obtained and processed according to the field's best practice and are presented to reflect the results of the experiments in an accurate and unbiased manner.
- ideally, figure panels should include only measurements that are directly comparable to each other and obtained with the same assay.
- plots include clearly labeled error bars for independent experiments and sample sizes. Unless justified, error bars should not be shown for technical
- if n<5, the individual data points from each experiment should be plotted. Any statistical test employed should be justified.
- Source Data should be included to report the data underlying figures according to the guidelines set out in the authorship guidelines on Data

**2. Captions**
Each figure caption should contain the following information, for each panel where they are relevant:
- a specification of the experimental system investigated (eg cell line, species name).
- the assay(s) and method(s) used to carry out the reported observations and measurements.
- an explicit mention of the biological and chemical entity(ies) that are being measured.
- an explicit mention of the biological and chemical entity(ies) that are altered/varied/perturbed in a controlled manner.
- the exact sample size (n) for each experimental group/condition, given as a number, not a range;
- a description of the sample collection allowing the reader to understand whether the samples represent technical or biological replicates (including how many animals, litters, cultures, etc.).
- a statement of how many times the experiment shown was independently replicated in the laboratory.
- definitions of statistical methods and measures:
  - common tests, such as t-test (please specify whether paired vs. unpaired), simple $\chi^2$ tests, Wilcoxon and Mann-Whitney tests, can be unambiguously identified by name only, but more complex techniques should be described in the methods section;
  - are tests one-sided or two-sided?
  - are there adjustments for multiple comparisons?
  - exact statistical test results, e.g., P values = x but not P values < x;
  - definition of 'center values' as median or average;
  - definition of error bars as s.d. or s.e.m.

**Please complete ALL of the questions below.**
**Select "Not Applicable" only when the requested information is not relevant for your study.**

**Materials**

| Newly Created Materials | Information included in the manuscript? | In which section is the information available? (Reagents and Tools Table, Materials and Methods, Figures, Data Availability Section) |
|---|---|---|
| New materials and reagents need to be available; do any restrictions apply? | Yes | No restrictions. Reagents and Tools Table lists datasets, materials and reagents uniquely created in this work, all of which are available under the Regents of the University of California's material transfer poicy |

| Antibodies | Information included in the manuscript? | In which section is the information available? (Reagents and Tools Table, Materials and Methods, Figures, Data Availability Section) |
|---|---|---|
| For **antibodies** provide the following information:<br>- Commercial antibodies: RRID (if possible) or supplier name, catalogue number and or/clone number<br>- Non-commercial: RRID or citation | Yes | Reagents and Tools Table |

| DNA and RNA sequences | Information included in the manuscript? | In which section is the information available? (Reagents and Tools Table, Materials and Methods, Figures, Data Availability Section) |
|---|---|---|
| **Short novel DNA or RNA including primers, probes**: provide the sequences. | Yes | Reagents and Tools Table |

| Cell materials | Information included in the manuscript? | In which section is the information available? (Reagents and Tools Table, Materials and Methods, Figures, Data Availability Section) |
|---|---|---|
| **Cell lines:** Provide species information, strain. Provide accession number in repository **OR** supplier name, catalog number, clone number, and/**OR** RRID. | Yes | Reagents and Tools Table |
| **Primary cultures:** Provide species, strain, sex of origin, genetic modification status. | Not Applicable | |
| Report if the cell lines were recently **authenticated** (e.g., by STR profiling) and tested for mycoplasma contamination. | Yes | Materials and Methods section |

| Experimental animals | Information included in the manuscript? | In which section is the information available? (Reagents and Tools Table, Materials and Methods, Figures, Data Availability Section) |
|---|---|---|
| **Laboratory animals or Model organisms:** Provide species, strain, sex, age, genetic modification status. Provide accession number in repository **OR** supplier name, catalog number, clone number, **OR** RRID. | Not Applicable | n/a |
| **Animal observed in or captured from the field:** Provide species, sex, and age where possible. | Not Applicable | n/a |
| Please detail **housing and husbandry conditions**. | Not Applicable | n/a |

| Plants and microbes | Information included in the manuscript? | In which section is the information available? (Reagents and Tools Table, Materials and Methods, Figures, Data Availability Section) |
|---|---|---|
| **Plants:** provide species and strain, ecotype and cultivar where relevant, unique accession number if available, and source (including location for collected wild specimens). | Not Applicable | n/a |
| **Microbes:** provide species and strain, unique accession number if available, and source. | Not Applicable | n/a |

| Human research participants | Information included in the manuscript? | In which section is the information available? (Reagents and Tools Table, Materials and Methods, Figures, Data Availability Section) |
|---|---|---|
| If collected and within the bounds of privacy constraints report on age, sex and gender or ethnicity for all study participants. | Not Applicable | n/a |

| Core facilities | Information included in the manuscript? | In which section is the information available? (Reagents and Tools Table, Materials and Methods, Figures, Data Availability Section) |
|---|---|---|
| If your work benefited from core facilities, was their service mentioned in the acknowledgments section? | Yes | Methods section |

**Design**

| Study protocol | Information included in the manuscript? | In which section is the information available? (Reagents and Tools Table, Materials and Methods, Figures, Data Availability Section) |
|---|---|---|
| If study protocol has been **pre-registered, provide DOI in the manuscript**. For clinical trials, provide the trial registration number **OR** cite DOI. | Not Applicable | |
| Report the **clinical trial registration number** (at ClinicalTrials.gov or equivalent), where applicable. | Not Applicable | |

| Laboratory protocol | Information included in the manuscript? | In which section is the information available? (Reagents and Tools Table, Materials and Methods, Figures, Data Availability Section) |
|---|---|---|
| Provide DOI OR other citation details if **external detailed step-by-step protocols** are available. | Yes | Materials and Methods section |

| Experimental study design and statistics | Information included in the manuscript? | In which section is the information available? (Reagents and Tools Table, Materials and Methods, Figures, Data Availability Section) |
|---|---|---|
| Include a statement about **sample size** estimate even if no statistical methods were used. | Not Applicable | Statistical analyses in experimental studies and replication |
| Were any steps taken to minimize the effects of subjective bias when allocating animals/samples to treatment (e.g. **randomization procedure**)? If yes, have they been described? | Not Applicable | |
| Include a statement about **blinding** even if no blinding was done. | Not Applicable | *Statistical analyses in experimental studies and replication* |
| Describe **inclusion/exclusion criteria** if samples or animals were excluded from the analysis. Were the criteria pre-established?<br><br>If sample or data points were omitted from analysis, report if this was due to attrition or intentional exclusion and provide justification. | Not Applicable | |
| For every figure, are **statistical tests** justified as appropriate? Do the data meet the assumptions of the tests (e.g., normal distribution)? Describe any methods used to assess it. Is there an estimate of variation within each group of data? Is the variance similar between the groups that are being statistically compared? | Yes | |

| Sample definition and in-laboratory replication | Information included in the manuscript? | In which section is the information available? (Reagents and Tools Table, Materials and Methods, Figures, Data Availability Section) |
|---|---|---|
| In the figure legends: state number of times the experiment was **replicated** in laboratory. | Yes | Figure legends for respective figures. |
| In the figure legends: define whether data describe **technical or biological replicates**. | Yes | Statistical analyses in experimental studies and replication |

## Ethics

| Ethics | Information included in the manuscript? | In which section is the information available? (Reagents and Tools Table, Materials and Methods, Figures, Data Availability Section) |
|---|---|---|
| Studies involving **human participants**: State details of **authority granting ethics approval** (IRB or equivalent committee(s), provide reference number for approval. | Not Applicable | |
| Studies involving **human participants**: Include a statement confirming that **informed consent** was obtained from all subjects and that the experiments conformed to the principles set out in the WMA Declaration of Helsinki and the Department of Health and Human Services Belmont Report. | Not Applicable | |
| Studies involving **human participants:** For publication of **patient photos**, include a statement confirming that consent to publish was obtained. | Not Applicable | |
| Studies involving experimental **animals**: State details of **authority granting ethics approva**l (IRB or equivalent committee(s), provide reference number for approval. Include a statement of compliance with ethical regulations. | Not Applicable | |
| Studies involving **specimen and field samples:** State if relevant **permits** obtained, provide details of authority approving study; if none were required, explain why. | Not Applicable | |

| Dual Use Research of Concern (DURC) | Information included in the manuscript? | In which section is the information available? (Reagents and Tools Table, Materials and Methods, Figures, Data Availability Section) |
|---|---|---|
| Could your study fall under dual use research restrictions? Please check biosecurity documents and list of **select agents and toxins** (CDC): https://www.selectagents.gov/sat/list.htm | Not Applicable | |
| If you used a select agent, is the security level of the lab appropriate and reported in the manuscript? | Not Applicable | |
| If a study is subject to dual use research of concern regulations, is the name of the **authority granting approval and reference number** for the regulatory approval provided in the manuscript? | Not Applicable | |

## Reporting

The MDAR framework recommends adoption of discipline-specific guidelines, established and endorsed through community initiatives. Journals have their own policy about requiring specific guidelines and recommendations to complement MDAR.

| Adherence to community standards | Information included in the manuscript? | In which section is the information available? (Reagents and Tools Table, Materials and Methods, Figures, Data Availability Section) |
|---|---|---|
| State if relevant guidelines or checklists (e.g., **ICMJE, MIBBI, ARRIVE, PRISMA**) have been followed or provided. | Not Applicable | |
| For **tumor marker prognostic studies,** we recommend that you follow the **REMARK** reporting guidelines (see link list at top right). See author guidelines, under 'Reporting Guidelines'. Please confirm you have followed these guidelines. | Not Applicable | |
| For **phase II and III randomized controlled trials**, please refer to the **CONSORT** flow diagram (see link list at top right) and submit the CONSORT checklist (see link list at top right) with your submission. See author guidelines, under 'Reporting Guidelines'. Please confirm you have submitted this list. | Not Applicable | |

## Data Availability

| Data availability | Information included in the manuscript? | In which section is the information available? (Reagents and Tools Table, Materials and Methods, Figures, Data Availability Section) |
|---|---|---|
| Have **primary datasets** been deposited according to the journal's guidelines (see 'Data Deposition' section) and the respective accession numbers provided in the Data Availability Section? | Yes | Data Availability Section |
| Were **human clinical and genomic datasets** deposited in a public access-controlled repository in accordance to ethical obligations to the patients and to the applicable consent agreement? | Not Applicable | |
| Are **computational models** that are central and integral to a study available without restrictions in a machine-readable form? Were the relevant accession numbers or links provided? | Yes | Data Availability Section |
| If publicly available data were reused, provide the respective **data citations in the reference list**. | Not Applicable | |
